# Financing for equity for women’s, children’s and adolescents’ health in low- and middle-income countries: A scoping review

**DOI:** 10.1371/journal.pgph.0003573

**Published:** 2024-09-12

**Authors:** Lama Bou-Karroum, Domenico G. Iaia, Fadi El-Jardali, Clara Abou Samra, Sabine Salameh, Zeina Sleem, Reem Masri, Aya Harb, Nour Hemadi, Nadeen Hilal, Layal Hneiny, Sahar Nassour, Mehr Gul Shah, Etienne V. Langlois

**Affiliations:** 1 Faculty of Health Sciences, Department of Health Management and Policy, American University of Beirut, Beirut, Lebanon; 2 Knowledge to Policy (K2P) Center, American University of Beirut, Beirut, Lebanon; 3 Partnership for Maternal Newborn and Child Health, World Health Organisation, Geneva, Switzerland; 4 Department of Health Research Methods, Evidence and Impact (HEI), McMaster University, Hamilton, Ontario, Canada; 5 Department of Internal Medicine, Ain Wazein Medical Village, Ain Wazein, Lebanon; 6 Saab Medical Library, American University of Beirut, Beirut, Lebanon; Indian Institute of Technology Bombay, INDIA

## Abstract

Over the past few decades, the world has witnessed considerable progress in women’s, children’s and adolescents’ health (WCAH) and the Sustainable Development Goals (SDGs). Yet deep inequities remain between and within countries. This scoping review aims to map financing interventions and measures to improve equity in WCAH in low- and middle-income countries (LMICs). This scoping review was conducted following Joanna Briggs Institute (JBI) guidance for conducting such reviews as well as the PRISMA Extension for Scoping Reviews (PRISMA-ScR) for reporting scoping reviews. We searched Medline, PubMed, EMBASE and the World Health Organization’s (WHO) Global Index Medicus, and relevant websites. The selection process was conducted in duplicate and independently. Out of 26 355 citations identified from electronic databases, relevant website searches and stakeholders’ consultations, 413 studies were included in the final review. Conditional cash transfers (CCTs) (22.3%), health insurance (21.4%), user fee exemptions (18.1%) and vouchers (16.9%) were the most reported financial interventions and measures. The majority were targeted at women (57%) and children (21%) with others targeting adolescents (2.7%) and newborns (0.7%). The findings highlighted that CCTs, voucher programs and various insurance schemes can improve the utilization of maternal and child health services for the poor and the disadvantaged, and improve mortality and morbidity rates. However, multiple implementation challenges impact the effectiveness of these programmes. Some studies suggested that financial interventions alone would not be sufficient to achieve equity in health coverage among those of a lower income and those residing in remote regions. This review provides evidence on financing interventions to address the health needs of the most vulnerable communities. It can be used to inform the design of equitable health financing policies and health system reform efforts that are essential to moving towards universal health coverage (UHC). By also unveiling the knowledge gaps, it can be used to inform future research on financing interventions and measures to improve equity when addressing WCAH in LMICs.

## Background

Over the past few decades, and prior to the onset of the COVID-19 pandemic, the world witnessed considerable progress in WCAH and well-being and the SDGs. The number of maternal deaths worldwide decreased to 223 000 in 2020 from 342 000 in 2000 [1], and the mortality rate for children under-five years decreased by almost 50% between 2000 and 2021 [2]. Yet deep inequities remain between and within countries. For instance, although the global maternal mortality ratio is estimated to have fallen by 34% between 2000 and 2020, 94% of all maternal deaths occur in LMICs with the risk of stillbirth being 23 times higher in the most severely affected countries [3].

Inequities are also pronounced within countries where progress is not reaching every woman, adolescent and child, especially those in population groups facing multiple deprivations. These groups are often found in settings that are remote, rural, urban, conflict-affected or mobile. They are bearing the disproportionate burden of death and are being left furthest behind. For instance, two-thirds of zero-dose children (those who have not received a single dose of the DTP-containing vaccine, which protects against diphtheria, tetanus and polio) live below the poverty line and suffer nearly 50% of global deaths from vaccine-preventable diseases. Similarly, maternal mortality increase on average by 11% in conflict zones and by 28% in the worst hit conflicted affected areas [4]. Similarly, while significant reductions have been achieved in under-five mortality rates, progress on newborn mortality and stillbirths has fallen behind. It is estimated that 47% of under-five deaths occur during the neonatal period [5]. Factors such as age, gender, ethnicity, sexual orientation, migration status, socio-economic status and geographic location contribute to the important inequities across the continuum of sexual, reproductive, maternal, neonatal, child and adolescent health (SRMNCAH).

The COVID-19 pandemic has exacerbated these existing inequities and compounded the difficulties that women, children and adolescents face in accessing health and social services, negatively impacting SRMNCAH outcomes. For instance, recent estimates provided by the United Nations Population Fund indicate that across 115 LMICs, the pandemic disrupted contraceptive use for approximately 12 million women, causing nearly 1.4 million unintended pregnancies in 2020 [6]. Findings from a Lancet systematic review also indicate that maternal deaths and stillbirths increased during the pandemic, as did ruptured ectopic pregnancies and maternal depression. There seemed to be a considerable disparity between high-resource and low-resource settings [7]. In 2020, the number of children who missed out on receiving even a single vaccine shot increased by 3 million, from 10.6 million in 2019 to 13.7 million in 2020, in Gavi-supported countries. Zero-dose children and the communities they live in often face multiple deprivations and can be regarded as a marker of inequity [8]. For example, in Nepal, hospital deliveries decreased, most markedly among disadvantaged groups, including women in castes perceived as lower in status [9].

Furthermore, the socioeconomic consequences of the COVID-19 pandemic and the related restrictions that were imposed indicate that approximately 131 million more people were pushed into poverty in 2020, many of them women, children and adolescents from marginalized communities [10]; a finding which suggests that COVID-19 has reversed progress for the first time since 1999 [11]. Nevertheless, the prospect of recovery from the COVID-19 pandemic is emerging with the count of zero-dose children almost at pre-pandemic levels [12].

Certain populations are being more affected than others by the socioeconomic consequences of COVID-19. These include: women, children, and adolescents; those who are marginalized and excluded; who depend on the informal sector for income; who live in areas prone to fragility; who have insufficient access to social and health services; who lack social protection; who are denied access to health services due to discrimination; who have low levels of political influence; who have low incomes and limited opportunities to cope and adapt; and who have limited or no access to technologies [13].

Conflict, climate change, COVID-19 and the cost-of-living crisis, known as the four Cs, present many challenges to the health and welfare of women, children and adolescents [14]. In 2022, more than 100 million people were forcibly displaced from their homes due to armed conflict and violence with women and children bearing the greater share of this burden [15]. In addition to the direct maternal and newborn fatalities attributed to the conflicts, there are greater indirect consequences such as the collapse of the health systems, the diminished access to health services and the disruption to food supplies [15,16]. Exacerbating these challenges are the effects of climate change which causes floods, droughts and crop failure that endanger the livelihoods of the poorest and most vulnerable populations [17]. This global polycrisis has drastically increased the cost of living worldwide; global inflation rose from 4.7% in 2021 to 8.8% in 2022. This has jeopardized lower income households’ access to maternal and newborn health services [14]. The impact and extent of this multifaceted crisis varies among countries, presenting distinct yet interconnected and complex challenges that exacerbate pre-existing inequalities, particularly in LMICs with vulnerable health systems [14].

In light of these emerging findings, there is an urgent need to stimulate, coordinate and deliver financing strategies that are equity-enhancing and that target the most vulnerable communities. Such efforts should be supported by evidence-based strategies and interventions to improve equity in WCAH and well-being, especially among vulnerable populations living in specific situations, such as humanitarian and fragile contexts. The objective of this scoping review is to explore the depth and breadth of existing literature on financing interventions and measures to improve equity in WCAH in LMICs and to map out and summarize the evidence to support decision-making and advocacy across different stakeholders, including governments, civil society organizations (CSOs) and donors

### Review questions

- What are the financing interventions and measures employed by different stakeholders, including governments, CSOs and donors, to improve equity in WCAH in LMICs?
- What is the available evidence on the effectiveness and implementation of financing interventions and measures to enhance equity for WCAH in LMICs?

## Methods

### Definitions

A scoping review is typically used to present “a broad overview of the evidence pertaining to a topic, irrespective of study quality, to examine areas that are emerging, to clarify key concepts and to identify gaps”. The updated JBI guidance for conducting scoping reviews was used [18] alongside the PRISMA Extension for Scoping Reviews (PRISMA-ScR) [19].

Financing for equity reflects on the need to “adapt, extend and scale up innovative and equity enhancing financing strategies that consider the differentiated reaches and impacts on vulnerable groups and populations, including women, children and adolescents in humanitarian and fragile settings” [20]).

Equity in health can be defined as “the absence of disparities in health (and in its key social determinants) that are systematically associated with social advantage/disadvantage” [21]. This report used the guidance framework PROGRESS-Plus, which identifies components affecting health and healthcare equity including place of residence, race or ethnicity, occupation, gender, religion, education, social capital, socioeconomic status (SES), plus age, disability and sexual orientation.

### Protocol and registration

The protocol was not registered as PROSPERO does not accept scoping review protocols. The protocol is available upon request from the corresponding author.

### Eligibility criteria

**Population of interest:** The population of interest consisted of women, newborns or neonates, children and adolescents. The explicit use of terms to define each population were used as they had been categorized into different age groups across the different articles.

**Intervention of interest:** The report looked at financing interventions and measures employed by different stakeholders including government, international donors and CSOs that aim to improve equity in WCAH. It considered demand and supply-side financial interventions including user fee exemption policies, national health insurance plans, subsidization policies, health equity funds, pro-equity policies, performance-based financing (PBF), financial protection schemes for vulnerable women, children and adolescents and incentive programmes such as CCTs, unconditional cash transfers (UCTs) and voucher schemes. The search excluded financial interventions targeting the general population without addressing any equity component such as SES, place of residence, disability etc. It also excluded studies that assessed financial interventions as part of a multi-component intervention and did not separate the results.

**Outcome of interest:** Only studies that assessed health outcomes were included, for instance those looking at morbidity and mortality and health systems outcomes such as access to healthcare, healthcare utilization and quality of care.

**Setting of interest:** The search focused on LMICs including humanitarian and fragile settings (HFS) as per the World Bank Country and Lending Groups’ classification by income issued in July 2022 [22] (S1 Appendix).

**Study design:** The study included primary studies, narrative reviews, systematic reviews and technical reports. The eligibility criteria was restricted to articles and reports published since the year 2000.

### Literature search

The following electronic databases were searched: Medline, PubMed, Emboss, the WHO’s Global Index Medicus. The websites of key actors in this space for technical reports were included, including Gavi, The Vaccine Alliance, the World Bank, the Global Financing Facility and UNHCR, The UN Refugee Agency. The search was conducted for studies published between January 2000 and June 2023. S2 Appendix provides the search strategies of the databases searched.

Both index terms and free text words for the three following concepts were used: health financing, women, children and adolescents, equity and setting (i.e. LMIC). The search strategy was co-developed and run by an information specialist who validated the information sources—for example, electronic databases and websites—the search and medical subject headings (Mesh) terms, the search techniques—for example, boolean operators and search filters—and the documentation of search strategies and results. The search was not limited to specific languages. Additionally, purposive outreach was conducted for a few key actors, such as WHO, Gavi, the Global Fund to Fight AIDS, Tuberculosis and Malaria and the World Bank, to identify additional documents.

### Selection process

**Title and abstract screening:** Teams of two reviewers used the above eligibility criteria to screen titles and abstracts of identified citations in duplicate and independently for potential eligibility. The full text for citations judged as potentially eligible by at least one of the two reviewers were retrieved.

**Full-text screening:** Teams of two reviewers used the above eligibility criteria to screen the full texts in duplicate and independently for eligibility. The teams of two reviewers resolved any disagreement by discussion or with the help of a third reviewer. Standardized and pilot-tested screening forms were used.

To ensure the validity of the selection process, calibration exercises were conducted. This involved a random subset of 100 articles for reviewers to independently screen against the eligibility criteria. Reviewers then met to discuss the point of disagreement and revise the eligibility criteria and instructions to ensure clarity, minimize disagreements and avoid confusion.

### Data extraction

One reviewer extracted data using standardized and pilot-tested forms and a senior reviewer validated these. The data extraction form was pilot tested to ensure the clarity and validity of the data extraction process.

The following information was extracted from each paper:

- Last name of first author or name of institutions for reports
- Year of publication
- Study characteristics:
- Type of publication
- Type of study design or report
- Language of publication
- Authors’ information:
- Country of affiliation of the contact author
- Country of affiliation of first author
- Source (Journal name or institution name)
- Setting:
- Country (ies) subject of the paper
- Income level classification according to the World Bank list of economies issued in July 2022 (S1 Appendix)
- HFS classification (S3 Appendix)
- Financing intervention or measure
- Type of intervention or measure
- Target population (women, newborns, children or adolescents)
- Equity component (PROGRESS Plus factor)
- Outcomes assessed and key findings including effects/implementation of interventions and SRMNCAH/well-being/equity outcomes
- Statements on funding and conflict of interest of authors

### Risk of bias assessment

No risk of bias assessment was conducted, consistent with the JBI guidance manual.

### Data analysis

A descriptive analysis of the general characteristics of the included papers, including study designs and settings, was conducted. A thematic analysis of the included studies was also done. These were categorized according to the intervention and outcomes assessed. Findings were further stratified according to variables such as country income group (LMIC level), HFS classification, population of interest and equity factor such as SES, migration etc.

## Results

**Fig 1:**
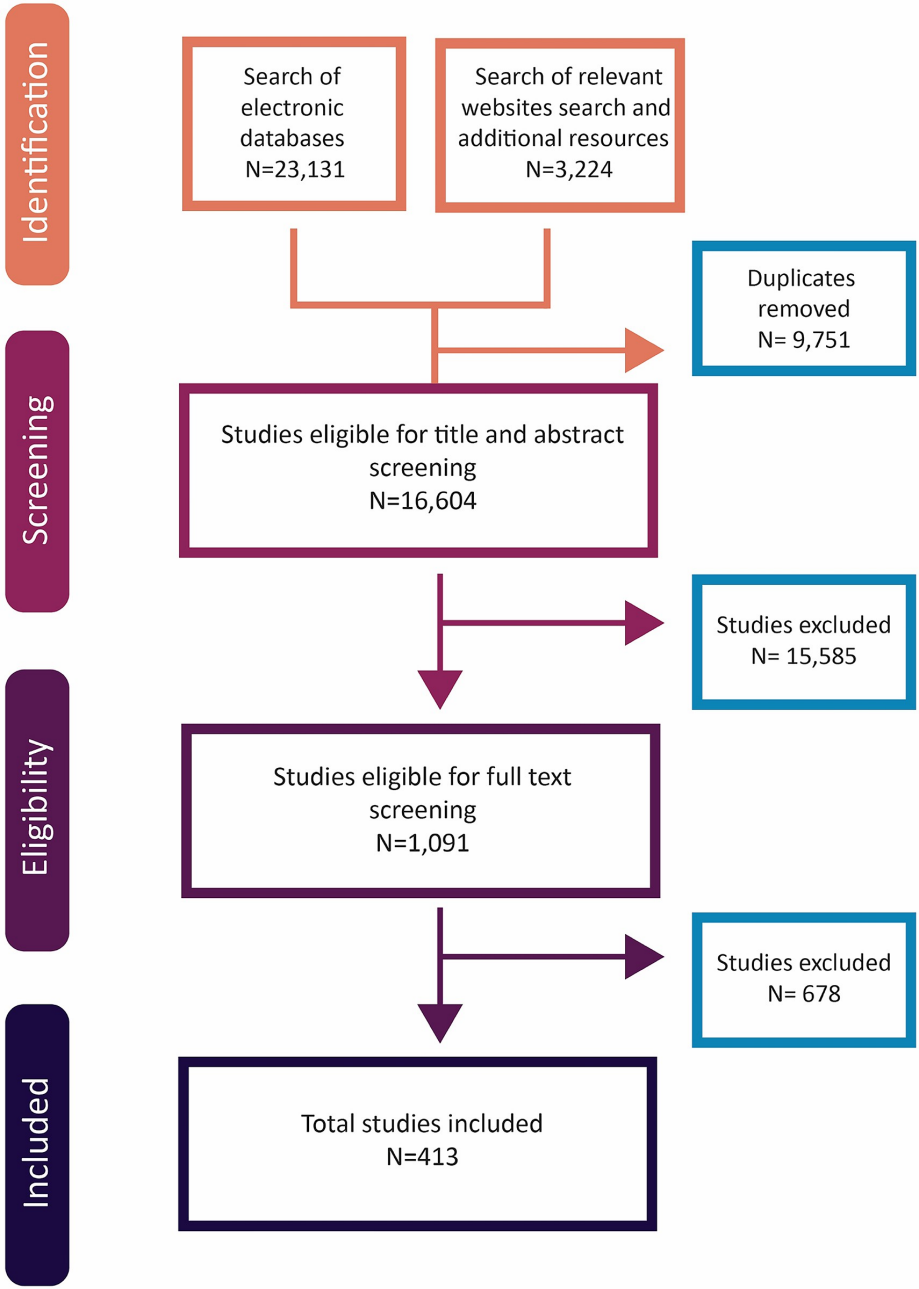
PRISMA flowchart.

Fig 1 summarizes the selection process. Out of 26 355 citations identified from electronic databases, relevant website searches and stakeholders consultations, 413 studies were included in the final scoping review [23–435]. 678 full texts were excluded for the following reasons: not an intervention of interest (n = 260); did not address equity (n = 152); not a population of interest (n = 134); not a design of interest (n = 61); not an outcome of interest (n = 18); not a setting of interest (n = 16); duplicate (n = 14); full text not retrievable (n = 13); no separate data for the effects of the financing policy (n = 9); or not a time frame of interest (n = 1).

### Characteristics of included studies

**Fig 2:**
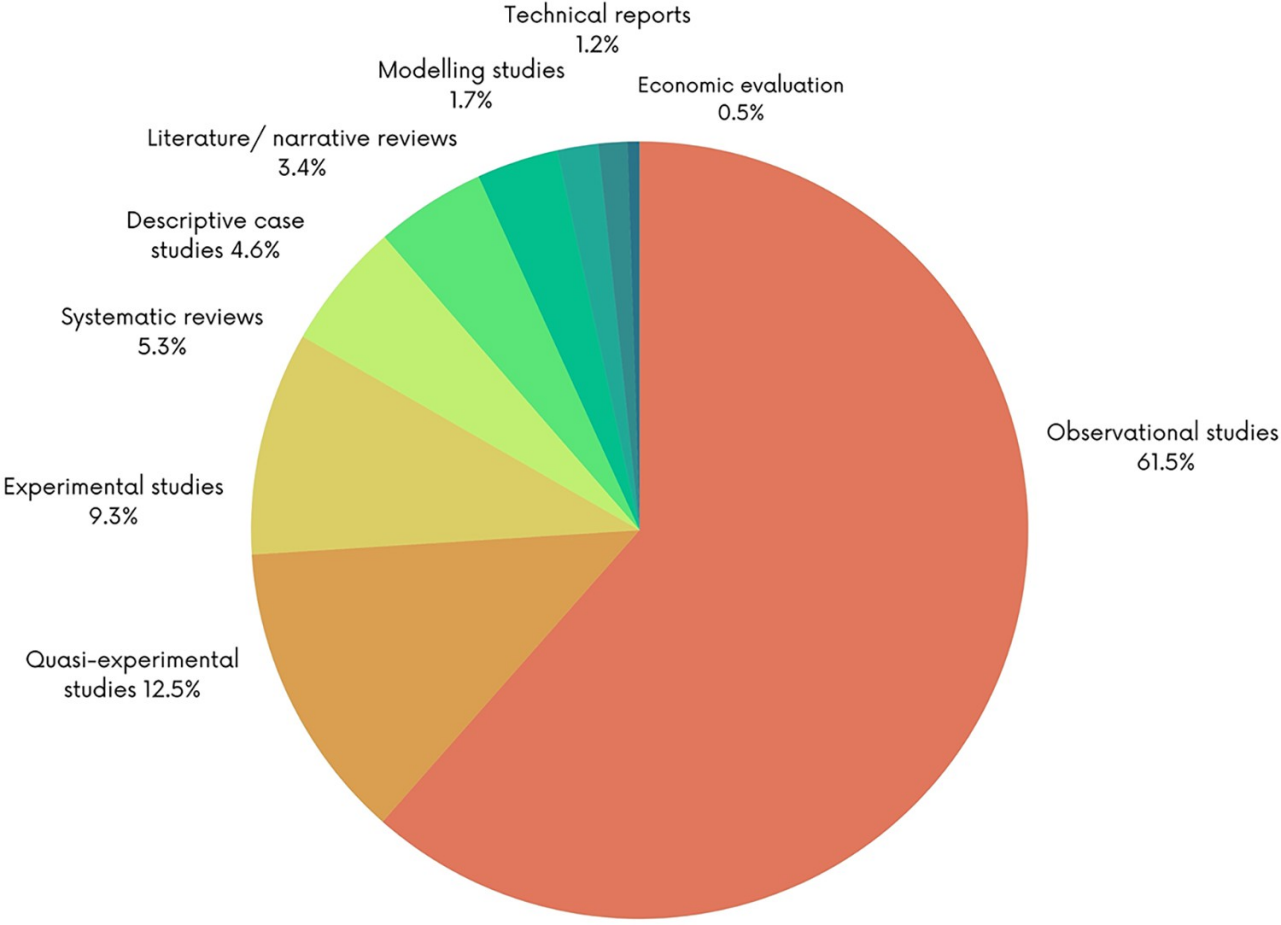
Type of study design.

**Fig 3:**
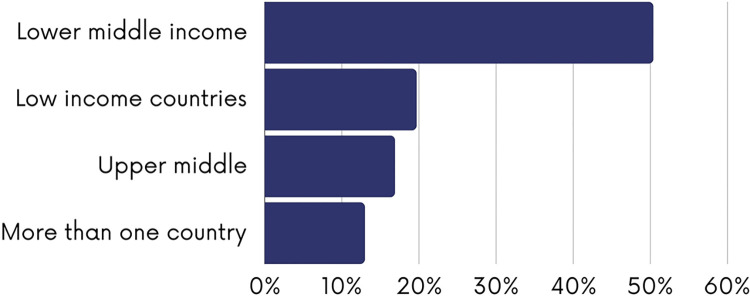
Classification of the country of the policy assessed.

**Fig 4:**
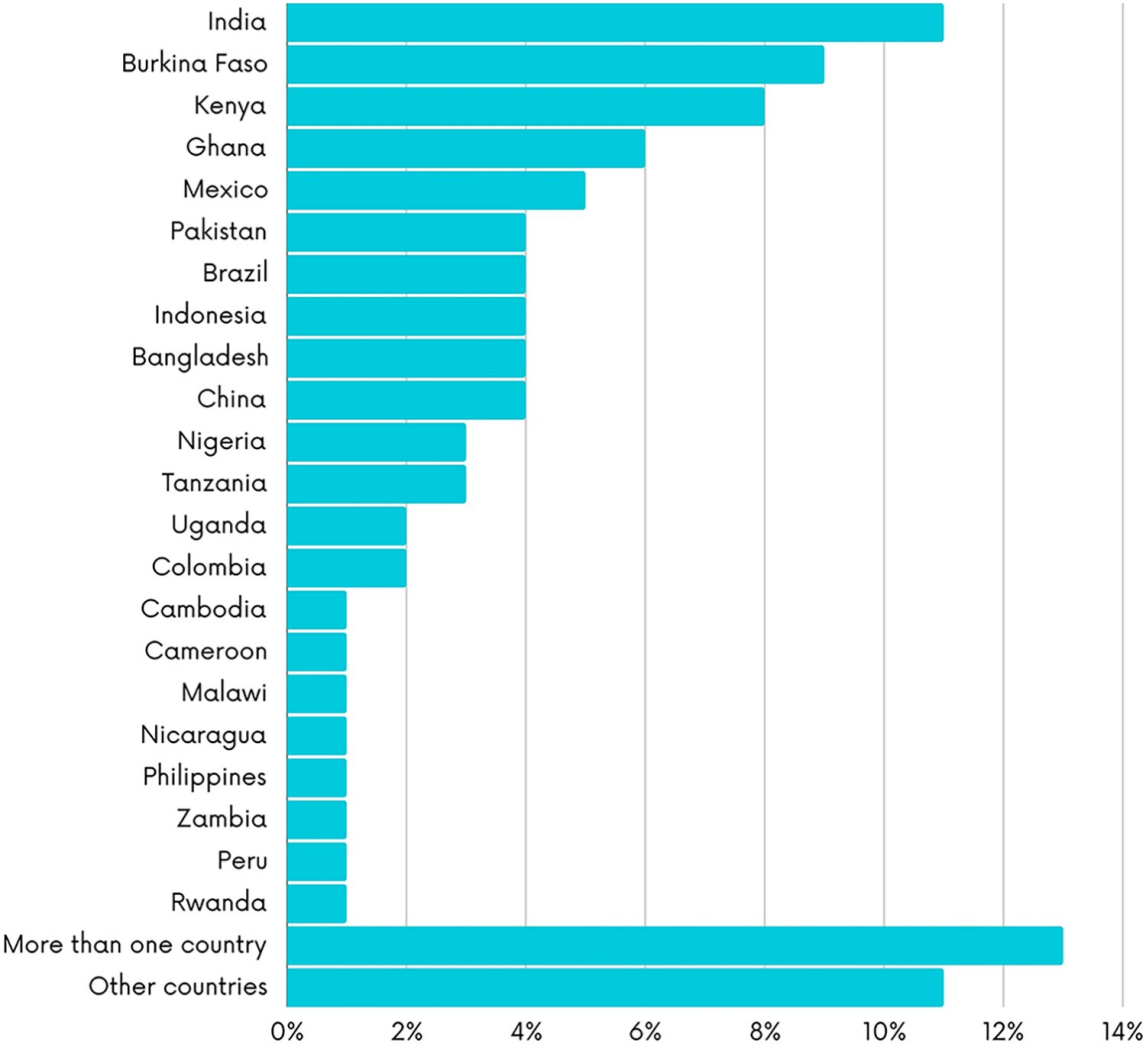
Country subject of the papers.

**Fig 5:**
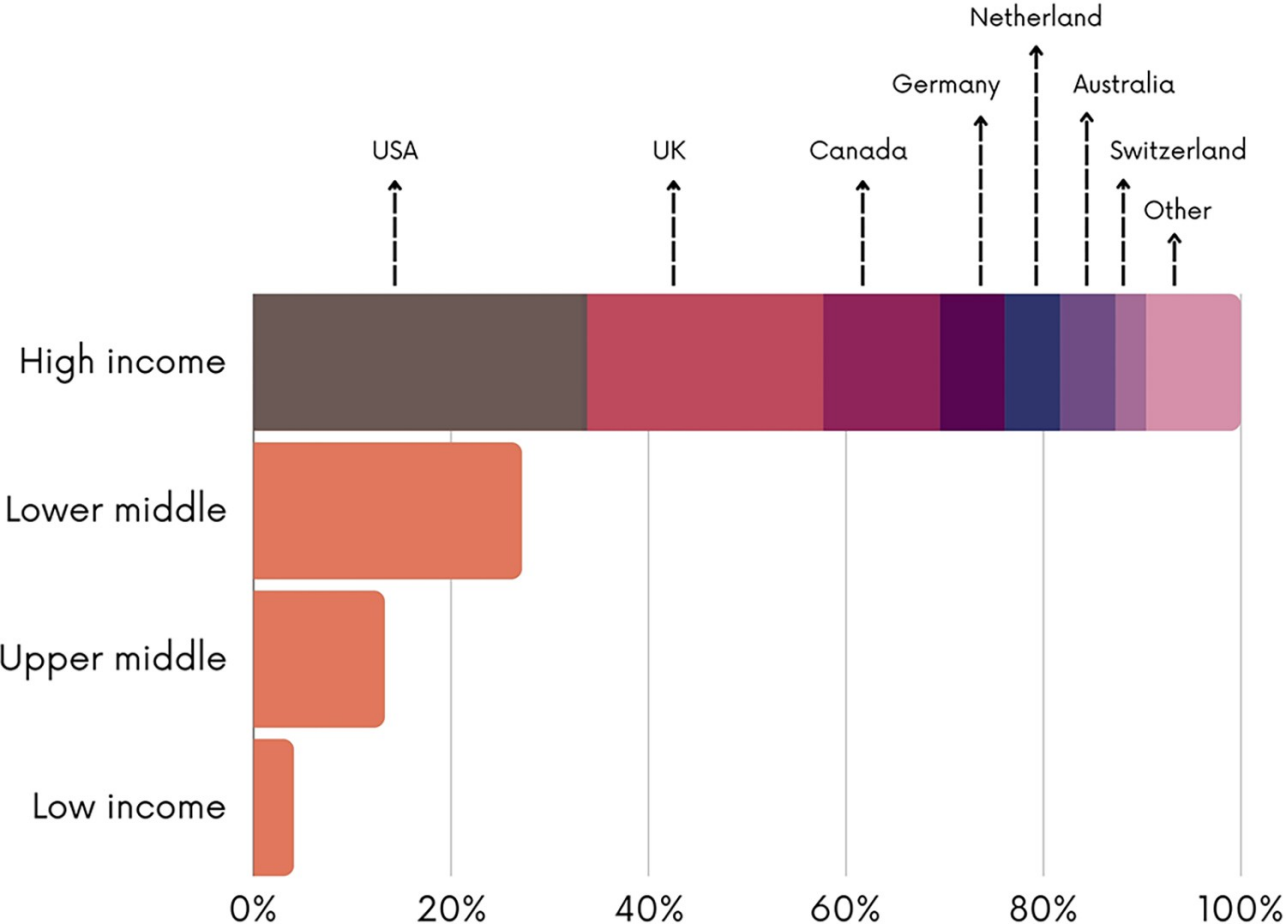
Classification of the country of the institution to which the first author is affiliated.

The figures below present the characteristics of the included studies. As shown in Fig 2, most of the studies employed observational study designs (n = 254; 61.5%), followed by quasi-experimental (n = 52; 12.5%) and experimental designs (n = 38; 9.3%). 22 systematic reviews on the topic were identified (5.3%). Most the studies were conducted in lower-middle income countries (n = 209; 50.6%) and low-income countries (n = 82; 19.7%) with fewer studies conducted in upper-middle income countries (n = 70; 13%) (Fig 3). Most of the studies were conducted in India (n = 40; 10.6%) or Burkina Faso (n = 35; 8.7%) (Fig 4). The first authors of included studies were mostly affiliated with institutions from high-income countries (n = 228; 55.2%) mainly the United States (n = 76; 33.2%), the United Kingdom (n = 56; 24.5%) and Canada (n = 27; 11.8%) while only 4.1% of studies had first authors affiliated with institutions from low-income countries (Fig 5).

**Fig 6:**
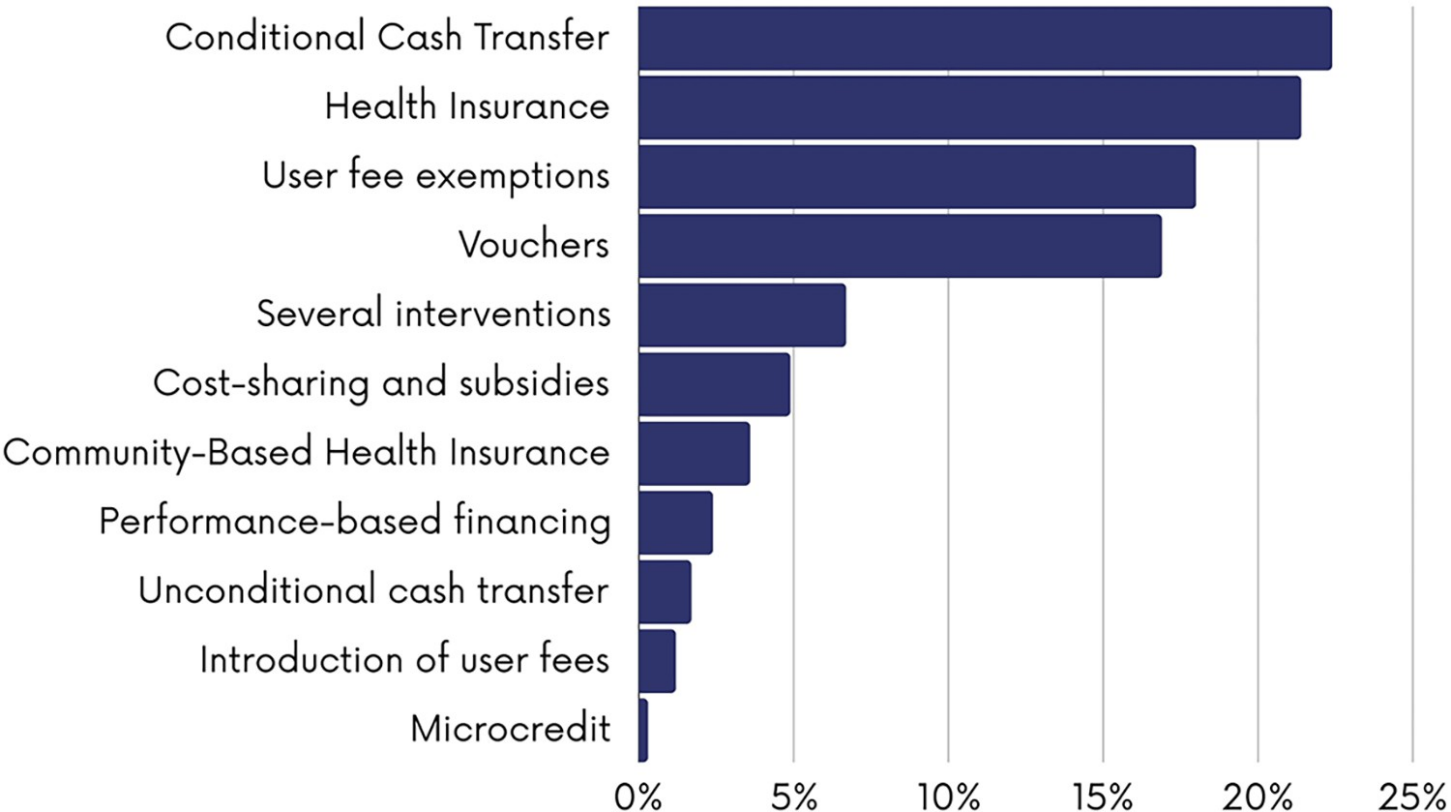
Financial mechanisms assessed.

**Fig 7:**
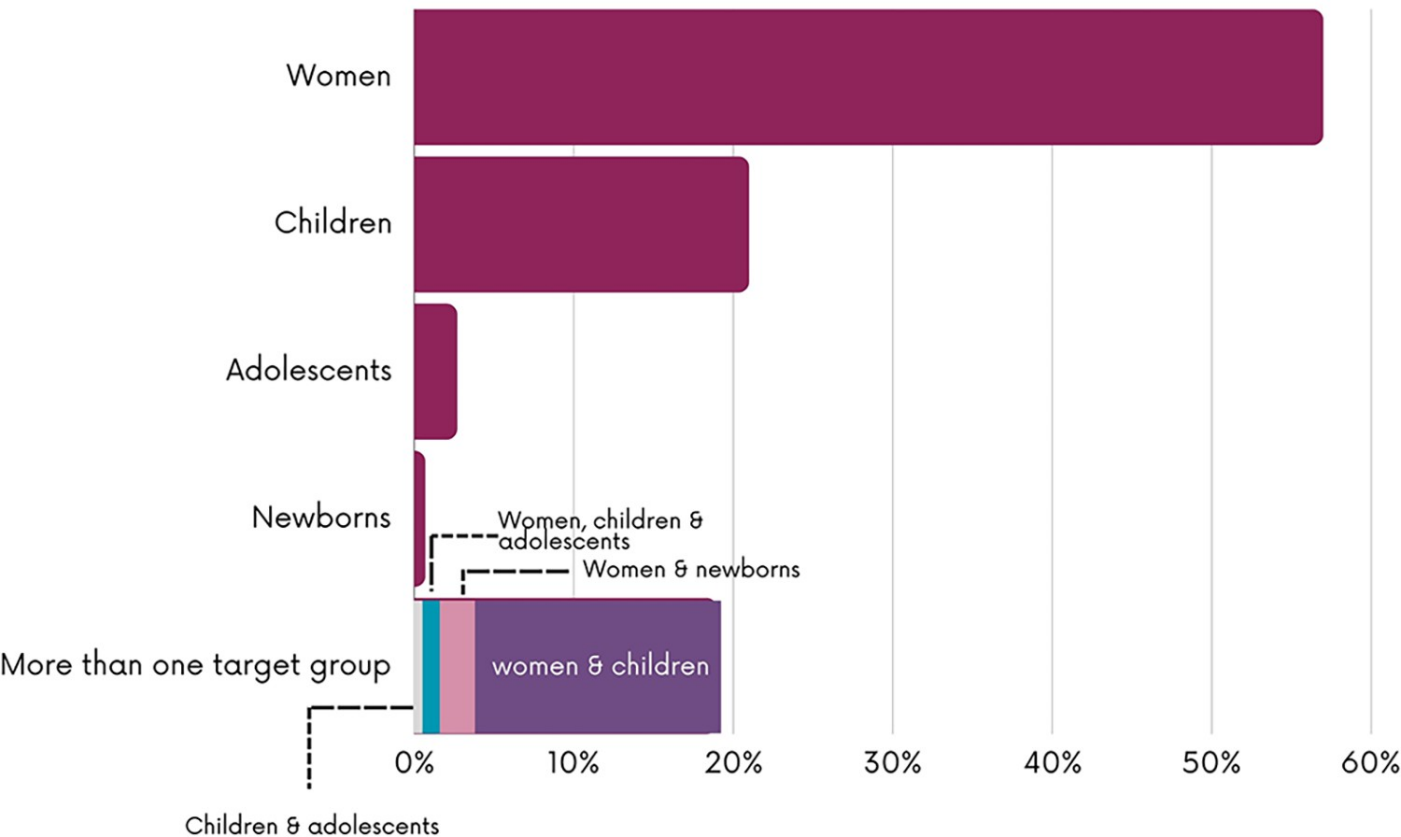
Target population of the financial mechanism.

**Fig 8:**
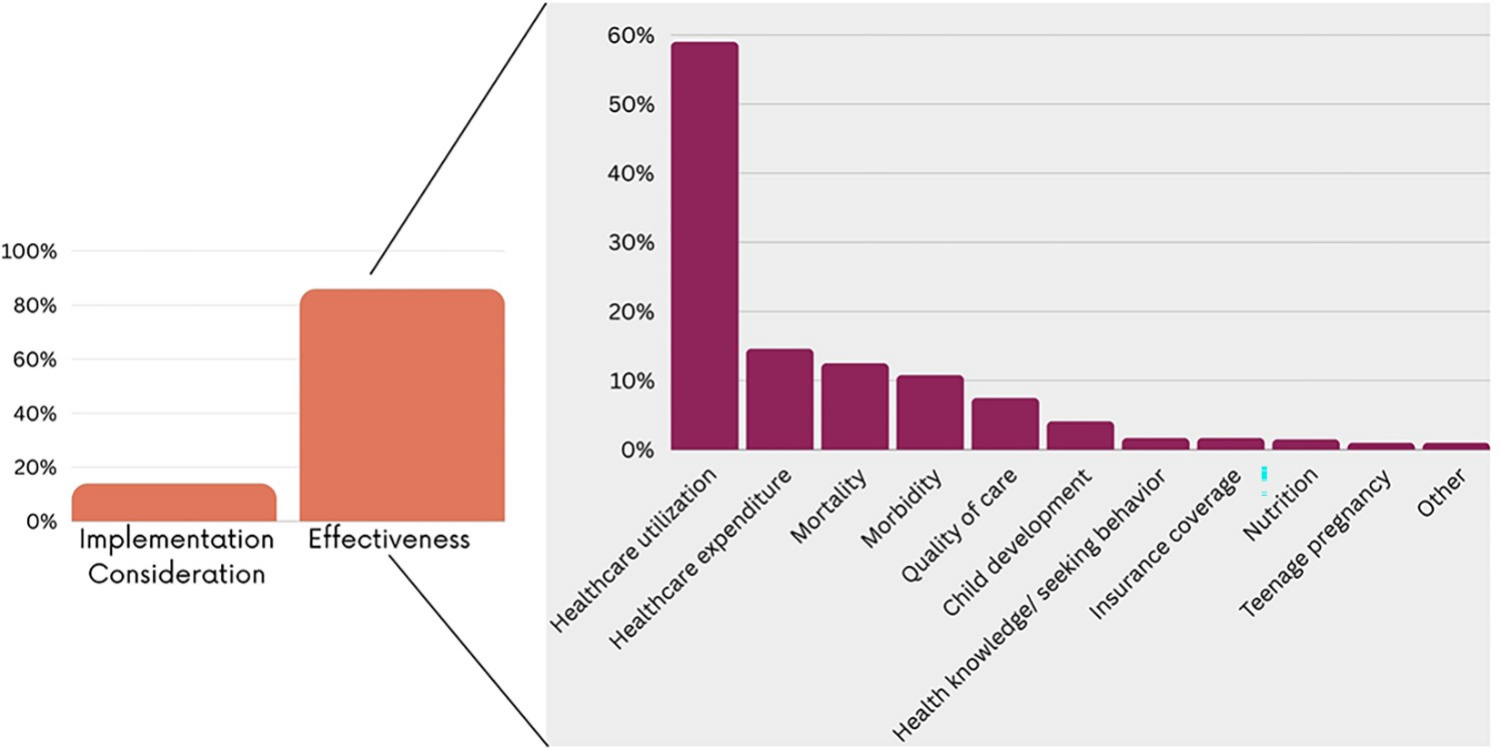
Type of assessment and outcomes measured.

As shown in Fig 6, CCTs (n = 92; 22.3%), health insurance (n = 89; 21.4%), user fee exemptions (n = 75; 18.1%) and vouchers (n = 70; 16.9%) were the most reported financial interventions and measures. Some studies assessed several financial interventions whether implemented separately or as multi-component financial interventions (n = 28; 6.7%). Financial interventions or measures mainly targeted women (n = 236; 57.1%), children (n = 87; 21%) or both women and children (n = 62; 15%) with fewer targeting adolescents (n = 10; 2.7%) and newborns (n = 3; 0.7%) (Fig 7). As shown in Fig 8, the majority of the studies were evaluation studies (n = 354; n = 85.7%) with 59 studies addressing implementation considerations (14.2%). The outcome mostly assessed was the healthcare utilization (n = 245; 59.3%) followed by health expenditures (n = 61; 14.6%), mortality rates (n = 52; 12.6%) and morbidity (n = 44; 10.8%).

### Equity components

**Fig 9:**
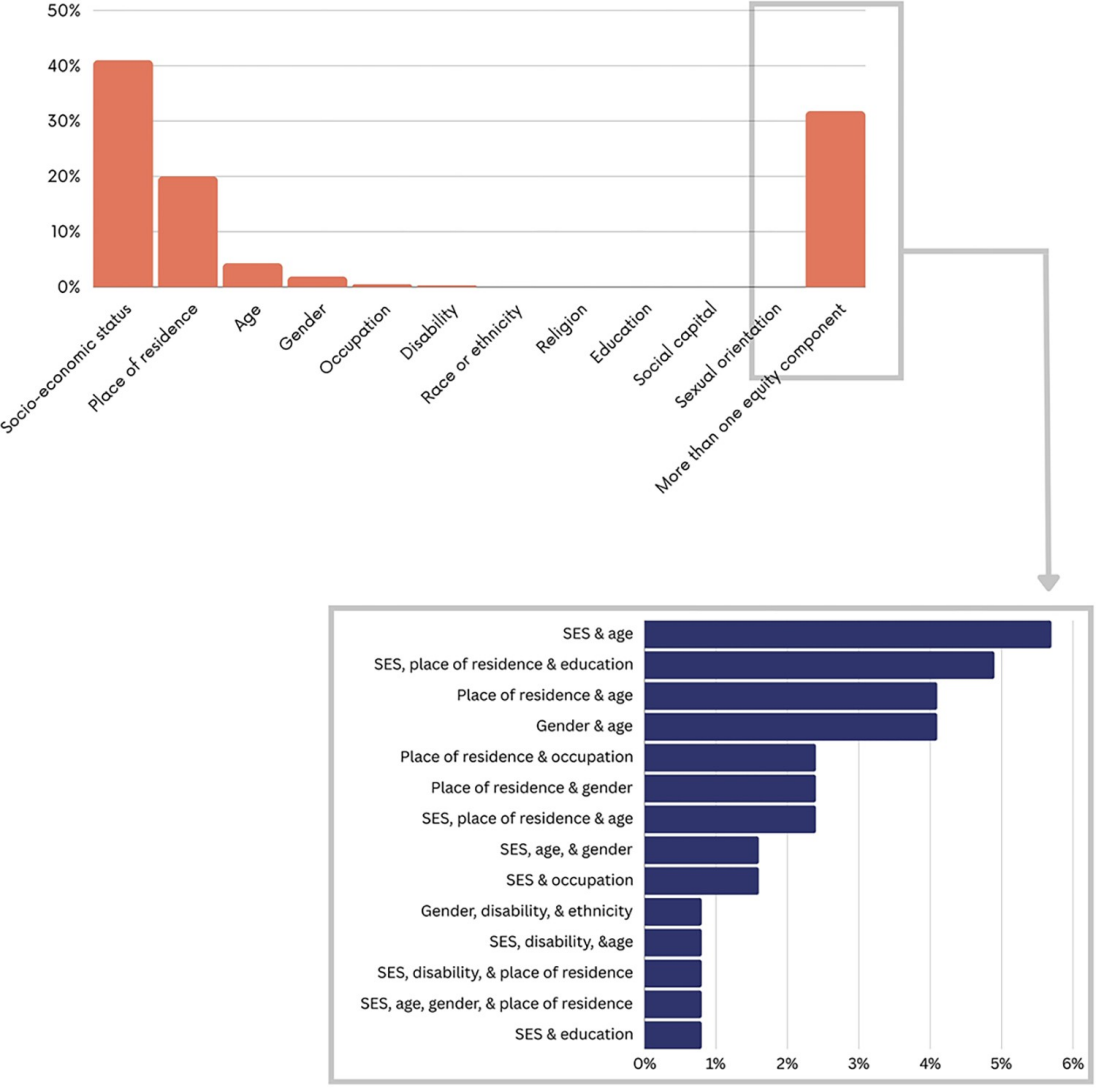
Equity components addressed.

Fig 9 presents the equity components addressed in the included studies. Most of the studies assessed the impact of financial interventions on addressing inequities related to SES (n = 171; 41.4%) followed by place of residence (n = 82; 19.8%) and age (n = 17; 4.2%). Many studies addressed several equity components (n = 132, 31.9%) including SES and place of residence (n = 70; 53%), SES and ethnicity (n = 8; 6.1%), SES and age (n = 7; 5.3%), SES, place of residence and education (n = 7; 5.3%), SES and occupation (n = 2; 1.6%), gender, disability and ethnicity (n = 1; 0.8%). None of the included studies addressed sexual orientation, religion or social capital.

### Funding and conflicts of interest characteristics of the included studies

**Fig 10:**
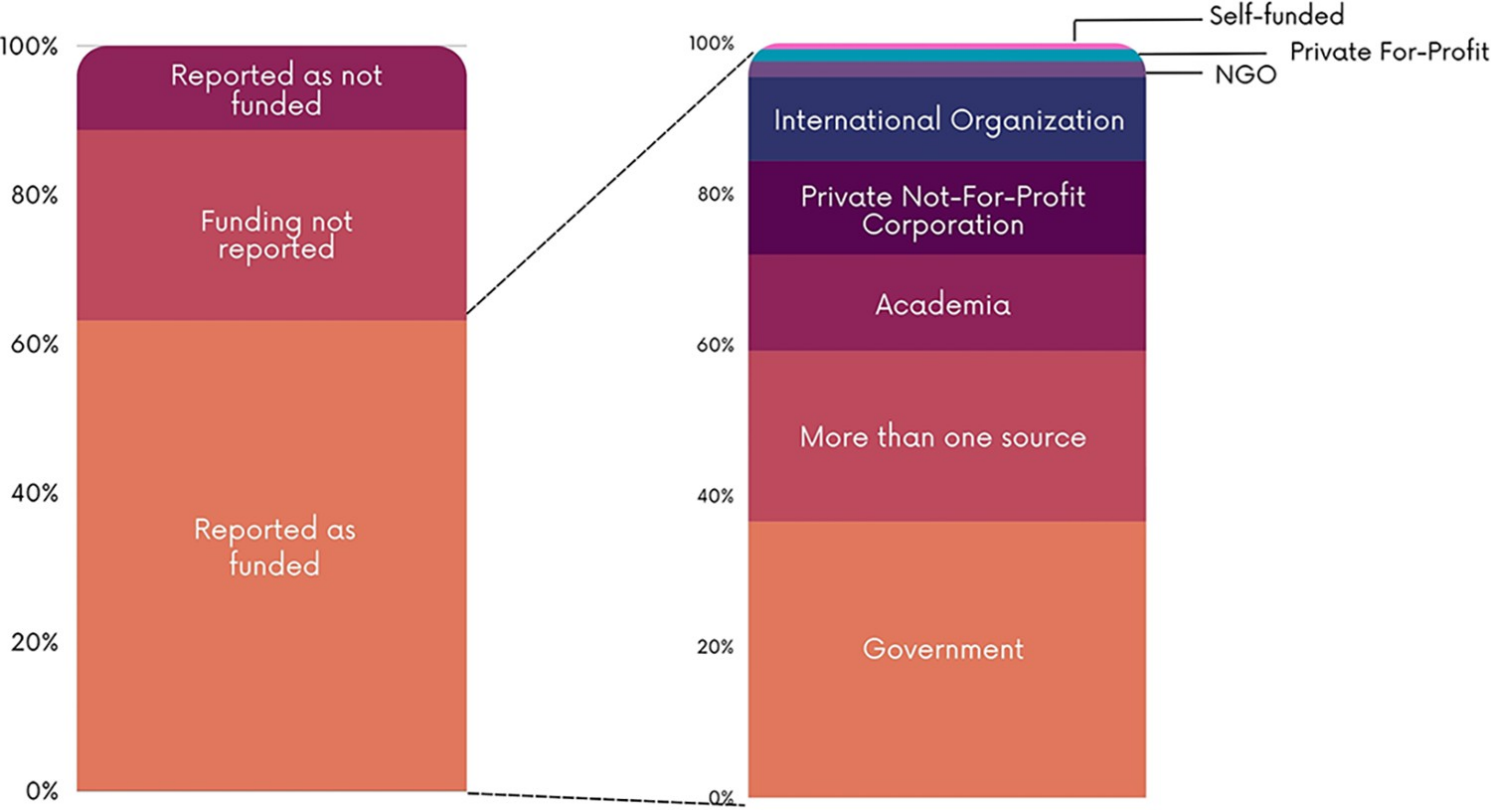
Reporting of funding.

**Fig 11:**
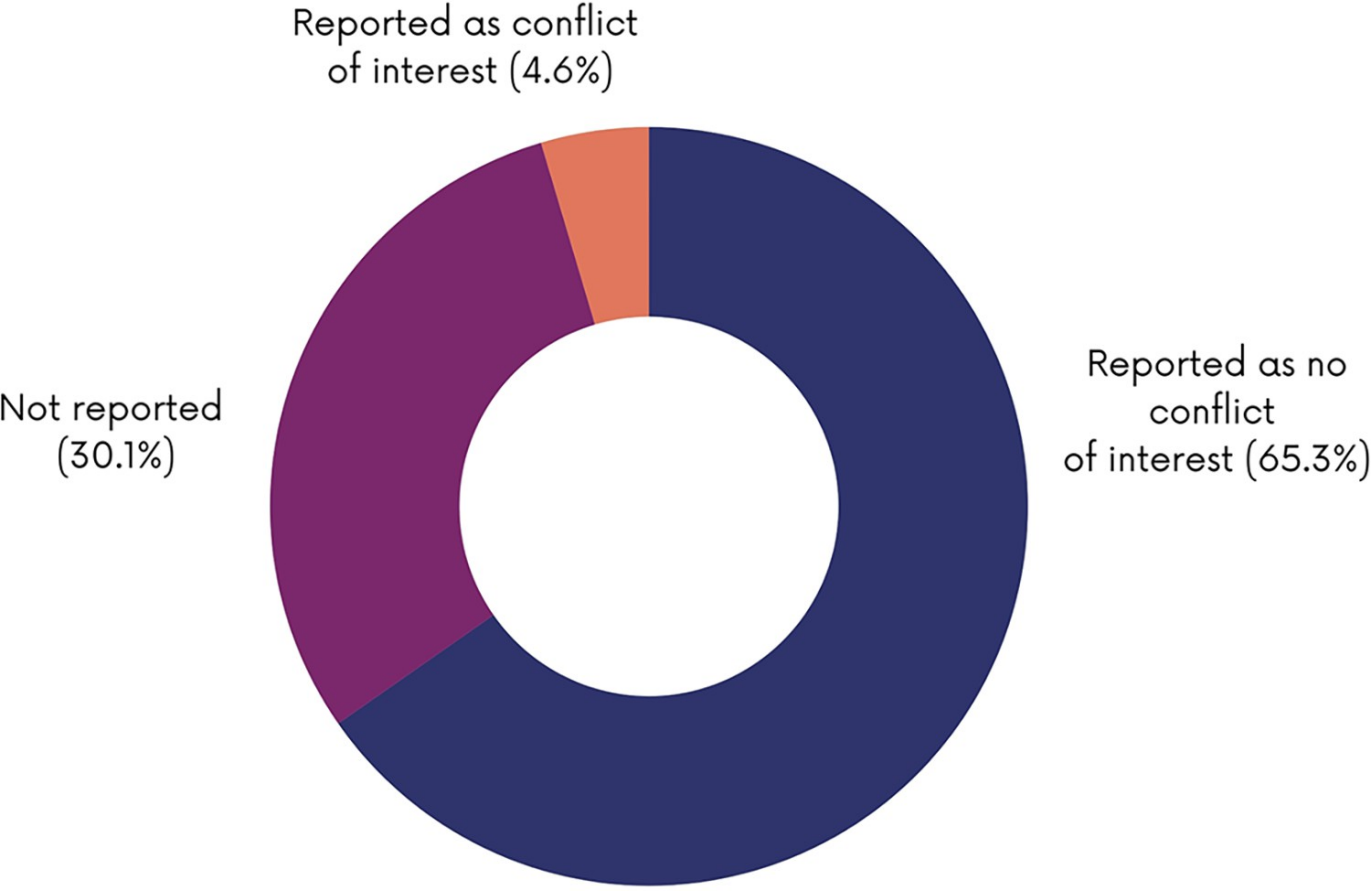
Reporting of conflict of interest.

As shown in Fig 10, most of the studies reported study funding (n = 261; 63.1%). Of the studies reported as funded, the funding sources were mainly governments (n = 96; 36.6%), academia (n = 31; 12.8%), private not-for-profit (n = 33; 12.4%) and international organizations (n = 30; 11.1%). Some studies reported being funded by multiple sources (n = 59; 22.6%). Almost a third of the papers (30.1%) did not report on conflict of interest of study authors (Fig 11).

### Findings

The findings below are presented by financial interventions and corresponding outcomes. S1 to S11 Tables present the characteristics of the individual studies.

### CCTs (n = 92)

CCTs have been introduced in many LMICs as a provision of monetary transfers to households on the condition that they comply with a set of requirements [46]. 92 studies evaluated the effectiveness of CCT programs on healthcare utilization, morbidity, mortality, child development, healthcare expenditure, quality of care and other outcomes including health knowledge, responsive caregiving, teenage pregnancy and nutrition as well as implementation considerations [23–109,407–411]. The majority of the studies assessed CCT programs in Latin America (n = 41), mainly Brazil (n = 17), and India (n = 24) assessing the Janani Suraksha Yojana (JSY) program.

Healthcare utilization (n = 48): 48 studies assessed the effect of CCT programs on healthcare utilization [23–27,29,30,32,33,35–37,39–43,45–50,59,62,63,71,78,81–87,89,90,94–97,100,101,105,107–109,408]. 40 studies found that CCT interventions can increase the utilization of maternal and child health services for the poor and the disadvantaged, including antenatal care visits [24–26,29,30,35,39,41–43,45,47,50,63,96,105,108], post-partum care [63,105], institutional delivery [23–26,29,33,36,37,47,78,82,84–87,89,94], caesarean sections [37,95], skilled birth attendance [26,45,94,96], contraceptive use among young women [90], child health check-ups [40–42,46,49,59,81] and immunization coverage [26,27,32,71,100,101]. One study reported that the CCT program encouraged adolescents to attend the clinic for assessments or HIV testing [109]. Although CCT was shown to increase access to institutional delivery, three studies revealed a disproportionate lower concentration of institutional deliveries in low-income populations in comparison to high-income populations [33,36,48]. One study revealed that CCT decreased rates of caesarean section deliveries, which is explained by a shift from the private sector [94]. Two studies suggested that CCT programs addressing both the supply and demand side of health services may increase the coverage of maternal and child health services [62,408]. Five studies found negative results on the impact of CCTs on immunization coverage [41,46,63,83,87]. One study reports the poor effect of CCTs on immunization coverage to be because of weak monitoring of conditionality [83]. One systematic review found mixed results on immunization coverage that might be due to the initial high rates of immunization [46] while another systematic review found insufficient evidence on the impact of CCTs on child health service utilization in sub-Saharan Africa [107].

Mortality (n = 20): 20 studies reported on the effect of CCT programmes on maternal and child mortality [23,24,26–30,32–34,73,85,87,88,91,94,99,103,409]. 13 studies and one systematic review revealed a positive impact of CCTs in reducing infant, child and maternal mortality rates for the poor and the disadvantaged [27–30,32,34,73,85,87,88,91,99,103,409,410]. Five studies reported a reduction in child mortality whereby the intervention led to a decrease in overall mortality from poverty-related causes, which included malnutrition and diarrhoea [24,27,34,73,91]. One study suggested an improvement in child mortality when CCTs are combined with improvements to adequate access to water, sanitation and solid waste collection [34]. Another study highlighted that although CCT programmes reduce maternal mortality, inequalities persisted whereby fewer death were observed in the richest divisions compared to the poorest division [33]. One study showed a positive impact on determinants of maternal mortality but suggested a small effect on maternal mortality due to implementation issues [25]. Three studies did not find any impact of CCTs on maternal or child mortality [23,26,94].

Morbidity (n = 16): 16 studies reported on the effects of CCT programmes on morbidity [32,46,55,56,64,66,79–82,85,91,98,102,107,411]. 10 studies found that CCT interventions can improve child health for vulnerable groups by reducing malnutrition and wasting [79,91,411], hospital admissions [27,91], illness and anemia rates [46,64,79,85], diarrhea and acute respiratory infections [32], psychosocial health [81] and incidences of dental caries [80]. One systematic review found insufficient evidence on the impact of CCTs on child health status in sub-Saharan Africa [107]. One study suggested that CCTs reduced HIV risks among young women [56]. Two studies reported a positive impact of CCTs on women’s health including a lower prevalence of being underweight or overweight [79] and lower current hypertension rates [55] while one study reported an increase in body mass index (BMI) and obesity risks among women [66]. Another study found no evidence that the CCTs led to a reduction in preventable delivery complications [82]. Two studies found no impact on mental health among youth [98,102], mainly among females, suggesting that conditions should not stereotype females [98].

Child development (n = 14): 14 studies addressed the effect of CCTs on child development [30,38,43,46,52,57,58,60,64,67,75,76,103,104]. 10 studies found that CCT interventions can improve child health by reducing the incidence of low birth weight [30,57,103,104], and improving height-for-age Z scores, length and stunting among children [38,43,46,58,64,75,76]. Four studies showed that CCTs did not significantly impact weight-for-age Z scores [43], height-for-age Z scores [67], BMI-for-age Z scores and stunting [52] and weight gain [60].

Other health outcomes (n = 9): Nine studies reported on the effect of CCTs on health knowledge and behavior [25,88], responsive care giving [43], teenage pregnancy [61,65], adult fertility [72] and nutrition [40,74,93]. One study showed an increase in demand for maternal and child health services within facilities among beneficiaries [88] while another study found no improvement in health knowledge among mothers [25]. Three studies reported on the effectiveness of CCT programmes on nutrition [40,74,93]. All studies suggested a positive impact of CCTs on nutrition determinants including an increase in dietary diversity and food consumption [40,93], especially in rural areas [40], and an increase in food per capita household consumption [74]. One study found that CCTs resulted in a positive change in key parenting practices, including children’s intake of protein-rich foods and care-seeking behavior [43] and a decrease in teen pregnancy and fertility [61,65] with no effect on adult fertility [72].

Health expenditures (n = 3): Three studies from India found that although CCTs can enhance financial access to maternal and child health services, it did not adequately protect beneficiaries from out-of-pocket expenditures [84,97,106].

Quality of care (n = 2): Two studies reported on the effect of CCTs on the quality of care and beneficiaries’ satisfaction [82,103]. CCT programmes significantly improved the quality of delivery care and satisfaction with care in one study [82]. One systematic review reported on two studies from Mexico suggesting that CCTs improved the quality of care but the measure of care was based on women’s recall [103].

Implementation considerations (n = 15): 15 studies reported on implementation considerations for CCT programmes [31,36,44,51,53,54,68–70,77,88,92,98,105,407]. Challenges to the implementation of CCT programmes included the complicated eligibility criteria, irregular cash transfers, insufficient entitlements, unclear payment procedures, the quality of care in public health facilities, insufficient healthcare personnel, infrastructure, medicine and equipment, increased workload and low literacy levels among beneficiaries [36,44,54,68,70,77,88,407]. The lack of awareness among beneficiaries about the CCT programme and the included services may have also hindered service uptake [36]. In India, the bribes imposed by providers incurred additional out-of-pocket expenditures for CCT beneficiaries [53]. The misuse and mismanagement of cash were also reported as a challenge [44,54,70]. In one study, beneficiaries proposed that CCT programmes may be unsustainable and expressed concern that young women may misuse or mismanage the transfers [54]. Another study reported that mothers’ in-laws forced their daughters-in-law to stay in their villages, not allowing them to go to the mother’s area at the time of delivery for the sake of securing the cash incentive, which is given only upon the condition of the women delivering in the local institution where she is registered [70]. To increase the effectiveness and acceptance of CCT programmes, studies suggested the need for adequate human resources, staff training systems, improving the access and quality of care, community engagement in the design and implementation of the programme, improvement in targeting, increasing awareness of the programme and removing any stereotype in conditions and extensive monitoring [31,36,51,69,77,92,98,105].

### Health insurance (n = 89)

89 studies tackling various health insurance schemes including national health insurance, social health insurance and employment-based health insurance were included. These studies evaluated the effectiveness of these various health insurance schemes on healthcare utilization, healthcare expenditure, mortality, morbidity, quality of care and other outcomes including insurance coverage and nutrition as well as implementation considerations [327–404,406,412–421].

Healthcare utilization (n = 46): 37 studies reported that various health insurance schemes increased skilled birth attendants, facility-based deliveries, caesarean section deliveries, antenatal care utilization, children health consultations and vaccination coverage, utilization of health services among women with disabilities and use of family planning services [328,334,336,337,339,341,345,346,349–352,354,358,359,365,367,368,370,374,376,377,379,383–387,390,391,394–397,399,401,404,406,414,420]. 11 of these studies indicated that these favorable effects were mostly prominent among poor households [334,337,367,370,377,394,396,397], those residing in rural areas [346], individuals with disability [345] and indigenous women [390]. This suggests that insurance schemes led to more equitable access to health services among the impoverished and vulnerable populations. While three studies reported that although there has been a notable increase in the utilization of maternal health services due to the insurance schemes, this increase was not as significant among the extremely poor [349,415] and those residing in rural areas [376]. Five studies showed that insurance schemes had no effect on the use of family planning services [329,363], access to HIV testing during antenatal care visits [332], utilization of outpatient services in children [343] and facility-based deliveries [403].

Health expenditures (n = 21): 10 studies reported that enrollment in an insurance scheme reduced households’ out-of-pocket expenditures for maternal care [340,352], facility-based deliveries [337], inpatient care [330,348], child care [334,365], neonates critical care [375] and breast cancer treatment [382,401]. Five studies indicated that insurance schemes can protect poor households and migrant women from the financial burden of out-of-pocket health expenses [369,380,392,420], and can provide adequate health coverage for children with mental disorders [347]. Four studies showed that these schemes had no significant effect on reducing out-of-pocket payments sustained by poor households for maternal and child care [336,345,362,421]. As for the remaining two studies, they reported that the enrollment in insurance schemes caused women extra financial hardship due to their lack of awareness about their rights within the programme and mistrust in the scheme that drove them to private health providers [389] and that women from lower socioeconomic disadvantage had higher out-of-pocket health expenses than those from higher socioeconomic disadvantage [402].

Mortality rate (n = 15): 12 studies reported that national health insurance schemes reduced maternal, neonatal and infant mortality rates [327,341,351,358,371,375,378,379,382,385,393,394]. Three studies reported that insurance schemes had no significant positive effect on neonatal mortality rates [403], didn’t reduce inequality in maternal mortalities within the regions [388] and that one year all-cause mortality rates were notably higher in women enrolled in rural insurance schemes than those enrolled in an urban insurance scheme [381].

Morbidity (n = 10): All 10 studies reported a positive effect of the different types of health insurance schemes on treatment adherence in children with cancer [333,394], overall health status of women and children [355,356,398,400], preventing malnutrition and stunting in children [344,366,379] and reducing the incidence of influenza and diarrhea in children [375].

Other outcomes (n = 9): Five studies highlighted inequities in health coverage under several insurance schemes with women who are poorer, less educated, unemployed or residing in remote areas being less likely to be insured [335,353,357,412,413]. One study showed that a health insurance scheme implemented in Peru had increased health coverage among marginalized women and contributed to more equity in healthcare access [364]. Another study reported that enrollment in a public health insurance scheme in China didn’t improve the nutritional status of rural poor children [372]. A study conducted in Colombia showed that women living in low socioeconomic residential areas and who were affiliated to a subsidized health insurance scheme (versus contributive) experienced a reduced probability of cervical cancer survival compared to women living in high socioeconomic areas [419]. One study found higher cancer survival rates for insured versus uninsured children in Kenya [417].

Implementation considerations (n = 8): Eight studies reported on different barriers that hindered either women’s enrollment in insurance schemes or their access to healthcare despite their enrollment. The barriers included: the need to move to another city to get the treatment; the authorizations of the paying entities; the medication costs [331], the distance to facilities [418]; having unreliable sources of income [342]; having invalid insurance cards [353]; having a low educational level [360]; encountering high premiums [416] and the extra costs of care [336]. One study addressed facilitators to the enrollment of children from lower socioeconomic status in insurance schemes. These included: eliminating the remaining small yearly renewal fee’ organizing outreach initiatives to offer registration assistance to female guardians of children; and establishing additional administrative offices for the programme in remote areas [361]. Another study addressed the facilitators of enrollment of poor women residing in rural areas. These were educating the public on the importance of enrolling in a health insurance programme and offering initial free subscriptions to individuals with the lowest economic wealth status [391].

### User fee exemption (n = 75)

User fee exemptions have been implemented in many LMICs to alleviate the substantial out-of-pocket payments and the resulting financial burden incurred by low-income households [113]. 69 studies evaluated the effectiveness of user fee exemption policies on healthcare utilization, healthcare expenditure, mortality, morbidity, quality of care and other outcomes including health-seeking behavior and teen pregnancy as well as implementation considerations [107–175,419–424].

Healthcare utilization (n = 43): 27 studies reported positive effects of these programmes on the use of facility-based delivery services [113,121,126,139,141,147,150,156,160,163,168,173,426], malaria prevention and treatment services for pregnant women and children [119,145,155,161], maternal and child consultations [122,132,133,136,142,158,164,177] and family planning and contraception services [167,170], leading to more equitable access among the poor and vulnerable populations. 16 studies indicated that user fee exemption programmes had minimal to no effect on increasing the utilization of maternal and sexual and reproductive health services among poor women or those living in rural areas [110–112,115,120,123,124,127–129,134,137,138,140,157,178].

Health expenditures (n = 17): Nine studies reported a positive effect of user fee exemption programmes on reducing out-of-pocket health expenditures and medical expenses among poor households [130,131,143,152,157,171,172,177,426]. While eight studies showed limited to no effect of these programmes on eliminating out-of-pocket health expenditures among impoverished households, this could be attributed to additional expenses not covered by the scheme such as transportation, medical supplies, medications and unofficial provider fees [116,150,154,159,165,175,176,425].

Mortality (n = 7): Four studies reported that despite the implementation of user fee exemption programmes there was no noticeable reduction in maternal, neonatal or under-five child mortality rates [119,126,139,168]. Three other studies reported that these programmes reduced maternal, neonatal and under-five child mortality rates due to the uptake of institutional deliveries and child health services [113,121,145].

Morbidity (n = 4): Three studies reported a positive effect of user fee exemption programmes on reducing wasting and stunting in children [162], on prompt treatment of sick newborns [166] and on improved screening for sexually transmitted infections (stir) in young women [175]. One study indicated that these programmes did not reduce illness in children [115].

Quality of care (n = 4): Three studies reported that user fee exemption programs had a positive effect on the quality of delivery and maternal care services [135,172] and on the quality of medical prescriptions with physicians reducing their use of antibiotics in for children by 62% [177]. One study indicated that the quality of maternal care provided under these programmes was perceived as poor by managers and health providers due to the increase in workload, delayed reimbursement of funds and stock-out of essential drugs and medical supplies [117].

Other outcomes (n = 2): Two studies reported that user fee exemption programs led to a decrease in teen pregnancies [169] and had a positive effect on health-seeking behaviors in children [149].

Implementation considerations (n = 15): Nine studies reported on several barriers to the implementation and uptake of user fee exemption policies. These included: a lack of infrastructure and human resources; sociocultural factors; limited awareness of the policy; out-of-pocket payments on drugs; transportation and lab tests; reduced quality of the services; and increased administrative workload [114,118,125,148,154,174,422,424,427]. Six studies reported on facilitators for the implementation of these policies. They included: residing near the facility; knowledge and awareness of the policy; residing in urban areas; having a good drug supply system in place; and having higher levels of education [144,146,151,153,156,423].

### Vouchers (n = 70)

This financial intervention involves distributing vouchers of a predetermined value to patients, which they can redeem to receive services from providers who are then repaid by the insurance or government payor that issued the voucher. 70 studies that evaluated the effectiveness of voucher schemes on healthcare utilization, quality of care, health expenditures, mortality, morbidity, health knowledge and child development as well as implementation considerations were included in the scoping review [176–243,425,426].

Healthcare utilization (n = 56): All 56 studies reported on the positive effect of voucher schemes on the use of sexual and reproductive health services [179,180,183,189,196,197,204,205,210,213,218,225,231], contraceptive and family planning services [181,182,187,192,200,201,208,211,212,222,228,241,244,246,428], facility-based deliveries [186,190,193,199,209,214,223,224,227,229,232,233,238,242,245], skilled birth attended deliveries [191,230], antenatal care services [185,198,215,219,226,239], child immunization services [194,429] and the use of insecticide-treated nets for pregnant women and children [188,216,236]. Across the majority of these studies, the effect of the voucher scheme was notably pronounced among the poorest populations, fostering more equity in healthcare utilization with the exception of one study where a voucher program aiming at providing insecticide-treated nets for pregnant women reported coverage of only 18% among the poorest compared to 37% among the richest [188].

Quality of care (n = 10): Seven studies showed that voucher schemes helped to improve the comfort of the patients, thus increasing their satisfaction and improving the interpersonal skills of the healthcare workforce [193,203,210,221,231,240,246]. However, one study showed that vouchers increased the workload for public health facilities [224]. Two studies showed that the current evidence is inconclusive on the effect of voucher programs on the quality of care [180,181].

Health expenditures (n = 6): Four studies agreed that voucher schemes helped reduce the out-of-pocket expenditure on healthcare services endured by women and decreased the financial barrier to access to maternal and sexual health services. [185,195,200,229]. One study highlighted that an SMS money transfer system can successfully reimburse healthcare providers located in remote and rural areas [182]. One study showed that the effect of vouchers on low-income households’ health spending is more substantial when coupled with health equity funds [206].

Mortality (n = 3): Two studies reported that vouchers helped to reduce maternal and newborn mortality rates [197] as well as malaria-related under-five mortality rates in the context of an insecticide-treated net voucher [220]. However, one study showed that a voucher scheme implemented in Bangladesh did not improve stillbirths, neonatal or infant mortality rates [245].

Health knowledge (n = 3): Two studies concluded that voucher schemes enhanced the knowledge and awareness of voucher receivers on contraceptives and STIs [183,201]. One study, however, showed that a voucher scheme had no effect on enhancing adolescent girls’ knowledge about STIs [211].

Morbidity (n = 1): One systematic review reported that the impact of vouchers on the overall health status of women was inconclusive since improvements in health outcomes require more time to manifest and may not be apparent within the designated evaluation time [189].

Child development (n = 1): One study conducted within a refugee camp in Bangladesh showed that the substitution of food rations with electronic food vouchers was linked to enhanced linear growth among children aged between six years and 23 months [217].

Implementation considerations (n = 8): Eight studies explored facilitators to the implementation of voucher programmes. Facilitators included: designing a context-specific programme [213,235]; sensitizing the community [233]; and integrating vouchers within other programmes [207]. Four other studies identified barriers, including the complex eligibility procedures used in determining beneficiaries that might at times mean missing out the poorest beneficiaries, to the implementation of voucher schemes [234]. Other barriers included: a lack of knowledge among the target population about the services provided by the voucher program [237]; the significant amount of time required to achieve awareness and uptake of the program [184]; and the high cost of starting a mobile e-voucher program that requires software systems and staff training [243].

### Studies assessing several interventions (n = 28)

28 studies addressed several financing interventions whether implemented separately or as part of a multi-component intervention. 19 studies, mainly literature reviews, assessed the use of different financing interventions including vouchers, user fee exemptions, cash transfers, the introduction of user fees, community healthcare plans, social protection schemes and insurance schemes that were either implemented in different countries or the same country but at different time periods or in different regions of the country [254–272]. The outcomes assessed were healthcare utilization [254–264,266,269,271], quality of care [254,258,259,264,268,269], healthcare expenditure [261,265,271], child development [267] and insurance coverage [256] as well as implementation considerations [255,257,268–270,272].

Five studies compared the effectiveness of CCT versus UCT interventions in Zimbabwe and other LMICs [247–249,431]. Four studies assessed the combination of different financing interventions to address health inequities [250–253]. A study conducted in Kenya found that the combination of a voucher and a CCT programme led to an increase in facility-based deliveries with 48% of the women using these schemes and subsequently delivering in a hospital or a clinic [250]. Another study assessing the use of a UCT programme paired with health insurance found that this combination increased the access of vulnerable households to healthcare services by reducing cost barriers [251]. The study evaluating the use of PBF in addition to user fee exemption found that despite the increase in facility-based deliveries due to these interventions, caesarean section rates remained alarmingly low (3%) and below the WHO recommendation [252].

Finally, according to a study assessing the combination of public insurance, a pay-for-performance scheme and a CCT program in Argentina, there was a significant decrease in the prevalence of stunting and underweight among children enrolled in these schemes [253]. However, stunting and obesity remained more common among rural populations [253]. One study assessing the effect of the a PBF program and a user fee removal policy on out-of-pocket expenditure found that user fee exemption can reduce out-of-pocket costs while there was no substantial effect of PBF [430].

### Cost-sharing (n = 20)

Cost-sharing has been used by the public health sector in several LMICs to partially redeem the expenses associated with providing healthcare services [273]. 20 studies that evaluated the effectiveness of cost-sharing and subsidies on healthcare utilization, health expenditures, morbidity, quality of care, and mortality, as well as implementation considerations were included in this scoping review [273–291,432].

Healthcare utilization (n = 11): 11 studies reported a positive impact of cost-sharing and subsidies on healthcare utilization with an increase in institutional deliveries [275–278,281], use of child health services [283], skilled birth attendance rates [280,288], households’ use of different health services [273,284] and consumption of fortified packaged complementary food (FPCF) in children [282].

Health expenditures (n = 5): Four studies reported that cost-sharing schemes led to a reduction in poor households’ facility-based delivery and reproductive health medical expenses [281,285,291] as well as a reduction in the societal cost of micronutrient deficiencies in children [282]. One study reported that despite the implementation of a cost-sharing scheme in Kenya, spending on family planning and reproductive health remained disproportionately borne by households with an increase in out-of-pocket expenses from 10% in 2005–06 to 14% in 2009–10 [274].

Morbidity (n = 2): Two studies reported a positive effect of cost-sharing policies on morbidity in women and children by reducing incidence and prevalence of reproductive tract infections and reducing the number of disability-adjusted life years due to iodine deficiency, vitamin A deficiency and iron-deficiency anemia in children aged six to 23 months [279,282].

Quality of care (n = 2): Two studies reported a positive effect of subsidy policies on the quality of care provided to women delivering in hospitals [281,291].

Mortality (n = 2): One study found significant reductions in national under-five mortality following a targeted subsidization of case management of under-five malaria [432] while another study conducted in Burkina Faso reported a non-significant decrease in deaths per live births [275].

Implementation considerations (n = 5): Five studies reported on implementation considerations for the cost-sharing policies [286–290]. A subsidy program implemented in Peru to improve the follow-up of cervical cytology was highly accepted by lower-income women [290]. The interaction between health workers and women is an important factor in determining their use of facility-based deliveries in light of a subsidy policy [286]. Moreover, three studies addressed implementation gaps which included health workers taking advantage of the subsidy policy to overestimate the cost of deliveries and incur financial gains [288], inadequate enforcement and organizational capacity [289] and limited understanding of the power dynamics between individuals representing the health system and communities [287].

### Community-based health insurance (CBHI) (n = 15)

CBHI is a form of health insurance that revolves around mutual assistance, solidarity and collective risk pooling. It offers flat rates to workers in the informal sector and rural communities [302]. 15 studies that evaluated the effectiveness of CBHI on healthcare utilization, health expenditures, morbidity, and mortality and implementation considerations were included in this scoping review [292–304,434,435].

Healthcare utilization (n = 9): Seven studies found a positive impact of CBHI on increasing the utilization of health services mainly when it came to institutional deliveries, antenatal and postnatal care, vaccinations among the poorest, and increasing the likelihood of mothers seeking healthcare services for child illnesses [292–294,297,300,302,303]. One systematic review found that the CBHI had a negative impact on the use of family planning and reproductive health services [296] and one study found no significant effects of CBHI on the utilization of maternal and child healthcare services [435].

Health expenditures (n = 5): Four studies reported that CBHI had a positive effect on reducing out-of-pocket expenditures at the point of service [293], providing substantial financial protection to households [300,304] and decreasing annual health expenditure [294]. One study reported that the high cost of transportation added to the cost of inpatient care was enough to prevent insured women with a low-income from being hospitalized [298].

Morbidity (n = 3): Two studies reported that household participation in a CBHI scheme was associated with a lower likelihood of stunting in offspring [299,302]. Another study reported that in terms of the distribution of stunting, children in the lowest socioeconomic welfare index had a higher prevalence of stunting and that there was no statistically significant association between CBHI participation and stunting [295].

Mortality (n = 2): One study conducted in Burkina Faso reported no significant difference in overall mortality rates between households enrolled and not enrolled in the CBHI scheme [292] while another study reported that the intervention was associated with lowering of the risk of mortality in children enrolled in health insurance when compared to those not enrolled [294].

Implementation considerations (n = 2): One study conducted in Cameroon reported that income was a barrier to women’s enrollment in health insurance schemes, which emphasizes the need for collaboration between health insurance programmes and governments in providing a financial safety net that will lessen the toll of illness on the underprivileged [301]. Another study suggested the need to increase women’s understanding of the CBHI system to develop their trust and enable recognition of its benefits [434].

### PBF (n = 10)

PBF is a supply-side intervention that offers incentives to healthcare providers upon the achievement of a set of objectives [312]. 10 studies that evaluated the effectiveness of PBF on healthcare utilization, quality of care, morbidity, resource availability and implementation considerations were included in this scoping review [305–313,433].

Healthcare utilization (n = 6): Six studies found no significant impact of PBF on increasing the utilization of maternal and child health services for institutional deliveries, antenatal and postnatal care and vaccinations among the poorest [305,306,309–311,433]. One study found that the effect of PBF is greater when supplemented by maternal vouchers that eliminate user fees [310]. Another study found that combining PBF with equity interventions, such as systematic targeting and subsidizing health services, did not improve the utilization of maternal health services. The study suggested that financial incentives for providers might not be enough to improve equity as other non-financial barriers to access, including distance to catchment primary health facility, literacy, parity and religion, should be considered [311].

Quality of care (n = 4): Four studies reported contradictory results in regards to the effect of PBF on the quality of care. Two studies found positive effects of PBF on improving the quality of care, specifically in improving the quality of treatment received by children from lower-income backgrounds [306] and on the motivation of health providers in delivering care to lower-income women. One study conducted in Malawi showed that PBF had neither a positive nor a negative effect on the quality of the antenatal care services provided by healthcare providers [313]. Another study on PBF covering under-five curative care, child vaccination and growth monitoring visits in Burkina Faso showed no impact on the quality of care [305,306,312].

Morbidity (n = 2): Two studies conducted in Burundi [308] and Rwanda [306] reported no significant effectiveness of PBF in reducing morbidity such as fever, diarrhea and malnutrition in children [306,308].

Resource availability (n = 1): One study conducted in Cameroon showed that PBF is associated with a significant reduction in stock-outs of family planning medicines but not in stock-outs of antenatal care drugs, vaccines, integrated management of childhood illness drugs and labour and delivery drugs [307].

Implementation considerations (n = 1): One study reported that the effectiveness of any PBF scheme addressing inequity specific to maternal health depends on the different stakeholders’ sharing a common understanding of what poor and vulnerable means as well as on the prompt payment of incentives to healthcare facilities and providers [312].

### UCTs (n = 7)

UCT programmes provide low-income and vulnerable populations with additional income without imposing any requirements on the recipients [320]. Seven studies that evaluated the effectiveness of UCTs on morbidity, healthcare utilization, health expenditures, nutritional status and child development were included in this scoping review [314–320].

Morbidity (n = 4): Two studies found a favorable effect of UCTs on the likelihood of having experienced any disease in children [320] and on the anthropometric measures in children [319]. Another two studies found no effects of UCT programmes on the prevalence of malnutrition, incidence of wasting or stunting in children [316,318].

Health expenditures (n = 2): One study conducted in Burkina Faso reported that the availability of UCTs supported families in spending more on healthcare to support children’s health [317]. One systematic review found that UCTs may help improve healthcare expenditures in LMICs [320].

Healthcare utilization (n = 2): One study noticed better access to maternal health services, specifically skilled attendance at birth, as a result of UCTs [315] while another study reported that UCTs had no effect on the utilization of health services among children and adults [320].

Nutrition (n = 2): Two studies mentioned that UCTs improved the quality of children’s diets and increased the food availability in the house [317] as well as households’ access to food [320].

Child development (n = 1): One study reported that a UCT programme had a preventive effect on children’s growth in Togo [314].

### Introduction of user fees (n = 5)

Across many sub-Saharan African countries user fees are paid directly at the point-of-care as the introduction of fees generates revenue for health systems and may decrease the demand for unnecessary care [321,323]. Five studies that evaluated the effect of the introduction of user fees on healthcare utilization were included in this study [321–325].

Healthcare utilization (n = 5): Three studies reported that this policy decreased the utilization of inpatient care by women and children in the rural areas of Zambia [321], decreased the use of health services and presentation for care by women in Mali [323] and decreased the number of skilled birth deliveries and antenatal care in Malawi [325]. One study reported an increase in the use of cervical screening despite the introduction of a fee-for-service policy [322]. Another study reported that the increase in prices did not have any disproportionate effect on the use of family planning and reproductive health services among lower-income women [324].

### Micro-credit (n = 2)

Microcredit extends loans to the impoverished without requiring collateral thereby including those who would o’t have been able to access credit otherwise [405]. One study reported that microcredit positively affected child mortality [405]. Another study highlighted that microcredit can complement health insurance in enhancing child health by increasing households’ affordability for out-of-pocket health expenditures, protecting them from the financial risks associated with health insurance plans and improving rural households’ access to insurance plans [326].

### Equity considerations

The scoping review’s findings highlight that CCT and voucher programmes can improve the utilization of maternal and child health services as well as the health outcomes for the poor and the disadvantaged. However, multiple implementation challenges impact the effectiveness of these programmes such as the complex eligibility procedures, unsustainability and misuse of transfers. Other barriers included irregular cash transfers, unclear payment mechanisms, and cultural barriers [24]. An increased workload and the insufficient healthcare personnel and infrastructure can also limit the effectiveness of CCT and voucher programmes in providing access to high-quality care to vulnerable groups. The various insurance schemes were found to have positive effect on healthcare utilization, healthcare expenditures, mortality rates and morbidity especially among the vulnerable populations including the poor, those residing in rural areas and other vulnerable populations. Few studies suggested that inequities in health coverage persisted under these programmes and schemes among the lower-income, less educated and those residing in remote regions, implying that demand-side interventions alone are insufficient in achieving equity. This also could be attributed to the persistence of other financial barriers and a lack of awareness about the programmes.

Findings showed that combining demand-side interventions with supply-side interventions can have better effect on maternal and child health outcomes [33,408]. Although user fee exemption of healthcare was found to have a positive impact on increasing utilization for the lower-income, leading to more equity, its effect is limited due to other financial and non-financial barriers. PBF was also found to have a limited impact on increasing utilization of maternal and child health services among the poorest, suggesting that financial incentives for providers might not be enough to improve equity due to non-financial barriers to access at the demand side. Not enough evidence was identified to draw conclusions on the other financial interventions such as UCTs, the introduction of user fees and microcredit. Also, evidence on the effect of financial interventions on improving equity for those living with a disablity, those less educated and LGBTIQ+ community was insufficient to draw any conclusions.

## Discussion

### Principal findings and research gaps

This scoping review mapped the literature on financing interventions and measures aiming to improve equity for WCAH. This review can inform the agendas of funders and researchers working in the field of WCAH of potential knowledge gaps. The review highlighted the lack of studies assessing financial interventions targeting adolescents and newborns; the majority of the studies focused on women and children.

The review’s findings show that most of the included studies assessed CCTs, health insurance, vouchers and user fee exemptions while fewer studies addressed supply-side interventions such as PBF. The findings highlight that even when financial barriers are removed, inequities can persist due to other non-financial barriers to access including distance to catchment primary health facility, literacy, parity and religion. Financial interventions and measures are not enough unless coupled with other non-financial interventions that address barriers such as knowledge and geographical barriers. The findings suggest a strong synergy between the effects of supply-side and demand-side incentives. The engagement of concerned stakeholders is crucial for the successful implementation of financial interventions and design of financial interventions should be context-specific. Maintaining the sustainability of the effect and the quality of care while implementing financial interventions is crucial to achieving a positive impact on the target population. Successful implementations require adequate health system inputs, such as a capable, motivated and sufficient health workforce, and provision of reimbursements, supplies and equipment. The findings can inform the design of equitable health financing policies and health system reform efforts essential in moving toward UHC [436].

This review highlighted the scarcity of studies addressing the effect of financial interventions on addressing inequities related to education, age, disability and sexual orientation. The included studies mainly focused on financing mechanisms aiming to improve socioeconomics and place of residence disparities. This finding reflects a gap in evidence of financing mechanisms targeted at people living with a disability and LGBTIQ+ communities. Other reviews also focused on equity of health financing based on SES and place of residence but with a lesser focus on other equity components [437,438].

Most of the studies were conducted by authors affiliated with institutions based in high-income countries. This can be interpreted as a result of limited research capacity in low-income countries to conduct health policy and systems research. This finding corroborates other studies and reviews, highlighting the imbalance of research capacity between high-income and low-income countries [439–441]. This scoping review found that studies are mostly funded by governments, academic institutions, private not-for-profits and international organizations.

### Strengths and limitations

To our knowledge, this scoping review is the first to map financing interventions and measures to improve equity in WCAH in LMICs. One strength of the review is that it followed JBI guidance for conducting scoping reviews [18] and the PRISMA-ScR for reporting scoping reviews [19]. One limitation is that the review aimed to include studies addressing at least one PROGRESS-plus equity component without due attention to the general pattern of inequity in low-income and LMICs where 91.9% of the global population living in extreme poverty, namely with less than US$ 2.15 per day, resides [442]. Another limitation is the fact that we did not search non-English databases although we did include non-English articles.

### Implications on policy and research

The latest data on progress towards the SDGs shows that vulnerable populations are not doing well with WCAH outcome improvements. The COVID-19 pandemic and climate change, which impact the most vulnerable the hardest, including those living in conflict settings, pose challenges. These intersectional issues amplify the exclusion and marginalization of the most vulnerable. Dedicated policy efforts are required to redress the imbalance. This review provides evidence on different financing interventions that may be used to address the needs of the most vulnerable communities and improve equity. The review can be used to inform future research on financing interventions and measures to improve equity when addressing WCAH in LMICs by revealing current knowledge gaps.

## Supporting information

S1 Appendix List of LMICs as per the World Bank Country and Lending Groups classification by income (July 2022). (DOCX)

S2 Appendix Search strategy. (DOCX)

S3 Appendix Composite humanitarian and fragile settings classification. (DOCX)

S1 Table Conditional Cash Transfer (CCT). (DOCX)

S2 Table Health insurance. (DOCX)

S3 Table User fee exemptions. (DOCX)

S4 Table Vouchers. (DOCX)

S5 Table Several interventions. (DOCX)

S6 Table Cost-sharing and subsidies. (DOCX)

S7 Table Community-Based Health Insurance (CBHI). (DOCX)

S8 Table Performance-based financing (PBF). (DOCX)

S9 Table Unconditional Cash Transfer (UCT). (DOCX)

S10 Table Introduction of user fees. (DOCX)

S11 Table Microcredit. (DOCX)
