# Supplementary material for: Financing for equity for women’s, children’s and adolescents’ health in low- and middle-income countries: A scoping review
Source: PLOS Glob Public Health. 2024 Sep 12;4(9):e0003573. doi: 10.1371/journal.pgph.0003573 (PMC11392393; doi:10.1371/journal.pgph.0003573)
Supplement: S1 Appendix — (DOCX) [file pgph.0003573.s001.docx]

**S1 Appendix**

List of LMICs as per the World Bank Country and Lending Groups classification by income (July 2022)

| **Upper-middle-income** | **Lower-middle-income** | **Low-income** |
| --- | --- | --- |
| Albania  American Samoa  Argentina  Armenia  Azerbaijan  Belarus  Belize  Bosnia and Herzegovina  Botswana  Brazil  Bulgaria  China  Colombia  Costa Rica  Cuba  Dominica  Dominican Republic  Ecuador  Equatorial Guinea  Fiji  Gabon  Georgia  Grenada  Guatemala  Guyana  Indonesia  Iran, Islamic Rep.  Iraq  Jamaica  Jordan  Kazakhstan  Kosovo  Lebanon  Libya  Malaysia  Maldives  Marshall Islands  Mexico  Montenegro  Namibia  North Macedonia  Paraguay  Peru  Russian Federation  Samoa  Serbia  South Africa  St. Lucia  St. Vincent and the Grenadines  Suriname  Thailand  Tonga  Turkey  Turkmenistan  Tuvalu  Venezuela, RB | Algeria  Angola  Bangladesh  Benin  Bhutan  Bolivia  Cabo Verde  Cambodia  Cameroon  Comoros  Congo, Rep.  Côte d'Ivoire  Djibouti  Egypt, Arab Rep.  El Salvador  Eswatini  Ghana  Honduras  India  Kenya  Kiribati  Kyrgyz Republic  Lao PDR  Lesotho  Mauritania  Micronesia, Fed. Sts.  Moldova  Mongolia  Morocco  Myanmar  Nepal  Nicaragua  Nigeria  Pakistan  Papua New Guinea  Philippines  São Tomé and Principe  Senegal  Solomon Islands  Sri Lanka  Tanzania  Timor-Leste  Tunisia  Ukraine  Uzbekistan  Vanuatu  Vietnam  West Bank and Gaza  Zambia  Zimbabwe | Afghanistan  Burkina Faso  Burundi  Central African Republic  Chad  Congo, Dem. Rep.  Eritrea  Ethiopia  Gambia, The  Guinea  Guinea-Bissau  Haiti  Korea, Dem. People's Rep.  Liberia  Madagascar  Malawi  Mali  Mozambique  Niger  Rwanda  Sierra Leone  Somalia  South Sudan  Sudan  Syrian Arab Republic  Tajikistan  Togo  Uganda  Yemen, Rep. |
