# Supplementary material for: Financing for equity for women’s, children’s and adolescents’ health in low- and middle-income countries: A scoping review
Source: PLOS Glob Public Health. 2024 Sep 12;4(9):e0003573. doi: 10.1371/journal.pgph.0003573 (PMC11392393; doi:10.1371/journal.pgph.0003573)
Supplement: S2 Appendix — (DOCX) [file pgph.0003573.s002.docx]

**S2_Appendix**

**Search strategies**

**Database: Ovid MEDLINE(R) and Epub Ahead of Print, In-Process, In-Data-Review & Other Non-Indexed Citations and Daily <1946 to November 22, 2023>**
**Search Strategy:**
**1**  insurance benefits/ or insurance coverage/ or prepaid health plans/ (18398)
**2**  insurance, health/ or children's health insurance program/ or community-based health insurance/ or single-payer system/ or exp insurance, health, reimbursement/ (85078)
**3**  Healthcare Financing/ (1285)
**4**  (financ* adj2 (mechanism? or strategy or strategies or scheme? or method or methods or protection or system or systems or policy or policies or reform or reforms or plan* or statement?)).ti,ab. (8955)
**5**  (payment? adj2 (mechanism? or strategy or strategies or scheme? or method* or system? or polic* or plan*)).ti,ab. (7276)
**6**  ((prepaid or (pre adj paid) or pre-payment?) adj2 (scheme? or plan or plans or coverage or insurance or package?)).ti,ab. (501)
**7**  ((targeted or direct) adj2 payment?).ti,ab. (335)
**8**  (insurance adj2 (scheme? or plan or plans or coverage or mechanism? or package? or system?)).ti,ab. (20737)
**9**  ((social or (community adj based) or health*) adj2 (insurance or coverage)).ti,ab. (65181)
**10**  (out-of-pocket or "out of pocket" or OOP or subsidy or subsidies or subsidized or "conditional cash transfer" or "conditional cash transfers" or co-payment? or premium? or deductible? or "cost sharing" or cost-sharing or capitation or taxation or tax or taxes or fee-exemption or user-fee or user-fees or "user fees" or "user fee" or single-payer or fee-for-service or "fee for service" or reimburs* or voucher?).ti,ab. (86851)
**11**  (donor? adj2 (funding or support or pool? or contribution?)).ti,ab. (4408)
**12**  ((fee or fees) adj2 (exemption or reduction or removal or reducing)).ti,ab. (299)
**13**  ("official development assistance" or ODA).ti,ab. (1199)
**14**  (health* adj benefit adj package?).ti,ab. (78)
**15**  (government* adj2 fund*).ti,ab. (3599)
**16**  (performance-based adj2 financing).ti,ab. (223)
**17**  (health* adj equity adj fund?).ti,ab. (33)
**18**  (financial adj2 coverage).ti,ab. (364)
**19**  or/1-18 (232693)
**20**  Women/ or adolescent health/ or child health/ or infant health/ or reproductive health/ or sexual health/ or child health services/ or family planning services/ (76361)
**21**  (wom?n or child or children or infant? or mother? or maternal or newborn? or new-born? or neonat* or teen* or adolescen* or youth or young or youngster).ti,ab. (3996403)
**22**  ((thirteen or fourteen or fifteen or sixteen or seventeen or eighteen or "13" or "14" or "15" or "16" or "17" or "18") adj ((years adj old) or yo or "y.o.")).ti,ab. (37543)
**23**  ((prenatal or postnatal or postpartum or antenatal or intrapartum or abortion or post-abortion or delivery or deliveries) adj2 (care or service?)).ti,ab. (82016)
**24**  (HIV adj2 (testing or service? or counsel?ing)).ti,ab. (22447)
**25**  ((family adj planning) or contraception?).ti,ab. (50100)
**26**  (sexual adj reproductive adj health).ti,ab. (269)
**27**  ((gender-based or domestic or (intimate adj partner)) adj violence).ti,ab. (18881)
**28**  (adolescent/ or child/ or infant/) and (care or service? or health* or wellbeing or well-being).ti,ab. (794598)
**29**  or/20-28 (4385919)
**30**  30 algeria/ or egypt/ or libya/ or morocco/ or tunisia/ or cameroon/ or central african republic/ or chad/ or congo/ or equatorial guinea/ or gabon/ or "sao tome and principe"/ or burundi/ or djibouti/ or eritrea/ or ethiopia/ or kenya/ or rwanda/ or somalia/ or south sudan/ or sudan/ or tanzania/ or uganda/ or angola/ or botswana/ or eswatini/ or lesotho/ or malawi/ or mozambique/ or namibia/ or south africa/ or zambia/ or zimbabwe/ or benin/ or burkina faso/ or cabo verde/ or cote d'ivoire/ or gambia/ or ghana/ or guinea/ or guinea-bissau/ or liberia/ or mali/ or mauritania/ or niger/ or nigeria/ or senegal/ or sierra leone/ or togo/ or cuba/ or dominica/ or dominican republic/ or grenada/ or haiti/ or jamaica/ or saint lucia/ or "saint vincent and the grenadines"/ or belize/ or costa rica/ or el salvador/ or guatemala/ or honduras/ or nicaragua/ or mexico/ or argentina/ or bolivia/ or brazil/ or colombia/ or ecuador/ or guyana/ or paraguay/ or peru/ or suriname/ or venezuela/ or kazakhstan/ or kyrgyzstan/ or tajikistan/ or turkmenistan/ or uzbekistan/ or russia/ or cambodia/ or indonesia/ or malaysia/ or myanmar/ or philippines/ or thailand/ or timor-leste/ or vietnam/ or bangladesh/ or bhutan/ or india/ or afghanistan/ or iran/ or iraq/ or jordan/ or lebanon/ or syria/ or turkey/ or yemen/ or nepal/ or pakistan/ or sri lanka/ or china/ or "democratic people's republic of korea"/ or mongolia/ or "Republic of North Macedonia"/ or Comoros/ or Micronesia/ or American Samoa/ or Serbia/ or Tonga/ or Madagascar/ or Moldova/ or Papua New Guinea/ or Ukraine/ or Vanuatu/ or Albania/ or Armenia/ or Azerbaijan/ or "Republic of Belarus"/ or Bulgaria/ or Fiji/ or Georgia/ or Kosovo/ or Montenegro/ or "Bosnia and Herzegovina"/ (1214903)
**31**  (Afghanistan* or "Burkina Faso" or Burundi or "Central African Republic" or Chad* or Congo or Eritrea or Ethiopia or Gambia or Guinea or "Guinea-Bissau" or Haiti or "Democratic People's Republic of Korea" or Liberia or Madagascar or Malawi or Mali or Mozambique or Niger or Rwanda or "Sierra Leone" or Somalia* or Sudan or "Syrian Arab Republic" or Syria* or Tajikistan or Togo or Uganda or Yemen or "North Korea").ti,ab. (266279)
**32**  (Algeria* or Angola or Bangladesh* or Benin or Bhutan or Bolivia* or "Cabo Verde" or Cambodia or Cameroon or Comoros or Congo or "Côte d'Ivoire" or "Ivory Coast" or Djibouti* or Egypt* or "El Salvador" or Eswatini or Ghana or Honduras or India or Kenya* or Kiribati or "Kyrgyz Republic" or "Lao PDR" or Lesotho or Mauritania or Micronesia or Moldova or Mongolia or Morocco* or Myanmar or Nepal* or Nicaragua or Nigeria? or Pakistan* or "Papua New Guinea" or Philippin* or "São Tomé and Principe" or Senegal or "Solomon Islands" or "Sri Lanka" or Tanzania* or "Timor-Leste" or Tunisi* or Ukrain* or Uzbekistan or Vanuatu or Vietnam* or "West Bank" or G?az?a or Zambia* or Zimbabwe).ti,ab. (466672)
**33**  (Albania* or "American Samoa" or Argentin* or Armenia* or Azerbaijan or Belarus or Belize or "Bosnia and Herzegovina" or Botswana or Brazil or Bulgaria* or China or Colombia* or "Costa Rica" or Cuba or Dominica or "Dominican Republic" or Ecuador or "Equatorial Guinea" or Fiji or Gabon or Georgia or Grenada or Guatemala or Guyana or Indonesia* or Iran* or Iraq* or Jamaica* or Jordan* or Kazakhstan or Kosovo or Leban* or Libya* or Malaysia* or Maldives or "Marshall Islands" or Mexico or Montenegro or Namibia* or "North Macedonia" or Paraguay or Peru or Russia* or Samoa or Serbia or "South Africa" or "Saint Lucia" or "St. Lucia" or "Saint Vincent and the Grenadines" or "St. Vincent and the Grenadines" or Suriname or Thailand or Tonga or Turkey or Turkmenistan or Tuvalu or Venezuela).ti,ab. (870701)
**34**  ((low or middle) adj2 countr*).ti,ab. (49342)
**35**  Developing Countries/ (81483)
**36**  ((developing or (less* adj developed) or (least adj developed) or under-developed or underdeveloped or "under developed" or underserved or "under served" or deprived or poor* or transition*) adj2 (countr* or nation? or population? or world or econom* or communit* or state? or people or cities or city)).ti,ab. (149809)
**37**  or/30-36 (2057634)
**38**  exp health services accessibility/ or healthcare disparities/ or exp disabled persons/ or exp educational status/ or exp socioeconomic factors/ or exp minority groups/ or exp ethnic groups/ or exp urban health/ or exp urban population/ or exp sex factors/ or exp geriatrics/ or exp homosexuality/ (1136931)
**39**  ((poverty or low-income or socioeconomic* or socio-economic* or social or ethnic$ or race or racial or religio$ or cultur$ or minorit$ or refugee or indigenous or aboriginal or sex or gender* or urban or rural or inner-city or slum) adj2 (difference$ or factor$ or discriminat$)).ti,ab. (212071)
**40**  (equit* or inequi* or fair* or disparit* or disadvantage* or inequalit*).ti,ab. (380271)
**41**  (Social adj (exclusion or inclusion)).ti,ab. (4006)
**42**  38 or 39 or 40 or 41 (1553193)
**43**  19 and 29 and 37 and 42 (5140)
**44**  limit 43 to ez="20000101-20211231" (4029)
**45**  limit 43 to ez="20210720-20220630" (316)
**46**  limit 43 to ez="20220630-20230530" (270)

**Global Health**

**Update 2023- 9**

|  | Date (GMT) | Results | |  |
| --- | --- | --- | --- | --- |
| Search term | 21 Jul 2021 14:43 | | 323 | |
| [((title:((Social near/1 (exclusion OR inclusion))) OR ab:((Social near/1 (exclusion OR inclusion)))) OR (title:(equit* OR inequi* OR fair* OR disparit* OR disadvantage* OR inequalit*) OR ab:(equit* OR inequi* OR fair* OR disparit* OR disadvantage* OR inequalit*)) OR (title:(((poverty OR low-income OR socioeconomic* OR socio-economic* OR social OR ethnic* OR race OR racial OR religio* OR cultur* OR minorit* OR refugee OR indigenous OR aboriginal OR sex OR gender* OR urban OR rural OR inner-city OR slum) near/2 (difference* OR factor* OR discriminat*))) OR ab:(((poverty OR low-income OR socioeconomic* OR socio-economic* OR social OR ethnic* OR race OR racial OR religio* OR cultur* OR minorit* OR refugee OR indigenous OR aboriginal OR sex OR gender* OR urban OR rural OR inner-city OR slum) near/2 (difference* OR factor* OR discriminat*)))) OR ("disabled persons" OR "socioeconomic status" OR "ethnic groups" OR "urban population" OR "geriatrics" OR "homosexuality")) AND ((title:(((developing OR less-developed OR lesser-developed OR least-developed OR under-developed OR underdeveloped OR under-developed OR underserved OR under-served OR deprived OR poor* OR transition*) near/2 (countr* OR nation* OR population* OR world OR econom* OR communit* OR state* OR people OR cities OR city))) OR ab:(((developing OR less-developed OR lesser-developed OR least-developed OR under-developed OR underdeveloped OR under-developed OR underserved OR under-served OR deprived OR poor* OR transition*) near/2 (countr* OR nation* OR population* OR world OR econom* OR communit* OR state* OR people OR cities OR city)))) OR (title:(Albania* OR “American Samoa” OR Argentin* OR Armenia* OR Azerbaijan OR Belarus OR Belize OR “Bosnia and Herzegovina” OR Botswana OR Brazil OR Bulgaria* OR China OR Colombia* OR “Costa Rica” OR Cuba OR Dominica OR “Dominican Republic” OR Ecuador OR “Equatorial Guinea” OR Fiji OR Gabon OR Georgia OR Grenada OR Guatemala OR Guyana OR Indonesia* OR Iran* OR Iraq* OR Jamaica* OR Jordan* OR Kazakhstan OR Kosovo OR Leban* OR Libya* OR Malaysia* OR Maldives OR “Marshall Islands” OR Mexico OR Montenegro OR Namibia* ) OR ab:(Albania* OR “American Samoa” OR Argentin* OR Armenia* OR Azerbaijan OR Belarus OR Belize OR “Bosnia and Herzegovina” OR Botswana OR Brazil OR Bulgaria* OR China OR Colombia* OR “Costa Rica” OR Cuba OR Dominica OR “Dominican Republic” OR Ecuador OR “Equatorial Guinea” OR Fiji OR Gabon OR Georgia OR Grenada OR Guatemala OR Guyana OR Indonesia* OR Iran* OR Iraq* OR Jamaica* OR Jordan* OR Kazakhstan OR Kosovo OR Leban* OR Libya* OR Malaysia* OR Maldives OR “Marshall Islands” OR Mexico OR Montenegro OR Namibia* )) OR (title:("North Macedonia” OR Paraguay OR Peru OR Russia* OR Samoa OR Serbia OR “South Africa” OR “Saint Lucia” OR “St. Lucia” OR “Saint Vincent and the Grenadines” OR “St. Vincent and the Grenadines” OR Suriname OR Thailand OR Tonga OR Turkey OR Turkmenistan OR Tuvalu OR Venezuela) OR ab:("North Macedonia” OR Paraguay OR Peru OR Russia* OR Samoa OR Serbia OR “South Africa” OR “Saint Lucia” OR “St. Lucia” OR “Saint Vincent and the Grenadines” OR “St. Vincent and the Grenadines” OR Suriname OR Thailand OR Tonga OR Turkey OR Turkmenistan OR Tuvalu OR Venezuela)) OR ("Developing Countries") OR (title:(Algeria* OR Angola OR Bangladesh* OR Benin OR Bhutan OR Bolivia* OR “Cabo Verde” OR Cambodia OR Cameroon OR Comoros OR Congo OR “Côte d'Ivoire” OR “Ivory Coast” OR Djibouti* OR Egypt* OR “El Salvador” OR Eswatini OR Ghana OR Honduras OR India OR Kenya* OR Kiribati OR “Kyrgyz Republic” OR “Lao PDR” OR Lesotho OR Mauritania OR Micronesia OR Moldova OR Mongolia OR Morocco* OR Myanmar OR Nepal* OR Nicaragua OR Nigeria* OR Pakistan* OR “Papua New Guinea” OR Philippin* OR “São Tomé and Principe” OR Senegal OR “Solomon Islands” OR “Sri Lanka” OR Tanzania* OR “Timor-Leste” OR Tunisi* OR Ukrain* OR Uzbekistan OR Vanuatu OR Vietnam* OR “West Bank” OR Ghaza OR ghaza OR gaza OR gazza OR Zambia* OR Zimbabwe) OR ab:(Algeria* OR Angola OR Bangladesh* OR Benin OR Bhutan OR Bolivia* OR “Cabo Verde” OR Cambodia OR Cameroon OR Comoros OR Congo OR “Côte d'Ivoire” OR “Ivory Coast” OR Djibouti* OR Egypt* OR “El Salvador” OR Eswatini OR Ghana OR Honduras OR India OR Kenya* OR Kiribati OR “Kyrgyz Republic” OR “Lao PDR” OR Lesotho OR Mauritania OR Micronesia OR Moldova OR Mongolia OR Morocco* OR Myanmar OR Nepal* OR Nicaragua OR Nigeria* OR Pakistan* OR “Papua New Guinea” OR Philippin* OR “São Tomé and Principe” OR Senegal OR “Solomon Islands” OR “Sri Lanka” OR Tanzania* OR “Timor-Leste” OR Tunisi* OR Ukrain* OR Uzbekistan OR Vanuatu OR Vietnam* OR “West Bank” OR Ghaza OR ghaza OR gaza OR gazza OR Zambia* OR Zimbabwe)) OR (title:(Afghanistan* OR “Burkina Faso” OR Burundi OR “Central African Republic” OR Chad* OR Congo OR Eritrea OR Ethiopia OR Gambia OR Guinea OR “Guinea-Bissau” OR Haiti OR “Democratic People's Republic of Korea” OR Liberia OR Madagascar OR Malawi OR Mali OR Mozambique OR Niger OR Rwanda OR “Sierra Leone” OR Somalia* OR Sudan OR Syria* OR Tajikistan OR Togo OR Uganda OR Yemen OR “North Korea”) OR ab:(Afghanistan* OR “Burkina Faso” OR Burundi OR “Central African Republic” OR Chad* OR Congo OR Eritrea OR Ethiopia OR Gambia OR Guinea OR “Guinea-Bissau” OR Haiti OR “Democratic People's Republic of Korea” OR Liberia OR Madagascar OR Malawi OR Mali OR Mozambique OR Niger OR Rwanda OR “Sierra Leone” OR Somalia* OR Sudan OR Syria* OR Tajikistan OR Togo OR Uganda OR Yemen OR “North Korea”)) OR ("Comoros" OR "Micronesia" OR "American Samoa" OR "Serbia" OR "Tonga" OR "Madagascar" OR "Moldova" OR "Papua New Guinea" OR "Ukraine" OR "Vanuatu" OR "Albania" OR "Armenia" OR "Azerbaijan" OR "Bulgaria" OR "Georgia" OR "Fiji" OR "Kosovo" OR "Montenegro" OR "Bosnia and Herzegovina") OR ("Algeria" OR "Egypt" OR "Libya" OR "Libyan Arab Jamahiriya" OR "Libyan Arab Republic" OR "Morocco" OR "Tunisia" OR "Cameroon" OR "Central African Republic" OR "Chad" OR "Congo" OR "Equatorial Guinea" OR "Gabon" OR "Sao Tome and Principe" OR "Burundi" OR "Djibouti" OR "Eritrea" OR "Ethiopia" OR "Kenya" OR "Rwanda" OR "Somalia" OR "South Sudan" OR "Sudan" OR "Tanzania" OR "Uganda" OR "Angola" OR "Botswana" OR "Lesotho" OR "Malawi" OR "Mozambique" OR "Namibia" OR "South Africa" OR "Zambia" OR "Zimbabwe" OR "Benin" OR "Burkina Faso" OR "Cote d'Ivoire" OR "Gambia" OR "Ghana" OR "Guinea" OR "Guinea-Bissau" OR "Liberia" OR "Mali" OR "Mauritania" OR "Niger" OR "Nigeria" OR "Senegal" OR "Sierra Leone" OR "Togo" OR "Cuba" OR "Dominica" OR "Dominican Republic" OR "Grenada" OR "Haiti" OR "Jamaica" OR "Saint Lucia" OR "Saint Vincent and the Grenadines" OR "Belize" OR "Costa Rica" OR "El Salvador" OR "Guatemala" OR "Honduras" OR "Nicaragua" OR "Mexico" OR "Argentina" OR "Brazil" OR "Colombia" OR "Ecuador" OR "Guyana" OR "Paraguay" OR "Turkmenistan" OR "Peru" OR "Suriname" OR "Venezuela" OR "Kazakhstan" OR "Tajikistan" OR "Kyrgyzstan" OR "Uzbekistan" OR "Russia" OR "Cambodia" OR "Lebanon" OR "Indonesia" OR "Malaysia" OR "Myanmar" OR "Philippines" OR "Thailand" OR "Timor-Leste" OR "Vietnam" OR "Bangladesh" OR "India" OR "Afghanistan" OR "Iran" OR "Iraq" OR "Jordan" OR "Syria" OR "Syrian Arab Republic" OR "Turkey" OR "Yemen" OR "Nepal" OR "Pakistan" OR "Sri Lanka" OR "China" OR "Democratic People's Republic of Korea" OR "Republic of North Macedonia" OR "Mongolia")) AND (((title:((care OR service* OR health* OR wellbeing OR well-being)) OR ab:((care OR service* OR health* OR wellbeing OR well-being))) AND ("adolescents" OR "children" OR "infants")) OR (title:(wom$n OR child OR children OR infant* OR mother* OR maternal OR newborn* OR "new born*" OR neonat* OR teen* OR adolescen* OR youth OR young OR youngster) OR ab:(wom$n OR child OR children OR infant* OR mother* OR maternal OR newborn* OR "new born*" OR neonat* OR teen* OR adolescen* OR youth OR young OR youngster)) OR (title:(((gender-based OR domestic OR intimate-partner) near/1 violence)) OR ab:(((gender-based OR domestic OR intimate-partner) near/1 violence))) OR (title:((sexual-reproductive-health)) OR ab:((sexual-reproductive-health))) OR (title:((family-planning OR contraception*)) OR ab:((family-planning OR contraception*))) OR (title:((HIV near/2 (testing OR service* OR counseling OR counselling))) OR ab:((HIV near/2 (testing OR service* OR counseling OR counselling)))) OR (title:(((prenatal OR postnatal OR postpartum OR antenatal OR intrapartum OR abortion OR post-abortion OR delivery OR deliveries) near/2 (care OR service*))) OR ab:(((prenatal OR postnatal OR postpartum OR antenatal OR intrapartum OR abortion OR post-abortion OR delivery OR deliveries) near/2 (care OR service*)))) OR (title:(((thirteen OR fourteen OR fifteen OR sixteen OR seventeen OR eighteen OR "13" OR "14" OR "15" OR "16" OR "17" OR "18") near/1 (years-old OR yo OR "y.o."))) OR ab:(((thirteen OR fourteen OR fifteen OR sixteen OR seventeen OR eighteen OR "13" OR "14" OR "15" OR "16" OR "17" OR "18") near/1 (years-old OR yo OR "y.o.")))) OR ("women" OR "women's health" OR "child health" OR "reproductive health" OR "sexual health")) AND ((title:(financial near/2 coverage) OR ab:(financial near/2 coverage)) OR (title:(health* near/1 equity-fund*) OR ab:(health* near/1 equity-fund*)) OR (title:(performance-based near/2 financing) OR ab:(performance-based near/2 financing)) OR (title:(government* near/2 fund*) OR ab:(government* near/2 fund*)) OR (title:(health* near/1 benefit-package*) OR ab:(health* near/1 benefit-package*)) OR (title:("official development assistance" OR ODA) OR ab:("official development assistance" OR ODA)) OR (title:((fee OR fees) near/2 (exemption OR reduction OR removal OR reducing) OR ab:((fee OR fees) near/2 (exemption OR reduction OR removal OR reducing)) OR (title:(donor* near/2 (funding OR support OR pool* OR contribution*)) OR ab:(donor* near/2 (funding OR support OR pool* OR contribution*))) OR (title:(out-of-pocket OR "out of pocket" OR OOP OR subsidy OR subsidies OR subsidized OR "conditional cash transfer" OR "conditional cash transfers" OR co-payment* OR premium* OR deductible* OR "cost sharing" OR cost-sharing OR capitation OR taxation OR tax OR taxes OR fee-exemption OR user-fee OR user-fees OR "user fees" OR "user fee" OR single-payer OR fee-for-service OR "fee for service" OR reimburs* OR voucher*) OR ab:(out-of-pocket OR "out of pocket" OR OOP OR subsidy OR subsidies OR subsidized OR "conditional cash transfer" OR "conditional cash transfers" OR co-payment* OR premium* OR deductible* OR "cost sharing" OR cost-sharing OR capitation OR taxation OR tax OR taxes OR fee-exemption OR user-fee OR user-fees OR "user fees" OR "user fee" OR single-payer OR fee-for-service OR "fee for service" OR reimburs* OR voucher*)) OR (title:((social OR community-based OR health*) near/2 (insurance OR coverage)) OR ab:((social OR community-based OR health*) near/2 (insurance OR coverage))) OR (title:(insurance near/2 (scheme* OR plan OR plans OR coverage OR mechanism* OR package* OR system*)) OR ab:(insurance near/2 (scheme* OR plan OR plans OR coverage OR mechanism* OR package* OR system*))) OR (title:(((targeted OR direct) near/2 payment*)) OR ab:(((targeted OR direct) near/2 payment*))) OR (title:(((prepaid OR pre-paid OR pre-payment*) near/2 (scheme* OR plan OR plans OR coverage OR insurance OR package*))) OR ab:(((prepaid OR pre-paid OR pre-payment*) near/2 (scheme* OR plan OR plans OR coverage OR insurance OR package*)))) OR (title:((financ* near/2 (mechanism OR strategy OR strategies OR scheme OR method OR methods OR protection OR system OR systems OR policy OR policies OR reform OR reforms OR plan* OR statement))) OR ab:((financ* near/2 (mechanism OR strategy OR strategies OR scheme OR method OR methods OR protection OR system OR systems OR policy OR policies OR reform OR reforms OR plan* OR statement)))) OR (title:((payment near/2 (mechanism OR strategy OR strategies OR scheme OR method* OR system OR polic* OR plan*))) OR ab:((payment near/2 (mechanism OR strategy OR strategies OR scheme OR method* OR system OR polic* OR plan*)))) OR ("insurance premiums" OR "health insurance"))](https://www-cabdirect-org.ezproxy.aub.edu.lb/cabdirect/search/?lq=fdb9dc1b-af9d-4c9c-9068-ed6a17c4b3ab&sort=Relevance) Refinements: | 21 Jul 2021 14:14 | | 326 | |
| [(title:((Social near/1 (exclusion OR inclusion))) OR ab:((Social near/1 (exclusion OR inclusion)))) OR (title:(equit* OR inequi* OR fair* OR disparit* OR disadvantage* OR inequalit*) OR ab:(equit* OR inequi* OR fair* OR disparit* OR disadvantage* OR inequalit*)) OR (title:(((poverty OR low-income OR socioeconomic* OR socio-economic* OR social OR ethnic* OR race OR racial OR religio* OR cultur* OR minorit* OR refugee OR indigenous OR aboriginal OR sex OR gender* OR urban OR rural OR inner-city OR slum) near/2 (difference* OR factor* OR discriminat*))) OR ab:(((poverty OR low-income OR socioeconomic* OR socio-economic* OR social OR ethnic* OR race OR racial OR religio* OR cultur* OR minorit* OR refugee OR indigenous OR aboriginal OR sex OR gender* OR urban OR rural OR inner-city OR slum) near/2 (difference* OR factor* OR discriminat*)))) OR ("disabled persons" OR "socioeconomic status" OR "ethnic groups" OR "urban population" OR "geriatrics" OR "homosexuality")](https://www-cabdirect-org.ezproxy.aub.edu.lb/cabdirect/search/?q=(title:((Social%20near/1%20(exclusion%20OR%20inclusion)))%20OR%20ab:((Social%20near/1%20(exclusion%20OR%20inclusion))))%20OR%20(title:(equit*%20OR%20inequi*%20OR%20fair*%20OR%20disparit*%20OR%20disadvantage*%20OR%20inequalit*)%20OR%20ab:(equit*%20OR%20inequi*%20OR%20fair*%20OR%20disparit*%20OR%20disadvantage*%20OR%20inequalit*))%20OR%20(title:(((poverty%20OR%20low-income%20OR%20socioeconomic*%20OR%20socio-economic*%20OR%20social%20OR%20ethnic*%20OR%20race%20OR%20racial%20OR%20religio*%20OR%20cultur*%20OR%20minorit*%20OR%20refugee%20OR%20indigenous%20OR%20aboriginal%20OR%20sex%20OR%20gender*%20OR%20urban%20OR%20rural%20OR%20inner-city%20OR%20slum)%20near/2%20(difference*%20OR%20factor*%20OR%20discriminat*)))%20OR%20ab:(((poverty%20OR%20low-income%20OR%20socioeconomic*%20OR%20socio-economic*%20OR%20social%20OR%20ethnic*%20OR%20race%20OR%20racial%20OR%20religio*%20OR%20cultur*%20OR%20minorit*%20OR%20refugee%20OR%20indigenous%20OR%20aboriginal%20OR%20sex%20OR%20gender*%20OR%20urban%20OR%20rural%20OR%20inner-city%20OR%20slum)%20near/2%20(difference*%20OR%20factor*%20OR%20discriminat*))))%20OR%20(%22disabled%20persons%22%20OR%20%22socioeconomic%20status%22%20OR%20%22ethnic%20groups%22%20OR%20%22urban%20population%22%20OR%20%22geriatrics%22%20OR%20%22homosexuality%22)&sort=Relevance) Refinements: | 21 Jul 2021 14:12 | | 352,919 | |
| [(title:(((developing OR less-developed OR lesser-developed OR least-developed OR under-developed OR underdeveloped OR under-developed OR underserved OR under-served OR deprived OR poor* OR transition*) near/2 (countr* OR nation* OR population* OR world OR econom* OR communit* OR state* OR people OR cities OR city))) OR ab:(((developing OR less-developed OR lesser-developed OR least-developed OR under-developed OR underdeveloped OR under-developed OR underserved OR under-served OR deprived OR poor* OR transition*) near/2 (countr* OR nation* OR population* OR world OR econom* OR communit* OR state* OR people OR cities OR city)))) OR (title:(Albania* OR “American Samoa” OR Argentin* OR Armenia* OR Azerbaijan OR Belarus OR Belize OR “Bosnia and Herzegovina” OR Botswana OR Brazil OR Bulgaria* OR China OR Colombia* OR “Costa Rica” OR Cuba OR Dominica OR “Dominican Republic” OR Ecuador OR “Equatorial Guinea” OR Fiji OR Gabon OR Georgia OR Grenada OR Guatemala OR Guyana OR Indonesia* OR Iran* OR Iraq* OR Jamaica* OR Jordan* OR Kazakhstan OR Kosovo OR Leban* OR Libya* OR Malaysia* OR Maldives OR “Marshall Islands” OR Mexico OR Montenegro OR Namibia* ) OR ab:(Albania* OR “American Samoa” OR Argentin* OR Armenia* OR Azerbaijan OR Belarus OR Belize OR “Bosnia and Herzegovina” OR Botswana OR Brazil OR Bulgaria* OR China OR Colombia* OR “Costa Rica” OR Cuba OR Dominica OR “Dominican Republic” OR Ecuador OR “Equatorial Guinea” OR Fiji OR Gabon OR Georgia OR Grenada OR Guatemala OR Guyana OR Indonesia* OR Iran* OR Iraq* OR Jamaica* OR Jordan* OR Kazakhstan OR Kosovo OR Leban* OR Libya* OR Malaysia* OR Maldives OR “Marshall Islands” OR Mexico OR Montenegro OR Namibia* )) OR (title:("North Macedonia” OR Paraguay OR Peru OR Russia* OR Samoa OR Serbia OR “South Africa” OR “Saint Lucia” OR “St. Lucia” OR “Saint Vincent and the Grenadines” OR “St. Vincent and the Grenadines” OR Suriname OR Thailand OR Tonga OR Turkey OR Turkmenistan OR Tuvalu OR Venezuela) OR ab:("North Macedonia” OR Paraguay OR Peru OR Russia* OR Samoa OR Serbia OR “South Africa” OR “Saint Lucia” OR “St. Lucia” OR “Saint Vincent and the Grenadines” OR “St. Vincent and the Grenadines” OR Suriname OR Thailand OR Tonga OR Turkey OR Turkmenistan OR Tuvalu OR Venezuela)) OR ("Developing Countries") OR (title:(Algeria* OR Angola OR Bangladesh* OR Benin OR Bhutan OR Bolivia* OR “Cabo Verde” OR Cambodia OR Cameroon OR Comoros OR Congo OR “Côte d'Ivoire” OR “Ivory Coast” OR Djibouti* OR Egypt* OR “El Salvador” OR Eswatini OR Ghana OR Honduras OR India OR Kenya* OR Kiribati OR “Kyrgyz Republic” OR “Lao PDR” OR Lesotho OR Mauritania OR Micronesia OR Moldova OR Mongolia OR Morocco* OR Myanmar OR Nepal* OR Nicaragua OR Nigeria* OR Pakistan* OR “Papua New Guinea” OR Philippin* OR “São Tomé and Principe” OR Senegal OR “Solomon Islands” OR “Sri Lanka” OR Tanzania* OR “Timor-Leste” OR Tunisi* OR Ukrain* OR Uzbekistan OR Vanuatu OR Vietnam* OR “West Bank” OR Ghaza OR ghaza OR gaza OR gazza OR Zambia* OR Zimbabwe) OR ab:(Algeria* OR Angola OR Bangladesh* OR Benin OR Bhutan OR Bolivia* OR “Cabo Verde” OR Cambodia OR Cameroon OR Comoros OR Congo OR “Côte d'Ivoire” OR “Ivory Coast” OR Djibouti* OR Egypt* OR “El Salvador” OR Eswatini OR Ghana OR Honduras OR India OR Kenya* OR Kiribati OR “Kyrgyz Republic” OR “Lao PDR” OR Lesotho OR Mauritania OR Micronesia OR Moldova OR Mongolia OR Morocco* OR Myanmar OR Nepal* OR Nicaragua OR Nigeria* OR Pakistan* OR “Papua New Guinea” OR Philippin* OR “São Tomé and Principe” OR Senegal OR “Solomon Islands” OR “Sri Lanka” OR Tanzania* OR “Timor-Leste” OR Tunisi* OR Ukrain* OR Uzbekistan OR Vanuatu OR Vietnam* OR “West Bank” OR Ghaza OR ghaza OR gaza OR gazza OR Zambia* OR Zimbabwe)) OR (title:(Afghanistan* OR “Burkina Faso” OR Burundi OR “Central African Republic” OR Chad* OR Congo OR Eritrea OR Ethiopia OR Gambia OR Guinea OR “Guinea-Bissau” OR Haiti OR “Democratic People's Republic of Korea” OR Liberia OR Madagascar OR Malawi OR Mali OR Mozambique OR Niger OR Rwanda OR “Sierra Leone” OR Somalia* OR Sudan OR Syria* OR Tajikistan OR Togo OR Uganda OR Yemen OR “North Korea”) OR ab:(Afghanistan* OR “Burkina Faso” OR Burundi OR “Central African Republic” OR Chad* OR Congo OR Eritrea OR Ethiopia OR Gambia OR Guinea OR “Guinea-Bissau” OR Haiti OR “Democratic People's Republic of Korea” OR Liberia OR Madagascar OR Malawi OR Mali OR Mozambique OR Niger OR Rwanda OR “Sierra Leone” OR Somalia* OR Sudan OR Syria* OR Tajikistan OR Togo OR Uganda OR Yemen OR “North Korea”)) OR ("Comoros" OR "Micronesia" OR "American Samoa" OR "Serbia" OR "Tonga" OR "Madagascar" OR "Moldova" OR "Papua New Guinea" OR "Ukraine" OR "Vanuatu" OR "Albania" OR "Armenia" OR "Azerbaijan" OR "Bulgaria" OR "Georgia" OR "Fiji" OR "Kosovo" OR "Montenegro" OR "Bosnia and Herzegovina") OR ("Algeria" OR "Egypt" OR "Libya" OR "Libyan Arab Jamahiriya" OR "Libyan Arab Republic" OR "Morocco" OR "Tunisia" OR "Cameroon" OR "Central African Republic" OR "Chad" OR "Congo" OR "Equatorial Guinea" OR "Gabon" OR "Sao Tome and Principe" OR "Burundi" OR "Djibouti" OR "Eritrea" OR "Ethiopia" OR "Kenya" OR "Rwanda" OR "Somalia" OR "South Sudan" OR "Sudan" OR "Tanzania" OR "Uganda" OR "Angola" OR "Botswana" OR "Lesotho" OR "Malawi" OR "Mozambique" OR "Namibia" OR "South Africa" OR "Zambia" OR "Zimbabwe" OR "Benin" OR "Burkina Faso" OR "Cote d'Ivoire" OR "Gambia" OR "Ghana" OR "Guinea" OR "Guinea-Bissau" OR "Liberia" OR "Mali" OR "Mauritania" OR "Niger" OR "Nigeria" OR "Senegal" OR "Sierra Leone" OR "Togo" OR "Cuba" OR "Dominica" OR "Dominican Republic" OR "Grenada" OR "Haiti" OR "Jamaica" OR "Saint Lucia" OR "Saint Vincent and the Grenadines" OR "Belize" OR "Costa Rica" OR "El Salvador" OR "Guatemala" OR "Honduras" OR "Nicaragua" OR "Mexico" OR "Argentina" OR "Brazil" OR "Colombia" OR "Ecuador" OR "Guyana" OR "Paraguay" OR "Turkmenistan" OR "Peru" OR "Suriname" OR "Venezuela" OR "Kazakhstan" OR "Tajikistan" OR "Kyrgyzstan" OR "Uzbekistan" OR "Russia" OR "Cambodia" OR "Lebanon" OR "Indonesia" OR "Malaysia" OR "Myanmar" OR "Philippines" OR "Thailand" OR "Timor-Leste" OR "Vietnam" OR "Bangladesh" OR "India" OR "Afghanistan" OR "Iran" OR "Iraq" OR "Jordan" OR "Syria" OR "Syrian Arab Republic" OR "Turkey" OR "Yemen" OR "Nepal" OR "Pakistan" OR "Sri Lanka" OR "China" OR "Democratic People's Republic of Korea" OR "Republic of North Macedonia" OR "Mongolia")](https://www-cabdirect-org.ezproxy.aub.edu.lb/cabdirect/search/?lq=edbf8116-b9f2-4fca-b6c1-ea0c02015e53&sort=Relevance) Refinements: | 21 Jul 2021 14:05 | | 8,337,654 | |
| [((title:((care OR service* OR health* OR wellbeing OR well-being)) OR ab:((care OR service* OR health* OR wellbeing OR well-being))) AND ("adolescents" OR "children" OR "infants")) OR (title:(wom$n OR child OR children OR infant* OR mother* OR maternal OR newborn* OR "new born*" OR neonat* OR teen* OR adolescen* OR youth OR young OR youngster) OR ab:(wom$n OR child OR children OR infant* OR mother* OR maternal OR newborn* OR "new born*" OR neonat* OR teen* OR adolescen* OR youth OR young OR youngster)) OR (title:(((gender-based OR domestic OR intimate-partner) near/1 violence)) OR ab:(((gender-based OR domestic OR intimate-partner) near/1 violence))) OR (title:((sexual-reproductive-health)) OR ab:((sexual-reproductive-health))) OR (title:((family-planning OR contraception*)) OR ab:((family-planning OR contraception*))) OR (title:((HIV near/2 (testing OR service* OR counseling OR counselling))) OR ab:((HIV near/2 (testing OR service* OR counseling OR counselling)))) OR (title:(((prenatal OR postnatal OR postpartum OR antenatal OR intrapartum OR abortion OR post-abortion OR delivery OR deliveries) near/2 (care OR service*))) OR ab:(((prenatal OR postnatal OR postpartum OR antenatal OR intrapartum OR abortion OR post-abortion OR delivery OR deliveries) near/2 (care OR service*)))) OR (title:(((thirteen OR fourteen OR fifteen OR sixteen OR seventeen OR eighteen OR "13" OR "14" OR "15" OR "16" OR "17" OR "18") near/1 (years-old OR yo OR "y.o."))) OR ab:(((thirteen OR fourteen OR fifteen OR sixteen OR seventeen OR eighteen OR "13" OR "14" OR "15" OR "16" OR "17" OR "18") near/1 (years-old OR yo OR "y.o.")))) OR ("women" OR "women's health" OR "child health" OR "reproductive health" OR "sexual health")](https://www-cabdirect-org.ezproxy.aub.edu.lb/cabdirect/search/?q=((title:((care%20OR%20service*%20OR%20health*%20OR%20wellbeing%20OR%20well-being))%20OR%20ab:((care%20OR%20service*%20OR%20health*%20OR%20wellbeing%20OR%20well-being)))%20AND%20(%22adolescents%22%20OR%20%22children%22%20OR%20%22infants%22))%20OR%20(title:(wom$n%20OR%20child%20OR%20children%20OR%20infant*%20OR%20mother*%20OR%20maternal%20OR%20newborn*%20OR%20%22new%20born*%22%20OR%20neonat*%20OR%20teen*%20OR%20adolescen*%20OR%20youth%20OR%20young%20OR%20youngster)%20OR%20ab:(wom$n%20OR%20child%20OR%20children%20OR%20infant*%20OR%20mother*%20OR%20maternal%20OR%20newborn*%20OR%20%22new%20born*%22%20OR%20neonat*%20OR%20teen*%20OR%20adolescen*%20OR%20youth%20OR%20young%20OR%20youngster))%20OR%20(title:(((gender-based%20OR%20domestic%20OR%20intimate-partner)%20near/1%20violence))%20OR%20ab:(((gender-based%20OR%20domestic%20OR%20intimate-partner)%20near/1%20violence)))%20OR%20(title:((sexual-reproductive-health))%20OR%20ab:((sexual-reproductive-health)))%20OR%20(title:((family-planning%20OR%20contraception*))%20OR%20ab:((family-planning%20OR%20contraception*)))%20OR%20(title:((HIV%20near/2%20(testing%20OR%20service*%20OR%20counseling%20OR%20counselling)))%20OR%20ab:((HIV%20near/2%20(testing%20OR%20service*%20OR%20counseling%20OR%20counselling))))%20OR%20(title:(((prenatal%20OR%20postnatal%20OR%20postpartum%20OR%20antenatal%20OR%20intrapartum%20OR%20abortion%20OR%20post-abortion%20OR%20delivery%20OR%20deliveries)%20near/2%20(care%20OR%20service*)))%20OR%20ab:(((prenatal%20OR%20postnatal%20OR%20postpartum%20OR%20antenatal%20OR%20intrapartum%20OR%20abortion%20OR%20post-abortion%20OR%20delivery%20OR%20deliveries)%20near/2%20(care%20OR%20service*))))%20OR%20(title:(((thirteen%20OR%20fourteen%20OR%20fifteen%20OR%20sixteen%20OR%20seventeen%20OR%20eighteen%20OR%20%2213%22%20OR%20%2214%22%20OR%20%2215%22%20OR%20%2216%22%20OR%20%2217%22%20OR%20%2218%22)%20near/1%20(years-old%20OR%20yo%20OR%20%22y.o.%22)))%20OR%20ab:(((thirteen%20OR%20fourteen%20OR%20fifteen%20OR%20sixteen%20OR%20seventeen%20OR%20eight) Refinements: | 21 Jul 2021 12:57 | | 651,736 | |
| [(title:(financial near/2 coverage) OR ab:(financial near/2 coverage)) OR (title:(health* near/1 equity-fund*) OR ab:(health* near/1 equity-fund*)) OR (title:(performance-based near/2 financing) OR ab:(performance-based near/2 financing)) OR (title:(government* near/2 fund*) OR ab:(government* near/2 fund*)) OR (title:(health* near/1 benefit-package*) OR ab:(health* near/1 benefit-package*)) OR (title:("official development assistance" OR ODA) OR ab:("official development assistance" OR ODA)) OR (title:((fee OR fees) near/2 (exemption OR reduction OR removal OR reducing) OR ab:((fee OR fees) near/2 (exemption OR reduction OR removal OR reducing)) OR (title:(donor* near/2 (funding OR support OR pool* OR contribution*)) OR ab:(donor* near/2 (funding OR support OR pool* OR contribution*))) OR (title:(out-of-pocket OR "out of pocket" OR OOP OR subsidy OR subsidies OR subsidized OR "conditional cash transfer" OR "conditional cash transfers" OR co-payment* OR premium* OR deductible* OR "cost sharing" OR cost-sharing OR capitation OR taxation OR tax OR taxes OR fee-exemption OR user-fee OR user-fees OR "user fees" OR "user fee" OR single-payer OR fee-for-service OR "fee for service" OR reimburs* OR voucher*) OR ab:(out-of-pocket OR "out of pocket" OR OOP OR subsidy OR subsidies OR subsidized OR "conditional cash transfer" OR "conditional cash transfers" OR co-payment* OR premium* OR deductible* OR "cost sharing" OR cost-sharing OR capitation OR taxation OR tax OR taxes OR fee-exemption OR user-fee OR user-fees OR "user fees" OR "user fee" OR single-payer OR fee-for-service OR "fee for service" OR reimburs* OR voucher*)) OR (title:((social OR community-based OR health*) near/2 (insurance OR coverage)) OR ab:((social OR community-based OR health*) near/2 (insurance OR coverage))) OR (title:(insurance near/2 (scheme* OR plan OR plans OR coverage OR mechanism* OR package* OR system*)) OR ab:(insurance near/2 (scheme* OR plan OR plans OR coverage OR mechanism* OR package* OR system*))) OR (title:(((targeted OR direct) near/2 payment*)) OR ab:(((targeted OR direct) near/2 payment*))) OR (title:(((prepaid OR pre-paid OR pre-payment*) near/2 (scheme* OR plan OR plans OR coverage OR insurance OR package*))) OR ab:(((prepaid OR pre-paid OR pre-payment*) near/2 (scheme* OR plan OR plans OR coverage OR insurance OR package*)))) OR (title:((financ* near/2 (mechanism OR strategy OR strategies OR scheme OR method OR methods OR protection OR system OR systems OR policy OR policies OR reform OR reforms OR plan* OR statement))) OR ab:((financ* near/2 (mechanism OR strategy OR strategies OR scheme OR method OR methods OR protection OR system OR systems OR policy OR policies OR reform OR reforms OR plan* OR statement)))) OR (title:((payment near/2 (mechanism OR strategy OR strategies OR scheme OR method* OR system OR polic* OR plan*))) OR ab:((payment near/2 (mechanism OR strategy OR strategies OR scheme OR method* OR system OR polic* OR plan*)))) OR ("insurance premiums" OR "health insurance")](https://www-cabdirect-org.ezproxy.aub.edu.lb/cabdirect/search/?lq=2f1828f9-7dfd-40f8-8eeb-0568e2a45b83&sort=Relevance) Refinements: | 21 Jul 2021 10:20 | | 5,444 | |

Global index medicus

Filtered to the years 2000- 2023

595

(tw:(“insurance benefits” OR “insurance coverage” OR “prepaid health plans” OR “children's health insurance program” OR “community-based health insurance” OR “single-payer system” OR “reimbursement” OR “Healthcare Financing” OR “Financial mechanism” OR “Financial strategy” OR “Financial strategies” OR “Financial scheme” OR “Financial method” OR “Financial protection” OR “Financial system” OR “Financial policy” OR “Financial policies” OR “Financial reform” OR “Financial plan” OR “Financial statement” OR “Payment mechanism” OR “Payment strategy” OR “Payment strategies” OR “Payment scheme” OR “Payment method” OR “Payment protection” OR “Payment system” OR “Payment policy” OR “Payment policies” OR “Payment reform” OR “Payment plan” OR “prepaid scheme” OR “prepaid plan” OR “prepaid plans” OR “prepaid coverage” OR “prepaid insurance” OR “prepaid package” OR “pre-paid scheme” OR “pre-paid plan” OR “pre-paid plans” OR “pre-paid coverage” OR “pre-paid insurance” OR “pre-paid package” OR “pre-payment scheme” OR “pre-payment plan” OR “pre-payment plans” OR “pre-payment coverage” OR “pre-payment insurance” OR “pre-payment package” OR “pre-payments scheme” OR “pre-payments plan” OR “pre-payments plans” OR “pre-payments coverage” OR “pre-payments insurance” OR “pre-payments package” OR “targeted payment” OR “direct payment” OR “insurance scheme” OR “insurance plan” OR “insurance plans” OR “insurance coverage” OR “insurance mechanism” OR “insurance package” OR “insurance system” OR “social insurance” OR “social coverage” OR “community-based insurance” OR “community-based coverage” OR “health insurance” OR “health coverage” OR “healthcare insurance” OR “healthcare coverage” OR “health-care insurance” OR “health-care coverage” OR out-of-pocket OR "out of pocket" OR OOP OR subsidy OR subsidies OR subsidized OR "conditional cash transfer" OR "conditional cash transfers" OR co-payment OR premium* OR deductible OR "cost sharing" OR cost-sharing OR capitation OR taxation OR tax OR taxes OR “fee exemption” OR “fee exempted” OR “exempted from fee” OR user-fee OR user-fees OR "user fees" OR "user fee" OR single-payer OR fee-for-service OR "fee for service" OR reimburs* OR voucher* OR “donor funding” OR “donor support” OR “donor pool” OR “donor contribution” OR “donor’s funding” OR “donor’s support” OR “donor’s pool” OR “donor’s contribution” OR “donors’ funding” OR “donors’ support” OR “donors’ pool” OR “donors’ contribution” OR “fees exemption” OR “exempted from fees” OR “reduction of fee” OR “fee reduction” OR “fees reduction” OR “fee removal” OR “fees removal” OR “removal of fees” OR “reducing fee” OR "official development assistance" OR “ODA” OR health-benefit-package OR healthcare-benefit-package OR “health-care benefit-package” OR “government fund” OR “governmental fund” OR “performance-based-financing” OR “health-equity-fund” OR “healthcare-equity-fund” OR “health-care equity-fund” OR “financial coverage” ))

| No. | Query | Results | Date |
| --- | --- | --- | --- |
| #46 | #45 AND [1-1-2000]/sd | 7937 | 21-Jul-21 |
| #45 | #21 AND #31 AND #39 AND #44 | 8335 | 21-Jul-21 |
| #44 | #40 OR #41 OR #42 OR #43 | 1342977 | 21-Jul-21 |
| #43 | (social NEXT/1 (exclusion OR inclusion)):ti,ab | 3927 | 21-Jul-21 |
| #42 | equit*:ti,ab OR inequi*:ti,ab OR fair*:ti,ab OR disparit*:ti,ab OR disadvantage*:ti,ab OR inequalit*:ti,ab | 376755 | 21-Jul-21 |
| #41 | ((poverty OR 'low income' OR socioeconomic* OR 'socio economic*' OR social OR ethnic$ OR race OR racial OR religio$ OR cultur$ OR minorit$ OR refugee OR indigenous OR aboriginal OR sex OR gender* OR urban OR rural OR 'inner city' OR slum) NEAR/2 (difference$ OR factor$ OR discriminat$)):ti,ab | 209613 | 21-Jul-21 |
| #40 | 'health care access'/exp OR 'health care disparity'/de OR 'disabled person'/exp OR 'educational status'/exp OR 'socioeconomics'/exp OR 'minority group'/de OR 'ethnic group'/exp OR 'urban health'/de OR 'urban population'/de OR 'sex factor'/exp OR 'geriatrics'/exp OR 'homosexuality'/exp | 871426 | 21-Jul-21 |
| #39 | #32 OR #33 OR #34 OR #35 OR #36 OR #37 OR #38 | 2123482 | 21-Jul-21 |
| #38 | ((developing OR 'less developed' OR 'lesser developed' OR 'least developed' OR 'under developed' OR underdeveloped OR 'under developed' OR underserved OR 'under served' OR deprived OR poor* OR transition*) NEAR/2 (countr* OR nation$ OR population$ OR world OR econom* OR communit* OR state$ OR people OR cities OR city)):ti,ab | 158763 | 21-Jul-21 |
| #37 | 'developing country'/de | 97479 | 21-Jul-21 |
| #36 | ((low OR middle) NEAR/2 countr*):ti,ab | 41004 | 21-Jul-21 |
| #35 | albania*:ti,ab OR 'american samoa':ti,ab OR argentin*:ti,ab OR armenia*:ti,ab OR azerbaijan:ti,ab OR belarus:ti,ab OR belize:ti,ab OR 'bosnia and herzegovina':ti,ab OR botswana:ti,ab OR brazil:ti,ab OR bulgaria*:ti,ab OR china:ti,ab OR colombia*:ti,ab OR 'costa rica':ti,ab OR cuba:ti,ab OR dominica:ti,ab OR 'dominican republic':ti,ab OR ecuador:ti,ab OR 'equatorial guinea':ti,ab OR fiji:ti,ab OR gabon:ti,ab OR georgia:ti,ab OR grenada:ti,ab OR guatemala:ti,ab OR guyana:ti,ab OR indonesia*:ti,ab OR iran*:ti,ab OR iraq*:ti,ab OR jamaica*:ti,ab OR jordan*:ti,ab OR kazakhstan:ti,ab OR kosovo:ti,ab OR leban*:ti,ab OR libya*:ti,ab OR malaysia*:ti,ab OR maldives:ti,ab OR 'marshall islands':ti,ab OR mexico:ti,ab OR montenegro:ti,ab OR namibia*:ti,ab OR 'north macedonia':ti,ab OR paraguay:ti,ab OR peru:ti,ab OR russia*:ti,ab OR samoa:ti,ab OR serbia:ti,ab OR 'south africa':ti,ab OR 'saint lucia':ti,ab OR 'st. lucia':ti,ab OR 'saint vincent and the grenadines':ti,ab OR 'st. vincent and the grenadines':ti,ab OR suriname:ti,ab OR thailand:ti,ab OR tonga:ti,ab OR turkey:ti,ab OR turkmenistan:ti,ab OR tuvalu:ti,ab OR venezuela:ti,ab | 927911 | 21-Jul-21 |
| #34 | algeria*:ti,ab OR angola:ti,ab OR bangladesh*:ti,ab OR benin:ti,ab OR bhutan:ti,ab OR bolivia*:ti,ab OR 'cabo verde':ti,ab OR cambodia:ti,ab OR cameroon:ti,ab OR comoros:ti,ab OR congo:ti,ab OR 'côte divoire':ti,ab OR 'ivory coast':ti,ab OR djibouti*:ti,ab OR egypt*:ti,ab OR 'el salvador':ti,ab OR eswatini:ti,ab OR ghana:ti,ab OR honduras:ti,ab OR india:ti,ab OR kenya*:ti,ab OR kiribati:ti,ab OR 'kyrgyz republic':ti,ab OR 'lao pdr':ti,ab OR lesotho:ti,ab OR mauritania:ti,ab OR micronesia:ti,ab OR moldova:ti,ab OR mongolia:ti,ab OR morocco*:ti,ab OR myanmar:ti,ab OR nepal*:ti,ab OR nicaragua:ti,ab OR nigeria$:ti,ab OR pakistan*:ti,ab OR 'papua new guinea':ti,ab OR philippin*:ti,ab OR 'são tomé and principe':ti,ab OR senegal:ti,ab OR 'solomon islands':ti,ab OR 'sri lanka':ti,ab OR tanzania*:ti,ab OR 'timor-leste':ti,ab OR tunisi*:ti,ab OR ukrain*:ti,ab OR uzbekistan:ti,ab OR vanuatu:ti,ab OR vietnam*:ti,ab OR 'west bank':ti,ab OR g$az$a:ti,ab OR zambia*:ti,ab OR zimbabwe:ti,ab | 500355 | 21-Jul-21 |
| #33 | afghanistan*:ti,ab OR 'burkina faso':ti,ab OR burundi:ti,ab OR 'central african republic':ti,ab OR chad*:ti,ab OR congo:ti,ab OR eritrea:ti,ab OR ethiopia:ti,ab OR gambia:ti,ab OR guinea:ti,ab OR 'guinea-bissau':ti,ab OR haiti:ti,ab OR liberia:ti,ab OR madagascar:ti,ab OR malawi:ti,ab OR mali:ti,ab OR mozambique:ti,ab OR niger:ti,ab OR rwanda:ti,ab OR 'sierra leone':ti,ab OR somalia*:ti,ab OR sudan:ti,ab OR 'syrian arab republic':ti,ab OR syria*:ti,ab OR tajikistan:ti,ab OR togo:ti,ab OR uganda:ti,ab OR yemen:ti,ab OR 'north korea':ti,ab | 273655 | 21-Jul-21 |
| #32 | 'algeria'/de OR 'egypt'/de OR 'libyan arab jamahiriya'/de OR 'morocco'/de OR 'tunisia'/de OR 'cameroon'/de OR 'central african republic'/de OR 'chad'/de OR 'congo'/de OR 'equatorial guinea'/de OR 'gabon'/de OR 'sao tome and principe'/de OR 'burundi'/de OR 'djibouti'/de OR 'eritrea'/de OR 'ethiopia'/de OR 'kenya'/de OR 'rwanda'/de OR 'somalia'/de OR 'south sudan'/de OR 'sudan'/de OR 'tanzania'/de OR 'uganda'/de OR 'angola'/de OR 'botswana'/de OR 'eswatini'/de OR 'lesotho'/de OR 'malawi'/de OR 'mozambique'/de OR 'namibia'/de OR 'south africa'/de OR 'zambia'/de OR 'zimbabwe'/de OR 'benin'/de OR 'burkina faso'/de OR 'cape verde'/de OR 'cote d`ivoire'/de OR 'gambia'/de OR 'ghana'/de OR 'guinea'/de OR 'guinea bissau'/de OR 'liberia'/de OR 'mali'/de OR 'mauritania'/de OR 'niger'/de OR 'nigeria'/de OR 'senegal'/de OR 'sierra leone'/de OR 'togo'/de OR 'cuba'/de OR 'dominica'/de OR 'dominican republic'/de OR 'grenada'/de OR 'haiti'/de OR 'jamaica'/de OR 'saint lucia'/de OR 'saint vincent and the grenadines'/de OR 'belize'/de OR 'costa rica'/de OR 'el salvador'/de OR 'guatemala'/de OR 'honduras'/de OR 'nicaragua'/de OR 'mexico'/de OR 'argentina'/de OR 'bolivia'/de OR 'brazil'/de OR 'colombia'/de OR 'ecuador'/de OR 'guyana'/de OR 'paraguay'/de OR 'peru'/de OR 'suriname'/de OR 'venezuela'/de OR 'kazakhstan'/de OR 'kyrgyzstan'/de OR 'tajikistan'/de OR 'turkmenistan'/de OR 'uzbekistan'/de OR 'russian federation'/de OR 'cambodia'/de OR 'indonesia'/de OR 'malaysia'/de OR 'myanmar'/de OR 'philippines'/de OR 'thailand'/de OR 'timor leste'/de OR 'viet nam'/de OR 'bangladesh'/de OR 'bhutan'/de OR 'india'/de OR 'afghanistan'/de OR 'iran'/de OR 'iraq'/de OR 'jordan'/de OR 'lebanon'/de OR 'syrian arab republic'/de OR 'turkey (republic)'/de OR 'yemen'/de OR 'nepal'/de OR 'pakistan'/de OR 'sri lanka'/de OR 'china'/de OR 'north korea'/de OR 'mongolia'/de OR 'republic of north macedonia'/de OR 'comoros'/de OR 'federated states of micronesia'/de OR 'american samoa'/de OR 'serbia'/de OR 'tonga'/de OR 'madagascar'/de OR 'moldova'/de OR 'papua new guinea'/de OR 'ukraine'/de OR 'vanuatu'/de OR 'albania'/de OR 'armenia'/de OR 'azerbaijan'/de OR 'belarus'/de OR 'bulgaria'/de OR 'fiji'/de OR 'georgia'/de OR 'georgia (republic)'/de OR 'kosovo'/de OR 'montenegro (republic)'/de OR 'bosnia and herzegovina'/de | 1353837 | 21-Jul-21 |
| #31 | #22 OR #23 OR #24 OR #25 OR #26 OR #27 OR #28 OR #29 OR #30 | 12407533 | 21-Jul-21 |
| #30 | ('adolescent'/exp OR 'child'/exp OR 'infant'/exp) AND (care:ti,ab OR service$:ti,ab OR health*:ti,ab OR wellbeing:ti,ab OR 'well being':ti,ab) | 859365 | 21-Jul-21 |
| #29 | (('gender based' OR domestic OR 'intimate partner') NEXT/1 violence):ti,ab | 17148 | 21-Jul-21 |
| #28 | 'sexual reproductive health':ti,ab | 278 | 21-Jul-21 |
| #27 | 'family planning':ti,ab OR contraception$:ti,ab | 52757 | 21-Jul-21 |
| #26 | (hiv NEAR/2 (testing OR service$ OR counsel$ing)):ti,ab | 25261 | 21-Jul-21 |
| #25 | ((prenatal OR postnatal OR postpartum OR antenatal OR intrapartum OR abortion OR 'post abortion' OR delivery OR deliveries) NEAR/2 (care OR service$)):ti,ab | 83621 | 21-Jul-21 |
| #24 | ((thirteen OR fourteen OR fifteen OR sixteen OR seventeen OR eighteen OR '13' OR '14' OR '15' OR '16' OR '17' OR '18') NEXT/1 ('years old' OR yo OR 'y.o.')):ti,ab | 48233 | 21-Jul-21 |
| #23 | wom$n:ti,ab OR child:ti,ab OR children:ti,ab OR infant$:ti,ab OR mother$:ti,ab OR maternal:ti,ab OR newborn$:ti,ab OR neonat*:ti,ab OR teen*:ti,ab OR adolescen*:ti,ab OR youth:ti,ab OR young:ti,ab OR youngster:ti,ab OR 'new born*':ti,ab | 4731809 | 21-Jul-21 |
| #22 | 'women`s health'/de OR 'female'/de OR 'female by marital status'/exp OR 'girl'/de OR 'adolescent health'/exp OR 'child health'/de OR 'reproductive health'/de OR 'sexual health'/exp OR 'child health care'/exp OR 'family planning'/de | 10469318 | 21-Jul-21 |
| #21 | #1 OR #2 OR #3 OR #4 OR #5 OR #6 OR #7 OR #8 OR #9 OR #10 OR #11 OR #12 OR #13 OR #14 OR #15 OR #16 OR #17 OR #18 OR #19 OR #20 | 463150 | 21-Jul-21 |
| #20 | (financial NEAR/2 coverage):ti,ab | 357 | 21-Jul-21 |
| #19 | ((health NEXT/1 'equity fund*'):ti,ab) OR ((healthcare NEXT/1 'equity fund*'):ti,ab) OR (('health care' NEXT/1 'equity fund*'):ti,ab) | 34 | 21-Jul-21 |
| #18 | ('performance based' NEAR/2 financing):ti,ab | 192 | 21-Jul-21 |
| #17 | (government* NEAR/2 fund*):ti,ab | 3792 | 21-Jul-21 |
| #16 | ((health NEXT/1 'benefit package'):ti,ab) OR ((healthcare NEXT/1 'benefit package'):ti,ab) OR (('health care' NEXT/1 'benefit package'):ti,ab) | 37 | 21-Jul-21 |
| #15 | 'official development assistance':ti,ab OR oda:ti,ab | 1165 | 21-Jul-21 |
| #14 | ((fee OR fees) NEAR/2 (exemption OR reduction OR removal OR reducing)):ti,ab | 269 | 21-Jul-21 |
| #13 | (donor$ NEAR/2 (funding OR support OR pool$ OR contribution$)):ti,ab | 6629 | 21-Jul-21 |
| #12 | 'co payment':ti,ab OR 'co payments':ti,ab | 1275 | 21-Jul-21 |
| #11 | 'conditional cash transfer':ti,ab | 303 | 21-Jul-21 |
| #10 | 'out of pocket':ti,ab OR oop:ti,ab OR subsidy:ti,ab OR subsidies:ti,ab OR subsidized:ti,ab | 17447 | 21-Jul-21 |
| #9 | premium$:ti,ab OR deductible$:ti,ab OR 'cost sharing':ti,ab OR capitation:ti,ab OR taxation:ti,ab OR tax:ti,ab OR taxes:ti,ab OR 'fee exemption':ti,ab OR 'user fee':ti,ab OR 'user fees':ti,ab OR 'single payer':ti,ab OR 'fee for service':ti,ab OR reimburs*:ti,ab OR voucher$:ti,ab | 85935 | 21-Jul-21 |
| #8 | ((social OR 'community based' OR health*) NEAR/2 (insurance OR coverage)):ti,ab | 72792 | 21-Jul-21 |
| #7 | (insurance NEAR/2 (scheme$ OR plan OR plans OR coverage OR mechanism$ OR package$ OR system$)):ti,ab | 23873 | 21-Jul-21 |
| #6 | ((targeted OR direct) NEAR/2 payment$):ti,ab | 393 | 21-Jul-21 |
| #5 | ('pre-payment' NEAR/2 (mechanism$ OR strategy OR strategies OR scheme$ OR method OR methods OR protection OR system OR systems OR policy OR policies OR reform OR reforms OR plan* OR statement$)):ti,ab | 29 | 21-Jul-21 |
| #4 | ('pre paid' NEAR/2 (mechanism$ OR strategy OR strategies OR scheme$ OR method OR methods OR protection OR system OR systems OR policy OR policies OR reform OR reforms OR plan* OR statement$)):ti,ab | 25 | 21-Jul-21 |
| #3 | (prepaid NEAR/2 (mechanism$ OR strategy OR strategies OR scheme$ OR method OR methods OR protection OR system OR systems OR policy OR policies OR reform OR reforms OR plan* OR statement$)):ti,ab | 522 | 21-Jul-21 |
| #2 | (financ* NEAR/2 (mechanism$ OR strategy OR strategies OR scheme$ OR method OR methods OR protection OR system OR systems OR policy OR policies OR reform OR reforms OR plan* OR statement$)):ti,ab | 9212 | 21-Jul-21 |
| #1 | 'insurance'/exp OR 'health care financing'/de | 374820 | 21-Jul-21 |

**PubMEd**

| Search number | Query | Sort By | Filters | Search Details | Results | Time |
| --- | --- | --- | --- | --- | --- | --- |
| 36 | #35 AND 2000/01:2021/12 [crdt] | | | ("insurance benefits"[MeSH Terms:noexp] OR "insurance coverage"[MeSH Terms:noexp] OR "prepaid health plans"[MeSH Terms:noexp] OR "insurance, health"[MeSH Terms:noexp] OR "children s health insurance program"[MeSH Terms:noexp] OR "community based health insurance"[MeSH Terms:noexp] OR "single payer system"[MeSH Terms:noexp] OR "insurance, health, reimbursement"[MeSH Terms] OR "healthcare financing"[MeSH Terms:noexp] OR ("financial mechanism"[Title/Abstract] OR "financial strategy"[Title/Abstract] OR "financial strategies"[Title/Abstract] OR "financial scheme"[Title/Abstract] OR "financial protection"[Title/Abstract] OR "financial system"[Title/Abstract] OR "financial policy"[Title/Abstract] OR "financial policies"[Title/Abstract] OR "financial reform"[Title/Abstract] OR "financial plan*"[Title/Abstract] OR "financial statement"[Title/Abstract]) OR 4[UID] OR ("prepaid plan"[Title/Abstract] OR "prepaid plans"[Title/Abstract] OR "prepaid coverage"[Title/Abstract] OR "prepaid insurance"[Title/Abstract] OR "pre paid insurance"[Title/Abstract] OR "pre payment scheme"[Title/Abstract]) OR "direct payment"[Title/Abstract] OR ("insurance scheme"[Title/Abstract] OR "insurance plan"[Title/Abstract] OR "insurance plans"[Title/Abstract] OR "insurance coverage"[Title/Abstract] OR "insurance mechanism"[Title/Abstract] OR "insurance package"[Title/Abstract] OR "insurance system"[Title/Abstract]) OR ("social insurance"[Title/Abstract] OR "social coverage"[Title/Abstract] OR "community based insurance"[Title/Abstract] OR "community based coverage"[Title/Abstract] OR "health insurance"[Title/Abstract] OR "health coverage"[Title/Abstract] OR "healthcare insurance"[Title/Abstract] OR "healthcare coverage"[Title/Abstract] OR "health care insurance"[Title/Abstract] OR "health care coverage"[Title/Abstract]) OR ("out of pocket"[Title/Abstract] OR "out of pocket"[Title/Abstract] OR "oop"[Title/Abstract] OR "subsidy"[Title/Abstract] OR "subsidies"[Title/Abstract] OR "subsidized"[Title/Abstract] OR "conditional cash transfer"[Title/Abstract] OR "conditional cash transfers"[Title/Abstract] OR "co payment"[Title/Abstract] OR "premium*"[Title/Abstract] OR "deductible"[Title/Abstract] OR "cost sharing"[Title/Abstract] OR "cost sharing"[Title/Abstract] OR "capitation"[Title/Abstract] OR "taxation"[Title/Abstract] OR "tax"[Title/Abstract] OR "taxes"[Title/Abstract] OR "fee exemption"[Title/Abstract] OR "fee exempted"[Title/Abstract] OR "user fee"[Title/Abstract] OR "user fees"[Title/Abstract] OR "user fees"[Title/Abstract] OR "user fee"[Title/Abstract] OR "single payer"[Title/Abstract] OR "fee for service"[Title/Abstract] OR "fee for service"[Title/Abstract] OR "reimburs*"[Title/Abstract] OR "voucher*"[Title/Abstract]) OR ("donor funding"[Title/Abstract] OR "donor support"[Title/Abstract] OR "donor pool"[Title/Abstract] OR "donor contribution"[Title/Abstract] OR "donor s pool"[Title/Abstract] OR "donor s contribution"[Title/Abstract] OR "donors pool"[Title/Abstract]) OR ("fees exemption"[Title/Abstract] OR "fee reduction"[Title/Abstract] OR "fee removal"[Title/Abstract] OR "fees removal"[Title/Abstract] OR "official development assistance"[Title/Abstract] OR "oda"[Title/Abstract] OR "health benefit package"[Title/Abstract] OR "healthcare benefit package"[Title/Abstract] OR "performance based financing"[Title/Abstract] OR "government fund"[Title/Abstract] OR "health equity fund"[Title/Abstract] OR "financial coverage"[Title/Abstract])) AND ("women"[MeSH Terms:noexp] OR "adolescent health"[MeSH Terms:noexp] OR "child health"[MeSH Terms:noexp] OR "infant health"[MeSH Terms:noexp] OR "reproductive health"[MeSH Terms:noexp] OR "sexual health"[MeSH Terms:noexp] OR "child health services"[MeSH Terms:noexp] OR "family planning services"[MeSH Terms:noexp] OR ("women"[Title/Abstract] OR "woman"[Title/Abstract] OR "child"[Title/Abstract] OR "children"[Title/Abstract] OR "infant"[Title/Abstract] OR "mother"[Title/Abstract] OR "maternal"[Title/Abstract] OR "neonat*"[Title/Abstract] OR "teen*"[Title/Abstract] OR "adolescen*"[Title/Abstract] OR "youth"[Title/Abstract] OR "young"[Title/Abstract] OR "youngster"[Title/Abstract] OR "newborn"[Title/Abstract] OR "new born"[Title/Abstract]) OR ("thirteen years old"[Title/Abstract] OR "fourteen years old"[Title/Abstract] OR "fifteen years old"[Title/Abstract] OR "sixteen years old"[Title/Abstract] OR "seventeen years old"[Title/Abstract] OR "eighteen years old"[Title/Abstract] OR "13 years old"[Title/Abstract] OR "14 years old"[Title/Abstract] OR "15 years old"[Title/Abstract] OR "16 years old"[Title/Abstract] OR "17 years old"[Title/Abstract] OR "18 years old"[Title/Abstract] OR "13 yo"[Title/Abstract] OR "15 yo"[Title/Abstract] OR "16 yo"[Title/Abstract] OR "17 yo"[Title/Abstract] OR "18 yo"[Title/Abstract] OR "13 y o"[Title/Abstract] OR "14 y o"[Title/Abstract] OR "15 y o"[Title/Abstract] OR "16 y o"[Title/Abstract] OR "17 y o"[Title/Abstract] OR "18 y o"[Title/Abstract]) OR ("prenatal care"[Title/Abstract] OR "postnatal care"[Title/Abstract] OR "postpartum care"[Title/Abstract] OR "antenatal care"[Title/Abstract] OR "intrapartum care"[Title/Abstract] OR "abortion care"[Title/Abstract] OR "post abortion care"[Title/Abstract] OR "delivery care"[Title/Abstract] OR "prenatal service"[Title/Abstract] OR "postnatal service"[Title/Abstract] OR "postpartum service"[Title/Abstract] OR "antenatal service"[Title/Abstract] OR "intrapartum service"[Title/Abstract] OR "abortion service"[Title/Abstract] OR "delivery service"[Title/Abstract]) OR ("hiv testing"[Title/Abstract] OR "hiv service"[Title/Abstract] OR "hiv counseling"[Title/Abstract] OR "hiv counselling"[Title/Abstract]) OR ("family planning"[Title/Abstract] OR "contraception"[Title/Abstract] OR "sexual reproductive health"[Title/Abstract] OR "gender based violence"[Title/Abstract] OR "domestic violence"[Title/Abstract] OR "intimate partner violence"[Title/Abstract]) OR (("adolescent"[MeSH Terms:noexp] OR "child"[MeSH Terms:noexp] OR "infant"[MeSH Terms:noexp]) AND ("care"[Title/Abstract] OR "service"[Title/Abstract] OR "health*"[Title/Abstract] OR "wellbeing"[Title/Abstract] OR "well being"[Title/Abstract]))) AND ("algeria"[MeSH Terms:noexp] OR "egypt"[MeSH Terms:noexp] OR "libya"[MeSH Terms:noexp] OR "morocco"[MeSH Terms:noexp] OR "tunisia"[MeSH Terms:noexp] OR "cameroon"[MeSH Terms:noexp] OR "central african republic"[MeSH Terms:noexp] OR "chad"[MeSH Terms:noexp] OR "congo"[MeSH Terms:noexp] OR "equatorial guinea"[MeSH Terms:noexp] OR "gabon"[MeSH Terms:noexp] OR "sao tome and principe"[MeSH Terms:noexp] OR "burundi"[MeSH Terms:noexp] OR "djibouti"[MeSH Terms:noexp] OR "eritrea"[MeSH Terms:noexp] OR "ethiopia"[MeSH Terms:noexp] OR "kenya"[MeSH Terms:noexp] OR "rwanda"[MeSH Terms:noexp] OR "somalia"[MeSH Terms:noexp] OR "south sudan"[MeSH Terms:noexp] OR "sudan"[MeSH Terms:noexp] OR "tanzania"[MeSH Terms:noexp] OR "uganda"[MeSH Terms:noexp] OR "angola"[MeSH Terms:noexp] OR "botswana"[MeSH Terms:noexp] OR "eswatini"[MeSH Terms:noexp] OR "lesotho"[MeSH Terms:noexp] OR "malawi"[MeSH Terms:noexp] OR "mozambique"[MeSH Terms:noexp] OR "namibia"[MeSH Terms:noexp] OR "south africa"[MeSH Terms:noexp] OR "zambia"[MeSH Terms:noexp] OR "zimbabwe"[MeSH Terms:noexp] OR "benin"[MeSH Terms:noexp] OR "burkina faso"[MeSH Terms:noexp] OR "cabo verde"[MeSH Terms:noexp] OR "cote d ivoire"[MeSH Terms:noexp] OR "gambia"[MeSH Terms:noexp] OR "ghana"[MeSH Terms:noexp] OR "guinea"[MeSH Terms:noexp] OR "guinea bissau"[MeSH Terms:noexp] OR "liberia"[MeSH Terms:noexp] OR "mali"[MeSH Terms:noexp] OR "mauritania"[MeSH Terms:noexp] OR "niger"[MeSH Terms:noexp] OR "nigeria"[MeSH Terms:noexp] OR "senegal"[MeSH Terms:noexp] OR "sierra leone"[MeSH Terms:noexp] OR "togo"[MeSH Terms:noexp] OR "cuba"[MeSH Terms:noexp] OR "dominica"[MeSH Terms:noexp] OR "dominican republic"[MeSH Terms:noexp] OR "grenada"[MeSH Terms:noexp] OR "haiti"[MeSH Terms:noexp] OR "jamaica"[MeSH Terms:noexp] OR "saint lucia"[MeSH Terms:noexp] OR "saint vincent and the grenadines"[MeSH Terms:noexp] OR "belize"[MeSH Terms:noexp] OR "costa rica"[MeSH Terms:noexp] OR "el salvador"[MeSH Terms:noexp] OR "guatemala"[MeSH Terms:noexp] OR "honduras"[MeSH Terms:noexp] OR "nicaragua"[MeSH Terms:noexp] OR "mexico"[MeSH Terms:noexp] OR "argentina"[MeSH Terms:noexp] OR "bolivia"[MeSH Terms:noexp] OR "brazil"[MeSH Terms:noexp] OR "colombia"[MeSH Terms:noexp] OR "ecuador"[MeSH Terms:noexp] OR "guyana"[MeSH Terms:noexp] OR "paraguay"[MeSH Terms:noexp] OR "peru"[MeSH Terms:noexp] OR "suriname"[MeSH Terms:noexp] OR "venezuela"[MeSH Terms:noexp] OR "kazakhstan"[MeSH Terms:noexp] OR "kyrgyzstan"[MeSH Terms:noexp] OR "tajikistan"[MeSH Terms:noexp] OR "turkmenistan"[MeSH Terms:noexp] OR "uzbekistan"[MeSH Terms:noexp] OR "russia"[MeSH Terms:noexp] OR "cambodia"[MeSH Terms:noexp] OR "indonesia"[MeSH Terms:noexp] OR "malaysia"[MeSH Terms:noexp] OR "myanmar"[MeSH Terms:noexp] OR "philippines"[MeSH Terms:noexp] OR "thailand"[MeSH Terms:noexp] OR "timor leste"[MeSH Terms:noexp] OR "vietnam"[MeSH Terms:noexp] OR "bangladesh"[MeSH Terms:noexp] OR "bhutan"[MeSH Terms:noexp] OR "india"[MeSH Terms:noexp] OR "afghanistan"[MeSH Terms:noexp] OR "iran"[MeSH Terms:noexp] OR "iraq"[MeSH Terms:noexp] OR "jordan"[MeSH Terms:noexp] OR "lebanon"[MeSH Terms:noexp] OR "syria"[MeSH Terms:noexp] OR "turkey"[MeSH Terms:noexp] OR "yemen"[MeSH Terms:noexp] OR "nepal"[MeSH Terms:noexp] OR "pakistan"[MeSH Terms:noexp] OR "sri lanka"[MeSH Terms:noexp] OR "china"[MeSH Terms:noexp] OR "democratic people s republic of korea"[MeSH Terms:noexp] OR "mongolia"[MeSH Terms:noexp] OR "republic of north macedonia"[MeSH Terms:noexp] OR "comoros"[MeSH Terms:noexp] OR "micronesia"[MeSH Terms:noexp] OR "american samoa"[MeSH Terms:noexp] OR "serbia"[MeSH Terms:noexp] OR "tonga"[MeSH Terms:noexp] OR "madagascar"[MeSH Terms:noexp] OR "moldova"[MeSH Terms:noexp] OR "papua new guinea"[MeSH Terms:noexp] OR "ukraine"[MeSH Terms:noexp] OR "vanuatu"[MeSH Terms:noexp] OR "albania"[MeSH Terms:noexp] OR "armenia"[MeSH Terms:noexp] OR "azerbaijan"[MeSH Terms:noexp] OR "republic of belarus"[MeSH Terms:noexp] OR "bulgaria"[MeSH Terms:noexp] OR "fiji"[MeSH Terms:noexp] OR "georgia"[MeSH Terms:noexp] OR "kosovo"[MeSH Terms:noexp] OR "montenegro"[MeSH Terms:noexp] OR "bosnia and herzegovina"[MeSH Terms:noexp] OR ("afghanistan*"[Title/Abstract] OR "burkina faso"[Title/Abstract] OR "burundi"[Title/Abstract] OR "central african republic"[Title/Abstract] OR "chad*"[Title/Abstract] OR "congo"[Title/Abstract] OR "eritrea"[Title/Abstract] OR "ethiopia"[Title/Abstract] OR "gambia"[Title/Abstract] OR "guinea"[Title/Abstract] OR "guinea bissau"[Title/Abstract] OR "haiti"[Title/Abstract] OR "democratic people s republic of korea"[Title/Abstract] OR "liberia"[Title/Abstract] OR "madagascar"[Title/Abstract] OR "malawi"[Title/Abstract] OR "mali"[Title/Abstract] OR "mozambique"[Title/Abstract] OR "niger"[Title/Abstract] OR "rwanda"[Title/Abstract] OR "sierra leone"[Title/Abstract] OR "somalia*"[Title/Abstract] OR "sudan"[Title/Abstract] OR "syrian arab republic"[Title/Abstract] OR "syria*"[Title/Abstract] OR "tajikistan"[Title/Abstract] OR "togo"[Title/Abstract] OR "uganda"[Title/Abstract] OR "yemen"[Title/Abstract] OR "north korea"[Title/Abstract] OR "algeria*"[Title/Abstract] OR "angola"[Title/Abstract] OR "bangladesh*"[Title/Abstract] OR "benin"[Title/Abstract] OR "bhutan"[Title/Abstract] OR "bolivia*"[Title/Abstract] OR "cabo verde"[Title/Abstract] OR "cambodia"[Title/Abstract] OR "cameroon"[Title/Abstract] OR "comoros"[Title/Abstract] OR "congo"[Title/Abstract] OR "cote d ivoire"[Title/Abstract] OR "ivory coast"[Title/Abstract] OR "djibouti*"[Title/Abstract] OR "egypt*"[Title/Abstract] OR "el salvador"[Title/Abstract] OR "eswatini"[Title/Abstract] OR "ghana"[Title/Abstract] OR "honduras"[Title/Abstract] OR "india"[Title/Abstract] OR "kenya*"[Title/Abstract] OR "kiribati"[Title/Abstract] OR "kyrgyz republic"[Title/Abstract] OR "lao pdr"[Title/Abstract] OR "lesotho"[Title/Abstract] OR "mauritania"[Title/Abstract] OR "micronesia"[Title/Abstract] OR "moldova"[Title/Abstract] OR "mongolia"[Title/Abstract] OR "morocco*"[Title/Abstract] OR "myanmar"[Title/Abstract] OR "nepal*"[Title/Abstract] OR "nicaragua"[Title/Abstract] OR "nigeria"[Title/Abstract] OR "pakistan*"[Title/Abstract] OR "papua new guinea"[Title/Abstract] OR "philippin*"[Title/Abstract] OR "sao tome and principe"[Title/Abstract] OR "senegal"[Title/Abstract] OR "solomon islands"[Title/Abstract] OR "sri lanka"[Title/Abstract] OR "tanzania*"[Title/Abstract] OR "timor leste"[Title/Abstract] OR "tunisi*"[Title/Abstract] OR "ukrain*"[Title/Abstract] OR "uzbekistan"[Title/Abstract] OR "vanuatu"[Title/Abstract] OR "vietnam*"[Title/Abstract] OR "west bank"[Title/Abstract] OR "ghaza"[Title/Abstract] OR "gazza"[Title/Abstract] OR "gaza"[Title/Abstract] OR "zambia*"[Title/Abstract] OR "zimbabwe"[Title/Abstract] OR "albania*"[Title/Abstract] OR "american samoa"[Title/Abstract] OR "argentin*"[Title/Abstract] OR "armenia*"[Title/Abstract] OR "azerbaijan"[Title/Abstract] OR "belarus"[Title/Abstract] OR "belize"[Title/Abstract] OR "bosnia and herzegovina"[Title/Abstract] OR "botswana"[Title/Abstract] OR "brazil"[Title/Abstract] OR "bulgaria*"[Title/Abstract] OR "china"[Title/Abstract] OR "colombia*"[Title/Abstract] OR "costa rica"[Title/Abstract] OR "cuba"[Title/Abstract] OR "dominica"[Title/Abstract] OR "dominican republic"[Title/Abstract] OR "ecuador"[Title/Abstract] OR "equatorial guinea"[Title/Abstract] OR "fiji"[Title/Abstract] OR "gabon"[Title/Abstract] OR "georgia"[Title/Abstract] OR "grenada"[Title/Abstract] OR "guatemala"[Title/Abstract] OR "guyana"[Title/Abstract] OR "indonesia*"[Title/Abstract] OR "iran*"[Title/Abstract] OR "iraq*"[Title/Abstract] OR "jamaica*"[Title/Abstract] OR "jordan*"[Title/Abstract] OR "kazakhstan"[Title/Abstract] OR "kosovo"[Title/Abstract] OR "leban*"[Title/Abstract] OR "libya*"[Title/Abstract] OR "malaysia*"[Title/Abstract] OR "maldives"[Title/Abstract] OR "marshall islands"[Title/Abstract] OR "mexico"[Title/Abstract] OR "montenegro"[Title/Abstract] OR "namibia*"[Title/Abstract] OR "north macedonia"[Title/Abstract] OR "paraguay"[Title/Abstract] OR "peru"[Title/Abstract] OR "russia*"[Title/Abstract] OR "samoa"[Title/Abstract] OR "serbia"[Title/Abstract] OR "south africa"[Title/Abstract] OR "saint lucia"[Title/Abstract] OR "st lucia"[Title/Abstract] OR "saint vincent and the grenadines"[Title/Abstract] OR "st vincent and the grenadines"[Title/Abstract] OR "suriname"[Title/Abstract] OR "thailand"[Title/Abstract] OR "tonga"[Title/Abstract] OR "turkey"[Title/Abstract] OR "turkmenistan"[Title/Abstract] OR "tuvalu"[Title/Abstract] OR "venezuela"[Title/Abstract]) OR ("low income country"[Title/Abstract] OR "middle income country"[Title/Abstract]) OR "developing countries"[MeSH Terms:noexp] OR ("developing countries"[Title/Abstract] OR "less developed countries"[Title/Abstract] OR "least developed countries"[Title/Abstract] OR "underdeveloped countries"[Title/Abstract] OR "under developed countries"[Title/Abstract] OR "underserved countries"[Title/Abstract] OR "deprived countries"[Title/Abstract] OR "poor countries"[Title/Abstract] OR "transition countries"[Title/Abstract] OR "transitional countries"[Title/Abstract] OR "developing nation"[Title/Abstract] OR "less developed nation"[Title/Abstract] OR "underdeveloped nation"[Title/Abstract] OR "poor nation"[Title/Abstract] OR "developing population"[Title/Abstract] OR "underdeveloped population"[Title/Abstract] OR "under served population"[Title/Abstract] OR "underserved population"[Title/Abstract] OR "deprived population"[Title/Abstract] OR "poor population"[Title/Abstract] OR "transition population"[Title/Abstract] OR "transitional population"[Title/Abstract] OR "developing world"[Title/Abstract] OR "less developed world"[Title/Abstract] OR "underdeveloped world"[Title/Abstract] OR "under developed world"[Title/Abstract] OR "poor world"[Title/Abstract] OR "transitional world"[Title/Abstract] OR "developing econom*"[Title/Abstract] OR "less developed econom*"[Title/Abstract] OR "least developed econom*"[Title/Abstract] OR "underdeveloped econom*"[Title/Abstract] OR "poor econom*"[Title/Abstract] OR "transition econom*"[Title/Abstract] OR "transitional econom*"[Title/Abstract] OR "developing communit*"[Title/Abstract] OR "less developed communit*"[Title/Abstract] OR "underdeveloped communit*"[Title/Abstract] OR "under developed communit*"[Title/Abstract] OR "under served communit*"[Title/Abstract] OR "underserved communit*"[Title/Abstract] OR "deprived communit*"[Title/Abstract] OR "poor communit*"[Title/Abstract] OR "transition communit*"[Title/Abstract] OR "transitional communit*"[Title/Abstract] OR "developing state"[Title/Abstract] OR "less developed state"[Title/Abstract] OR "underdeveloped state"[Title/Abstract] OR "underserved state"[Title/Abstract] OR "deprived state"[Title/Abstract] OR "poor state"[Title/Abstract] OR "transition state"[Title/Abstract] OR "transitional state"[Title/Abstract] OR "developing people"[Title/Abstract] OR "underserved people"[Title/Abstract] OR "deprived people"[Title/Abstract] OR "developing cities"[Title/Abstract] OR "less developed cities"[Title/Abstract] OR "underdeveloped cities"[Title/Abstract] OR "poor cities"[Title/Abstract] OR "developing city"[Title/Abstract] OR "less developed city"[Title/Abstract] OR "underdeveloped city"[Title/Abstract] OR "deprived city"[Title/Abstract] OR "poor city"[Title/Abstract])) AND ("ethnic difference"[Title/Abstract] OR "ethnic factor"[Title/Abstract] OR "ethnic discriminat*"[Title/Abstract] OR "ethnical difference"[Title/Abstract] OR "poverty difference*"[Title/Abstract] OR "poverty factor*"[Title/Abstract] OR "socioeconomic difference*"[Title/Abstract] OR "socio economic difference*"[Title/Abstract] OR "socioeconomic factor*"[Title/Abstract] OR "socio economic factor*"[Title/Abstract] OR "social difference*"[Title/Abstract] OR "social factor*"[Title/Abstract] OR "social discriminat*"[Title/Abstract] OR "race difference*"[Title/Abstract] OR "race factor*"[Title/Abstract] OR "race discriminat*"[Title/Abstract] OR "racial difference*"[Title/Abstract] OR "racial factor*"[Title/Abstract] OR "racial discriminat*"[Title/Abstract] OR "religion factor*"[Title/Abstract] OR "religious difference*"[Title/Abstract] OR "religious factor*"[Title/Abstract] OR "religious discriminat*"[Title/Abstract] OR "cultural difference*"[Title/Abstract] OR "cultural factor*"[Title/Abstract] OR "cultural discriminat*"[Title/Abstract] OR "culture difference*"[Title/Abstract] OR "culture factor*"[Title/Abstract] OR "minority difference*"[Title/Abstract] OR "minority factor*"[Title/Abstract] OR "minority discriminat*"[Title/Abstract] OR "indigenous difference*"[Title/Abstract] OR "indigenous factor*"[Title/Abstract] OR "sex difference*"[Title/Abstract] OR "sex factor*"[Title/Abstract] OR "sex discriminat*"[Title/Abstract] OR "gender difference*"[Title/Abstract] OR "gender factor*"[Title/Abstract] OR "gender discriminat*"[Title/Abstract] OR "urban difference*"[Title/Abstract] OR "urban factor*"[Title/Abstract] OR "rural difference*"[Title/Abstract] OR "rural factor*"[Title/Abstract] OR "gender based difference*"[Title/Abstract] OR "gender based factor*"[Title/Abstract] OR "gender based discriminat*"[Title/Abstract] OR ("sex factors"[MeSH Terms] OR "geriatrics"[MeSH Terms] OR "homosexuality"[MeSH Terms] OR "disabled persons"[MeSH Terms] OR "educational status"[MeSH Terms] OR "socioeconomic factors"[MeSH Terms] OR "minority groups"[MeSH Terms] OR "ethnic groups"[MeSH Terms] OR "urban health"[MeSH Terms] OR "urban population"[MeSH Terms] OR "health services accessibility"[MeSH Terms] OR "healthcare disparities"[MeSH Terms]) OR ("equit*"[Title/Abstract] OR "inequi*"[Title/Abstract] OR "fair*"[Title/Abstract] OR "disparit*"[Title/Abstract] OR "disadvantage*"[Title/Abstract] OR "inequality*"[Title/Abstract] OR "social inclusion"[Title/Abstract])) AND 2000/01/01:2021/12/31[Date - Create] | 3,107 | 17:24:48 |
| 35 | #13 AND #21 AND #27 AND #34 | | | (("insurance benefits"[MeSH Terms:noexp] OR "insurance coverage"[MeSH Terms:noexp] OR "prepaid health plans"[MeSH Terms:noexp] OR "insurance, health"[MeSH Terms:noexp] OR "children s health insurance program"[MeSH Terms:noexp] OR "community based health insurance"[MeSH Terms:noexp] OR "single payer system"[MeSH Terms:noexp] OR "insurance, health, reimbursement"[MeSH Terms] OR "healthcare financing"[MeSH Terms:noexp] OR ("financial mechanism"[Title/Abstract] OR "financial strategy"[Title/Abstract] OR "financial strategies"[Title/Abstract] OR "financial scheme"[Title/Abstract] OR "financial protection"[Title/Abstract] OR "financial system"[Title/Abstract] OR "financial policy"[Title/Abstract] OR "financial policies"[Title/Abstract] OR "financial reform"[Title/Abstract] OR "financial plan*"[Title/Abstract] OR "financial statement"[Title/Abstract]) OR 4[UID] OR ("prepaid plan"[Title/Abstract] OR "prepaid plans"[Title/Abstract] OR "prepaid coverage"[Title/Abstract] OR "prepaid insurance"[Title/Abstract] OR "pre paid insurance"[Title/Abstract] OR "pre payment scheme"[Title/Abstract]) OR "direct payment"[Title/Abstract] OR ("insurance scheme"[Title/Abstract] OR "insurance plan"[Title/Abstract] OR "insurance plans"[Title/Abstract] OR "insurance coverage"[Title/Abstract] OR "insurance mechanism"[Title/Abstract] OR "insurance package"[Title/Abstract] OR "insurance system"[Title/Abstract]) OR ("social insurance"[Title/Abstract] OR "social coverage"[Title/Abstract] OR "community based insurance"[Title/Abstract] OR "community based coverage"[Title/Abstract] OR "health insurance"[Title/Abstract] OR "health coverage"[Title/Abstract] OR "healthcare insurance"[Title/Abstract] OR "healthcare coverage"[Title/Abstract] OR "health care insurance"[Title/Abstract] OR "health care coverage"[Title/Abstract]) OR ("out of pocket"[Title/Abstract] OR "out of pocket"[Title/Abstract] OR "oop"[Title/Abstract] OR "subsidy"[Title/Abstract] OR "subsidies"[Title/Abstract] OR "subsidized"[Title/Abstract] OR "conditional cash transfer"[Title/Abstract] OR "conditional cash transfers"[Title/Abstract] OR "co payment"[Title/Abstract] OR "premium*"[Title/Abstract] OR "deductible"[Title/Abstract] OR "cost sharing"[Title/Abstract] OR "cost sharing"[Title/Abstract] OR "capitation"[Title/Abstract] OR "taxation"[Title/Abstract] OR "tax"[Title/Abstract] OR "taxes"[Title/Abstract] OR "fee exemption"[Title/Abstract] OR "fee exempted"[Title/Abstract] OR "user fee"[Title/Abstract] OR "user fees"[Title/Abstract] OR "user fees"[Title/Abstract] OR "user fee"[Title/Abstract] OR "single payer"[Title/Abstract] OR "fee for service"[Title/Abstract] OR "fee for service"[Title/Abstract] OR "reimburs*"[Title/Abstract] OR "voucher*"[Title/Abstract]) OR ("donor funding"[Title/Abstract] OR "donor support"[Title/Abstract] OR "donor pool"[Title/Abstract] OR "donor contribution"[Title/Abstract] OR "donor s pool"[Title/Abstract] OR "donor s contribution"[Title/Abstract] OR "donors pool"[Title/Abstract]) OR ("fees exemption"[Title/Abstract] OR "fee reduction"[Title/Abstract] OR "fee removal"[Title/Abstract] OR "fees removal"[Title/Abstract] OR "official development assistance"[Title/Abstract] OR "oda"[Title/Abstract] OR "health benefit package"[Title/Abstract] OR "healthcare benefit package"[Title/Abstract] OR "performance based financing"[Title/Abstract] OR "government fund"[Title/Abstract] OR "health equity fund"[Title/Abstract] OR "financial coverage"[Title/Abstract])) AND ("women"[MeSH Terms:noexp] OR "adolescent health"[MeSH Terms:noexp] OR "child health"[MeSH Terms:noexp] OR "infant health"[MeSH Terms:noexp] OR "reproductive health"[MeSH Terms:noexp] OR "sexual health"[MeSH Terms:noexp] OR "child health services"[MeSH Terms:noexp] OR "family planning services"[MeSH Terms:noexp] OR ("women"[Title/Abstract] OR "woman"[Title/Abstract] OR "child"[Title/Abstract] OR "children"[Title/Abstract] OR "infant"[Title/Abstract] OR "mother"[Title/Abstract] OR "maternal"[Title/Abstract] OR "neonat*"[Title/Abstract] OR "teen*"[Title/Abstract] OR "adolescen*"[Title/Abstract] OR "youth"[Title/Abstract] OR "young"[Title/Abstract] OR "youngster"[Title/Abstract] OR "newborn"[Title/Abstract] OR "new born"[Title/Abstract]) OR ("thirteen years old"[Title/Abstract] OR "fourteen years old"[Title/Abstract] OR "fifteen years old"[Title/Abstract] OR "sixteen years old"[Title/Abstract] OR "seventeen years old"[Title/Abstract] OR "eighteen years old"[Title/Abstract] OR "13 years old"[Title/Abstract] OR "14 years old"[Title/Abstract] OR "15 years old"[Title/Abstract] OR "16 years old"[Title/Abstract] OR "17 years old"[Title/Abstract] OR "18 years old"[Title/Abstract] OR "13 yo"[Title/Abstract] OR "15 yo"[Title/Abstract] OR "16 yo"[Title/Abstract] OR "17 yo"[Title/Abstract] OR "18 yo"[Title/Abstract] OR "13 y o"[Title/Abstract] OR "14 y o"[Title/Abstract] OR "15 y o"[Title/Abstract] OR "16 y o"[Title/Abstract] OR "17 y o"[Title/Abstract] OR "18 y o"[Title/Abstract]) OR ("prenatal care"[Title/Abstract] OR "postnatal care"[Title/Abstract] OR "postpartum care"[Title/Abstract] OR "antenatal care"[Title/Abstract] OR "intrapartum care"[Title/Abstract] OR "abortion care"[Title/Abstract] OR "post abortion care"[Title/Abstract] OR "delivery care"[Title/Abstract] OR "prenatal service"[Title/Abstract] OR "postnatal service"[Title/Abstract] OR "postpartum service"[Title/Abstract] OR "antenatal service"[Title/Abstract] OR "intrapartum service"[Title/Abstract] OR "abortion service"[Title/Abstract] OR "delivery service"[Title/Abstract]) OR ("hiv testing"[Title/Abstract] OR "hiv service"[Title/Abstract] OR "hiv counseling"[Title/Abstract] OR "hiv counselling"[Title/Abstract]) OR ("family planning"[Title/Abstract] OR "contraception"[Title/Abstract] OR "sexual reproductive health"[Title/Abstract] OR "gender based violence"[Title/Abstract] OR "domestic violence"[Title/Abstract] OR "intimate partner violence"[Title/Abstract]) OR (("adolescent"[MeSH Terms:noexp] OR "child"[MeSH Terms:noexp] OR "infant"[MeSH Terms:noexp]) AND ("care"[Title/Abstract] OR "service"[Title/Abstract] OR "health*"[Title/Abstract] OR "wellbeing"[Title/Abstract] OR "well being"[Title/Abstract]))) AND ("algeria"[MeSH Terms:noexp] OR "egypt"[MeSH Terms:noexp] OR "libya"[MeSH Terms:noexp] OR "morocco"[MeSH Terms:noexp] OR "tunisia"[MeSH Terms:noexp] OR "cameroon"[MeSH Terms:noexp] OR "central african republic"[MeSH Terms:noexp] OR "chad"[MeSH Terms:noexp] OR "congo"[MeSH Terms:noexp] OR "equatorial guinea"[MeSH Terms:noexp] OR "gabon"[MeSH Terms:noexp] OR "sao tome and principe"[MeSH Terms:noexp] OR "burundi"[MeSH Terms:noexp] OR "djibouti"[MeSH Terms:noexp] OR "eritrea"[MeSH Terms:noexp] OR "ethiopia"[MeSH Terms:noexp] OR "kenya"[MeSH Terms:noexp] OR "rwanda"[MeSH Terms:noexp] OR "somalia"[MeSH Terms:noexp] OR "south sudan"[MeSH Terms:noexp] OR "sudan"[MeSH Terms:noexp] OR "tanzania"[MeSH Terms:noexp] OR "uganda"[MeSH Terms:noexp] OR "angola"[MeSH Terms:noexp] OR "botswana"[MeSH Terms:noexp] OR "eswatini"[MeSH Terms:noexp] OR "lesotho"[MeSH Terms:noexp] OR "malawi"[MeSH Terms:noexp] OR "mozambique"[MeSH Terms:noexp] OR "namibia"[MeSH Terms:noexp] OR "south africa"[MeSH Terms:noexp] OR "zambia"[MeSH Terms:noexp] OR "zimbabwe"[MeSH Terms:noexp] OR "benin"[MeSH Terms:noexp] OR "burkina faso"[MeSH Terms:noexp] OR "cabo verde"[MeSH Terms:noexp] OR "cote d ivoire"[MeSH Terms:noexp] OR "gambia"[MeSH Terms:noexp] OR "ghana"[MeSH Terms:noexp] OR "guinea"[MeSH Terms:noexp] OR "guinea bissau"[MeSH Terms:noexp] OR "liberia"[MeSH Terms:noexp] OR "mali"[MeSH Terms:noexp] OR "mauritania"[MeSH Terms:noexp] OR "niger"[MeSH Terms:noexp] OR "nigeria"[MeSH Terms:noexp] OR "senegal"[MeSH Terms:noexp] OR "sierra leone"[MeSH Terms:noexp] OR "togo"[MeSH Terms:noexp] OR "cuba"[MeSH Terms:noexp] OR "dominica"[MeSH Terms:noexp] OR "dominican republic"[MeSH Terms:noexp] OR "grenada"[MeSH Terms:noexp] OR "haiti"[MeSH Terms:noexp] OR "jamaica"[MeSH Terms:noexp] OR "saint lucia"[MeSH Terms:noexp] OR "saint vincent and the grenadines"[MeSH Terms:noexp] OR "belize"[MeSH Terms:noexp] OR "costa rica"[MeSH Terms:noexp] OR "el salvador"[MeSH Terms:noexp] OR "guatemala"[MeSH Terms:noexp] OR "honduras"[MeSH Terms:noexp] OR "nicaragua"[MeSH Terms:noexp] OR "mexico"[MeSH Terms:noexp] OR "argentina"[MeSH Terms:noexp] OR "bolivia"[MeSH Terms:noexp] OR "brazil"[MeSH Terms:noexp] OR "colombia"[MeSH Terms:noexp] OR "ecuador"[MeSH Terms:noexp] OR "guyana"[MeSH Terms:noexp] OR "paraguay"[MeSH Terms:noexp] OR "peru"[MeSH Terms:noexp] OR "suriname"[MeSH Terms:noexp] OR "venezuela"[MeSH Terms:noexp] OR "kazakhstan"[MeSH Terms:noexp] OR "kyrgyzstan"[MeSH Terms:noexp] OR "tajikistan"[MeSH Terms:noexp] OR "turkmenistan"[MeSH Terms:noexp] OR "uzbekistan"[MeSH Terms:noexp] OR "russia"[MeSH Terms:noexp] OR "cambodia"[MeSH Terms:noexp] OR "indonesia"[MeSH Terms:noexp] OR "malaysia"[MeSH Terms:noexp] OR "myanmar"[MeSH Terms:noexp] OR "philippines"[MeSH Terms:noexp] OR "thailand"[MeSH Terms:noexp] OR "timor leste"[MeSH Terms:noexp] OR "vietnam"[MeSH Terms:noexp] OR "bangladesh"[MeSH Terms:noexp] OR "bhutan"[MeSH Terms:noexp] OR "india"[MeSH Terms:noexp] OR "afghanistan"[MeSH Terms:noexp] OR "iran"[MeSH Terms:noexp] OR "iraq"[MeSH Terms:noexp] OR "jordan"[MeSH Terms:noexp] OR "lebanon"[MeSH Terms:noexp] OR "syria"[MeSH Terms:noexp] OR "turkey"[MeSH Terms:noexp] OR "yemen"[MeSH Terms:noexp] OR "nepal"[MeSH Terms:noexp] OR "pakistan"[MeSH Terms:noexp] OR "sri lanka"[MeSH Terms:noexp] OR "china"[MeSH Terms:noexp] OR "democratic people s republic of korea"[MeSH Terms:noexp] OR "mongolia"[MeSH Terms:noexp] OR "republic of north macedonia"[MeSH Terms:noexp] OR "comoros"[MeSH Terms:noexp] OR "micronesia"[MeSH Terms:noexp] OR "american samoa"[MeSH Terms:noexp] OR "serbia"[MeSH Terms:noexp] OR "tonga"[MeSH Terms:noexp] OR "madagascar"[MeSH Terms:noexp] OR "moldova"[MeSH Terms:noexp] OR "papua new guinea"[MeSH Terms:noexp] OR "ukraine"[MeSH Terms:noexp] OR "vanuatu"[MeSH Terms:noexp] OR "albania"[MeSH Terms:noexp] OR "armenia"[MeSH Terms:noexp] OR "azerbaijan"[MeSH Terms:noexp] OR "republic of belarus"[MeSH Terms:noexp] OR "bulgaria"[MeSH Terms:noexp] OR "fiji"[MeSH Terms:noexp] OR "georgia"[MeSH Terms:noexp] OR "kosovo"[MeSH Terms:noexp] OR "montenegro"[MeSH Terms:noexp] OR "bosnia and herzegovina"[MeSH Terms:noexp] OR ("afghanistan*"[Title/Abstract] OR "burkina faso"[Title/Abstract] OR "burundi"[Title/Abstract] OR "central african republic"[Title/Abstract] OR "chad*"[Title/Abstract] OR "congo"[Title/Abstract] OR "eritrea"[Title/Abstract] OR "ethiopia"[Title/Abstract] OR "gambia"[Title/Abstract] OR "guinea"[Title/Abstract] OR "guinea bissau"[Title/Abstract] OR "haiti"[Title/Abstract] OR "democratic people s republic of korea"[Title/Abstract] OR "liberia"[Title/Abstract] OR "madagascar"[Title/Abstract] OR "malawi"[Title/Abstract] OR "mali"[Title/Abstract] OR "mozambique"[Title/Abstract] OR "niger"[Title/Abstract] OR "rwanda"[Title/Abstract] OR "sierra leone"[Title/Abstract] OR "somalia*"[Title/Abstract] OR "sudan"[Title/Abstract] OR "syrian arab republic"[Title/Abstract] OR "syria*"[Title/Abstract] OR "tajikistan"[Title/Abstract] OR "togo"[Title/Abstract] OR "uganda"[Title/Abstract] OR "yemen"[Title/Abstract] OR "north korea"[Title/Abstract] OR "algeria*"[Title/Abstract] OR "angola"[Title/Abstract] OR "bangladesh*"[Title/Abstract] OR "benin"[Title/Abstract] OR "bhutan"[Title/Abstract] OR "bolivia*"[Title/Abstract] OR "cabo verde"[Title/Abstract] OR "cambodia"[Title/Abstract] OR "cameroon"[Title/Abstract] OR "comoros"[Title/Abstract] OR "congo"[Title/Abstract] OR "cote d ivoire"[Title/Abstract] OR "ivory coast"[Title/Abstract] OR "djibouti*"[Title/Abstract] OR "egypt*"[Title/Abstract] OR "el salvador"[Title/Abstract] OR "eswatini"[Title/Abstract] OR "ghana"[Title/Abstract] OR "honduras"[Title/Abstract] OR "india"[Title/Abstract] OR "kenya*"[Title/Abstract] OR "kiribati"[Title/Abstract] OR "kyrgyz republic"[Title/Abstract] OR "lao pdr"[Title/Abstract] OR "lesotho"[Title/Abstract] OR "mauritania"[Title/Abstract] OR "micronesia"[Title/Abstract] OR "moldova"[Title/Abstract] OR "mongolia"[Title/Abstract] OR "morocco*"[Title/Abstract] OR "myanmar"[Title/Abstract] OR "nepal*"[Title/Abstract] OR "nicaragua"[Title/Abstract] OR "nigeria"[Title/Abstract] OR "pakistan*"[Title/Abstract] OR "papua new guinea"[Title/Abstract] OR "philippin*"[Title/Abstract] OR "sao tome and principe"[Title/Abstract] OR "senegal"[Title/Abstract] OR "solomon islands"[Title/Abstract] OR "sri lanka"[Title/Abstract] OR "tanzania*"[Title/Abstract] OR "timor leste"[Title/Abstract] OR "tunisi*"[Title/Abstract] OR "ukrain*"[Title/Abstract] OR "uzbekistan"[Title/Abstract] OR "vanuatu"[Title/Abstract] OR "vietnam*"[Title/Abstract] OR "west bank"[Title/Abstract] OR "ghaza"[Title/Abstract] OR "gazza"[Title/Abstract] OR "gaza"[Title/Abstract] OR "zambia*"[Title/Abstract] OR "zimbabwe"[Title/Abstract] OR "albania*"[Title/Abstract] OR "american samoa"[Title/Abstract] OR "argentin*"[Title/Abstract] OR "armenia*"[Title/Abstract] OR "azerbaijan"[Title/Abstract] OR "belarus"[Title/Abstract] OR "belize"[Title/Abstract] OR "bosnia and herzegovina"[Title/Abstract] OR "botswana"[Title/Abstract] OR "brazil"[Title/Abstract] OR "bulgaria*"[Title/Abstract] OR "china"[Title/Abstract] OR "colombia*"[Title/Abstract] OR "costa rica"[Title/Abstract] OR "cuba"[Title/Abstract] OR "dominica"[Title/Abstract] OR "dominican republic"[Title/Abstract] OR "ecuador"[Title/Abstract] OR "equatorial guinea"[Title/Abstract] OR "fiji"[Title/Abstract] OR "gabon"[Title/Abstract] OR "georgia"[Title/Abstract] OR "grenada"[Title/Abstract] OR "guatemala"[Title/Abstract] OR "guyana"[Title/Abstract] OR "indonesia*"[Title/Abstract] OR "iran*"[Title/Abstract] OR "iraq*"[Title/Abstract] OR "jamaica*"[Title/Abstract] OR "jordan*"[Title/Abstract] OR "kazakhstan"[Title/Abstract] OR "kosovo"[Title/Abstract] OR "leban*"[Title/Abstract] OR "libya*"[Title/Abstract] OR "malaysia*"[Title/Abstract] OR "maldives"[Title/Abstract] OR "marshall islands"[Title/Abstract] OR "mexico"[Title/Abstract] OR "montenegro"[Title/Abstract] OR "namibia*"[Title/Abstract] OR "north macedonia"[Title/Abstract] OR "paraguay"[Title/Abstract] OR "peru"[Title/Abstract] OR "russia*"[Title/Abstract] OR "samoa"[Title/Abstract] OR "serbia"[Title/Abstract] OR "south africa"[Title/Abstract] OR "saint lucia"[Title/Abstract] OR "st lucia"[Title/Abstract] OR "saint vincent and the grenadines"[Title/Abstract] OR "st vincent and the grenadines"[Title/Abstract] OR "suriname"[Title/Abstract] OR "thailand"[Title/Abstract] OR "tonga"[Title/Abstract] OR "turkey"[Title/Abstract] OR "turkmenistan"[Title/Abstract] OR "tuvalu"[Title/Abstract] OR "venezuela"[Title/Abstract]) OR ("low income country"[Title/Abstract] OR "middle income country"[Title/Abstract]) OR "developing countries"[MeSH Terms:noexp] OR ("developing countries"[Title/Abstract] OR "less developed countries"[Title/Abstract] OR "least developed countries"[Title/Abstract] OR "underdeveloped countries"[Title/Abstract] OR "under developed countries"[Title/Abstract] OR "underserved countries"[Title/Abstract] OR "deprived countries"[Title/Abstract] OR "poor countries"[Title/Abstract] OR "transition countries"[Title/Abstract] OR "transitional countries"[Title/Abstract] OR "developing nation"[Title/Abstract] OR "less developed nation"[Title/Abstract] OR "underdeveloped nation"[Title/Abstract] OR "poor nation"[Title/Abstract] OR "developing population"[Title/Abstract] OR "underdeveloped population"[Title/Abstract] OR "under served population"[Title/Abstract] OR "underserved population"[Title/Abstract] OR "deprived population"[Title/Abstract] OR "poor population"[Title/Abstract] OR "transition population"[Title/Abstract] OR "transitional population"[Title/Abstract] OR "developing world"[Title/Abstract] OR "less developed world"[Title/Abstract] OR "underdeveloped world"[Title/Abstract] OR "under developed world"[Title/Abstract] OR "poor world"[Title/Abstract] OR "transitional world"[Title/Abstract] OR "developing econom*"[Title/Abstract] OR "less developed econom*"[Title/Abstract] OR "least developed econom*"[Title/Abstract] OR "underdeveloped econom*"[Title/Abstract] OR "poor econom*"[Title/Abstract] OR "transition econom*"[Title/Abstract] OR "transitional econom*"[Title/Abstract] OR "developing communit*"[Title/Abstract] OR "less developed communit*"[Title/Abstract] OR "underdeveloped communit*"[Title/Abstract] OR "under developed communit*"[Title/Abstract] OR "under served communit*"[Title/Abstract] OR "underserved communit*"[Title/Abstract] OR "deprived communit*"[Title/Abstract] OR "poor communit*"[Title/Abstract] OR "transition communit*"[Title/Abstract] OR "transitional communit*"[Title/Abstract] OR "developing state"[Title/Abstract] OR "less developed state"[Title/Abstract] OR "underdeveloped state"[Title/Abstract] OR "underserved state"[Title/Abstract] OR "deprived state"[Title/Abstract] OR "poor state"[Title/Abstract] OR "transition state"[Title/Abstract] OR "transitional state"[Title/Abstract] OR "developing people"[Title/Abstract] OR "underserved people"[Title/Abstract] OR "deprived people"[Title/Abstract] OR "developing cities"[Title/Abstract] OR "less developed cities"[Title/Abstract] OR "underdeveloped cities"[Title/Abstract] OR "poor cities"[Title/Abstract] OR "developing city"[Title/Abstract] OR "less developed city"[Title/Abstract] OR "underdeveloped city"[Title/Abstract] OR "deprived city"[Title/Abstract] OR "poor city"[Title/Abstract])) AND ("ethnic difference"[Title/Abstract] OR "ethnic factor"[Title/Abstract] OR "ethnic discriminat*"[Title/Abstract] OR "ethnical difference"[Title/Abstract] OR "poverty difference*"[Title/Abstract] OR "poverty factor*"[Title/Abstract] OR "socioeconomic difference*"[Title/Abstract] OR "socio economic difference*"[Title/Abstract] OR "socioeconomic factor*"[Title/Abstract] OR "socio economic factor*"[Title/Abstract] OR "social difference*"[Title/Abstract] OR "social factor*"[Title/Abstract] OR "social discriminat*"[Title/Abstract] OR "race difference*"[Title/Abstract] OR "race factor*"[Title/Abstract] OR "race discriminat*"[Title/Abstract] OR "racial difference*"[Title/Abstract] OR "racial factor*"[Title/Abstract] OR "racial discriminat*"[Title/Abstract] OR "religion factor*"[Title/Abstract] OR "religious difference*"[Title/Abstract] OR "religious factor*"[Title/Abstract] OR "religious discriminat*"[Title/Abstract] OR "cultural difference*"[Title/Abstract] OR "cultural factor*"[Title/Abstract] OR "cultural discriminat*"[Title/Abstract] OR "culture difference*"[Title/Abstract] OR "culture factor*"[Title/Abstract] OR "minority difference*"[Title/Abstract] OR "minority factor*"[Title/Abstract] OR "minority discriminat*"[Title/Abstract] OR "indigenous difference*"[Title/Abstract] OR "indigenous factor*"[Title/Abstract] OR "sex difference*"[Title/Abstract] OR "sex factor*"[Title/Abstract] OR "sex discriminat*"[Title/Abstract] OR "gender difference*"[Title/Abstract] OR "gender factor*"[Title/Abstract] OR "gender discriminat*"[Title/Abstract] OR "urban difference*"[Title/Abstract] OR "urban factor*"[Title/Abstract] OR "rural difference*"[Title/Abstract] OR "rural factor*"[Title/Abstract] OR "gender based difference*"[Title/Abstract] OR "gender based factor*"[Title/Abstract] OR "gender based discriminat*"[Title/Abstract])) OR ("sex factors"[MeSH Terms] OR "geriatrics"[MeSH Terms] OR "homosexuality"[MeSH Terms] OR "disabled persons"[MeSH Terms] OR "educational status"[MeSH Terms] OR "socioeconomic factors"[MeSH Terms] OR "minority groups"[MeSH Terms] OR "ethnic groups"[MeSH Terms] OR "urban health"[MeSH Terms] OR "urban population"[MeSH Terms] OR "health services accessibility"[MeSH Terms] OR "healthcare disparities"[MeSH Terms]) OR ("equit*"[Title/Abstract] OR "inequi*"[Title/Abstract] OR "fair*"[Title/Abstract] OR "disparit*"[Title/Abstract] OR "disadvantage*"[Title/Abstract] OR "inequality*"[Title/Abstract] OR "social inclusion"[Title/Abstract]) | 1,313,752 | 17:24:01 |
| 34 | #29 OR #31 OR #32 OR #33 | | | "ethnic difference"[Title/Abstract] OR "ethnic factor"[Title/Abstract] OR "ethnic discriminat*"[Title/Abstract] OR "ethnical difference"[Title/Abstract] OR "poverty difference*"[Title/Abstract] OR "poverty factor*"[Title/Abstract] OR "socioeconomic difference*"[Title/Abstract] OR "socio economic difference*"[Title/Abstract] OR "socioeconomic factor*"[Title/Abstract] OR "socio economic factor*"[Title/Abstract] OR "social difference*"[Title/Abstract] OR "social factor*"[Title/Abstract] OR "social discriminat*"[Title/Abstract] OR "race difference*"[Title/Abstract] OR "race factor*"[Title/Abstract] OR "race discriminat*"[Title/Abstract] OR "racial difference*"[Title/Abstract] OR "racial factor*"[Title/Abstract] OR "racial discriminat*"[Title/Abstract] OR "religion factor*"[Title/Abstract] OR "religious difference*"[Title/Abstract] OR "religious factor*"[Title/Abstract] OR "religious discriminat*"[Title/Abstract] OR "cultural difference*"[Title/Abstract] OR "cultural factor*"[Title/Abstract] OR "cultural discriminat*"[Title/Abstract] OR "culture difference*"[Title/Abstract] OR "culture factor*"[Title/Abstract] OR "minority difference*"[Title/Abstract] OR "minority factor*"[Title/Abstract] OR "minority discriminat*"[Title/Abstract] OR "indigenous difference*"[Title/Abstract] OR "indigenous factor*"[Title/Abstract] OR "sex difference*"[Title/Abstract] OR "sex factor*"[Title/Abstract] OR "sex discriminat*"[Title/Abstract] OR "gender difference*"[Title/Abstract] OR "gender factor*"[Title/Abstract] OR "gender discriminat*"[Title/Abstract] OR "urban difference*"[Title/Abstract] OR "urban factor*"[Title/Abstract] OR "rural difference*"[Title/Abstract] OR "rural factor*"[Title/Abstract] OR "gender based difference*"[Title/Abstract] OR "gender based factor*"[Title/Abstract] OR "gender based discriminat*"[Title/Abstract] OR "sex factors"[MeSH Terms] OR "geriatrics"[MeSH Terms] OR "homosexuality"[MeSH Terms] OR "disabled persons"[MeSH Terms] OR "educational status"[MeSH Terms] OR "socioeconomic factors"[MeSH Terms] OR "minority groups"[MeSH Terms] OR "ethnic groups"[MeSH Terms] OR "urban health"[MeSH Terms] OR "urban population"[MeSH Terms] OR "health services accessibility"[MeSH Terms] OR "healthcare disparities"[MeSH Terms] OR "equit*"[Title/Abstract] OR "inequi*"[Title/Abstract] OR "fair*"[Title/Abstract] OR "disparit*"[Title/Abstract] OR "disadvantage*"[Title/Abstract] OR "inequality*"[Title/Abstract] OR "social-inclusion"[Title/Abstract] | 1,380,724 | 17:21:30 |
| 33 | social-exlusion[tiab] OR social-inclusion[tiab] | | | "social-inclusion"[Title/Abstract] | 1,441 | 17:20:51 |
| 32 | (equit*[tiab] OR inequi*[tiab] OR fair*[tiab] OR disparit*[tiab] OR disadvantage*[tiab] OR inequality*[tiab] | | | "equit*"[Title/Abstract] OR "inequi*"[Title/Abstract] OR "fair*"[Title/Abstract] OR "disparit*"[Title/Abstract] OR "disadvantage*"[Title/Abstract] OR "inequality*"[Title/Abstract] | 294,150 | 17:20:46 |
| 31 | "sex factors"[Mesh] OR "geriatrics"[Mesh] OR "homosexuality"[Mesh] OR "disabled persons"[Mesh] OR "educational status"[Mesh] OR "socioeconomic factors"[Mesh] OR "minority groups"[Mesh] OR "ethnic groups"[Mesh] OR "urban health"[Mesh] OR "urban population"[Mesh] OR "health services accessibility"[Mesh] OR "healthcare disparities"[Mesh] | | | "sex factors"[MeSH Terms] OR "geriatrics"[MeSH Terms] OR "homosexuality"[MeSH Terms] OR "disabled persons"[MeSH Terms] OR "educational status"[MeSH Terms] OR "socioeconomic factors"[MeSH Terms] OR "minority groups"[MeSH Terms] OR "ethnic groups"[MeSH Terms] OR "urban health"[MeSH Terms] OR "urban population"[MeSH Terms] OR "health services accessibility"[MeSH Terms] OR "healthcare disparities"[MeSH Terms] | 1,090,036 | 17:20:14 |
| 29 | "ethnic difference"[tiab] OR "ethnic factor"[tiab] OR "ethnic discriminat*"[tiab] OR "ethnical difference"[tiab] OR "ethnical factor"[tiab] OR "ethnical discriminat*"[tiab] OR "poverty difference*"[tiab] OR "poverty factor*"[tiab] OR "poverty discriminat*"[tiab] OR OR "low-income difference*"[tiab] OR "low-income factor*"[tiab] OR "low-income discriminat*"[tiab] OR "socioeconomic* difference*"[tiab] OR "socio-economic* difference*"[tiab] OR "socioeconomic* factor*"[tiab] OR "socio-economic* factor*"[tiab] OR "discriminat* factor*"[tiab] OR "socio-economic* discriminat*"[tiab] OR "social difference*"[tiab] OR "social factor*"[tiab] OR "social discriminat*"[tiab] OR "race difference*"[tiab] OR "race factor*"[tiab] OR "race discriminat*"[tiab] OR "racial difference*"[tiab] OR "racial factor*"[tiab] OR "racial discriminat*"[tiab] OR "religion difference*"[tiab] OR "religion factor*"[tiab] OR "religion discriminat*"[tiab] OR "religious difference*"[tiab] OR "religious factor*"[tiab] OR "religious discriminat*"[tiab] OR "cultural difference*"[tiab] OR "cultural factor*"[tiab] OR "cultural discriminat*"[tiab] OR "culture difference*"[tiab] OR "culture factor*"[tiab] OR "culture discriminat*"[tiab] OR "minority difference*"[tiab] OR "minority factor*"[tiab] OR "minority discriminat*"[tiab] OR "refugee difference*"[tiab] OR "refugee factor*"[tiab] OR "refugee discriminat*"[tiab] OR "indigenous difference*"[tiab] OR "indigenous factor*"[tiab] OR "indigenous discriminat*"[tiab] OR "aboriginal difference*"[tiab] OR "aboriginal factor*"[tiab] OR "aboriginal discriminat*"[tiab] OR "sex difference*"[tiab] OR "sex factor*"[tiab] OR "sex discriminat*"[tiab] OR "gender difference*"[tiab] OR "gender factor*"[tiab] OR "gender discriminat*"[tiab] OR "urban difference*"[tiab] OR "urban factor*"[tiab] OR "urban discriminat*"[tiab] OR "rural difference*"[tiab] OR "rural factor*"[tiab] OR "rural discriminat*"[tiab] OR "inner-city difference*"[tiab] OR "inner-city factor*"[tiab] OR "inner-city discriminat*"[tiab] OR "slum difference*"[tiab] OR "slum factor*"[tiab] OR "slum discriminat*"[tiab] OR "gender-based difference*"[tiab] OR "gender-based factor*"[tiab] OR "gender-based discriminat*"[tiab] | | | "ethnic difference"[Title/Abstract] OR "ethnic factor"[Title/Abstract] OR "ethnic discriminat*"[Title/Abstract] OR "ethnical difference"[Title/Abstract] OR "poverty difference*"[Title/Abstract] OR "poverty factor*"[Title/Abstract] OR "socioeconomic difference*"[Title/Abstract] OR "socio economic difference*"[Title/Abstract] OR "socioeconomic factor*"[Title/Abstract] OR "socio economic factor*"[Title/Abstract] OR "social difference*"[Title/Abstract] OR "social factor*"[Title/Abstract] OR "social discriminat*"[Title/Abstract] OR "race difference*"[Title/Abstract] OR "race factor*"[Title/Abstract] OR "race discriminat*"[Title/Abstract] OR "racial difference*"[Title/Abstract] OR "racial factor*"[Title/Abstract] OR "racial discriminat*"[Title/Abstract] OR "religion factor*"[Title/Abstract] OR "religious difference*"[Title/Abstract] OR "religious factor*"[Title/Abstract] OR "religious discriminat*"[Title/Abstract] OR "cultural difference*"[Title/Abstract] OR "cultural factor*"[Title/Abstract] OR "cultural discriminat*"[Title/Abstract] OR "culture difference*"[Title/Abstract] OR "culture factor*"[Title/Abstract] OR "minority difference*"[Title/Abstract] OR "minority factor*"[Title/Abstract] OR "minority discriminat*"[Title/Abstract] OR "indigenous difference*"[Title/Abstract] OR "indigenous factor*"[Title/Abstract] OR "sex difference*"[Title/Abstract] OR "sex factor*"[Title/Abstract] OR "sex discriminat*"[Title/Abstract] OR "gender difference*"[Title/Abstract] OR "gender factor*"[Title/Abstract] OR "gender discriminat*"[Title/Abstract] OR "urban difference*"[Title/Abstract] OR "urban factor*"[Title/Abstract] OR "rural difference*"[Title/Abstract] OR "rural factor*"[Title/Abstract] OR "gender based difference*"[Title/Abstract] OR "gender based factor*"[Title/Abstract] OR "gender based discriminat*"[Title/Abstract] | 136,504 | 17:19:25 |
| 27 | #22 OR #23 OR #24 OR #25 OR #26 | | | "algeria"[MeSH Terms:noexp] OR "egypt"[MeSH Terms:noexp] OR "libya"[MeSH Terms:noexp] OR "morocco"[MeSH Terms:noexp] OR "tunisia"[MeSH Terms:noexp] OR "Cameroon"[MeSH Terms:noexp] OR "Central African Republic"[MeSH Terms:noexp] OR "chad"[MeSH Terms:noexp] OR "Congo"[MeSH Terms:noexp] OR "Equatorial Guinea"[MeSH Terms:noexp] OR "Gabon"[MeSH Terms:noexp] OR "Sao Tome and Principe"[MeSH Terms:noexp] OR "Burundi"[MeSH Terms:noexp] OR "djibouti"[MeSH Terms:noexp] OR "Eritrea"[MeSH Terms:noexp] OR "Ethiopia"[MeSH Terms:noexp] OR "kenya"[MeSH Terms:noexp] OR "Rwanda"[MeSH Terms:noexp] OR "somalia"[MeSH Terms:noexp] OR "south sudan"[MeSH Terms:noexp] OR "Sudan"[MeSH Terms:noexp] OR "tanzania"[MeSH Terms:noexp] OR "Uganda"[MeSH Terms:noexp] OR "Angola"[MeSH Terms:noexp] OR "Botswana"[MeSH Terms:noexp] OR "Eswatini"[MeSH Terms:noexp] OR "Lesotho"[MeSH Terms:noexp] OR "Malawi"[MeSH Terms:noexp] OR "Mozambique"[MeSH Terms:noexp] OR "namibia"[MeSH Terms:noexp] OR "South Africa"[MeSH Terms:noexp] OR "zambia"[MeSH Terms:noexp] OR "Zimbabwe"[MeSH Terms:noexp] OR "Benin"[MeSH Terms:noexp] OR "Burkina Faso"[MeSH Terms:noexp] OR "Cabo Verde"[MeSH Terms:noexp] OR "cote d ivoire"[MeSH Terms:noexp] OR "Gambia"[MeSH Terms:noexp] OR "Ghana"[MeSH Terms:noexp] OR "Guinea"[MeSH Terms:noexp] OR "guinea bissau"[MeSH Terms:noexp] OR "Liberia"[MeSH Terms:noexp] OR "Mali"[MeSH Terms:noexp] OR "Mauritania"[MeSH Terms:noexp] OR "Niger"[MeSH Terms:noexp] OR "Nigeria"[MeSH Terms:noexp] OR "Senegal"[MeSH Terms:noexp] OR "Sierra Leone"[MeSH Terms:noexp] OR "Togo"[MeSH Terms:noexp] OR "Cuba"[MeSH Terms:noexp] OR "Dominica"[MeSH Terms:noexp] OR "Dominican Republic"[MeSH Terms:noexp] OR "Grenada"[MeSH Terms:noexp] OR "Haiti"[MeSH Terms:noexp] OR "jamaica"[MeSH Terms:noexp] OR "Saint Lucia"[MeSH Terms:noexp] OR "Saint Vincent and the Grenadines"[MeSH Terms:noexp] OR "Belize"[MeSH Terms:noexp] OR "Costa Rica"[MeSH Terms:noexp] OR "El Salvador"[MeSH Terms:noexp] OR "Guatemala"[MeSH Terms:noexp] OR "Honduras"[MeSH Terms:noexp] OR "Nicaragua"[MeSH Terms:noexp] OR "Mexico"[MeSH Terms:noexp] OR "argentina"[MeSH Terms:noexp] OR "bolivia"[MeSH Terms:noexp] OR "Brazil"[MeSH Terms:noexp] OR "colombia"[MeSH Terms:noexp] OR "Ecuador"[MeSH Terms:noexp] OR "Guyana"[MeSH Terms:noexp] OR "Paraguay"[MeSH Terms:noexp] OR "Peru"[MeSH Terms:noexp] OR "Suriname"[MeSH Terms:noexp] OR "Venezuela"[MeSH Terms:noexp] OR "Kazakhstan"[MeSH Terms:noexp] OR "kyrgyzstan"[MeSH Terms:noexp] OR "Tajikistan"[MeSH Terms:noexp] OR "Turkmenistan"[MeSH Terms:noexp] OR "Uzbekistan"[MeSH Terms:noexp] OR "russia"[MeSH Terms:noexp] OR "Cambodia"[MeSH Terms:noexp] OR "indonesia"[MeSH Terms:noexp] OR "malaysia"[MeSH Terms:noexp] OR "Myanmar"[MeSH Terms:noexp] OR "philippines"[MeSH Terms:noexp] OR "Thailand"[MeSH Terms:noexp] OR "timor leste"[MeSH Terms:noexp] OR "vietnam"[MeSH Terms:noexp] OR "bangladesh"[MeSH Terms:noexp] OR "Bhutan"[MeSH Terms:noexp] OR "India"[MeSH Terms:noexp] OR "afghanistan"[MeSH Terms:noexp] OR "iran"[MeSH Terms:noexp] OR "iraq"[MeSH Terms:noexp] OR "jordan"[MeSH Terms:noexp] OR "lebanon"[MeSH Terms:noexp] OR "syria"[MeSH Terms:noexp] OR "Turkey"[MeSH Terms:noexp] OR "Yemen"[MeSH Terms:noexp] OR "nepal"[MeSH Terms:noexp] OR "pakistan"[MeSH Terms:noexp] OR "Sri Lanka"[MeSH Terms:noexp] OR "China"[MeSH Terms:noexp] OR "democratic people s republic of korea"[MeSH Terms:noexp] OR "Mongolia"[MeSH Terms:noexp] OR "Republic of North Macedonia"[MeSH Terms:noexp] OR "Comoros"[MeSH Terms:noexp] OR "Micronesia"[MeSH Terms:noexp] OR "American Samoa"[MeSH Terms:noexp] OR "Serbia"[MeSH Terms:noexp] OR "Tonga"[MeSH Terms:noexp] OR "Madagascar"[MeSH Terms:noexp] OR "Moldova"[MeSH Terms:noexp] OR "Papua New Guinea"[MeSH Terms:noexp] OR "Ukraine"[MeSH Terms:noexp] OR "Vanuatu"[MeSH Terms:noexp] OR "Albania"[MeSH Terms:noexp] OR "Armenia"[MeSH Terms:noexp] OR "Azerbaijan"[MeSH Terms:noexp] OR "Republic of Belarus"[MeSH Terms:noexp] OR "Bulgaria"[MeSH Terms:noexp] OR "Fiji"[MeSH Terms:noexp] OR "Georgia"[MeSH Terms:noexp] OR "Kosovo"[MeSH Terms:noexp] OR "Montenegro"[MeSH Terms:noexp] OR "Bosnia and Herzegovina"[MeSH Terms:noexp] OR "afghanistan*"[Title/Abstract] OR "Burkina Faso"[Title/Abstract] OR "Burundi"[Title/Abstract] OR "Central African Republic"[Title/Abstract] OR "chad*"[Title/Abstract] OR "Congo"[Title/Abstract] OR "Eritrea"[Title/Abstract] OR "Ethiopia"[Title/Abstract] OR "Gambia"[Title/Abstract] OR "Guinea"[Title/Abstract] OR "guinea bissau"[Title/Abstract] OR "Haiti"[Title/Abstract] OR "democratic people s republic of korea"[Title/Abstract] OR "Liberia"[Title/Abstract] OR "Madagascar"[Title/Abstract] OR "Malawi"[Title/Abstract] OR "Mali"[Title/Abstract] OR "Mozambique"[Title/Abstract] OR "Niger"[Title/Abstract] OR "Rwanda"[Title/Abstract] OR "Sierra Leone"[Title/Abstract] OR "somalia*"[Title/Abstract] OR "Sudan"[Title/Abstract] OR "Syrian Arab Republic"[Title/Abstract] OR "syria*"[Title/Abstract] OR "Tajikistan"[Title/Abstract] OR "Togo"[Title/Abstract] OR "Uganda"[Title/Abstract] OR "Yemen"[Title/Abstract] OR "North Korea"[Title/Abstract] OR "algeria*"[Title/Abstract] OR "Angola"[Title/Abstract] OR "bangladesh*"[Title/Abstract] OR "Benin"[Title/Abstract] OR "Bhutan"[Title/Abstract] OR "bolivia*"[Title/Abstract] OR "Cabo Verde"[Title/Abstract] OR "Cambodia"[Title/Abstract] OR "Cameroon"[Title/Abstract] OR "Comoros"[Title/Abstract] OR "Congo"[Title/Abstract] OR "cote d ivoire"[Title/Abstract] OR "Ivory Coast"[Title/Abstract] OR "djibouti*"[Title/Abstract] OR "egypt*"[Title/Abstract] OR "El Salvador"[Title/Abstract] OR "Eswatini"[Title/Abstract] OR "Ghana"[Title/Abstract] OR "Honduras"[Title/Abstract] OR "India"[Title/Abstract] OR "kenya*"[Title/Abstract] OR "Kiribati"[Title/Abstract] OR "Kyrgyz Republic"[Title/Abstract] OR "Lao PDR"[Title/Abstract] OR "Lesotho"[Title/Abstract] OR "Mauritania"[Title/Abstract] OR "Micronesia"[Title/Abstract] OR "Moldova"[Title/Abstract] OR "Mongolia"[Title/Abstract] OR "morocco*"[Title/Abstract] OR "Myanmar"[Title/Abstract] OR "nepal*"[Title/Abstract] OR "Nicaragua"[Title/Abstract] OR "Nigeria"[Title/Abstract] OR "pakistan*"[Title/Abstract] OR "Papua New Guinea"[Title/Abstract] OR "philippin*"[Title/Abstract] OR "Sao Tome and Principe"[Title/Abstract] OR "Senegal"[Title/Abstract] OR "Solomon Islands"[Title/Abstract] OR "Sri Lanka"[Title/Abstract] OR "tanzania*"[Title/Abstract] OR "timor leste"[Title/Abstract] OR "tunisi*"[Title/Abstract] OR "ukrain*"[Title/Abstract] OR "Uzbekistan"[Title/Abstract] OR "Vanuatu"[Title/Abstract] OR "vietnam*"[Title/Abstract] OR "West Bank"[Title/Abstract] OR "ghaza"[Title/Abstract] OR "gazza"[Title/Abstract] OR "gaza"[Title/Abstract] OR "zambia*"[Title/Abstract] OR "Zimbabwe"[Title/Abstract] OR "albania*"[Title/Abstract] OR "American Samoa"[Title/Abstract] OR "argentin*"[Title/Abstract] OR "armenia*"[Title/Abstract] OR "Azerbaijan"[Title/Abstract] OR "Belarus"[Title/Abstract] OR "Belize"[Title/Abstract] OR "Bosnia and Herzegovina"[Title/Abstract] OR "Botswana"[Title/Abstract] OR "Brazil"[Title/Abstract] OR "bulgaria*"[Title/Abstract] OR "China"[Title/Abstract] OR "colombia*"[Title/Abstract] OR "Costa Rica"[Title/Abstract] OR "Cuba"[Title/Abstract] OR "Dominica"[Title/Abstract] OR "Dominican Republic"[Title/Abstract] OR "Ecuador"[Title/Abstract] OR "Equatorial Guinea"[Title/Abstract] OR "Fiji"[Title/Abstract] OR "Gabon"[Title/Abstract] OR "Georgia"[Title/Abstract] OR "Grenada"[Title/Abstract] OR "Guatemala"[Title/Abstract] OR "Guyana"[Title/Abstract] OR "indonesia*"[Title/Abstract] OR "iran*"[Title/Abstract] OR "iraq*"[Title/Abstract] OR "jamaica*"[Title/Abstract] OR "jordan*"[Title/Abstract] OR "Kazakhstan"[Title/Abstract] OR "Kosovo"[Title/Abstract] OR "leban*"[Title/Abstract] OR "libya*"[Title/Abstract] OR "malaysia*"[Title/Abstract] OR "Maldives"[Title/Abstract] OR "Marshall Islands"[Title/Abstract] OR "Mexico"[Title/Abstract] OR "Montenegro"[Title/Abstract] OR "namibia*"[Title/Abstract] OR "North Macedonia"[Title/Abstract] OR "Paraguay"[Title/Abstract] OR "Peru"[Title/Abstract] OR "russia*"[Title/Abstract] OR "Samoa"[Title/Abstract] OR "Serbia"[Title/Abstract] OR "South Africa"[Title/Abstract] OR "Saint Lucia"[Title/Abstract] OR "st lucia"[Title/Abstract] OR "Saint Vincent and the Grenadines"[Title/Abstract] OR "st vincent and the grenadines"[Title/Abstract] OR "Suriname"[Title/Abstract] OR "Thailand"[Title/Abstract] OR "Tonga"[Title/Abstract] OR "Turkey"[Title/Abstract] OR "Turkmenistan"[Title/Abstract] OR "Tuvalu"[Title/Abstract] OR "Venezuela"[Title/Abstract] OR "Low-income-country"[Title/Abstract] OR "middle-income-country"[Title/Abstract] OR "Developing Countries"[MeSH Terms:noexp] OR "Developing Countries"[Title/Abstract] OR "less-developed Countries"[Title/Abstract] OR "least-developed Countries"[Title/Abstract] OR "underdeveloped Countries"[Title/Abstract] OR "under-developed Countries"[Title/Abstract] OR "underserved Countries"[Title/Abstract] OR "deprived Countries"[Title/Abstract] OR "poor Countries"[Title/Abstract] OR "transition Countries"[Title/Abstract] OR "transitional Countries"[Title/Abstract] OR "Developing Nation"[Title/Abstract] OR "less-developed Nation"[Title/Abstract] OR "underdeveloped Nation"[Title/Abstract] OR "poor Nation"[Title/Abstract] OR "Developing Population"[Title/Abstract] OR "underdeveloped Population"[Title/Abstract] OR "under-served Population"[Title/Abstract] OR "underserved Population"[Title/Abstract] OR "deprived Population"[Title/Abstract] OR "poor Population"[Title/Abstract] OR "transition Population"[Title/Abstract] OR "transitional Population"[Title/Abstract] OR "Developing World"[Title/Abstract] OR "less-developed World"[Title/Abstract] OR "underdeveloped World"[Title/Abstract] OR "under-developed World"[Title/Abstract] OR "poor World"[Title/Abstract] OR "transitional World"[Title/Abstract] OR "developing econom*"[Title/Abstract] OR "less developed econom*"[Title/Abstract] OR "least developed econom*"[Title/Abstract] OR "underdeveloped econom*"[Title/Abstract] OR "poor econom*"[Title/Abstract] OR "transition econom*"[Title/Abstract] OR "transitional econom*"[Title/Abstract] OR "developing communit*"[Title/Abstract] OR "less developed communit*"[Title/Abstract] OR "underdeveloped communit*"[Title/Abstract] OR "under developed communit*"[Title/Abstract] OR "under served communit*"[Title/Abstract] OR "underserved communit*"[Title/Abstract] OR "deprived communit*"[Title/Abstract] OR "poor communit*"[Title/Abstract] OR "transition communit*"[Title/Abstract] OR "transitional communit*"[Title/Abstract] OR "Developing State"[Title/Abstract] OR "less-developed State"[Title/Abstract] OR "underdeveloped State"[Title/Abstract] OR "underserved State"[Title/Abstract] OR "deprived State"[Title/Abstract] OR "poor State"[Title/Abstract] OR "transition State"[Title/Abstract] OR "transitional State"[Title/Abstract] OR "Developing People"[Title/Abstract] OR "underserved People"[Title/Abstract] OR "deprived People"[Title/Abstract] OR "Developing Cities"[Title/Abstract] OR "less-developed Cities"[Title/Abstract] OR "underdeveloped Cities"[Title/Abstract] OR "poor Cities"[Title/Abstract] OR "Developing City"[Title/Abstract] OR "less-developed City"[Title/Abstract] OR "underdeveloped City"[Title/Abstract] OR "deprived City"[Title/Abstract] OR "poor City"[Title/Abstract] | 1,705,315 | 17:18:34 |
| 26 | "Developing Countries"[tiab] OR "less-developed Countries"[tiab] OR "least-developed Countries"[tiab] OR "underdeveloped Countries"[tiab] OR "under-developed Countries"[tiab] OR "under-served Countries"[tiab] OR "underserved Countries"[tiab] OR "deprived Countries"[tiab] OR "poor Countries"[tiab] OR "transition Countries"[tiab] OR "transitional Countries"[tiab] OR "Developing Nation"[tiab] OR "less-developed Nation"[tiab] OR "least-developed Nation"[tiab] OR "underdeveloped Nation"[tiab] OR "under-developed Nation"[tiab] OR "under-served Nation"[tiab] OR "underserved Nation"[tiab] OR "deprived Nation"[tiab] OR "poor Nation"[tiab] OR "transition Nation"[tiab] OR "transitional Nation"[tiab] OR "Developing Population"[tiab] OR "less-developed Population"[tiab] OR "least-developed Population"[tiab] OR "underdeveloped Population"[tiab] OR "under-developed Population"[tiab] OR "under-served Population"[tiab] OR "underserved Population"[tiab] OR "deprived Population"[tiab] OR "poor Population"[tiab] OR "transition Population"[tiab] OR "transitional Population"[tiab] OR "Developing World"[tiab] OR "less-developed World"[tiab] OR "least-developed World"[tiab] OR "underdeveloped World"[tiab] OR "under-developed World"[tiab] OR "under-served World"[tiab] OR "underserved World"[tiab] OR "deprived World"[tiab] OR "poor World"[tiab] OR "transition World"[tiab] OR "transitional World"[tiab] OR "Developing Econom*"[tiab] OR "less-developed Econom*"[tiab] OR "least-developed Econom*"[tiab] OR "underdeveloped Econom*"[tiab] OR "under-developed Econom*"[tiab] OR "under-served Econom*"[tiab] OR "underserved Econom*"[tiab] OR "deprived Econom*"[tiab] OR "poor Econom*"[tiab] OR "transition Econom*"[tiab] OR "transitional Econom*"[tiab] OR "Developing Communit*"[tiab] OR "less-developed Communit*"[tiab] OR "least-developed Communit*"[tiab] OR "underdeveloped Communit*"[tiab] OR "under-developed Communit*"[tiab] OR "under-served Communit*"[tiab] OR "underserved Communit*"[tiab] OR "deprived Communit*"[tiab] OR "poor Communit*"[tiab] OR "transition Communit*"[tiab] OR "transitional Communit*"[tiab] OR "Developing State"[tiab] OR "less-developed State"[tiab] OR "least-developed State"[tiab] OR "underdeveloped State"[tiab] OR "under-developed State"[tiab] OR "under-served State"[tiab] OR "underserved State"[tiab] OR "deprived State"[tiab] OR "poor State"[tiab] OR "transition State"[tiab] OR "transitional State"[tiab] OR "Developing People"[tiab] OR "less-developed People"[tiab] OR "least-developed People"[tiab] OR "underdeveloped People"[tiab] OR "under-developed People"[tiab] OR "under-served People"[tiab] OR "underserved People"[tiab] OR "deprived People"[tiab] OR "po8or People"[tiab] OR "transition People"[tiab] OR "transitional People"[tiab] OR "Developing Cities"[tiab] OR "less-developed Cities"[tiab] OR "least-developed Cities"[tiab] OR "underdeveloped Cities"[tiab] OR "under-developed Cities"[tiab] OR "under-served Cities"[tiab] OR "underserved Cities"[tiab] OR "deprived Cities"[tiab] OR "poor Cities"[tiab] OR "transition Cities"[tiab] OR "transitional Cities"[tiab] OR "Developing City"[tiab] OR "less-developed City"[tiab] OR "least-developed City"[tiab] OR "underdeveloped City"[tiab] OR "under-developed City"[tiab] OR "under-served City"[tiab] OR "underserved City"[tiab] OR "deprived City"[tiab] OR "poor City"[tiab] OR "transition City"[tiab] OR "transitional City"[tiab] | | | "Developing Countries"[Title/Abstract] OR "less-developed Countries"[Title/Abstract] OR "least-developed Countries"[Title/Abstract] OR "underdeveloped Countries"[Title/Abstract] OR "under-developed Countries"[Title/Abstract] OR "underserved Countries"[Title/Abstract] OR "deprived Countries"[Title/Abstract] OR "poor Countries"[Title/Abstract] OR "transition Countries"[Title/Abstract] OR "transitional Countries"[Title/Abstract] OR "Developing Nation"[Title/Abstract] OR "less-developed Nation"[Title/Abstract] OR "underdeveloped Nation"[Title/Abstract] OR "poor Nation"[Title/Abstract] OR "Developing Population"[Title/Abstract] OR "underdeveloped Population"[Title/Abstract] OR "under-served Population"[Title/Abstract] OR "underserved Population"[Title/Abstract] OR "deprived Population"[Title/Abstract] OR "poor Population"[Title/Abstract] OR "transition Population"[Title/Abstract] OR "transitional Population"[Title/Abstract] OR "Developing World"[Title/Abstract] OR "less-developed World"[Title/Abstract] OR "underdeveloped World"[Title/Abstract] OR "under-developed World"[Title/Abstract] OR "poor World"[Title/Abstract] OR "transitional World"[Title/Abstract] OR "developing econom*"[Title/Abstract] OR "less developed econom*"[Title/Abstract] OR "least developed econom*"[Title/Abstract] OR "underdeveloped econom*"[Title/Abstract] OR "poor econom*"[Title/Abstract] OR "transition econom*"[Title/Abstract] OR "transitional econom*"[Title/Abstract] OR "developing communit*"[Title/Abstract] OR "less developed communit*"[Title/Abstract] OR "underdeveloped communit*"[Title/Abstract] OR "under developed communit*"[Title/Abstract] OR "under served communit*"[Title/Abstract] OR "underserved communit*"[Title/Abstract] OR "deprived communit*"[Title/Abstract] OR "poor communit*"[Title/Abstract] OR "transition communit*"[Title/Abstract] OR "transitional communit*"[Title/Abstract] OR "Developing State"[Title/Abstract] OR "less-developed State"[Title/Abstract] OR "underdeveloped State"[Title/Abstract] OR "underserved State"[Title/Abstract] OR "deprived State"[Title/Abstract] OR "poor State"[Title/Abstract] OR "transition State"[Title/Abstract] OR "transitional State"[Title/Abstract] OR "Developing People"[Title/Abstract] OR "underserved People"[Title/Abstract] OR "deprived People"[Title/Abstract] OR "Developing Cities"[Title/Abstract] OR "less-developed Cities"[Title/Abstract] OR "underdeveloped Cities"[Title/Abstract] OR "poor Cities"[Title/Abstract] OR "Developing City"[Title/Abstract] OR "less-developed City"[Title/Abstract] OR "underdeveloped City"[Title/Abstract] OR "deprived City"[Title/Abstract] OR "poor City"[Title/Abstract] | 122,105 | 17:17:58 |
| 25 | "Developing Countries"[Mesh:noexp] | | | "Developing Countries"[MeSH Terms:noexp] | 77,225 | 17:17:50 |
| 24 | Low-income-country[tiab] OR middle-income-country[tiab] | | | "Low-income-country"[Title/Abstract] OR "middle-income-country"[Title/Abstract] | 3,462 | 17:17:44 |
| 23 | Afghanistan*[tiab] OR "Burkina Faso"[tiab] OR Burundi[tiab] OR "Central African Republic"[tiab] OR Chad*[tiab] OR Congo[tiab] OR Eritrea[tiab] OR Ethiopia[tiab] OR Gambia[tiab] OR Guinea[tiab] OR "Guinea-Bissau"[tiab] OR Haiti[tiab] OR "Democratic People’s Republic of Korea"[tiab] OR Liberia[tiab] OR Madagascar[tiab] OR Malawi[tiab] OR Mali[tiab] OR Mozambique[tiab] OR Niger[tiab] OR Rwanda[tiab] OR "Sierra Leone"[tiab] OR Somalia*[tiab] OR Sudan[tiab] OR "Syrian Arab Republic"[tiab] OR Syria*[tiab] OR Tajikistan[tiab] OR Togo[tiab] OR Uganda[tiab] OR Yemen[tiab] OR "North Korea"[tiab] OR Algeria*[tiab] OR Angola[tiab] OR Bangladesh*[tiab] OR Benin[tiab] OR Bhutan[tiab] OR Bolivia*[tiab] OR "Cabo Verde"[tiab] OR Cambodia[tiab] OR Cameroon[tiab] OR Comoros[tiab] OR Congo[tiab] OR "Côte d'Ivoire"[tiab] OR "Ivory Coast"[tiab] OR Djibouti*[tiab] OR Egypt*[tiab] OR "El Salvador"[tiab] OR Eswatini[tiab] OR Ghana[tiab] OR Honduras[tiab] OR India[tiab] OR Kenya*[tiab] OR Kiribati[tiab] OR "Kyrgyz Republic"[tiab] OR "Lao PDR"[tiab] OR Lesotho[tiab] OR Mauritania[tiab] OR Micronesia[tiab] OR Moldova[tiab] OR Mongolia[tiab] OR Morocco*[tiab] OR Myanmar[tiab] OR Nepal*[tiab] OR Nicaragua[tiab] OR Nigeria[tiab] OR Pakistan*[tiab] OR "Papua New Guinea"[tiab] OR Philippin*[tiab] OR "Sao Tome and Principe"[tiab] OR Senegal[tiab] OR "Solomon Islands"[tiab] OR "Sri Lanka"[tiab] OR Tanzania*[tiab] OR "Timor-Leste"[tiab] OR Tunisi*[tiab] OR Ukrain*[tiab] OR Uzbekistan[tiab] OR Vanuatu[tiab] OR Vietnam*[tiab] OR "West Bank"[tiab] OR ghazza[tiab] OR ghaza[tiab] OR gazza[tiab] OR gaza[tiab] OR Zambia*[tiab] OR Zimbabwe[tiab] OR Albania*[tiab] OR "American Samoa"[tiab] OR Argentin*[tiab] OR Armenia*[tiab] OR Azerbaijan[tiab] OR Belarus[tiab] OR Belize[tiab] OR "Bosnia and Herzegovina"[tiab] OR Botswana[tiab] OR Brazil[tiab] OR Bulgaria*[tiab] OR China[tiab] OR Colombia*[tiab] OR "Costa Rica"[tiab] OR Cuba[tiab] OR Dominica[tiab] OR "Dominican Republic"[tiab] OR Ecuador[tiab] OR "Equatorial Guinea"[tiab] OR Fiji[tiab] OR Gabon[tiab] OR Georgia[tiab] OR Grenada[tiab] OR Guatemala[tiab] OR Guyana[tiab] OR Indonesia*[tiab] OR Iran*[tiab] OR Iraq*[tiab] OR Jamaica*[tiab] OR Jordan*[tiab] OR Kazakhstan[tiab] OR Kosovo[tiab] OR Leban*[tiab] OR Libya*[tiab] OR Malaysia*[tiab] OR Maldives[tiab] OR "Marshall Islands"[tiab] OR Mexico[tiab] OR Montenegro[tiab] OR Namibia*[tiab] OR "North Macedonia"[tiab] OR Paraguay[tiab] OR Peru[tiab] OR Russia*[tiab] OR Samoa[tiab] OR Serbia[tiab] OR "South Africa"[tiab] OR "Saint Lucia"[tiab] OR "St. Lucia"[tiab] OR "Saint Vincent and the Grenadines"[tiab] OR "St. Vincent and the Grenadines"[tiab] OR Suriname[tiab] OR Thailand[tiab] OR Tonga[tiab] OR Turkey[tiab] OR Turkmenistan[tiab] OR Tuvalu[tiab] OR Venezuela[tiab] | | | "afghanistan*"[Title/Abstract] OR "Burkina Faso"[Title/Abstract] OR "Burundi"[Title/Abstract] OR "Central African Republic"[Title/Abstract] OR "chad*"[Title/Abstract] OR "Congo"[Title/Abstract] OR "Eritrea"[Title/Abstract] OR "Ethiopia"[Title/Abstract] OR "Gambia"[Title/Abstract] OR "Guinea"[Title/Abstract] OR "Guinea-Bissau"[Title/Abstract] OR "Haiti"[Title/Abstract] OR "Democratic People's Republic of Korea"[Title/Abstract] OR "Liberia"[Title/Abstract] OR "Madagascar"[Title/Abstract] OR "Malawi"[Title/Abstract] OR "Mali"[Title/Abstract] OR "Mozambique"[Title/Abstract] OR "Niger"[Title/Abstract] OR "Rwanda"[Title/Abstract] OR "Sierra Leone"[Title/Abstract] OR "somalia*"[Title/Abstract] OR "Sudan"[Title/Abstract] OR "Syrian Arab Republic"[Title/Abstract] OR "syria*"[Title/Abstract] OR "Tajikistan"[Title/Abstract] OR "Togo"[Title/Abstract] OR "Uganda"[Title/Abstract] OR "Yemen"[Title/Abstract] OR "North Korea"[Title/Abstract] OR "algeria*"[Title/Abstract] OR "Angola"[Title/Abstract] OR "bangladesh*"[Title/Abstract] OR "Benin"[Title/Abstract] OR "Bhutan"[Title/Abstract] OR "bolivia*"[Title/Abstract] OR "Cabo Verde"[Title/Abstract] OR "Cambodia"[Title/Abstract] OR "Cameroon"[Title/Abstract] OR "Comoros"[Title/Abstract] OR "Congo"[Title/Abstract] OR "Cote d'Ivoire"[Title/Abstract] OR "Ivory Coast"[Title/Abstract] OR "djibouti*"[Title/Abstract] OR "egypt*"[Title/Abstract] OR "El Salvador"[Title/Abstract] OR "Eswatini"[Title/Abstract] OR "Ghana"[Title/Abstract] OR "Honduras"[Title/Abstract] OR "India"[Title/Abstract] OR "kenya*"[Title/Abstract] OR "Kiribati"[Title/Abstract] OR "Kyrgyz Republic"[Title/Abstract] OR "Lao PDR"[Title/Abstract] OR "Lesotho"[Title/Abstract] OR "Mauritania"[Title/Abstract] OR "Micronesia"[Title/Abstract] OR "Moldova"[Title/Abstract] OR "Mongolia"[Title/Abstract] OR "morocco*"[Title/Abstract] OR "Myanmar"[Title/Abstract] OR "nepal*"[Title/Abstract] OR "Nicaragua"[Title/Abstract] OR "Nigeria"[Title/Abstract] OR "pakistan*"[Title/Abstract] OR "Papua New Guinea"[Title/Abstract] OR "philippin*"[Title/Abstract] OR "Sao Tome and Principe"[Title/Abstract] OR "Senegal"[Title/Abstract] OR "Solomon Islands"[Title/Abstract] OR "Sri Lanka"[Title/Abstract] OR "tanzania*"[Title/Abstract] OR "Timor-Leste"[Title/Abstract] OR "tunisi*"[Title/Abstract] OR "ukrain*"[Title/Abstract] OR "Uzbekistan"[Title/Abstract] OR "Vanuatu"[Title/Abstract] OR "vietnam*"[Title/Abstract] OR "West Bank"[Title/Abstract] OR "ghaza"[Title/Abstract] OR "gazza"[Title/Abstract] OR "gaza"[Title/Abstract] OR "zambia*"[Title/Abstract] OR "Zimbabwe"[Title/Abstract] OR "albania*"[Title/Abstract] OR "American Samoa"[Title/Abstract] OR "argentin*"[Title/Abstract] OR "armenia*"[Title/Abstract] OR "Azerbaijan"[Title/Abstract] OR "Belarus"[Title/Abstract] OR "Belize"[Title/Abstract] OR "Bosnia and Herzegovina"[Title/Abstract] OR "Botswana"[Title/Abstract] OR "Brazil"[Title/Abstract] OR "bulgaria*"[Title/Abstract] OR "China"[Title/Abstract] OR "colombia*"[Title/Abstract] OR "Costa Rica"[Title/Abstract] OR "Cuba"[Title/Abstract] OR "Dominica"[Title/Abstract] OR "Dominican Republic"[Title/Abstract] OR "Ecuador"[Title/Abstract] OR "Equatorial Guinea"[Title/Abstract] OR "Fiji"[Title/Abstract] OR "Gabon"[Title/Abstract] OR "Georgia"[Title/Abstract] OR "Grenada"[Title/Abstract] OR "Guatemala"[Title/Abstract] OR "Guyana"[Title/Abstract] OR "indonesia*"[Title/Abstract] OR "iran*"[Title/Abstract] OR "iraq*"[Title/Abstract] OR "jamaica*"[Title/Abstract] OR "jordan*"[Title/Abstract] OR "Kazakhstan"[Title/Abstract] OR "Kosovo"[Title/Abstract] OR "leban*"[Title/Abstract] OR "libya*"[Title/Abstract] OR "malaysia*"[Title/Abstract] OR "Maldives"[Title/Abstract] OR "Marshall Islands"[Title/Abstract] OR "Mexico"[Title/Abstract] OR "Montenegro"[Title/Abstract] OR "namibia*"[Title/Abstract] OR "North Macedonia"[Title/Abstract] OR "Paraguay"[Title/Abstract] OR "Peru"[Title/Abstract] OR "russia*"[Title/Abstract] OR "Samoa"[Title/Abstract] OR "Serbia"[Title/Abstract] OR "South Africa"[Title/Abstract] OR "Saint Lucia"[Title/Abstract] OR "st lucia"[Title/Abstract] OR "Saint Vincent and the Grenadines"[Title/Abstract] OR "st vincent and the grenadines"[Title/Abstract] OR "Suriname"[Title/Abstract] OR "Thailand"[Title/Abstract] OR "Tonga"[Title/Abstract] OR "Turkey"[Title/Abstract] OR "Turkmenistan"[Title/Abstract] OR "Tuvalu"[Title/Abstract] OR "Venezuela"[Title/Abstract] | 1,252,431 | 17:17:37 |
| 22 | "algeria"[Mesh:noexp] OR "egypt"[Mesh:noexp] OR "libya"[Mesh:noexp] OR "morocco"[Mesh:noexp] OR "tunisia"[Mesh:noexp] OR "cameroon"[Mesh:noexp] OR "central african republic"[Mesh:noexp] OR "chad"[Mesh:noexp] OR "congo"[Mesh:noexp] OR "equatorial guinea"[Mesh:noexp] OR "gabon"[Mesh:noexp] OR "sao tome and principe"[Mesh:noexp] OR "burundi"[Mesh:noexp] OR "djibouti"[Mesh:noexp] OR "eritrea"[Mesh:noexp] OR "ethiopia"[Mesh:noexp] OR "kenya"[Mesh:noexp] OR "rwanda"[Mesh:noexp] OR "somalia"[Mesh:noexp] OR "south sudan"[Mesh:noexp] OR "sudan"[Mesh:noexp] OR "tanzania"[Mesh:noexp] OR "uganda"[Mesh:noexp] OR "angola"[Mesh:noexp] OR "botswana"[Mesh:noexp] OR "eswatini"[Mesh:noexp] OR "lesotho"[Mesh:noexp] OR "malawi"[Mesh:noexp] OR "mozambique"[Mesh:noexp] OR "namibia"[Mesh:noexp] OR "south africa"[Mesh:noexp] OR "zambia"[Mesh:noexp] OR "zimbabwe"[Mesh:noexp] OR "benin"[Mesh:noexp] OR "burkina faso"[Mesh:noexp] OR "cabo verde"[Mesh:noexp] OR "cote d'ivoire"[Mesh:noexp] OR "gambia"[Mesh:noexp] OR "ghana"[Mesh:noexp] OR "guinea"[Mesh:noexp] OR "guinea-bissau"[Mesh:noexp] OR "liberia"[Mesh:noexp] OR "mali"[Mesh:noexp] OR "mauritania"[Mesh:noexp] OR "niger"[Mesh:noexp] OR "nigeria"[Mesh:noexp] OR "senegal"[Mesh:noexp] OR "sierra leone"[Mesh:noexp] OR "togo"[Mesh:noexp] OR "cuba"[Mesh:noexp] OR "dominica"[Mesh:noexp] OR "dominican republic"[Mesh:noexp] OR "grenada"[Mesh:noexp] OR "haiti"[Mesh:noexp] OR "jamaica"[Mesh:noexp] OR "saint lucia"[Mesh:noexp] OR "saint vincent and the grenadines"[Mesh:noexp] OR "belize"[Mesh:noexp] OR "costa rica"[Mesh:noexp] OR "el salvador"[Mesh:noexp] OR "guatemala"[Mesh:noexp] OR "honduras"[Mesh:noexp] OR "nicaragua"[Mesh:noexp] OR "mexico"[Mesh:noexp] OR "argentina"[Mesh:noexp] OR "bolivia"[Mesh:noexp] OR "brazil"[Mesh:noexp] OR "colombia"[Mesh:noexp] OR "ecuador"[Mesh:noexp] OR "guyana"[Mesh:noexp] OR "paraguay"[Mesh:noexp] OR "peru"[Mesh:noexp] OR "suriname"[Mesh:noexp] OR "venezuela"[Mesh:noexp] OR "kazakhstan"[Mesh:noexp] OR "kyrgyzstan"[Mesh:noexp] OR "tajikistan"[Mesh:noexp] OR "turkmenistan"[Mesh:noexp] OR "uzbekistan"[Mesh:noexp] OR "russia"[Mesh:noexp] OR "cambodia"[Mesh:noexp] OR "indonesia"[Mesh:noexp] OR "malaysia"[Mesh:noexp] OR "myanmar"[Mesh:noexp] OR "philippines"[Mesh:noexp] OR "thailand"[Mesh:noexp] OR "timor-leste"[Mesh:noexp] OR "vietnam"[Mesh:noexp] OR "bangladesh"[Mesh:noexp] OR "bhutan"[Mesh:noexp] OR "india"[Mesh:noexp] OR "afghanistan"[Mesh:noexp] OR "iran"[Mesh:noexp] OR "iraq"[Mesh:noexp] OR "jordan"[Mesh:noexp] OR "lebanon"[Mesh:noexp] OR "syria"[Mesh:noexp] OR "turkey"[Mesh:noexp] OR "yemen"[Mesh:noexp] OR "nepal"[Mesh:noexp] OR "pakistan"[Mesh:noexp] OR "sri lanka"[Mesh:noexp] OR "china"[Mesh:noexp] OR "democratic people's republic of korea"[Mesh:noexp] OR "mongolia"[Mesh:noexp] OR "Republic of North Macedonia"[Mesh:noexp] OR "Comoros"[Mesh:noexp] OR "Micronesia"[Mesh:noexp] OR "American Samoa"[Mesh:noexp] OR "Serbia"[Mesh:noexp] OR "Tonga"[Mesh:noexp] OR "Madagascar"[Mesh:noexp] OR "Moldova"[Mesh:noexp] OR "Papua New Guinea"[Mesh:noexp] OR "Ukraine"[Mesh:noexp] OR "Vanuatu"[Mesh:noexp] OR "Albania"[Mesh:noexp] OR "Armenia"[Mesh:noexp] OR "Azerbaijan"[Mesh:noexp] OR "Republic of Belarus"[Mesh:noexp] OR "Bulgaria"[Mesh:noexp] OR "Fiji"[Mesh:noexp] OR "Georgia"[Mesh:noexp] OR "Kosovo"[Mesh:noexp] OR "Montenegro"[Mesh:noexp] OR "Bosnia and Herzegovina"[Mesh:noexp] | | | "algeria"[MeSH Terms:noexp] OR "egypt"[MeSH Terms:noexp] OR "libya"[MeSH Terms:noexp] OR "morocco"[MeSH Terms:noexp] OR "tunisia"[MeSH Terms:noexp] OR "cameroon"[MeSH Terms:noexp] OR "central african republic"[MeSH Terms:noexp] OR "chad"[MeSH Terms:noexp] OR "congo"[MeSH Terms:noexp] OR "equatorial guinea"[MeSH Terms:noexp] OR "gabon"[MeSH Terms:noexp] OR "sao tome and principe"[MeSH Terms:noexp] OR "burundi"[MeSH Terms:noexp] OR "djibouti"[MeSH Terms:noexp] OR "eritrea"[MeSH Terms:noexp] OR "ethiopia"[MeSH Terms:noexp] OR "kenya"[MeSH Terms:noexp] OR "rwanda"[MeSH Terms:noexp] OR "somalia"[MeSH Terms:noexp] OR "south sudan"[MeSH Terms:noexp] OR "sudan"[MeSH Terms:noexp] OR "tanzania"[MeSH Terms:noexp] OR "uganda"[MeSH Terms:noexp] OR "angola"[MeSH Terms:noexp] OR "botswana"[MeSH Terms:noexp] OR "eswatini"[MeSH Terms:noexp] OR "lesotho"[MeSH Terms:noexp] OR "malawi"[MeSH Terms:noexp] OR "mozambique"[MeSH Terms:noexp] OR "namibia"[MeSH Terms:noexp] OR "south africa"[MeSH Terms:noexp] OR "zambia"[MeSH Terms:noexp] OR "zimbabwe"[MeSH Terms:noexp] OR "benin"[MeSH Terms:noexp] OR "burkina faso"[MeSH Terms:noexp] OR "cabo verde"[MeSH Terms:noexp] OR "cote d'ivoire"[MeSH Terms:noexp] OR "gambia"[MeSH Terms:noexp] OR "ghana"[MeSH Terms:noexp] OR "guinea"[MeSH Terms:noexp] OR "guinea-bissau"[MeSH Terms:noexp] OR "liberia"[MeSH Terms:noexp] OR "mali"[MeSH Terms:noexp] OR "mauritania"[MeSH Terms:noexp] OR "niger"[MeSH Terms:noexp] OR "nigeria"[MeSH Terms:noexp] OR "senegal"[MeSH Terms:noexp] OR "sierra leone"[MeSH Terms:noexp] OR "togo"[MeSH Terms:noexp] OR "cuba"[MeSH Terms:noexp] OR "dominica"[MeSH Terms:noexp] OR "dominican republic"[MeSH Terms:noexp] OR "grenada"[MeSH Terms:noexp] OR "haiti"[MeSH Terms:noexp] OR "jamaica"[MeSH Terms:noexp] OR "saint lucia"[MeSH Terms:noexp] OR "saint vincent and the grenadines"[MeSH Terms:noexp] OR "belize"[MeSH Terms:noexp] OR "costa rica"[MeSH Terms:noexp] OR "el salvador"[MeSH Terms:noexp] OR "guatemala"[MeSH Terms:noexp] OR "honduras"[MeSH Terms:noexp] OR "nicaragua"[MeSH Terms:noexp] OR "mexico"[MeSH Terms:noexp] OR "argentina"[MeSH Terms:noexp] OR "bolivia"[MeSH Terms:noexp] OR "brazil"[MeSH Terms:noexp] OR "colombia"[MeSH Terms:noexp] OR "ecuador"[MeSH Terms:noexp] OR "guyana"[MeSH Terms:noexp] OR "paraguay"[MeSH Terms:noexp] OR "peru"[MeSH Terms:noexp] OR "suriname"[MeSH Terms:noexp] OR "venezuela"[MeSH Terms:noexp] OR "kazakhstan"[MeSH Terms:noexp] OR "kyrgyzstan"[MeSH Terms:noexp] OR "tajikistan"[MeSH Terms:noexp] OR "turkmenistan"[MeSH Terms:noexp] OR "uzbekistan"[MeSH Terms:noexp] OR "russia"[MeSH Terms:noexp] OR "cambodia"[MeSH Terms:noexp] OR "indonesia"[MeSH Terms:noexp] OR "malaysia"[MeSH Terms:noexp] OR "myanmar"[MeSH Terms:noexp] OR "philippines"[MeSH Terms:noexp] OR "thailand"[MeSH Terms:noexp] OR "timor-leste"[MeSH Terms:noexp] OR "vietnam"[MeSH Terms:noexp] OR "bangladesh"[MeSH Terms:noexp] OR "bhutan"[MeSH Terms:noexp] OR "india"[MeSH Terms:noexp] OR "afghanistan"[MeSH Terms:noexp] OR "iran"[MeSH Terms:noexp] OR "iraq"[MeSH Terms:noexp] OR "jordan"[MeSH Terms:noexp] OR "lebanon"[MeSH Terms:noexp] OR "syria"[MeSH Terms:noexp] OR "turkey"[MeSH Terms:noexp] OR "yemen"[MeSH Terms:noexp] OR "nepal"[MeSH Terms:noexp] OR "pakistan"[MeSH Terms:noexp] OR "sri lanka"[MeSH Terms:noexp] OR "china"[MeSH Terms:noexp] OR "democratic people's republic of korea"[MeSH Terms:noexp] OR "mongolia"[MeSH Terms:noexp] OR "Republic of North Macedonia"[MeSH Terms:noexp] OR "Comoros"[MeSH Terms:noexp] OR "Micronesia"[MeSH Terms:noexp] OR "American Samoa"[MeSH Terms:noexp] OR "Serbia"[MeSH Terms:noexp] OR "Tonga"[MeSH Terms:noexp] OR "Madagascar"[MeSH Terms:noexp] OR "Moldova"[MeSH Terms:noexp] OR "Papua New Guinea"[MeSH Terms:noexp] OR "Ukraine"[MeSH Terms:noexp] OR "Vanuatu"[MeSH Terms:noexp] OR "Albania"[MeSH Terms:noexp] OR "Armenia"[MeSH Terms:noexp] OR "Azerbaijan"[MeSH Terms:noexp] OR "Republic of Belarus"[MeSH Terms:noexp] OR "Bulgaria"[MeSH Terms:noexp] OR "Fiji"[MeSH Terms:noexp] OR "Georgia"[MeSH Terms:noexp] OR "Kosovo"[MeSH Terms:noexp] OR "Montenegro"[MeSH Terms:noexp] OR "Bosnia and Herzegovina"[MeSH Terms:noexp] | 1,057,352 | 17:17:29 |
| 21 | #14 OR #15 OR #16 OR #17 OR #18 OR #19 OR #20 | | | "Women"[MeSH Terms:noexp] OR "adolescent health"[MeSH Terms:noexp] OR "child health"[MeSH Terms:noexp] OR "infant health"[MeSH Terms:noexp] OR "reproductive health"[MeSH Terms:noexp] OR "sexual health"[MeSH Terms:noexp] OR "child health services"[MeSH Terms:noexp] OR "family planning services"[MeSH Terms:noexp] OR ("Women"[Title/Abstract] OR "woman"[Title/Abstract] OR "child"[Title/Abstract] OR "children"[Title/Abstract] OR "infant"[Title/Abstract] OR "mother"[Title/Abstract] OR "maternal"[Title/Abstract] OR "neonat*"[Title/Abstract] OR "teen*"[Title/Abstract] OR "adolescen*"[Title/Abstract] OR "youth"[Title/Abstract] OR "young"[Title/Abstract] OR "youngster"[Title/Abstract] OR "newborn"[Title/Abstract] OR "new-born"[Title/Abstract]) OR ("Thirteen years old"[Title/Abstract] OR "fourteen years old"[Title/Abstract] OR "fifteen years old"[Title/Abstract] OR "sixteen years old"[Title/Abstract] OR "seventeen years old"[Title/Abstract] OR "eighteen years old"[Title/Abstract] OR "13 years old"[Title/Abstract] OR "14 years old"[Title/Abstract] OR "15 years old"[Title/Abstract] OR "16 years old"[Title/Abstract] OR "17 years old"[Title/Abstract] OR "18 years old"[Title/Abstract] OR "13 yo"[Title/Abstract] OR "15 yo"[Title/Abstract] OR "16 yo"[Title/Abstract] OR "17 yo"[Title/Abstract] OR "18 yo"[Title/Abstract] OR "13 y o"[Title/Abstract] OR "14 y o"[Title/Abstract] OR "15 y o"[Title/Abstract] OR "16 y o"[Title/Abstract] OR "17 y o"[Title/Abstract] OR "18 y o"[Title/Abstract]) OR ("prenatal care"[Title/Abstract] OR "postnatal care"[Title/Abstract] OR "postpartum care"[Title/Abstract] OR "antenatal care"[Title/Abstract] OR "intrapartum care"[Title/Abstract] OR "abortion care"[Title/Abstract] OR "post-abortion care"[Title/Abstract] OR "delivery care"[Title/Abstract] OR "prenatal service"[Title/Abstract] OR "postnatal service"[Title/Abstract] OR "postpartum service"[Title/Abstract] OR "antenatal service"[Title/Abstract] OR "intrapartum service"[Title/Abstract] OR "abortion service"[Title/Abstract] OR "delivery service"[Title/Abstract]) OR ("HIV testing"[Title/Abstract] OR "HIV service"[Title/Abstract] OR "HIV counseling"[Title/Abstract] OR "HIV counselling"[Title/Abstract]) OR ("family-planning"[Title/Abstract] OR "contraception"[Title/Abstract] OR "sexual-reproductive-health"[Title/Abstract] OR "gender-based violence"[Title/Abstract] OR "domestic violence"[Title/Abstract] OR "intimate-partner-violence"[Title/Abstract]) OR (("adolescent"[MeSH Terms:noexp] OR "child"[MeSH Terms:noexp] OR "infant"[MeSH Terms:noexp]) AND ("care"[Title/Abstract] OR "service"[Title/Abstract] OR "health*"[Title/Abstract] OR "wellbeing"[Title/Abstract] OR "well-being"[Title/Abstract])) | 3,880,749 | 17:17:15 |
| 20 | (adolescent[Mesh:noexp] OR child[Mesh:noexp] OR infant[Mesh:noexp]) AND (care[tiab] OR service[tiab] OR health*[tiab] OR wellbeing[tiab] OR well-being[tiab]) | | | ("adolescent"[MeSH Terms:noexp] OR "child"[MeSH Terms:noexp] OR "infant"[MeSH Terms:noexp]) AND ("care"[Title/Abstract] OR "service"[Title/Abstract] OR "health*"[Title/Abstract] OR "wellbeing"[Title/Abstract] OR "well-being"[Title/Abstract]) | 680,100 | 17:16:28 |
| 19 | family-planning[tiab] OR contraception[tiab] OR sexual-reproductive-health[tiab] OR "gender-based violence"[tiab] OR "domestic violence"[tiab] OR "intimate-partner-violence"[tiab] | | | "family-planning"[Title/Abstract] OR "contraception"[Title/Abstract] OR "sexual-reproductive-health"[Title/Abstract] OR "gender-based violence"[Title/Abstract] OR "domestic violence"[Title/Abstract] OR "intimate-partner-violence"[Title/Abstract] | 77,990 | 17:16:16 |
| 18 | "HIV testing"[tiab] OR "HIV service"[tiab] OR "HIV counseling"[tiab] OR "HIV counselling"[tiab] | | | "HIV testing"[Title/Abstract] OR "HIV service"[Title/Abstract] OR "HIV counseling"[Title/Abstract] OR "HIV counselling"[Title/Abstract] | 13,794 | 17:15:39 |
| 17 | "prenatal care"[tiab] OR "postnatal care"[tiab] OR "postpartum care"[tiab] OR "antenatal care"[tiab] OR "intrapartum care"[tiab] OR "abortion care"[tiab] OR "post-abortion care"[tiab] OR "delivery care"[tiab] OR "deliveries care"[tiab] OR "prenatal service"[tiab] OR "postnatal service"[tiab] OR "postpartum service"[tiab] OR "antenatal service"[tiab] OR "intrapartum service"[tiab] OR "abortion service"[tiab] OR "post-abortion service"[tiab] OR "delivery service"[tiab] OR "deliveries service"[tiab] | | | "prenatal care"[Title/Abstract] OR "postnatal care"[Title/Abstract] OR "postpartum care"[Title/Abstract] OR "antenatal care"[Title/Abstract] OR "intrapartum care"[Title/Abstract] OR "abortion care"[Title/Abstract] OR "post-abortion care"[Title/Abstract] OR "delivery care"[Title/Abstract] OR "prenatal service"[Title/Abstract] OR "postnatal service"[Title/Abstract] OR "postpartum service"[Title/Abstract] OR "antenatal service"[Title/Abstract] OR "intrapartum service"[Title/Abstract] OR "abortion service"[Title/Abstract] OR "delivery service"[Title/Abstract] | 27,085 | 17:15:33 |
| 16 | "Thirteen years old"[tiab] OR "fourteen years old"[tiab] OR "fifteen years old"[tiab] OR "sixteen years old"[tiab] OR "seventeen years old"[tiab] OR "eighteen years old"[tiab] OR "13 years old"[tiab] OR "14 years old"[tiab] OR "15 years old"[tiab] OR "16 years old"[tiab] OR "17 years old"[tiab] OR "18 years old"[tiab] OR "Thirteen yo"[tiab] OR "fourteen yo"[tiab] OR "fifteen yo"[tiab] OR "sixteen yo"[tiab] OR "seventeen yo"[tiab] OR "eighteen yo"[tiab] OR "13 yo"[tiab] OR "14 yo"[tiab] OR "15 yo"[tiab] OR "16 yo"[tiab] OR "17 yo"[tiab] OR "18 yo"[tiab] OR "Thirteen y.o."[tiab] OR "fourteen y.o."[tiab] OR "fifteen y.o."[tiab] OR "sixteen y.o."[tiab] OR "seventeen y.o."[tiab] OR "eighteen y.o."[tiab] OR "13 y.o."[tiab] OR "14 y.o."[tiab] OR "15 y.o."[tiab] OR "16 y.o."[tiab] OR "17 y.o."[tiab] OR "18 y.o."[tiab] | | | "Thirteen years old"[Title/Abstract] OR "fourteen years old"[Title/Abstract] OR "fifteen years old"[Title/Abstract] OR "sixteen years old"[Title/Abstract] OR "seventeen years old"[Title/Abstract] OR "eighteen years old"[Title/Abstract] OR "13 years old"[Title/Abstract] OR "14 years old"[Title/Abstract] OR "15 years old"[Title/Abstract] OR "16 years old"[Title/Abstract] OR "17 years old"[Title/Abstract] OR "18 years old"[Title/Abstract] OR "13 yo"[Title/Abstract] OR "15 yo"[Title/Abstract] OR "16 yo"[Title/Abstract] OR "17 yo"[Title/Abstract] OR "18 yo"[Title/Abstract] OR "13 y o"[Title/Abstract] OR "14 y o"[Title/Abstract] OR "15 y o"[Title/Abstract] OR "16 y o"[Title/Abstract] OR "17 y o"[Title/Abstract] OR "18 y o"[Title/Abstract] | 29,798 | 17:15:19 |
| 15 | Women[TIAB] OR woman[TIAB] OR child[TIAB] OR children[TIAB] OR infant[TIAB] OR mother[TIAB] OR maternal[TIAB] OR neonat*[TIAB] OR teen*[TIAB] OR adolescen*[TIAB] OR youth[TIAB] OR young[TIAB] OR youngster[tiab] OR newborn[tiab] OR new-born[tiab] | | | "Women"[Title/Abstract] OR "woman"[Title/Abstract] OR "child"[Title/Abstract] OR "children"[Title/Abstract] OR "infant"[Title/Abstract] OR "mother"[Title/Abstract] OR "maternal"[Title/Abstract] OR "neonat*"[Title/Abstract] OR "teen*"[Title/Abstract] OR "adolescen*"[Title/Abstract] OR "youth"[Title/Abstract] OR "young"[Title/Abstract] OR "youngster"[Title/Abstract] OR "newborn"[Title/Abstract] OR "new-born"[Title/Abstract] | 3,560,713 | 17:15:07 |
| 14 | "Women"[Mesh:noexp] OR "adolescent health" [Mesh:noexp] OR "child health"[Mesh:noexp] OR "infant health"[Mesh:noexp] OR "reproductive health"[Mesh:noexp] OR "sexual health"[Mesh:noexp] OR "child health services" [Mesh:noexp] OR "family planning services" [Mesh:noexp] | | | "Women"[MeSH Terms:noexp] OR "adolescent health"[MeSH Terms:noexp] OR "child health"[MeSH Terms:noexp] OR "infant health"[MeSH Terms:noexp] OR "reproductive health"[MeSH Terms:noexp] OR "sexual health"[MeSH Terms:noexp] OR "child health services"[MeSH Terms:noexp] OR "family planning services"[MeSH Terms:noexp] | 70,355 | 17:14:23 |
| 13 | #1 OR #3 OR 4 OR #5 OR #6 OR #7 OR #8 OR #9 OR #11 OR #12 | | | "insurance benefits"[MeSH Terms:noexp] OR "insurance coverage"[MeSH Terms:noexp] OR "prepaid health plans"[MeSH Terms:noexp] OR "insurance, health"[MeSH Terms:noexp] OR "children's health insurance program"[MeSH Terms:noexp] OR "community-based health insurance"[MeSH Terms:noexp] OR "single-payer system"[MeSH Terms:noexp] OR "insurance, health, reimbursement"[MeSH Terms] OR "Healthcare Financing"[MeSH Terms:noexp] OR "Financial mechanism"[Title/Abstract] OR "Financial strategy"[Title/Abstract] OR "Financial strategies"[Title/Abstract] OR "Financial scheme"[Title/Abstract] OR "Financial protection"[Title/Abstract] OR "Financial system"[Title/Abstract] OR "Financial policy"[Title/Abstract] OR "Financial policies"[Title/Abstract] OR "Financial reform"[Title/Abstract] OR "financial plan*"[Title/Abstract] OR "Financial statement"[Title/Abstract] OR 4[UID] OR "prepaid plan"[Title/Abstract] OR "prepaid plans"[Title/Abstract] OR "prepaid coverage"[Title/Abstract] OR "prepaid insurance"[Title/Abstract] OR "pre-paid insurance"[Title/Abstract] OR "pre-payment scheme"[Title/Abstract] OR "direct payment"[Title/Abstract] OR "insurance scheme"[Title/Abstract] OR "insurance plan"[Title/Abstract] OR "insurance plans"[Title/Abstract] OR "insurance coverage"[Title/Abstract] OR "insurance mechanism"[Title/Abstract] OR "insurance package"[Title/Abstract] OR "insurance system"[Title/Abstract] OR "social insurance"[Title/Abstract] OR "social coverage"[Title/Abstract] OR "community-based insurance"[Title/Abstract] OR "community-based coverage"[Title/Abstract] OR "health insurance"[Title/Abstract] OR "health coverage"[Title/Abstract] OR "healthcare insurance"[Title/Abstract] OR "healthcare coverage"[Title/Abstract] OR "health-care insurance"[Title/Abstract] OR "health-care coverage"[Title/Abstract] OR "out-of-pocket"[Title/Abstract] OR "out-of-pocket"[Title/Abstract] OR "OOP"[Title/Abstract] OR "subsidy"[Title/Abstract] OR "subsidies"[Title/Abstract] OR "subsidized"[Title/Abstract] OR "conditional cash transfer"[Title/Abstract] OR "conditional cash transfers"[Title/Abstract] OR "co-payment"[Title/Abstract] OR "premium*"[Title/Abstract] OR "deductible"[Title/Abstract] OR "cost-sharing"[Title/Abstract] OR "cost-sharing"[Title/Abstract] OR "capitation"[Title/Abstract] OR "taxation"[Title/Abstract] OR "tax"[Title/Abstract] OR "taxes"[Title/Abstract] OR "fee exemption"[Title/Abstract] OR "fee exempted"[Title/Abstract] OR "user-fee"[Title/Abstract] OR "user-fees"[Title/Abstract] OR "user-fees"[Title/Abstract] OR "user-fee"[Title/Abstract] OR "single-payer"[Title/Abstract] OR "fee-for-service"[Title/Abstract] OR "fee-for-service"[Title/Abstract] OR "reimburs*"[Title/Abstract] OR "voucher*"[Title/Abstract] OR "donor funding"[Title/Abstract] OR "donor support"[Title/Abstract] OR "donor pool"[Title/Abstract] OR "donor contribution"[Title/Abstract] OR "donor's pool"[Title/Abstract] OR "donor's contribution"[Title/Abstract] OR "donors pool"[Title/Abstract] OR "fees exemption"[Title/Abstract] OR "fee reduction"[Title/Abstract] OR "fee removal"[Title/Abstract] OR "fees removal"[Title/Abstract] OR "official development assistance"[Title/Abstract] OR "ODA"[Title/Abstract] OR "health-benefit-package"[Title/Abstract] OR "healthcare-benefit-package"[Title/Abstract] OR "performance-based-financing"[Title/Abstract] OR "government fund"[Title/Abstract] OR "health-equity-fund"[Title/Abstract] OR "financial coverage"[Title/Abstract] | 193,807 | 9:02:21 |
| 12 | "fees exemption"[tiab] OR "exempted from fees"[tiab] OR "reduction of fee"[tiab] OR "fee reduction"[tiab] OR "fees reduction"[tiab] OR "fee removal"[tiab] OR "fees removal"[tiab] OR "removal of fees"[tiab] OR "reducing fee"[tiab] OR "official development assistance"[tiab] OR "ODA"[tiab] OR health-benefit-package[tiab] OR healthcare-benefit-package[tiab] OR "health-care benefit-package"[tiab] OR "performance-based-financing"[tiab] OR "government fund"[tiab] OR "governmental fund"[tiab] OR "health-equity-fund"[tiab] OR "healthcare-equity-fund"[tiab] OR "health-care equity-fund"[tiab] OR "financial coverage"[tiab] | | | "fees exemption"[Title/Abstract] OR "fee reduction"[Title/Abstract] OR "fee removal"[Title/Abstract] OR "fees removal"[Title/Abstract] OR "official development assistance"[Title/Abstract] OR "ODA"[Title/Abstract] OR "health-benefit-package"[Title/Abstract] OR "healthcare-benefit-package"[Title/Abstract] OR "performance-based-financing"[Title/Abstract] OR "government fund"[Title/Abstract] OR "health-equity-fund"[Title/Abstract] OR "financial coverage"[Title/Abstract] | 1,538 | 9:00:13 |
| 11 | "donor funding"[tiab] OR "donor support"[tiab] OR "donor pool"[tiab] OR "donor contribution"[tiab] OR "donor’s funding"[tiab] OR "donor’s support"[tiab] OR "donor’s pool"[tiab] OR "donor’s contribution"[tiab] OR "donors’ funding"[tiab] OR "donors’ support"[tiab] OR "donors’ pool"[tiab] OR "donors’ contribution"[tiab] | | | "donor funding"[Title/Abstract] OR "donor support"[Title/Abstract] OR "donor pool"[Title/Abstract] OR "donor contribution"[Title/Abstract] OR "donor's pool"[Title/Abstract] OR "donor's contribution"[Title/Abstract] OR "donors pool"[Title/Abstract] | 2,817 | 8:52:35 |
| 9 | out-of-pocket[tiab] OR "out of pocket"[tiab] OR OOP[tiab] OR subsidy[tiab] OR subsidies[tiab] OR subsidized[tiab] OR "conditional cash transfer"[tiab] OR "conditional cash transfers"[tiab] OR co-payment[tiab] OR premium*[tiab] OR deductible[tiab] OR "cost sharing"[tiab] OR cost-sharing[tiab] OR capitation[tiab] OR taxation[tiab] OR tax[tiab] OR taxes[tiab] OR "fee exemption"[tiab] OR "fee exempted"[tiab] OR "exempted from fee"[tiab] OR user-fee[tiab] OR user-fees[tiab] OR "user fees"[tiab] OR "user fee"[tiab] OR single-payer[tiab] OR fee-for-service[tiab] OR "fee for service"[tiab] OR reimburs*[tiab] OR voucher*[tiab] | | | "out-of-pocket"[Title/Abstract] OR "out-of-pocket"[Title/Abstract] OR "OOP"[Title/Abstract] OR "subsidy"[Title/Abstract] OR "subsidies"[Title/Abstract] OR "subsidized"[Title/Abstract] OR "conditional cash transfer"[Title/Abstract] OR "conditional cash transfers"[Title/Abstract] OR "co-payment"[Title/Abstract] OR "premium*"[Title/Abstract] OR "deductible"[Title/Abstract] OR "cost-sharing"[Title/Abstract] OR "cost-sharing"[Title/Abstract] OR "capitation"[Title/Abstract] OR "taxation"[Title/Abstract] OR "tax"[Title/Abstract] OR "taxes"[Title/Abstract] OR "fee exemption"[Title/Abstract] OR "fee exempted"[Title/Abstract] OR "user-fee"[Title/Abstract] OR "user-fees"[Title/Abstract] OR "user-fees"[Title/Abstract] OR "user-fee"[Title/Abstract] OR "single-payer"[Title/Abstract] OR "fee-for-service"[Title/Abstract] OR "fee-for-service"[Title/Abstract] OR "reimburs*"[Title/Abstract] OR "voucher*"[Title/Abstract] | 74,804 | 8:51:52 |
| 8 | "social insurance"[tiab] OR "social coverage"[tiab] OR "community-based insurance"[tiab] OR "community-based coverage"[tiab] OR "health insurance"[tiab] OR "health coverage"[tiab] OR "healthcare insurance"[tiab] OR "healthcare coverage"[tiab] OR "health-care insurance"[tiab] OR "health-care coverage"[tiab] | | | "social insurance"[Title/Abstract] OR "social coverage"[Title/Abstract] OR "community-based insurance"[Title/Abstract] OR "community-based coverage"[Title/Abstract] OR "health insurance"[Title/Abstract] OR "health coverage"[Title/Abstract] OR "healthcare insurance"[Title/Abstract] OR "healthcare coverage"[Title/Abstract] OR "health-care insurance"[Title/Abstract] OR "health-care coverage"[Title/Abstract] | 53,262 | 8:51:43 |
| 7 | "insurance scheme"[tiab] OR "insurance plan"[tiab] OR "insurance plans"[tiab] OR "insurance coverage"[tiab] OR "insurance mechanism"[tiab] OR "insurance package"[tiab] OR "insurance system"[tiab] | | | "insurance scheme"[Title/Abstract] OR "insurance plan"[Title/Abstract] OR "insurance plans"[Title/Abstract] OR "insurance coverage"[Title/Abstract] OR "insurance mechanism"[Title/Abstract] OR "insurance package"[Title/Abstract] OR "insurance system"[Title/Abstract] | 15,727 | 8:51:34 |
| 6 | "targeted payment"[tiab] OR "direct payment"[tiab] | | | "direct payment"[Title/Abstract] | 97 | 8:51:28 |
| 5 | "prepaid scheme"[tiab] OR "prepaid plan"[tiab] OR "prepaid plans"[tiab] OR "prepaid coverage"[tiab] OR "prepaid insurance"[tiab] OR "prepaid package"[tiab] OR "pre-paid scheme"[tiab] OR "pre-paid plan"[tiab] OR "pre-paid plans"[tiab] OR "pre-paid coverage"[tiab] OR "pre-paid insurance"[tiab] OR "pre-paid package"[tiab] OR "pre-payment scheme"[tiab] OR "pre-payment plan"[tiab] OR "pre-payment plans"[tiab] OR "pre-payment coverage"[tiab] OR "pre-payment insurance"[tiab] OR "pre-payment package"[tiab] OR "pre-payments scheme"[tiab] OR "pre-payments plan"[tiab] OR "pre-payments plans"[tiab] OR "pre-payments coverage"[tiab] OR "pre-payments insurance"[tiab] OR "pre-payments package"[tiab] | | | "prepaid plan"[Title/Abstract] OR "prepaid plans"[Title/Abstract] OR "prepaid coverage"[Title/Abstract] OR "prepaid insurance"[Title/Abstract] OR "pre-paid insurance"[Title/Abstract] OR "pre-payment scheme"[Title/Abstract] | 117 | 8:50:59 |
| 4 | "Payment mechanism"[tiab] OR "Payment strategy"[tiab] OR "Payment strategies"[tiab] OR "Payment scheme"[tiab] OR "Payment method"[tiab] OR "Payment protection" [tiab] OR "Payment system"[tiab] OR "Payment policy"[tiab] OR "Payment policies"[tiab] OR "Payment reform"[tiab] OR "Payment plan*"[tiab] | | | "Payment mechanism"[Title/Abstract] OR "Payment strategy"[Title/Abstract] OR "Payment strategies"[Title/Abstract] OR "Payment scheme"[Title/Abstract] OR "Payment method"[Title/Abstract] OR "Payment system"[Title/Abstract] OR "Payment policy"[Title/Abstract] OR "Payment policies"[Title/Abstract] OR "Payment reform"[Title/Abstract] OR "payment plan*"[Title/Abstract] | 5,244 | 8:50:46 |

| 3 | "Financial mechanism"[tiab] OR "Financial strategy" [tiab] OR "Financial strategies" [tiab] OR "Financial scheme"[tiab] OR "Financial method"[tiab] OR "Financial protection" [tiab] OR "Financial system"[tiab] OR "Financial policy"[tiab] OR "Financial policies"[tiab] OR "Financial reform"[tiab] OR "Financial plan*"[tiab] OR "Financial statement"[tiab] | "Financial mechanism"[Title/Abstract] OR "Financial strategy"[Title/Abstract] OR "Financial strategies"[Title/Abstract] OR "Financial scheme"[Title/Abstract] OR "Financial protection"[Title/Abstract] OR "Financial system"[Title/Abstract] OR "Financial policy"[Title/Abstract] OR "Financial policies"[Title/Abstract] OR "Financial reform"[Title/Abstract] OR "financial plan*"[Title/Abstract] OR "Financial statement"[Title/Abstract] | 1,701 | 8:50:12 |
| --- | --- | --- | --- | --- |
| 1 | "insurance benefits"[Mesh:noexp] OR "insurance coverage" [Mesh:noexp] OR "prepaid health plans" [Mesh:noexp] OR "insurance, health" [Mesh:noexp] OR "children's health insurance program" [Mesh:noexp] OR "community-based health insurance" [Mesh:noexp] OR "single-payer system" [Mesh:noexp] OR "insurance, health, reimbursement" [Mesh] OR "Healthcare Financing"[Mesh:noexp] | "insurance benefits"[MeSH Terms:noexp] OR "insurance coverage"[MeSH Terms:noexp] OR "prepaid health plans"[MeSH Terms:noexp] OR "insurance, health"[MeSH Terms:noexp] OR "children's health insurance program"[MeSH Terms:noexp] OR "community-based health insurance"[MeSH Terms:noexp] OR "single-payer system"[MeSH Terms:noexp] OR "insurance, health, reimbursement"[MeSH Terms] OR "Healthcare Financing"[MeSH Terms:noexp] | 93,169 | 8:49:19 |

**Grey literature search**

**GAVI**

“social exclusion” OR “social inclusion” OR inequity OR inequality OR equity OR equality OR poverty OR refugees OR minority OR minorities OR homosexuality OR rural OR slum OR disparity OR disparities OR discrimination

[https://www.gavi.org/search?s=+%25E2%2580%259C+social+exclusion%25E2%2580%259D+OR+%25E2%2580%259Csocial+inclusion%25E2%2580%259D+OR+inequity+OR+inequality+OR+equity+OR+equality+OR+poverty+OR+refugees+OR+minority++O](https://st1.zoom.us/web_client/in43ub/html/externalLinkPage.html?ref=https://www.gavi.org/search?s=+%2525E2%252580%25259C+social+exclusion%2525E2%252580%25259D+OR+%2525E2%252580%25259Csocial+inclusion%2525E2%252580%25259D+OR+inequity+OR+inequality+OR+equity+OR+equality+OR+poverty+OR+refugees+OR+minority++O)

**UNHCR**

[https://www.unhcr.org/search?query=equity](https://st1.zoom.us/web_client/in43ub/html/externalLinkPage.html?ref=https://www.unhcr.org/search?query=equity)

<https://www.unhcr.org/search?query=inequity>

**WORLD BANK**

(“social exclusion” OR “social inclusion” OR inequity OR inequality OR equity OR equality OR minority OR minorities OR homosexuality OR rural OR slum OR disparity OR disparities OR discrimination) AND (women OR child OR children OR woman OR adolescent OR adolescence OR maternal OR mother OR new-born OR infant OR infancy) AND (financial OR financing OR monetary OR fiscal)

[https://openknowledge.worldbank.org/discover?scope=%252F&query=%2528%25E2%2580%259Csocial+exclusion%25E2%2580%259D+OR+%25E2%2580%259Csocial+inclusion%25E2%2580%259D+OR+inequity+OR+inequality+OR+equity+OR+equality+OR+minority++OR+minorities+OR+homosexuality+OR+rural+OR+slum+OR+disparity+OR+disparities+OR+discrimination%2529+AND+%2528women+OR+child+OR+children+OR+woman+OR+adolescent+OR+adolescence+OR+maternal+OR+mother+OR+new-born+OR+infant+OR+infancy%2529++AND+%2528financial+OR+financing++OR+monetary+OR+fiscal%2529&submit=](https://st1.zoom.us/web_client/in43ub/html/externalLinkPage.html?ref=https://openknowledge.worldbank.org/discover?scope=%25252F&query=%252528%2525E2%252580%25259Csocial+exclusion%2525E2%252580%25259D+OR+%2525E2%252580%25259Csocial+inclusion%2525E2%252580%25259D+OR+inequity+OR+inequality+OR+equity+OR+equality+OR+minority++OR+minorities+OR+homosexuality+OR+rural+OR+slum+OR+disparity+OR+disparities+OR+discrimination%252529+AND+%252528women+OR+child+OR+children+OR+woman+OR+adolescent+OR+adolescence+OR+maternal+OR+mother+OR+new-born+OR+infant+OR+infancy%252529++AND+%252528financial+OR+financing++OR+monetary+OR+fiscal%252529&submit=)
