# Supplementary material for: Financing for equity for women’s, children’s and adolescents’ health in low- and middle-income countries: A scoping review
Source: PLOS Glob Public Health. 2024 Sep 12;4(9):e0003573. doi: 10.1371/journal.pgph.0003573 (PMC11392393; doi:10.1371/journal.pgph.0003573)
Supplement: S3 Appendix — (DOCX) [file pgph.0003573.s003.docx]

**S3 Appendix**

**Composite Humanitarian and Fragile Settings Classification**

The humanitarian and fragile settings classification included in the review is based on the triangulation of data from four humanitarian and fragile states classifications: [OECD](https://www.oecd.org/dac/states-of-fragility-fa5a6770-en.htm), [The World Bank 2021 Harmonized list of fragile situations](https://pubdocs.worldbank.org/en/888211594267968803/FCSList-FY21.pdf); [Fragile State Index 2020](https://fragilestatesindex.org/), the [INFORM Severity Index](https://drmkc.jrc.ec.europa.eu/inform-index/INFORM-Severity), as well as [UNOCHA data on humanitarian response plans](https://reliefweb.int/report/world/global-humanitarian-overview-2021-enarfres). The classification includes four categories – highest fragility, very high fragility, high fragility and fragile settings (please see below tables).

| **Highest Fragility** | **Very High Fragility** | **High Fragility** | **Fragile Setting** |
| --- | --- | --- | --- |
| **Afghanistan** | **Burundi** | **Burkina Faso** | **Bangladesh** |
| **Somalia** | **CAR** | **Cameroon** | **Colombia** |
| **Sudan** | **Chad** | **Congo,Rep** | **Comoros** |
| **Syria** | **DRC** | **DPPK** | **Cote D'ivoire** |
| **Yemen** | **Haiti** | **Eritrea** | **Djibouti** |
| **South Sudan** | **Libya** | **Ethiopia** | **Eswatini** |
|  | **Nigeria** | **Mali** | **Gambia** |
|  | **Venezuela** | **Mozambique** | **Guatemala** |
|  |  | **Myanmar** | **Guinea** |
|  |  | **Niger** | **Guinea-Bissau** |
|  |  | **Pakistan** | **Honduras** |
|  |  | **State of Palestine** | **Kenya** |
|  |  | **Zimbabwe** | **Lao PDR** |
|  |  |  | **Liberia** |
|  |  |  | **Madagascar** |
|  |  |  | **Mauritania** |
|  |  |  | **Papa New guinea** |
|  |  |  | **Solomon Islands** |
|  |  |  | **Timor-Leste** |
|  |  |  | **Uganda** |
|  |  |  | **Ukraine** |
|  |  |  | **Zambia** |
