# Supplementary material for: Financing for equity for women’s, children’s and adolescents’ health in low- and middle-income countries: A scoping review
Source: PLOS Glob Public Health. 2024 Sep 12;4(9):e0003573. doi: 10.1371/journal.pgph.0003573 (PMC11392393; doi:10.1371/journal.pgph.0003573)
Supplement: S1 Table — (DOCX) [file pgph.0003573.s004.docx]

| **Author Year** | **Country** | **Study design** | **Health service covered** | **Target group and PROGRESS Plus measures** | **Outcome(s)** | **Main Results**  **(yes/no/inconclusive)** |
| --- | --- | --- | --- | --- | --- | --- |
| Randive 2013 | India | Observational (Survey) | Health facility delivery & obstetric care | **Target group:** Pregnant women  **PROGRESS Plus Measure:** Socioeconomic status (SES) | Healthcare Utilization  Mortality | The conditional cash transfer program resulted in significant improvement in institutional delivery  **Positive impact**  **No impact** on mortality outcomes |
| Lim 2010 | India | Observational (Survey) | Antenatal care  In-facility births | **Target group:** Women and children  **PROGRESS Plus Measure:** Socioeconomic status (SES) | Healthcare Utilization  Mortality | The conditional cash transfer program resulted in a significant increase in antenatal care and in-facility births.  **Positive impact**  The conditional cash transfer program resulted in a significant reduction in perinatal and neonatal death  **Positive impact** |
| De Brauw 2020 | Brazil | Quasi experimental  (Mixed ecological design) | Maternal care | **Target group:** Women  **PROGRESS Plus Measure:** Socioeconomic status (SES) | Healthcare Utilization  Mortality | The conditional cash transfer program led to a reduction in barriers to service utilization including prenatal care, skilled attendance, and facility births  **Positive impact**  The program improved tetanus toxoid vaccination for mothers and low birth weight children  **Positive impact**  No significant result on child and maternal mortality  **No impact** |
| Rasella 2013 | Brazil | Quasi experimental  (Mixed ecological design) | Children Vaccination, Child health check-up and growth monitoring, prenatal and postnatal visits, health and nutritional education activities | **Target group:** children up to 17 years of age or pregnant or lactating women.5  **PROGRESS Plus Measure:** Socioeconomic status (SES) | Healthcare Utilization  Mortality | The program resulted in an increased vaccination coverage and a reduction in hospital admissions  **Positive impact**  The conditional cash transfer program led to a significant reduction of under-5 mortality, overall and from poverty-related causes including malnutrition and diarrhea  **Positive impact** |
| Rasella 2021 | Brazil | Observational (Retrospective cohort) | Prenatal care | **Target group:** Pregnant women and children  **PROGRESS Plus Measure:** Socioeconomic status (SES) | Healthcare Utilization  Mortality | The conditional cash transfer program improved healthcare-related variables, including the number of physicians and private care coverage  **Positive impact**  The conditional cash transfer program resulted in a significant reduction in maternal mortality ratio for pregnant women younger than 30 years of age  **Positive impact** |
| Shei 2013 | Brazil | Observational (Cross-sectional) | Preventive care | **Target group:** Children under 7  **PROGRESS Plus Measure:** Socioeconomic status (SES) | Mortality | The conditional cash transfer program resulted in a significant reduction in  infant mortality, especially during the post neonatal period.  **Positive impact** |
| Ramos 2021 | Brazil | Observational (Cross-sectional) | not specified | **Target population:**  low income families  (In this study children aged 1–4  **Progress plus:**  socio-economic status, ethnicity | Mortality | Receiving a Bolsa Family Program (BPF) stipend is significantly associated with a reduced probability of death between the ages 1 and 4 years. This association was stronger for preterm children, children of Black mothers, children living in poorer municipalities, and children living in municipalities with better indexes of BFP management  **positive impact** |
| Poirier, M. J. P., 2020 | Colombia,  Ecuador, Peru, and Bolivia | Descriptive (Case series) | Vaccination | **Target group:** Pregnant women who report not having social protection coverage  **PROGRESS Plus Measure:** Socioeconomic status (SES), Place of residence | Implementation considerations | Household SES was found to be an important driver of malnutrition and mortality compared to geography in every country and year  **Inconclusive** |
| De Souza 2021 | Brazil | Observational Prospective Cohort | Children health services | **Target group:** Children less than 5  **PROGRESS Plus Measure:** Socioeconomic status (SES) | Mortality | The CCT program adequate access to water, sanitation and solid waste collection resulted in a reduction in child mortality rates due to malnutrition and diarrhea  **Positive impact** |
| Glassman 2013 | Low-middle income countries | Systematic review | Maternal and newborn health | **Target group:** Women and children  **PROGRESS Plus Measure:** Socioeconomic status (SES) | Healthcare Utilization  Mortality  Child development | The program resulted in a significant Increase in adequate prenatal monitoring  **Positive impact**  The conditional cash transfer program resulted in a significant reduction in maternal mortality (11% decline)  **Positive impact**  The program resulted in a small but significant decline in the incidence of low birthweight  **Positive impact** |
| Fernald 2008  Role of cash in conditional cash transfer | Mexico | Observational Prospective cohort | Preventive care, preventive health requirements, nutrition supplementation, education, and monitoring | **Target group: children**  **PROGRESS Plus Measure:** Socioeconomic Status (SES) | Child development | When doubling the cumulative cash transfers, the intervention was associated with an increase in height-for-age Z score and a lower prevalence of stunting  **Positive impact** |
| Andersen 2015 | Peru | Observational  Secondary data analysis | Regular healthcare visits | **Target group:** Children between 7-8 years old  **PROGRESS Plus Measure:** Place of residence and socioeconomic status (SES) | Child development | Enrollment in the conditional cash transfer program was not associated with any change in Height for age z score  **No impact** |
| Paes-Sousa 2011 | Brazil | Observational (Cross-sectional surveys) | Nutrition services | **Target group:** Children  **PROGRESS Plus Measure:** Socioeconomic Status (SES) | Child development | Children from families exposed to the program were more likely to have normal height for age and weight for age compared to non-exposed families, however results were not signicant for weight for height.  **Inconclusive** |
| Barber 2008 | Mexico | Experimental  (RCT) | Regular clinic consultation, pre-natal visits, monitoring the pregnancy’s progression; and the prevention, detection, and control of obstetric and perinatal risk factor | **Target group:** Pregnant women  **PROGRESS Plus Measure:** Place of residence | Child development | The intervention was shown to improve birthweight outcomes including a higher birthweight among participating women and a reduction in the percentage of low birthweight  **Positive impact** |
| Leroy 2008 | Mexico | Observational Survey | Immunization of children, growth monitoring for children 0–60 mo of age, and prenatal and postpartum care visits | **Target group:** Children  **PROGRESS Plus Measure:** socioeconomic status and place of residence | Child development | The program was associated with an increase in length gain in children 0-12 month of age, though not significant. Gains in length were mostly observed for children from most vulnerable families  **Positive Impact** |
| Fernald 2009 | Mexico | Observational  Prospective cohort | Preventive medical care for family members | **Target group:** School aged children  **PROGRESS Plus Measure:** Socioeconomic Status (SES) | Child development | No differences were observed between the intervention and control group for mean height-for-age Z scores, BMI-for-age Z scores  **No impact** |
| Lucas 2022 | Brazil | Observational secondary data analysis | Child maternal | **Target population:**  poor and extremely poor households in Brazil that have a pregnant woman or a child.  **Progress plus:**  Socioeconomic status | Child development | Children born in households where the mother received BF aid were less likely to have low birth weight, very low birth weight, as well as to be born after 37 weeks of gestation (hereinafter PTB) or 28 weeks of gestation (hereinafter VPTB). There was no significant effect on congenital malformation  Our results **suggest a potential positive intergenerational effect of the BFP**. On average, the higher the probability that the grandmother received BF aid, the lower was the prevalence of low birth weight, very low birth weight and congenital malformation of the newborn. No trend was noted for preterm birth. At the same time, the obtainment of BF support by the mother showed a positive correlation with the health of the newborn.  **Positive impact/ no impact congenital malformation** |
| Leroy 2021 | Mexico, Colombia, Uruguay, India  Colombia  Uruguay  India | Systematic review | basic preventive health-care intervention  birth in public or accredited private health facilities, ANC services | **Low-income HHs, women, children**  place of Residence & socio-economic status, age | Mortality  Child development  Quality of care | The **Uruguayan program** did not have a positive effect on ANC seeking but increased public hospital delivery by 3.1 pp  In Colombia, the impact of the program on birth weight was limited to urban areas  Progresa had a positive effect on birth weight, ranging from 102 g. the program almost halved the prevalence of low birth weight  **Positive impact**  PANES in *Uruguay* was found to have a positive effect on birth weight of 31 g. equivalent to a 20% reduction of the preprogram prevalence. the authors concluded that the effect on birth weight must be driven by improvements in maternal nutrition during pregnancy due to the cash transfers.  **Positive impact**  The authors reported that the program had a positive effect on the quality of the care received. The assessment of quality, however, was based on mothers’ recall of the different procedures received. The authors concluded that the impact on birth weight was due to the higher quality of the prenatal care received, which was in turn a consequence of empowering women to negotiate better care from health-care providers  **Positive impact** |
| Oduenyi 2019 | Nigeria | Observational Retrospective cohort | Maternal, newborn, and child health (MNCH) | **Target group:**  Pregnant women, children  **PROGRESS Plus Measure:** Socioeconomic status (SES) | Mortality  Other outcome  *Health Seeking Behavior*  Implementation considerations | The intervention was believed to improve the health status of the communities, including significantly reducing maternal and infant deaths.  **Positive Impact**  The intervention resulted in an increased demand for maternal and child health services in the facilities and ultimately stimulated changes in health‐seeking behaviours of their clients.  **Positive Impact**  97.9% of participants reported that the cash incentive was very helpful to them with the majority (59.2%) reporting that they used the cash incentives to assist their families or subsidize transportation costs to the PHC.  **Positive Impact** |
| Lagarde 2009 | Low middle income countries | Systematic review | N/A | **Target group:** Women, children, pregnant women  **PROGRESS Plus Measure:** Socioeconomic Status (SES) | Healthcare utilization  Morbidity  Child development | The intervention resulted in an increase in daily outpatient visits to health facilities, a significant increase in the use of services for pre-school children but a non-significant increase in the uptake of antenatal care or 10-day postnatal checkups.  **Inconclusive**  The intervention resulted in a decrease in stunting and an average improvement of the height-for-age and the proportion of under-weight children aged 0 to 5 years old  **Positive impact**  The intervention did not have any impact on the proportion of wasted children aged 0 to 5 years old.  **No impact**  A positive effect of the intervention was shown on the nutritional status for children under 24 months including newborn weight. However, no impact was shown on the nutritional status of children older than 24 months, or on newborn weight in rural areas.  **Inconclusive** |
|  |  |  |  |  |  |  |
| Onwuchekwa 2021 | sub-Saharan Africa | Systematic review | Child Health | **Target population:**  Children  **Progress plus:**  Age | Healthcare utilization  Morbidity  Nutrition | The current body of evidence suggests that CCTs may only result in an increase in health service utilisation in the short term with the incentive value of the transferred cash diminishing over time. This might explain the observation in the TASAF programme in Tanzania, where a significant increase in clinic visits was seen at 1.5 years after initiation, which disappeared afterwards  **unsustainable impact**  Overall, this review found that there is no evidence that demonstrates a positive effect of CCT on the illness among children in sub-Saharan Africa.  The evidence of improved health service use and nutritional status is inconsistent between studies. Despite indications that the extra household income from CCT is mostly used for improving the household diet, this has not consistently translated into  better nutrition for children in SSA  **no impact**  **positive nutritional impact** was observed when cash transfers were  given to households experiencing sudden and profound food  insecurity, as shown in the emergency programme in Niger |
| Okeke 2020 | Nigeria | Experimental RCT | Prenatal, delivery, and postnatal care | **Target group:** Pregnant women  **PROGRESS Plus Measure:** Place of residence | Healthcare utilization  Morbidity  Quality of care | an increase in the number of facility deliveries among beneficiaries (41%).  **Positive impact**  No impact of the intervention was noticed in terms of preventing complications that could lead to maternal deaths.  **No impact**  An improvement in the quality of delivery of care and in overall satisfaction with care  **Positive impact** |
| Cruz, 2017 | Low-and middle-income countries | literature review | Vaccination and others health services | **Target group:** Children under 5 years old  **PROGRESS Plus Measure:** Socioeconomic status (SES) | Healthcare utilization  Mortality  Morbidity | Studies that used immunization rates or vaccination resulted in a significant increase in childhood immunization rates  **Positive impact**  Several conditional cash transfer programs reported a reduction of infant, perinatal and postnatal mortality  **Positive impact**  Conditional cash transfer program was shown to improve child morbidity whereby the incidence of diarrhea and acute respiratory infections has been reduced  **Positive impact** |
| Owusu-Addo 2016 | Ghana | Observational (Qualitative) |  | **Target group:** Orphans and vulnerable children in rural Ghana  **PROGRESS Plus Measure:** Socioeconomic status (SES) and place of residence | Healthcare Utilization  Mortality  Morbidity | Across diverse settings, the intervention resulted in an increase in the use of health services by children  **Positive impact**  The intervention resulted in an increase in institutional delivery among eligible participants  **Positive impact**  The intervention resulted in a reduction in mortality,  The intervention resulted in a reduction anemia and stunting among vulnerable children in Latin America.  **Positive impact** |
| Moncayo 2019 | Ecaudor | Observational Retrospective cohort |  | **Target group:** children under 18  **PROGRESS Plus Measure:** Socioeconomic status (SES) | Mortality  Morbidity | The conditional cash transfer program resulted in a decrease in the mean under-5 mortality rate from 15.2 to 12.9 per 1000 live births from 2009 to 2014.  **Positive Impact**  The intervention resulted in a 79% decline in the U5MR attributed to diarrheal diseases  **Positive impact**  The intervention resulted in reduced hospitalization due to diarrheal diseases and malnutrition decreased by 1.9% and 27.6%, respectively.  **Positive Impact** |
| Neves 2020 | Brazil | literature review | Immunisation, prenatal care, growth monitoring and child development. | **Target group:** Mothers, children between the ages of zero to 12 years or adolescents up to the age of 17 years  **PROGRESS Plus Measure:** Socioeconomic status (SES) | Mortality | The Brazilian conditional cash transfer problem resulted in a reduction in child mortality rates, suicide rates, and tuberculosis mortality.  **Positive impact** |
| Ziebold 2021 | Brazil | Observational retrospective cohort | maternal and child services | **Target population:**  pregnant women and Poor families with children aged 0-6 years or children 0-17 years enrolled in school  **Progress plus:**  socio-economic | Morbidity | The intervention is not associated with worsened or improved externalising mental health problems and socio-emotional competencies among low-income adolescents living in Pelo  **Inconclusive outcome** |
| Fernald 2008  (Cash component of conditional cash transfer program is associated with higher body mass index and blood pressure in adults) | Mexico | Experimental (RCTs) | Preventive medical care | **Target group:** women Adults aged 18–65 y  **PROGRESS Plus Measure:** Socioeconomic Status (SES) and place of residence | Morbidity | Doubling of cumulative cash transfers to the household was associated with higher BMI, higher diastolic blood pressure, and higher prevalence of overweight, grade I obesity, and grade II obesity.  **Negative impact** |
| Pettifor 2016 | South Africa | Experimental RCTs | HIV care, HIV testing | Females aged 13–20 years, enrolled in grades 8–11  within participating public schools, not married or pregnant  **PROGRESS Plus Measure:** Socioeconomic Status (SES) and place of residence | Morbidity | Cash transfers that address structural factors such as schooling and poverty have the potential to reduce HIV risk in young women in South Africa. |
| Morris 2004 (2)  Conditional cash transfers are associated with a Small reduction in the rate of weight gain of preschool children in Northeast Brazil | Brazil | Experimental RCT | Child health services | **Target group:** Children and Women  **PROGRESS Plus Measure:** Socioeconomic Status (SES) | Child development | The intervention resulted in a reduction in weight gain throughout months of exposure whereas children experienced a 274 g less weight gained over a 6-mo period.  **Positive Impact** |
| Forde 2012 | Colombia | Observational  Prospective cohort | Immunization and health check ups | **Target group:** Children  **PROGRESS Plus Measure:** Socioeconomic Status (SES) | Morbidity | The study resulted in an increase in women’s BMI and odds of obesity  **Negative impact** |
| Gertler 2004 | Mexico | Experimental  RCTs | Immunization, nutrition monitoring, nutrition supplementation, growth monitoring, prenatal care, health education for mothers, physical check-ups | **Target group:** Children aged 0-23 month, children aged 24-60 month , pregnant women and other family members  **PROGRESS Plus Measure:** Place of residence | Child development  Morbidity | improving height-for-age z scores, length and stunting among children  The intervention resulted in a reduction in illness rate and a reduction in anemia among children enrolled in the program  **Positive impact** |
| Perez-Lu 2017 | Peru | Observational Survey | Antenatal care visits, child check up | **Target group:** Women and children  **PROGRESS Plus Measure:** socioeconomic status and place of residence | Morbidity | Enrollment in the intervention resulted in a lower prevalence of underweight among women and anemia among children. The analysis resulted in a lower prevalence of overweight for women, and acute malnutrition in children. Nevertheless, residence in the district of program implementation was associated with an increase in the prevalence of anemia in children  **Positive impact** |
| Calvasina 2018 | Brazil | Observational Survey | Immunization, regular health check-ups, and growth monitoring | **Target group:** Children younger than 7 years of age  **PROGRESS Plus Measure:** Socioeconomic status (SES) | Morbidity | The conditional cash transfer program resulted in lower odds of children having dental caries mainly among extremely poor families  **Positive impact** |
| Lopez-Arana 2016 | colombia | Observational  (Secondary data analysis) | Vaccination, growth, and development check-ups | **Target group:** Children up to 17 years of age  **PROGRESS Plus Measure:** Socioeconomic Status (SES) | Child development | The study resulted in an increase in the mean heigh-for-age z score and a decrease in the prevalence of stunting in both the intervention and control group.  **Positive Impact** |
| Prencipe 2021 | Tanzania | Experimental (RCT) | health services in general | **Target population:**  the poorest 10% of the population  (youth)  **Progress plus:**  socio-economic status, gender, age | Morbidity  Implementation considerations | This intervention positively impacted the mental health of young males and  negatively impacted young females, particularly older adolescent girls/young women. CCT programs provided to households can improve the mental health of young males but may negatively impact females.  **Positive for males negative for females**  conditions may increase women’s time poverty and contribute to reduced mental health  Some suggestions to mitigate the potential burdens include providing childcare mechanisms and other support systems for beneficiaries, conducting community sessions to address gender inequities and stereotypes, and removing conditions (i.e., making the CTs unconditional) |
| Randive 2014 | India | Observational (Secondary data analysis) | Delivery care | **Target group:** Pregnant Women  **PROGRESS Plus Measure:** Socioeconomic status (SES) | Healthcare utilization  Mortality | The study revealed a disproportionate lower concentration of institutional deliveries in poor areas than in rich areas  **Inconclusive**  Fewer maternal death was observed in the richest divisions compared to the poorest division. The study revealed increased inequalities because of a lower decline in maternal mortality rate in the poorer divisions  **Positive impact** |
| Adato 2009 | Many countries | Literature review | Maternal and child services: antenatal care, prenatal care, immunization, check-ups. | **Target group:**  vulnerable children and families  **PROGRESS Plus Measure:** Socioeconomic status (SES) | Healthcare Utilization  Mortality | The interventions resulted in an increase in children’s health check, growth monitoring visits and well-child visits  **Positive impact**  The intervention resulted in 11% decline in maternal mortality and a 2% decline in infant mortality, with the stronger impacts in the poorer municipalities  **Positive impact** |
| Powell-Jackson 2015 | India | Observational Survey | Maternal health services | **Target group:**  Pregnant women  **PROGRESS Plus Measure:** Socioeconomic status (SES) | Healthcare utilization  Mortality | The intervention resulted in an increase in the proportion of women delivering with the attendance of a health worker  **Positive impact**  The intervention resulted in a decrease in the percentage of women going into caesarean section  **Negative**  The intervention resulted in a a 7.5 percentage point increase in facility births and in an 11-percentage point increase in public facility births.  **Positive impact**  The intervention did not have any impact on the utilisation of antenatal care services  **No impact**  The intervention did not result in a strong effect on neonatal mortality  **No impact** |
| Barber 2009 | Mexico | Observational Survey | Prenatal Care with emphasis on pregnancy’s progression; and the prevention, detection, and control of obstetric and perinatal risk factors & nutrition supplementation & Educational and programmatic meeting | **Target group:** Women of Reproductive Age  **PROGRESS Plus Measure:** Socioeconomic Status (SES) | Healthcare utilization | Beneficiaries were shown to receive a higher number of prenatal procedures related to history taking, diagnostics, physical examination and prevention and case-management services compared with non-beneficiaries.  **Positive Impact** |
| Lahariya 2011 | India | Quasi-experimental (Controlled before and after) | The condition for the cash incentives: Institutional delivery, caesarean section, compensation in case of death during delivery for the incentive program | **Target group:** Women of reproductive age  **PROGRESS Plus Measure:** Socioeconomic Status (SES) | Healthcare utilization  Implementation considerations | The conditional cash transfer program resulted in an increased in the proportion of institutional deliveries, however resulted were the lowest among the poorest population  **Positive impact**  Lack of awareness, cumbersome cash disbursement procedure, intricate eligibility criteria, extensive paper work, and insufficient focus on community involvement |
| Barber 2010 | Mexico | Experimental (RCT) | Prenatal care, nutrition supplementation, nutrition, and health education | **Target group:** Pregnant women  **PROGRESS Plus Measure:** Socioeconomic Status (SES) | Healthcare utilization | The conditional cash transfer program resulted in an increased percentage of women delivering in a health facility and an increase in caesarean rates.  **Positive Impact** |
| Triyana 2017 | Indonesia | Experimental (RCT) | Antenatal care | **Target group:** Women  **PROGRESS Plus Measure:** Socioeconomic Status (SES) | Healthcare utilization | The program resulted in an improvement in ANC among women who attended private practice. Women were shown to be more likely assessed for weight, height, blood pressure, fundal height measurement, fetal heartbeat monitoring, external pelvic examination, iron-folic acid supplementation, information on pregnancy.  **Positive Impact** |
| Lopez-Arana 2016 | Colombia | Observational survey | Preventive health services & immunization | **Target group:** Children  **PROGRESS Plus Measure:** Place of residence and socioeconomic status | Healthcare Utilization  Other outcomes  *Nutrition* | The conditional cash transfer program was shown to significantly increase the probability of using preventive care services and growth and development check-ups  **Positive Impact**  The intervention led to significant positive impacts on dietary diversity and food consumption.  **Positive Impact**  The effects were mostly realized in rural areas |
| Kilburn, 2019 | Kenya | Observational Survey | N/A | **Target group:** Females aged 13–20  **PROGRESS Plus Measure:** Place of residence and socioeconomic status (SES) | Other outcome  *Nutrition* | The conditional cash transfer program was shown to significantly increase both total and food per capita household consumption.  **Positive impact** |
| Kusuma 2017 | Indonesia | Observational Survey | Growth monitoring and nutritional supplements | **Target group:**  children  **PROGRESS Plus Measure:**  Place of residence | Other outcome  *Nutrition* | The interventions resulted in an increase in children's food consumption from particularly for milk (19% increase) and fish.  **Positive impact** |
| Baird 2010 | Malawi | Experimental RCT | No health services, incentives are only for school attendance | **Target group:** young women  **PROGRESS Plus Measure:** socioeconomic status (SES) and age | Other outcome  *teenage pregnancy, risky sexual behavior* | reduction in teenage pregnancy, sexual activity, and risky sexual behavior.  **Positive Impact** |
| Olson 2019 | Brazil | Observational survey | Immunization, prenatal care | **Target group:** Pregnant mothers or children up to the age of 15 years  **PROGRESS Plus Measure:** Socioeconomic Status (SES) | Other outcome  *teenage pregnancy* | The intervention led to a 10% reduction in pregnancy among 16-17 years old girls and to a considerable delay in pregnancy for the same age group which suggest a positive impact of programs targeting education for teenage girls  **Positive impact** |
| Stecklov 2007 | Honduras,  Mexico,  Nicaragua | Experimental (Randomized impact evaluation) | Nutrition health services | **Target group:**  1) Pregnant women or children younger than 3 years of age  **PROGRESS Plus Measure:** Socioeconomic Status (SES) and place of residence | Other outcome  *fertility* | The intervention has shown no clear effect on fertility rates over the years.  **No impact** |
| Darney 2012 | Mexico | Observational Retrospective cohort | School education | **Target group:**  Women and children  **PROGRESS Plus Measure:** Place of residence | Healthcare utilization  *contraceptive use* | The intervention resulted in a higher proportion of women beneficiary using contraceptive method 41% compared with non-beneficiaries  **Positive impact**  The intervention highlighted disparities in adolescent pregnancy and education between rural and urban adolescent women over time |
| Kusuma 2016 | Indonesia | Experimental (RCT) | Prenatal care, assisted delivery, postnatal care, iron supplementation | **Target group:** Women and  children  **PROGRESS Plus Measure:** Socioeconomic status (SES) | Healthcare Utilization  Other outcomes  *Health Knowledge* | The conditional cash transfer program resulted in an increase in delivery preference at community-based facilities  **Positive impact**  The conditional cash transfer program resulted in improvement in Behavior change communication intervention attendance, but no improvement in health knowledge among mothers  **Inconclusive** |
| Morris 2004  Monetary incentives in primary health care and effects | Honduras | Experimental (RCT) | Child health services | **Target group:** Pregnant and lactating women and/or children under 7 y of age  **PROGRESS Plus Measure:** Socioeconomic status (SES) | Healthcare utilization | The intervention led to an increased coverage of antenatal care and well-child check-ups.  **Positive Impact**  Measles and tetanus toxoid immunization were not shown to be affected.  **Negative Impact** |
| Owusu-Addo 2014 | Latin America countries: Mexico/Colombia/ Honduras/Brazil/Zimbabwe/ Nicaragua | Systematic review | Health care utilisation, growth monitoring | **Target group:** Pregnant women, lactating mothers, infants, and children  **PROGRESS Plus Measure:** Socioeconomic | Healthcare utilization | The conditional cash transfer programs resulted in a significant impact on health service utilization including growth monitoring, antenatal care, and preventive check-ups for children.  **Positive Impact**  The program led to an increase in immunisation and vaccination coverage for children less than five years of age  **Positive Impact** |
| Kandpal 2016 | Philippines | Experimental (RCT) | Institutional delivery & antenatal care, prenatal care  immunization &growth monitoring & deworming | **Target group:** Children aged 0–5 y and 10–14 y, pregnant women  **PROGRESS Plus Measure:** Socioeconomic Status (SES) | Healthcare utilization  Child development  Other outcome  *Responsive caregiving* | The program resulted in a marginal significant increase in ANC visits.  Poor children aged 0–36 month were more likely to receive age-appropriate health services in the previous 6 month including regular growth monitoring, MMR vaccination and treatment-seeking cough, fever, and diarrheal disease  **Positive Impact**  The program resulted in a significant reduction in severe stunting in children aged 6–36 mo  **Positive Impact**  The program resulted in a significant change in key parenting practices, including childrens intake of protein-rich foods and care-seeking behavior, were concurrent.  **Positive Impact** |
| Shei 2014 | Brazil | Observational Survey | Immunization, growth monitoring | **Target group:** Children up to 17 years of age  **PROGRESS Plus Measure:** Socioeconomic status (SES) | Healthcare utilization  Morbidity | The program resulted in an increased number of health check-ups, growth monitoring.  **Positive impact**  The program led to an increase in health check-ups post visit for children younger than 7 years of age but not for older children.  **Inconclusive**  The intervention was associated with an increase odd of having diarrhea in children younger than 7 years of age but a decreased odd in older children  **Inconclusive**  The program was not shown to have any impact on cough or fever, physical and psychological health in children  **No impact** |
| Andrade 2012 | Brazil | Observational Survey | Immunization, prenatal care, postnatal care | **Target group:** Children 0 to 6 years of age  **PROGRESS Plus Measure:** Socioeconomic status (SES) | Healthcare Utilization | No impact on the immunization status of children was apparent throughout the study  **No impact** |
| Baba-Ari 2018 | Nigeria | Observational (Qualitative) | Maternal and child health service s | **Target group:** Pregnant women  **PROGRESS Plus Measure:** Socioeconomic Status (SES), Place of residence | Implementation considerations | Beneficiaries cash was shown to be the main incentive to increase women’s utilization of maternal and health services.  Barriers to service utilization included irregularity of cash transfers, and unclear payment mechanism. Moreover, beneficiaries reported using the cash transfer for other goods such as home appliances and food. |
| Sosa-Rubi 2011 | Mexico | Observational (Survey) | Regular use of health services including child health services and reproductive health consultations and health talks (called platicas) | **Target group:** Children, Pregnant/postpartum women  **PROGRESS Plus Measure:** Socioeconomic Status (SES) | Healthcare utilization | The conditional cash transfer program resulted in an increase in ANC visits, skilled birth attendance.  **Positive impact** |
| Edmond 2019 | Afghanistan | Observational Survey | Maternal and newborn health services | **Target group:** Rural pregnant women  **PROGRESS Plus Measure:** Place of residence | Healthcare utilization | The program resulted in an overall increase in facility deliveries and  ANC visit.  **Positive impact** |
| Thongkong 2017 | India | Modelling study | Hospital delivery in a government facility  Free transport to facility for pregnant women and newborns | **Target group:** Pregnant Women  **PROGRESS Plus Measure:** Socioeconomic Status (SES) | Healthcare utilization | The results revealed Poor-rich inequalities regarding delivery of the program with pro-rich inequalities when it comes to institutional delivery rate.  **Inconclusive**  Better off women were more likely to receive the benefits of the program |
| Thomas, R., 2012 | Nicaragua | Modelling study | Growth monitoring, vaccinations, nutrition supplements for anaemia, anti-parasite medicines for children | **Target group:** Children in rural households  **PROGRESS Plus Measure:** Socioeconomic Status (SES) | Healthcare utilization | The program resulted in an improvement in health checks for children younger than 3 years of age  **Positive impact** |
| Njuguna, J., 2019 | Kenya | Quasi-experimental | Postnatal care, immunization, growth monitoring, nutritional support, ANC | **Target group:** Pregnant Women  **PROGRESS Plus Measure:** Socioeconomic Status (SES) | Healthcare utilization | The intervention resulted in an increase in ANC attendance  **Positive impact** |
| Fink 2017 | Zambia | Observational Cohort study | Child health services | **Target group:** Children  **PROGRESS Plus Measure:** Place of residence | Healthcare utilization | Health check-ups were shown to increase with increased cash incentives.  **Positive Impact** |
| Servan-Mori 2019 | Mexico | Experimental  RCTs | Maternal and child health care | **Target group:** Women and Children  **PROGRESS Plus Measure:** socioeconomic status (SES) | Healthcare utilization | This study suggests that conditional cash transfer programs that address both the supply and demand side of health services (through strengthening the technical and human resource capacities) may increase the coverage of maternal and child health services  **Positive impact** |
| Rahman 2018 | India | Observational  Correlational  Fuzzy Regression Discontinuity  (FRD) | Institutional deliveries, antenatal care (ANC), PNC | **Target group:** Socio-economically disadvantaged women (19 years or older)  **PROGRESS Plus Measure:** Socioeconomic Status (SES) | Healthcare utilization | The program led to an increase in ANC and PNC service delivery, however not on immunization.  **Inconclusive** |
| Rao 2020 | India | Observational  (Discrete choice experiment) | Maternal and child health services: ANC visit, birth registration and  child vaccination | **Target group:** Children and pregnant women  **PROGRESS Plus Measure:** Socioeconomic Status (SES) | Implementation considerations | The study revealed that low service uptake was mostly among beneficiaries with low literacy and of low socioeconomic status |
| Barham, 2009 | Nicaragua | Experimental  RCTs | Growth monitoring; well-baby care; vaccinations; supplementation for anemia; and provision of anti-parasite medicine, preventive health care | **Target group:** Children younger than 3 years of age  **PROGRESS Plus Measure:** Socioeconomic Status (SES) and place of residence | Healthcare Utilization | This study resulted in a dramatic increase in vaccination coverage among treatment areas, in parallel with a rise in control areas. The program led to a 100 percent increase in immunization coverage over 2 years.  **Positive impact** |
| Sidney, K 2012 | India | Observational Survey | Healthcare setting deliveries | **Target group:** Women residing in the villages who gave birth in 2009  **PROGRESS Plus Measure:** Place of residence | Healthcare utilization | A number of surveys have demonstrated an increase in institutional delivery at the national level  **Positive impact** |
| Witvorapong 2016 | Afghanistan | Modelling study | Institutional delivery, vaccination | **Target group:**  Mothers and children  **PROGRESS Plus Measure:** Place of residence | Healthcare Utilization | The intervention resulted in an increase in institutional delivery among eligible participants  **Positive Impact** |
| Thomas 2015 | India | Descriptive (Case Study) | Institutional delivery | **Target group:**  Pregnant women and neonates  **PROGRESS Plus Measure:** Place of residence | Healthcare Utilization | The intervention has increased access to services by providing pregnant women with cash for transportation to a health facility at the time of delivery which contributed to an increase in institutional deliveries from 35% to 79.85% from 2005 2011.  **Positive impact** |
| Vellakkal 2017 | India | Observational (Qualitative study) | Antenatal and post-natal services, transport to facilities | **Target group:**  Pregnant women  **PROGRESS Plus Measure:** Place of residence | Implementation considerations | Cash incentive program did not have an impact on institutional delivery as it was perceived to cover an among lower than the total expenses of facility-based childbirth, in terms of both monetary and real costs.  **No impact** |
| Bogg 2016 | India | Observational (Secondary data analysis) | maternity services (delivery) | **Target group:**  pregnant women  **Progress plus:**  place of residence | Healthcare utilization | The JSY program led to an increase in C-section rates, in Madhya Pradesh (26.6. to 40.7%). |
| Anderson 2011 | Several countries | Technical report | nutrition: immunization, full antenatal care, nutritional supplements , increased skilled birth attendance | **Target population:**  CCT: children , pregnant women  **Progress plus:**  Socioeconomic status | Healthcare utilization | **Positive:** CCTs encourage access and uptake of essential health services |
| Souza 2022 | Brazil | Observational cross-sectional | antenatal  care, vaccination | **Target population:**  low-income families  (focus on 12 and 24 months children)  **Progress plus:**  socio-economic | Healthcare utilization | The study adds to the literature the positive impact of the conditional cash transfer programme on childhood vaccination, demonstrating that vaccination coverage of the up-to-date schedule was higher among beneficiaries when compared to the non-beneficiaries’ group  **Positive impact** |
| vonHaaren 2021 | India | Quasi-experimental (matched-pair design) | maternal and child health | **Target population:**  women aged 19 and older (covering the first two live births of all women)  **Progress plus:**  gender | Healthcare utilization | Consistent with the program’s conditions, we find increases in infant immunization.  long-term utilization of public health facilities becomes more frequent and intervals between eligible births increase by 17%  **positive impact** |
| Jacobs, 2022 | LMIC | systematic review | At least 3 ANC visits,  facility-based delivery  and 1 PNC visit. | **Target population:**  Pregnant women, children  **Progress plus:**  place of residence, gender | Healthcare utilization | Seven studies reported a statistically significant increase of over 5% in ANC service uptake. Two reported significantly higher ANC attendance in lower SES groups compared with control populations than did higher SES groups.  **Positive impact** |
| Khoza 2018 | Johannesburg, South Africa | Experimental (RCT) | Sexual reproductive health education, services related to family planning and contraception, HIV counselling and testing, HIV risk assessment, and HIV risk reduction counselling. | **Target group:** Adolescents  **PROGRESS Plus Measure:** Socioeconomic Status (SES), place of residence | Healthcare utilization | The condition of the conditional cash assistance program encouraged adolescents to attend the clinic. Girls were more likely to attend the clinic for assessments and boys were more likely to attend the clinic and get tested for HIV.  **Positive impact** |
| Rout 2018 | India | Descriptive study | free treatment, drugs, diagnostics, and transport service for mother and infant in public hospitals | Pregnant women  **PROGRESS Plus:** Place of residence | Healthcare utilization  Healthcare expenditure | The percentage of mothers that received JSY benefits was highest in Odisha followed by Chhattisgarh and Madhya Pradesh. In comparison to the major states, Odisha accounts for the highest (around 75%) institutional delivery in the public health facilities  **Positive impact**  In the light of available evidences, inadequate financial coverage by JSY scheme, additional expenses incurred by mothers on medicines, baby food and informal payments have been reported to be the contributing factors to the overall increase in OOPE. |
| Gopalan 2012 | India | Observational  Mixed metod (quantitative & qualitative) | In-facility delivery, skilled birth attendance and ANC, PNC | **Target group:**  Pregnant women  **PROGRESS Plus Measure:** Socioeconomic status (SES) | Healthcare Utilization  Healthcare Expenditure | The intervention resulted in considerable improvement in maternal healthcare utilization whereas the number of institutional deliveries increased by 20.3% annually  **Positive impact**  The intervention resulted in partial financial risk protection whereby it covered up to 25.5% of the maternal healthcare cost of the beneficiaries in rural areas and 14.3% in urban areas.  **Positive impact** |
| Landrian2020 | India | Observational Survey | Health facility delivery & obstetric care | **Target group:** Pregnant women  **PROGRESS Plus Measure:** Place of residence | Implementation considerations | More than 40% and 70% had to spend some out-of-pocket expenditures during childbirth  **Positive Impact**  Mean OOPEs were found to be the highest among those in the youngest age group, those with a higher education, those from the wealthiest groups. |
| Godha 2022 | India | Observational  survey | reproductive health services | **Target population:**  pregnant women  **Progress plus:**  Gender | Healthcare utilization  Implementation considerations | Overall, the national-level results suggest that not only did the utilization of maternal health care service increase substantially over the 2005 to 2015–16 study period, but also that wealth-based inequalities in the use of services decreased significantly.  the prevalence of having had three or more ANC visits increased by around 13 percentage points over the study period while ID and PNC almost doubled  **Positive impact**  The analysis of program participation by wealth quantiles suggests that program leakage to the richer population is evident, particularly within rural areas in both state categories. This indicates that improvements in targeting are needed, and this could potentially yield further progress in reducing wealth-based service inequality. |
| Kennedy 2014 | Tanzania | Observational (Qualitative study) | N/A | **Target group:** Young women (15-25 years of age)  **PROGRESS Plus Measure:** Place of residence | Implementation consideration | Beneficiaries suggested that conditional cash transfer programs are not sustainable and expressed worry that young women may misuse or mismanage. Respondents suggested that money may be sent to parents to advice their daughters on their use |
| Goli 2021 | India | Observational survey | MCH services | **Target population:**  pregnant women  **Progress plus:**  Gender | Healthcare expenditure | Except at rural public facilities, the average OOPE for institutional delivery has increased significantly in both rural and urban areas even after  adjusting to inflation in the prices. These facilities have experienced more than 50% raise in OOPE on institutional delivery during 2004-2017/18, despite JSY (CCT) incentives  **negative impact** |
| Skovdal 2014 | Zimbabwe | Experimental RCTs | vaccinations and attendance at a growth-monitoring clinic | **Target group:** Orphaned and vulnerable children  **PROGRESS Plus Measure:** Socioeconomic Status (SES) and place of residence | Implementation consideration  (Facilitators) | The study points to a widespread acceptance of conditions when it comes to community-led cash transfer initiative. Two main pathways are identified for the acceptance of conditions. (1) Conditions were, reasonable and viewed are proxy for good parenting and guardianship, (2) social recognition of respondents to the need to be held accountable for the money provided.  **Positive impact** |
| Gopichandran 2012 | India | Descriptive  (Case study) | Maternal services: antenatal and delivery services | **Target group:** Pregnant women  **PROGRESS Plus Measure:** Socioeconomic Status (SES) | Implementation consideration  (Barriers) | The case report suggests that mothers’ in-laws were shown to force their daughter-in-law to stay in their village, not allowing her to go to her mother’s house for delivery for the sake of securing the cash incentive, which is given only upon condition of the women delivering in the local institution where she is registered  **Negative impact** |
| Sidney 2016 | India | Observational Survey | Maternal health services | **Target group:** Pregnant women  **PROGRESS Plus Measure:** Place of residence | Implementation consideration (Facilitators) | Beneficiaries reported that delivering in health facility was nowadays considered a social norm, and that delivering in health facilities may help for proper care before and after giving birth |
| Gupta 2018 | India | Observational (Qualitative study) | Maternity health services | **Target group:** Pregnant women  **PROGRESS Plus Measure:** Place of residence | Implementation consideration: *Barriers*  *Facilitators* | The conditional cash transfer program was found to be not effective due to a variety of factors affecting the provision of quality care at the public health facility including: (1) the inability of the team to manage intra-partum and post-partum complications, leading to an increase in referrals, mostly to private facilities (2) insufficient healthcare personnel, infrastructure, medicine, and equipment; (3) uniform distribution of resources; and (4) the standalone nature of the elements of the program  Suggested improvements for the provision of care include extensive monitoring and evaluation, adequate human resources and financing schemes, staff training systems, early identification, and detection of medical problems. |
| Alves, 2023 | Brazil | Observational  Cohort | Maternity health services | **Women**  **Socio-economic status, place of residence, and race** | Mortality  (maternal mortality) | Our findings in this cohort study suggest that BFP (Bolsa Family Program) is associated with a reduction in maternal mortality among the poorest Brazilian women  The CCT BFP might affect maternal mortality through different mechanisms. First, the income transferred to women can have a more immediate effect, with the allocation of money for purchase of food, the use of health services, and mobility, particularly if it is coupled with shifts in intrahousehold power or autonomy favoring women. Second, by fulfilling conditionalities, BFP can increase access and the use of health services by reducing barriers and increasing the monitoring and treatment of comorbidities, facilitating referrals to high-risk prenatal care, and ensuring adequate assistance while giving birth  **Positive effect**  **Another important finding of our study is the association of BFP and maternal mortality among the most vulnerable (Black women, *Pardo*women, and women who live in rural areas and less developed municipalities), suggesting a potential contribution of BFP to reducing social inequalities** |
| Bustos, 2023 | Philippines | Observational |  | **Children**  **Socio-economic status** | Implementation considerations |  |
| Cavalcanti, 2023 | Latin America | Observational  Cohort | Child services | **Children**  **Socio-economic status and age** | Mortality  (under five child mortality rate) | Conditional cash transfer programs were associated with significant reductions of mortality rates in those younger than 5 years. The results of this cohort study suggest that the expansion of CCT programs could strongly reduce childhood hospitalization and mortality in Latin America and should be considered an effective strategy to mitigate the health impact of the current global economic crisis in low- and middle-income countries.  **Positive effect** |
| Palmer, 2022 | Kenya | Experimental  RCT | The Afya intervention was a CCT aiming to retain women in the continuum of care, from their first ANC visit until their children reach 1 year of age | **Women and children**  **Socio-economic status** | Implementation consideration | This study provides estimates of the cost of implementing the Afya intervention in rural Kenya, contributing to limited overall evidence on the cost-effectiveness of cash transfer programmes to improve maternal and child health. Based on the available evidence, it is not possible to conclude whether the Afya intervention was cost-effective. A simple comparison with current health expenditure in Siaya county suggests that the intervention as implemented is likely to be unaffordable |
| Patwardhan, 2023 | India | Observational |  | **Women and children**  **Socio-economic status** | Morbidity  (wasting) | the program reduced child wasting by 7 percentage points, a 39% reduction compared to the average prevalence of wasting in the pre-program period.  Reduction of 80% for children from households in the top four of five national wealth quintiles Vs 40% reduction for children from the bottom wealth quintile  **Positive impact still unequitable effect** |
