# Supplementary material for: Financing for equity for women’s, children’s and adolescents’ health in low- and middle-income countries: A scoping review
Source: PLOS Glob Public Health. 2024 Sep 12;4(9):e0003573. doi: 10.1371/journal.pgph.0003573 (PMC11392393; doi:10.1371/journal.pgph.0003573)
Supplement: S2 Table — (DOCX) [file pgph.0003573.s005.docx]

**S2 Table of characteristics: HI (n=89)**

| **Author Year** | **Country** | **Study design** | **Type of insurance** | **Health service covered** | **Target group and PROGRESS Plus**  **measures** | **Outcome(s)** | **Main Results**  **Is the intervention effective overall? (yes/no/inconclusive)** |
| --- | --- | --- | --- | --- | --- | --- | --- |
| Mozumdar 2018 | India | Observational (Cross-sectional survey) | National Health Insurance Scheme: (RSBY) | Reproductive healthcare services (family planning included) | Target: women  PROGRESS plus: place of residence and socio-economic status | Healthcare utilization | The use of FP/RH services in RSBY empaneled hospitals was very low (2% or less for any service).  **No impact on** |
| Ettenger 2014 | Colombia | Observational Survey | Colombia’s nationalized health insurance schemes (Ministerio de Proteccio´n Social (MPS) 2000; Grisales and Giraldo 2008 | ANC health services with HIV test inclusion | Target: Pregnant women  PROGRESS plus: Occupation | Healthcare Utilization | Women enrolled in the subsidized regime were significantly less likely to be offered and receive an HIV test in ANC  than women without any health insurance (adjusted odds ratio ¼ 0.820, P < 0.001), when controlling for the other independent variables.  enrolment in the subsidized regime reduced access to HIV testing in ANC  The creation of Colombia’s national subsidized regime has increased health insurance coverage, yet, for one particular  essential health service, there is evidence of decreased access.  **No impact** |
| Yip 2001 | Egypt | Observational Survey | The School Health Insurance Plan (SHIP) | The health insurance programme provides a comprehensive benefit package, including preventive services, outpatient care, inpatient care, subsidized  pharmaceuticals and medical appliances | Target: School children  PROGRESS plus: Age | Healthcare utilization  Healthcare expenditure | SHIP significantly improved access by increasing visit rates  With regard to the success  of targeting the poor, conditional upon being covered, the SHIP reduced the differentials in visit rates between the  highest and lowest income children  **positive**  Reducing financial burden of use (out-of-pocket expenditures).  Positive |
| Long 2010 | China | Observational (Cross sectional study) | New rural health insurance system (New Co-operative Medical system) | Maternal health services | Target: Women  PROGRESS plus: place of residence | Healthcare utilization  Healthcare Expenditure | Between 2002 and 2007, having no any pre-natal visit decreased from 25% to 12% (differ- ence 13%, 95% CI 10–17%); facility-based delivery increased from 45% to 80% (difference 35%, 95% CI 29–37%); and differences in using pre-natal and delivery care between the income groups narrowed.  **Positive Impact**  the expenditure for facility-based delivery increased over the period, but the out-of-pocket expenditure for delivery as a percentage of the annual household income decreased. In 2007, it was 14% in the low-income group. NCMS participation  was found positively correlated with lower out-of-pocket expenditure for facility-based delivery (coefficient )1.14 P < 0.05) in 2007.  **Positive Impact** |
| Palmer 2015 | Vietnam | Observational  Regression Discontinuity approach | Health Insurance |  | Target: Children under 6  PROGRESS plus: Age | Healthcare Utilization  Healthcare Expenditure  Implementation Considerations | The study finds a positive impact on inpatient and outpatient visits. We find moderately high use of private outpatient services and no evidence of a switch from private to covered public facilities under insurance.  **Positive Impact**  No significant impact on expenditures per visit at public facilities.  **No impact**  Results suggest that adopting public health insurance programs for children under age 6 may be an important vehicle to improving service utilization in a low- and middle-income country context. Challenges remain in providing adequate protections from the costs and other barriers to care. |
| Wagner 2018 | Philippines | Experimental (RCT) | Health Insurance | Hospital services | Target: Children  PROGRESS plus: socio-economic status and place of residence | Healthcare Expenditure | Compared to controls, interventions that expanded insurance and provided performance-based provider payments to improve quality both resulted in a decline in out- of-pocket spending (21% decline, p-value = 0.061; and 24% decline, p-value = 0.017, respectively). With lower out-of-pocket payments for hospital care, monthly household spending on personal hygiene rose by 0.9 (p- value = 0.026) and 0.6 US$ (p-value = 0.098) under the expanded insurance and provider payment interventions, respectively, amounting to roughly a 40–60% increase relative to the controls.  **Positive impact** |
| Velasquez-DeCharry 2009 | Colombia | Observational (Retrospective cohort) | Social Security health insurance | Breast cancer treatment | Target: Women  PROGRESS plus: Gender | Implementation Considerations | Now, with respect to the barriers that women faced in making use of the intervention’s therapies included in their treatment, it was recognized  that the main obstacle was the need to move to another city (25.3%), followed by authorizations  of the paying entities (20.4%) and the amounts of the medications (14.3%). |
| Agbadi 2021 | Ghana | Observational Survey | Ghana’s health insurance scheme | Maternal and child health services | Target: women and children  PROGRESS plus: socio-economic status | Healthcare utilization  Mortality | Assisted health facility deliveries have improved from 47% in 2003 to 78.1% in 2017.Basic vaccination  coverage among children 12–23 months has improved from 69% in  2003 to 77% in 2014  **Positive impact**  Maternal mortality has reduced from 484 per 100,000 live births in 2000  to 308 per 100,000 live births in 2017  **Positive impact** |
| Chen 2012 | China | Observational (Cross sectional survey) | National Cooperative Medical System | General health | Target: children from 6- 16 years of age  PROGRESS plus: age and place of residence | Mortality | Lower mortality of young children and pregnant women.  **Positive impact** |
| Ganguly 2021 | India | Experimental (RCT) | National Health Protection Scheme under Ayushman Bharat Program | Oncology Services | Target: Children with cancer  PROGRESS plus: Socio-economic status | Morbidity | Financial support through the government promoted health insurance and holistic support through philanthropic organizations have improved treatment adherence and outcome |
| Perez-Cueva 2012  *Lessons learned from the first evaluation of the Medical Insurance for a New Generation: Bridging research and policy* | Mexico | Descriptive  case study | Medical Insurance for a New  Generation program (SMNG) | Child and neonates’ health services | Target: children under 5 years of age  PROGRESS plus: age | Morbidity | The findings support the conclusion that a program like this is necessary  to improve the health conditions of Mexican children.  **Positive impact** |
| Zhou 2021 | China | Observational  Logistic regression model | Urban Employee Basic Medical Insurance (UEBMI) | General medical services | women  PROGRESS plus: Place of residence and occupation | other outcomes  *insurance coverage* | because the UEBMI in China mainly covers people with formal jobs, a lower labor participation rate (even much lower in formal jobs) of women has led to their greater difficulty in obtaining health insurance. Since the older women’s greater difficulty in obtaining jobs or susceptibility to lay-offs during the period of the UEBMI’s implementation, the possibility of being covered was even much lower. In fact, it was because of the combined effects of the UEBMI system and the labor market condition at that time that older women had a lower proportion of being covered under the UEBMI.  Negative impact |
| Blanchet 2012 | Ghana | Observational Survey | National Health Insurance Scheme | Outpatient services, maternity care services | Target: Women  PROGRESS plus: Socio-economic status | Healthcare utilization | We find that on average individuals enrolled in the insurance scheme are significantly more likely to  obtain prescriptions, visit clinics and seek formal health care when sick,e only 7.4 per cent of women not enrolled in the NHIS report a hospitalization during the 12 months  preceding the interview, the same was true for 12.7 per cent of women enrolled in NHIS. Similarly, women  with NHIS insurance report on average 0.576 prescriptions, which is more than twice as many as  women who were not enrolled at the time of the interview (0.274 prescriptions on average  **Positive impact** |
| Quayyum 2010 | Indonesia | Observational Retrospective cohort | Health insurance scheme | obstetric care, life threatenning obstetric compications | Target: pregnant women  PROGRESS plus: socio-economic status | Healthcare expenditure | Insurance for the poor appeared to be relatively effective in protecting households from catastrophic consequences of  payment for obstetric care. Insurance for the poor appears to have some  positive association with the hospitals’ expenditure for treatment of different  types of maternal care  **Positive impact** |
| Dixon 2014 | Ghana | Observational Survey | National health insurance scheme | Health care services | Target: women  PROGRESS plus: place of residence and socio-economic status | Implementation consideration | Insurance drop-out due to income:  those with progressively less reliable incomes were more likely to drop out of the NHIS. Specifically, women with somewhat unreliable or very unreliable incomes  were 2.1 times and 2.5 times, respectively, more likely to drop out of the NHIS when compared with women having very reliable incomes.  Bivariate results also indicate that food security was a highly significant predictor of dropping out, especially for women. Women who were food- insecure were 50% more likely to drop out of the NHIS compared with those who were food- secure.  The second factor that weighed heavily on both genders was education: the higher the level of education an individual has completed the less likely he or she is to drop out of the NHIS.  Women in particular seem to be dropping out for reasons that are tightly intertwined with cultural gender norms, and are not therefore making rational and free decisions that could be discouraged through a block- out penalty. |
| Zhu, 2008 | China | Observational Survey | National health insurance scheme | Hospitalization, surgery, special treatments such as chemotherapy and dialysis | Target: children  PROGRESS plus: place of residence and occupation | `Healthcare Utilization | because the three groups of children were generally healthy and similar in baseline health, the differences in health-seeking behaviors were likely attributable to the security afforded by insurance. However, as indicated by self-reported 12- month outpatient visitation data, care utilization patterns did not differ among the three groups (p = 0.796)  **No impact** |
| Quimbo, S. A.; 2011 | Philippines | Modelling study | Social Health insurance Program | Hospitalization and In-Patient care | Target: Children under 5  PROGRESS plus: Socio economic status | Morbidity | we estimated a 9-12 and 4-9 percentage point reduction in the likelihood of wasting and having an infection, as measured by a common biomarker C-reactive Protein, respectively. Interestingly, these benefits were not apparent at the time of discharge; the beneficial health effects were manifest several weeks after release from the hospital.  **Positive Impact** |
| van Hees, S., 2019 | LMICs | Systematic Review | SHI: Social Health insurance , PHI: private Health Insurance , CBHI: Community Based Health Insurance | N/A | Target: women &children  PROGRESS plus: gender, disability, race or ethnicity | Healthcare Utilization  Healthcare Expenditure | There were generally positive effects of health insurance on utilization of services for individuals with disabilities  **Positive Impact**  Financial protection was underreported,  however the majority of included studies showed negative results  **No Impact** |
| Parmar, 2019  Impact of an employment guarantee scheme on utilisation of maternal healthcare services: Results from a natural experiment in India | India | Observational Survey | Employment Guarantee Scheme | N/A | Target: ever-married women (aged 15–49) who have given birth  PROGRESS plus: place of residence and socio-economic status | Healthcare utilization | We find NREG increased deliveries at public facilities by 3.7 percentage points in disaster-prone regions compared to an increase of 1.9 percentage points in regions that are not disaster-prone. Furthermore, in disaster-prone regions, NREG increased facility delivery by 2.4 percentage points while reducing home deliveries by 2.8 percentage points. This indicates that the availability of good public infrastructure, often developed as part of NREG, can reduce barriers to facility utilisation. Finally, NREG also reduced borrowing in disaster-prone regions by 4.2 percentage points, indicating that NREG may have provided some financial protection in the rural areas that are prone to disasters  **Positive Impact** |
| Sivakumar, 2017 | India | Narrative/litterature Review | Swavlamban Health insurance scheme | N/A | Target: children and adolescents with disabilities  PROGRESS plus: Disabilities | Healthcare expenditure | Swavlamban health insurance scheme has added benefit of covering parents/ legal guardian of child/ adolescent with disability with a family floater of Rs 2  **Positive Impact** |
| Mendhe, 2021 | India | Observational Survey | National health insurance scheme | N/A | Target: women above 18 years of age who were hospitalized  6 months prior to the study  PROGRESS plus: place of residence and socio-economic status | Healthcare expenditure | The RSBY card reduced the inpatient OOPE. RSBY card prevented catastrophic OOP in most of the respondent. RSBY has made health services available to all sections of community at minimal costs, helping them to access better health care that could not be afforded by them.  **Positive impact** |
| Islam, 2012 | Bangladesh | Observational Survey | Micro Health Insurance plan (MHI) | ANC services | Target : pregnant women  PROGRESS plus: place of residence and socio-economic status | Healthcare utilization | It is possible that enrollment in MHI is effective in increasing the number of ANC visits, except for those in the destitute and ultra-poor classes  **Positive impact but not for ultra-poor classes** |
| Servan-Mori, 2015 | Mexico | Observational Survey | voluntary health insurance | Maternal services | Target : pregnant women  PROGRESS Plus: Occupation | Healthcare Utilization | among women with untimely antenatal  care (i.e., first visit after the third gestational month), only  67 % of non-SP women attended a minimum of four  antenatal visits, compared to 75 % in SP women  (p<0.01).  SP membership  increased the odds of four antenatal visits by 65 %  (p<0.01), and each additional year of education increased  those odds ratios by 1.06 (p<0.01). However, when timely antenatal care was included in the model, the  results showed that SP lost it influence on complete antenatal care: regardless of SP status, among women with  timely antenatal care, the probability of≥ 4 antenatal visits  (p<0.01) was 6.22 times higher  **Positive impact** |
| Comfort 2013 | LMICs | Systematic review | Health insurance | Maternal health services | Target: women and children  PROGRESS plus: socioeconomic status | Healthcare Utilization  Mortality | None of the studies can conclusively demonstrate a causal relationship between insurance and maternal healthcare-use because none relied on randomized methods. The literature does consistently indicate the expected associations between insurance and MH service-use. Among the studies which focused on facility-based deliveries and skilled attendance at birth, there was mostly consistent evidence that health insurance is positively correlated with both measures. The studies provided examples of this positive correlation in different geographic areas, including SSA (Ghana, Mali, Rwanda, and Senegal), Asia (India and China), Latin America (Peru and Colombia), and Europe (Turkey)  **Positive impact on Facility based deliveries but inconclusive Impact on overall maternal health care uses**  The available evidence (in two studies) identified a negative correlation between health insurance and neonatal deaths (15,29). In Brazil, gestational age-specific neonatal mortality and birthweight specific neonatal mortality decreased among birth cohorts over time as insurance coverage expanded.  **Positive Impact** |
| Mohanty 2017 | India | Observational Survey | The National Health mission (NHM) | Delivery services | Target: pregnant women and children under 5  PROGRESS plus: place of residence | Healthcare utilization  Healthcare expenditure | The utilization of prenatal care had increased from 75% in 2004 to 90% in 2014. Postnatal care was estimated at 64% in 2004 and 79% in 2014. Among the three maternal care services, the increase in prenatal care was the highest while the postnatal care was the least during the post NHM period.Since 2004, all three maternal care  has recorded increase in public health centers  **Positive Impact especially on prenatal services**  Based on these findings, we conclude that the NHM is effective in increasing in utilization, continuation of services in public health centers and reducing OOPE and CHS in public health centers on maternal care.  Positive impact on reducing OOPE |
| Akazili 2014 | Ghana | Observational Survey | National health insurance | delivery, encouraging access to quality health care services, | Target: women and children  PROGRESS plus: socio-economic status | Other outcomes  *Insurance coverage*  Implementation consideration | 33.9 percent of women in the lowest SES quintile compared to 58.3 percent for those in the highest quintile were insured. Results show that the relatively well educated, prosperous, married and Christian respondents were more likely to be insured than other women. Conversely, women living in remote households that were relatively poor or where traditional religion was practiced had lower odds of insurance coverage.  ***inequity***  About 60 percent of respondents were registered. However, only 40 percent had valid insurance cards indicating that over 20 percent of the registered respondents did not have insurance cards. Thus, a fifth of the respondents were women who were registered but unprotected from the burden of health care payments |
| Bonfrer 2016 | Ghana | Observational Survey | National Health Insurance Scheme | Maternal services | Target: pregnant women and children  PROGRESS plus: socio-economic status | Healthcare utilization | shows that NHIS enrollment increased the percentage of children who obtained at least four ANC visits by 7 percentage points. * NHIS membership has a positive  effect on attended delivery (10 pp, p = 0.002). When limiting this to only include the attended  deliveries in public facilities, we again find significant positive effects of NHIS enrollment,  with 12 pp increase (p = 0.000), which suggests that NHIS membership is not so much crowding out private deliveries.  **Positive Impact on ANC visits and attended deliveries and on decreasing the percentages of unwanted pregnancies especially among the poor**  NHIS membership seems to have had no effect on children receiving Vitamin A supplement, nor on childhood vaccinations  **No impact on vaccinations and vitamin a supplement consumption because they are usually provided for free** |
| Jafree 2021 | Pakistan | Observational Retrospective cohort | Microfinance health interventions | N/A | Target: women  PROGRESS plus: socio-economic status and occupation | Morbidity | women receiving health insurance had a significantly greater chance of overall perceived good health. According to NNM, 17.4% of women with health insurance had a greater likelihood of overall perceived good health; the results for KM showed a greater likelihood in 11.8%.  **Positive impact on improving overall health of enrolled women** |
| Perez-Cuevas 2012  *Utilization of health care services among children memebrs of Medical Insurance for a New generation* | Mexico | Observational  Survey | Medical Insurance for a New Generation | Provision of healthcare comprises ambulatory (preventive and curative care), and hospital services (emergency and in-patient care) at MoH facilities through a package of 116 medical care interventions | Target: children under 5  PROGRESS plus: Age | Healthcare Utilization | This analysis was of children who acute diarrhea, acute respiratory infection or other  acute and chronic conditions. Up to 83% of children used the services. Regarding the institutions that provided curative care, 80% of children in the rural area were taken to MoH facilities, whereas 61% of urban children used the services of this institution. Private services were used 35% of urban and 22% of rural children.  **Positive Impact on increase services use by children with acute diarrhea and acute respiratory infection** |
| Ahinkorah 2020 | Ghana | Observational Survey | National health Insurance | Maternal services | Target: adolescents  PROGRESS plus: place of residence and socio-economic status | Other outcomes  *Insurance coverage* | The study found that ecological zone has a statistically significant association with health insurance coverage among adolescent girls in Ghana, with adolescent girls in the Northern Zone less likely to be covered by health insurance than those in the Coastal Zone. Other factors that determined health insurance coverage among adolescent girls in Ghana were age, parity, education level and marital status.  **inequity** |
| Ibrahim 2016 | Ghana | Observational Retrospective cohort | National Insurance Scheme (NHIS) | Prenatal and delivery services | Target: pregnant women  PROGRESS plus: socio-economic status and place of residence | Healthcare utilization  Mortality rates | On average, less than 14% of infants were born through Caesarean method during the Cash and Carry period compared to 20% Caesarean births during the NHIS period  **Positive impact**  Overall, about 8% of infants born during the Cash and Carry system died, compared to an average of 4% during the NHIS period in the Northern Region of Ghana. There were no remarkable differences observed in the rate of infant deaths among mothers in all age  categories in both the Cash and Carry and the NHIS systems except in mothers aged 35 or more who lost more  infants during the Cash and Carry period (p = 0.001)  compared to mothers in the same age group under NHIS  (p = 0.504)  **Positive impact on decreased infant mortality rate especially in women aged 35 and above** |
| Nasution 2020 | Indonesia | Observational Survey | National health Insurance( (Jaminan Kesehatan Nasional [JKN]) | maternal services in specific delivery services- Skilled birth attendants | Target: women  PROGRESS plus: socio-economic status | Healthcare utilization | The progress of SBA coverage in every province indicated an increase from 2012 to 2016, but the progress varied in every region and province. At the province level, the lowest progress in 5 years (2012-2016) was in Maluku and the highest progress was in DI Yogyakarta Province  **Positive impact on increase on skilled birth attendants with variations to provenance**  At the national level, patients’ preferences for doctors as birth attendants increased after the implementation of JKN in 2014. In contrast, preferences for nurses and midwives decreased. Preferences for doctors increased significantly in the nonsubsidized health insurance group, mostly referred to the wealthy group  **Positive impact on consulting with physicians especially among the wealthy group and no impact on consulting midwives and nurses** |
| Kuwawenaruwa 2016  *Implementing demand side targeting mechanisms for maternal and child health- experiences from national health insurance fund program in Rungwe District, Tanzania* | Tanzania | Descriptive (Case study) | MCH insurance card (social health insurance program) | maternal services | Target: pregnant women  PROGRESS plus: socio-economic status and place of residence | Healthcare utilization | The switch to geographic targeting also resulted in an  increase in enrolment in the national insurance scheme.  Women who receive the MCH card are entitled to a  CHF card for one year together with their partner and  up to four children. This card can be used to access health care services from public primary-level facilities.  With the greater enrolment following the switch to geographic targeting for the MCH card, coverage of CHF in  the district has increased overall from about 3 to 11 %  **Positive impact when geographical targeting was used** |
| Kuwawenaruwa 2016  *The effect of MCH insurance cards on improving equity access and use of maternal and child health care services in Tanzania: a mixed method analysis* | Tanzania | Observational  Qualitative study | Free insurance cards to poor women and their families | pregnancy and delivery-related complications | women  socioeconomic status | Implementation considerations | Women with low educational attainment were under-represented amongst those who reported having received the maternal and child health insurance card and used it for facility delivery. To improve equity in access to facility-based delivery care using strategies such as maternal and child health insurance cards is necessary to ensure beneficiaries and other stakeholders are well informed of the programme, as giving women insurance cards only does not guarantee facility-based delivery |
| Williams 2017 | Ghana | Observational Survey | National Health Insurance Scheme | Children health services | Target: everyone with focus on children under 18  PROGRESS plus: Age | Implementation consideration | Our findings indicate that 45.6% of sampled children remain uninsured, despite the introduction of premium waivers for this group. Furthermore, only 42.5% of households enrolled all household children; 15.8% of households only insured some children, thus remaining partially-enrolled, while 41.6% of households have not enrolled any child members.  Our results indicate that socioculturally, economically and politically excluded children are less likely to enroll in the NHIS. These results suggest that equity in access for socially excluded children has not yet been achieved. Efforts should be taken to improve coverage by removing the remaining small, annually renewable registration fee, implementing and publicising the new clause that de-links premium waivers from parental membership, establishing additional scheme administrative offices in remote areas, holding regular registration sessions in schools and conducting outreach sessions and providing registration support to female guardians of children |
| Wang 2014  *Role of the new rural cooperative medical system in alleviating catastrophic medical payments for hypertension, stroke and coronary hearl disease in poor rural areas of China* | China | Observational Survey | New Rural Cooperative Medical System (NRCMS) | inpatient and outpatient services for Hypertension, CHD and stroke | Target: everyone with a focus on women  PROGRESS plus: place of residence | Healthcare expenditure | he NRCMS managed to decrease the poverty impact of OOP payments due  to hypertension, stroke and CHD to 1.2% (from 6.9%  pre-reimbursement to 5.7% post-reimbursement).The NRCMS alleviated the financial burden of these  diseases on the households to some extent, but the effects  were statistically insignificant  **inconclusive impact** |
| Kuwawenaru., 2019 | Tanzania | quasi-experimental  Controlled before and after | National health insurance | N/A | Target: pregnant women  PROGRESS plus: place of residence & socio-economic status | Healthcare Utilization  Healthcare expenditure | The KfW scheme had no effect on the utilization of ANC, deliveries, postnatal care, childhood immunization or family planning  **No impact**  There was no evidence of a programme effect on the probability of paying for ANC, but the amount paid reduced by TZS 95.72 (95% CI: -205.9; +14.5; p = 0.087) (1 USD was equal to 1600 TZS in 2013); (Table 2). The probability of paying for delivery care halved as a result of the intervention but the effect was not statistically significant (Table 2). There was a reduction in the amount paid for deliveries by TZS 6,237 (95% CI: -10429.8, −2043.1; p = 0.004). There was no evidence of programme effect on the probability of paying for PNC or of giving a gift or the value of the gift  **No impact** |
| Ramos, 2020 | Peru | Observational (Survey) | Peru has introduced a tax-financed health insurance scheme called “Sistema Integral de Salud (SIS)” to foster progress towards Universal Health Coverage. The scheme explicitly targets the poorest sections of the population | N/A | Target: vulnerable and poor communities, including uninsured children & pregnant women  PROGRESS plus: socio-economic status | Other outcomes  *Insurance coverage* | Women in the SIS group were found to have lower educational levels, live in rural areas and more likely to be poorer. Women in the Standard insurance group were found to be more educated, more likely to be “Spanish”, and to be wealthier. Our study confirms that SIS has been effective in increasing coverage among vulnerable women, with coverage rates comparable with those observed among men.  **Positive impact/equity** |
| Muñoz-Hernández 2012 | Mexico | Technical report | Medical Insurance for a New Generation (SMNG) | primary, secondary and tertiary level health services | Target: children  PROGRESS plus: Age | Healthcare utilization | If the MoH services are unavailable, SMNG will make health care accessible to the children, through agreements reached between SMNG and social security institutions and private providers  **Positive impact** |
| Peng 2016 | China | Observational (Survey) | the New Rural Cooperative Medical System (NRCMS) | children and nutriotional status of mothers | Target: women & children  PROGRESS plus: place of residence | Morbidity | Our DID estimation shows that probability of malnutrition for children who live in the counties which enrolled in the NCMS in 2004 is significantly decreased (by 15 % points), while the effect is not significant for those counties that rolled out the program in 2006.That is, the uptake of NCMS insurance results in a 27 %-point decline in the likelihood of being malnourished among children. Our results  show that it is the youngest children who benefit most  from the NCMS, thus implying  **Positive Impact on decreasing malnourishment among children especially females** |
| Brooks 2017 | Indonesia | Observational  mixed methods (quanitative and qualitative) | health insurance for the population program | maternity benefits, including antenatal  care, institutional delivery, and postnatal care | Target: pregnant women  PROGRESS plus: socioeconomic status (SES) | Healthcare Utilization | The results of this study demonstrate that the recent  Jamkesmas health insurance program in Indonesia targeted  to the poor and the near-poor is positively associated  with HFD and SBD..  Poor women with  Jamkesmas were 19% (OR = 1.19 [1.03–1.37]) more likely to have health facility delivery (HFD) and 17% (OR = 1.17 [1.01–1.35]) more likely to  have skilled birth delivery (SBD) compared to poor women without insurance  **Positive impact** |
| Saavedra-Avendano 2016 | Mexico | Observational (Cross-sectional study) | public health insurance | prenatal care services | Adolescents  PROGRESS plus: age | Healthcare Utilization | The multivariable predicted probability of timely and frequent prenatal care improved over time, women with Social Security had higher probability than women with Seguro Popular and without health insurance.  The results presented support the study hypotheses. Among the adolescent women who received prenatal care, those with health insurance were more likely to receive timely, frequent and adequate prenatal care.  **Positive impact** |
| Lattof 2018 | Ghana | Observational  mixed-methods | publicly funded  NHIS |  | Migrant women  PROGRESS plus: socio-economic status and ethnicity | Healthcare expenditure | Both insured and uninsured migrants did not seek formal health services due to the unpredictable nature of out-of-pocket expenses.  Catastrophic and impoverishing medical expenses  also drove participants’ migration in search of work to repay loans and hospital bills. Health insurance can help minimize these expenditures, but only 17.4% of currently insured participants  (58.2%) reported holding a valid health insurance card in Accra. The others lost their cards or forgot  them when migrating  positive |
| Chen 2014 | china | Observational  Correlational | China’s Urban Resident Basic Medical Insurance |  | children, the elderly, and other unemployed urban residents  PROGRESS plus: socio-economic status and age | Healthcare utilization | this program has significantly increased the utilization of formal medical services. this program has improved medical care utilization more for the elderly, for the low- and middle-income families, and for the residents in the relatively poor western region.  Positive impact |
| Castro-Ríos 2019 | Mexico | Experimental (RCTs) | The Instituto Mexicano del Seguro Social (IMSS) | Acute lymphoblastic leukemia, Cancer survival, | children  PROGRESS plus: socio-economic status | Mortality | The Cox model showed that children who had been IMSS-insured for less than half their lives had more than double the risk of dying than those who had been insured for their entire lives |
| Fan 2021 | China | Observational | public health insurance |  | Children  PROGRESS plus: socio-economic status and place of residence | Other outcomes  *nutrition* | NCMS coverage did not significantly improve nutritional status, especially with respect to high-quality nutrient intake among rural poor children. Meanwhile, the findings also showed that there were differential effects by age and sex. Specifically, the greatest negative effect of NCMS coverage on nutritional intake was among children aged 0 to 5 years and for girls  **No impact/negative mostly for 0-5 and girls** |
| Renaudin, 2007 | Mauritania | Descriptive (case study) | Obstetric risk insurance |  | pregnant women  PROGRESS plus:  place of residence & socioeconomic status | Healthcare utilization | In 5 years, the percentage of the city's deliveries in ORI health facilities has increased by 64.3%, from 29.4% in 2001 to 48.3% in 2005. The highest increase rate occurred in 2004 when a new district joined the program. the post-natal consultation rate is 81% in the zone versus 50% outside.  The cesarean delivery rate increased from 2.6% in 2003 to 3.5% in 2005.  **Positive impact** |
| Celhay 2019 | Mexico | Observational (retrospective study) | public health insurance | - | children younger than 5 years  PROGRESS plus: socio-economic status | Mortality  Morbidity  Healthcare expenditure  Quality of care | SMSXXI reduced late neonatal mortality (7%) and infant mortality (5%) related to conditions covered  About 3–6 years after SMSXXI started, children reported having better health status and lower incidence of influenza and diarrhea  SMSXXI contributed to financial protection by reducing OOPHE by 15·6% and even more for expenditures related to neonates with critical conditions.  positive  SMSXXI promoted increased supply and quality of care in hospitals through improvements in infrastructure (ie, more neonatal care units) and specialized staff. |
| Gardezi 2021 | Pakistan | Observational (cross-sectional) | Public health insurance | Maternal health services | women  PROGRESS plus:  socio-economic status and place of residence | Healthcare utilization | there has been a positive increase in utilization of hospital services. The results show that districts where the programme was introduced have higher likelihood of hospital birth and of having skilled attendants at birth  *the programme did not lead to an increase in utilization of these maternal health outcomes in rural areas* **(no increase in rural areas)** |
| Gouda 2016 | Philippines | Observational survey | health insurance | facility based delivery | pregnant women  PROGRESS plus:  place of residence and socio-economic status | Healthcare utilization | increasing health insurance coverage is likely to be an effective approach  to increase women’s access to facility based delivery FBD. our results suggest that insurance coverage for women in poor and  rural households is possibly leading to an increasing demand for services and higher probabilities of FBD. Our results also suggest  that for poor and rural women, having access to insurance led to increased utilization of FBD.  **Positive impact** |
| Kesuma 2016 | Indonesia | Observational Survey | insurance scheme called Jaminan Kesehatan Aceh (JKA) | maternal healthcare services | pregnant women  PROGRESS plus:  socio-economic status | Mortality | Utilization of maternal health-care services and satisfaction has a positive relationship, and this helps to reduce maternal mortality |
| Boutayeb 2016 | morocco | Observational Survey | mandatory health insurance |  | women and children  PROGRESS plus:  socio-economic status, education and place of residence | Mortality  Healthcare utilization  Morbidity | Indeed, infant mortality decreased significantly from 63.1 deaths for 1000 live births in 1992 to 28.8 in 2011 (p < 0.05). Over the same period, the under-five mortality rate has seen a more accelerated decrease from 83.9 to 30.5. The maternal mortality decreased from 227 in 2000 to 112 in 2010  **Positive impact**  the percentage of deliveries assisted by skilled  personnel increased from 31 % in 1992 to 73.5 % in  2011. Similarly, the percentage of women having at least  one prenatal visit increased significantly from 33 to  77.1 % during the same period (p < 0.05). The postnatal  care also increased but it remains at a low level (21.9 %). The contraception prevalence and vaccination of infant  aged 12–23 months increased significantly from 41.5 % in 1992 to 67.4 and 99.6 % in 2011, respectively (p < 0.05). The percentage of caesarean sections increased from 2 % in 1992 to 9.6 % in 2011.  **Positive impact**  The proportion of children suffering from stunting decreased from 22.6 % in 1992 to 14.9 % in 2011. Deficiency in Iron, vitamin A and vitamin D affect respectively one third of children aged 6 months to 5 years, 20 and 10 % of under 5 years children. Similarly, pregnant women have an anemia prevalence of 37.2 %. |
| Habib 2021 | Pakistan | Observational survey | insurance benefit package |  | women from low-income households  PROGRESS plus:  socio-economic status | Healthcare expenditure | Our study concludes that health insurance schemes can be introduced in urban areas, to prevent low-income households from facing impoverishment and financial catastrophe due to the burden of OOP payments.  Positive impact |
| Gu 2019 | china | Observational Prospective cohort | insurance scheme | stroke management | Women patients with stroke  PROGRESS plus:  place of residence | Mortality | One-year all-cause mortality and stroke recurrence were both significantly higher in women patients with NRCMS than those with URBMI/UEBMI. One-year all-cause mortality and stroke recurrence were both significantly higher in women patients with NRCMS than those with URBMI/UEBMI |
| Xie 2021 | china | Observational Prospective cohort | public health insurance | breast cancer treatment | women: female breast cancer patients  PROGRESS plus:  place of residence | Mortality  Healthcare expenditure | Findings suggest that underinsured patients  face a higher risk of breast cancer-specific mortality in developing countries.  underinsured patients face greater financial burden and are less likely to afford out-of-pocket medical expenses for advanced therapy.24 For instance, trastuzumab was not covered by the insurances during the study period, and the high out-of-pocket medical cost may prevent financially vulnerable patients from such therapy |
| Barua 2020 | Thailand | Observational Retrospective cohort | Insurance schemes | outpatient (OP) and inpatient (IP)services, health promotion and disease prevention, and high-cost care | Stateless children aged between 0and 18years  PROGRESS plus:  place of residence | Healthcare utilization | The results suggest that the stateless insurance tended to boost utilization of IP admission for its insurees. Overall IP utilization rates increased among children by 14% in 2017 compared with utilization in 2013  **positive** |
| Aizawa 2019 | Indonesia | Observational survey | social health insurance | maternal and neonatal health | pregnant women  PROGRESS plus:  socioeconomic status: | Healthcare utilization | a positive effect on institutional delivery (p < 0:01) and the number of antenatal care visits  during the second (p < 0:10) and third (p < 0:01) trimesters. The noncontributory insurance increases the probability of delivering at a medical facility by 20.3 percentage points.  Positive impact |
| Houweling 2017 | Colombia | Observational retrospective cohort | health insurance | neonates health services | Neonates  PROGRESS plus:  socioeconomic status | Mortality  Healthcare utilization | The crude NMR was lower among babies born to mothers in the contributory scheme (6.13/1000) than among babies born to mothers in the subsidised scheme (7.69/1000) and uninsured mothers (8.38/1000).  **Positive impact**  The fraction of babies delivered through C-section was higher among mothers insured in the contributory scheme (49%) than among mothers in the subsidised scheme (34%) or uninsured mothers (28%)  **Positive impact** |
| Anindya 2020 | Indonesia | Observational survey | NHIS | maternal health services | pregnant women  PROGRESS plus:  socioeconomic status | Healthcare utilization | Enrollment in JKN was  associated with greater prevalence in ANC visits, skilled  birth attendance, facility-based  delivery, and PNC with skilled provider  **Positive impact** |
| Kesuma 2015 | Indonesia | Observational  Survey | JKA) government health inusrance scheme | Maternal healthcare | pregnant women  PROGRESS plus:  socioeconomic status,  place of residence | Healthcare utilization | Utilization of family planning  services increased significantly for all insurance schemes  positive |
| Rivillas 2020 | Colombia | Observational  mixed methods | HI | maternal health services | women  socio-economic status: | Mortality | inequality in maternal mortality across regions and in particular in the subsidized health insurance.. 20% of territories with the lowest health spending per capita have reached 35% of maternal mortality, and it such rates are worsening.  **Negative impact** |
| Rajalakshmi 2021 | India | Observational Qualitative | Public HI | provide free medical and surgical treatment in Government and private hospitals | Any family whose annual family income is less than INR 72000/- (One USD approximately 70 INR) per annum (this paper focuses on women)  socio-economic status, gender | Healthcare expenditure | With poor monitoring by the State of public and private hospitals, combined with low awareness among women on their entitlements under the scheme, women continue to either be excluded or may be inappropriately included  in treatment plans. With very little changes to OOPEs even while using the CMCHIS, the paper documents, women’s lack of trust in, acceptance of, and consequent poor utilization of the scheme. The processes cumulatively push women towards unempaneled private providers, which causes financial stress.  **Negative impact** |
| ServÃ¡n-Mori 2021 | Mexico | Observational (Cross-sectional & retrospective analysis) | Public HI (Seguro Popular programme) | extensive package of health services including  maternal health care (in this study focus on antenatal–postnatal care) | the population without Social  Security support  (in this study vulnerable women  socio-economic status, ethnicity | Healthcare utilization | the implementation of this programme made it possible to improve coverage and access  to a comprehensive package of health services for indigenous populations, thus increasing the odds of continuum-of-care  participation.  Positive impact  Seguro Popular showed that **it could help counteract the effects of structures of inequality in the care of indigenous**  women by reducing the gaps in the continuum of care between affiliated indigenous and non-indigenous women. |
| Solanke 2021 | Nigeria | Observational (Secondary data analysis) | health insurance scheme | Institutional delivery in this study | reproductive age women in this study  socio-economic status | Healthcare utilization  Implementation consideration | strong variations in the prevalence of institutional delivery among childbearing women in Nigeria with higher prevalence among women enrolled in health insurance.  **Positive impact**  Findings in the study also confirm low enrolment of child bearing women in health insurance schemes in Nigeria. This corroborates earlier observations that health insurance enrolment is very low among child-bearing women in developing countries. For the country to benefit optimally from the implementation of health insurance schemes, health planners and authorities must seek new ways of encouraging widespread enrolment in the schemes especially in rural areas and in Norther Nigeria. This may be achieved through sustained public education on the need for enrolment in health insurance schemes in the country in addition to providing initial subscription for people at the lowest economic wealth level |
| Tschirhart 2021 | Thailand-Myanmar border region | Observational Qualitative | voluntary non-profit health insurance | emergency obstetric  care in labor | migrant undocumented women  socio-economic, migrant status | Healthcare expenditure | This fund was well received by participants affiliated with NGOs/non-profits and the Tai healthcare system and was identified as a mechanism that can help decrease institutional debt related to uninsured patients. A non-profit insurance fund for undocumented population can help to improve healthcare entitlements, provide financial protection and reduce service providers’ debt.  **Positive impact** |
| Imo, 2022 | Nigeria | Observational (Cross-Sectional) | National Health Insurance Scheme | antenatal, delivery and postnatal care, | Women  Age | Mortality | larger proportion of dead children was born to mothers who reported not being covered by health insurance and those who had inadequate healthcare services. the risk of U-5M was significantly reduced for children whose mothers reported to be covered by health insurance and those who had adequate healthcare services utilization  **positive impact** |
| Indrasawri, 2021 | Indonesia | Observational (Retrospective analysis) | National Health Insurance Scheme (UHC) | all medical treatment expenses in hospital, including hospital stay fee,  physician fee, and medication during both inpatient and outpatient care | all Indonesian citizens  (in this study focus on children with cancer)  socio-economic status | Healthcare utilization  Mortality  Morbidity | After introduction of UHC, the number of insured patients increased from 38% to 82%. Among low SES population, insurance coverage increased from 40% to 85%, and among high SES population from 33% to 77%  **Positive impact**  the event-free survival estimates at four years after diagnosis of the low SES population improved after introduction of UHC from 14% to 22%  **Positive impact**  In the low SES population, treatment abandonment decreased from 36% to 19%  **Positive impact** |
| Kofinti 2022 | Ghana | observational | National Health Insurance Scheme | antenatal care (ANC), delivery, postnatal care and free neonatal care for up to three months a | women  Place of Residence | Healthcare utilization | women enrolled in the NHIS at the national level were more likely to access maternal health care (delivery care and ANC visits) compared to their counterparts who were not enrolled  **positive** |
| Liyanto 2022 | Indonesia | Observational (secondary data analysis) | National Health Insurance Scheme | JKN is intended to provide health insurance coverage as  well as a social safety net in the form of disability and retirement benefits | women  Socio-economic status | Healthcare utilization | increased numbers of ANC service visits, proportions of deliveries at health facilities and  attended by doctors (and a corresponding decline in home deliveries), and timing of initial  post-delivery checks being closer to the time of delivery  rural women who are enrolled are more likely to use delivery care services compared to their urban counterparts who are also enrolled across the country. This positions the NHIS as an enabling factor in promoting universal access to maternal health care services in Ghana, and as a pro-poor policy in bridging the within-country inequality in access to useful services such as delivery care and ANC utilization. |
| Marthias 2022 | Indonesia | Observational secondary data analysis | National Health Insurance Scheme | comprehensive care including promotive, preventive and curative programmes | Entire population (focus of the study was on mothers, neonates, and infants)  Socio-economic status | Healthcare utilization | Our results suggested that the policy was associated with significant level increase in the overall maternal health service coverage, except for ANC service contact and CS.  The effects of JKN on ANC crude coverage and safe delivery service contact were more pronounced for the lowest economic group than the most affluent population |
| Aziz 2022 | Pakistan | Observational (Survey) | health insurance | Child Health | Children under 5  age | Morbidity | the treatment effect (health insurance) on the  outcome variable (child health) (ATT) shows a significant effect at a 1% significance level indicating that insurance significantly improves the child’s health  Positive impact |
| Bolarinwa 2022 | Nigeria | Observational Survey | health insurance | reproductive health services | Women  gender | Healthcare utilization | Health insurance coverage shows that 25.47% of sexually active women in Nigeria covered by health insurance were using modern contraceptives, while 86.50% of those not covered by health insurance were not using modern contraceptives.  Nigerian sexually active women  who were covered by health insurance had higher odds of using modern contraceptives compared to sexually active women who  had no health insurance coverage in Nigeria.  **Positive impact** |
| Chen 2021  *Heterogeneous Effects of Health Insurance on Rural Children's Health in China: A Causal Machine Learning Approach* | China | Observational Survey | Health Insurance | children health services | children in rural areas  Place of residence and age | Morbidity | URRBMI has significantly improved the nutritional and health status of  insured children in the short-term, medium-term, and long-term  **Positive impact** |
| Diao 2022 | China | Quasi-experimental | Health insurance | cancer treatment | Women  place of residence | Healthcare utilization  Healthcare expenditure | the proportion of patients who adopted trastuzumab for treatment increased from 29.9% before the public health insurance coverage to 61.8% afterwards.  **Positive impact**  The reductions of medical expenditure and share of patient OOP expenditure for HER2-positive breast cancer treatment were attributed to the public health insurance coverage of novel antibreast-cancer medicines.  **Positive impact**  There were differences in the OOP expenditure between the different residents. Rural patients, patients enrolled in urban and rural resident health insurance programme and non-local medical patients were entitled to relatively weak health insurance benefit packages. These patients might be the ones who still had the affordability problem and, thus, might be less likely to benefit from the public health insurance coverage of novel antibreast cancer medicines.  **Disproportionate benefit** |
| Ekholuenetale 2021 | Ghana | Observational survey | Health Insurance | reproductive and MNCH services | women of reproductive age  gender, age | Healthcare expenditure | women from low neighborhood socioeconomic disadvantage status had increased out-of-pocket health expenditures in most maternal healthcare services than women of high neighborhood socioeconomic disadvantage status. women of high neighborhood socioeconomic disadvantage status had the least out-of-pocket expenditure for total healthcare utilization, laboratory investigations, antenatal care visits, post-natal care visits, care for new born for up to 3 months, and other healthcare services.  **Negative impact** |
| Philibert 2017 | Mauritania | quasi experimental | Obstetric Risk Insurance | maternal and perinatal health services | pregnant women  gender | Healthcare utilization  Mortality | the ORI scheme did not cause the expected increase in facility-based deliveries. No significant association between the implementation of the ORI scheme and increased  utilization of maternal health services was detected  **no impact**  the ORI scheme was not found to have a significant positive effect on neonatal mortality  **no impact** |
| Sanogo 2020 | Gabon | Observational Qualitative study | A social health insurance scheme | Antenatal care | Target: Women  PROGRESS plus: socio-economic status and ethnicity | Quality of Care | The findings demonstrated that women had positive experiences in terms of the services they are  able to access and use in a health facility. Moreover, all  women agree that they receive adequate information and  advice about maternal requirements throughout the continuum of care.  According to the women’s perceptions,  The health professionals also professed of the positive effect the NFHISG has had on their ability to provide care to women. However, some participants criticized the long waiting times and the inequity of treatment, with some health professionals accused of favoring and providing better care for privileged patients who pay out of pocket over those who have the NFHISG card. |
| Liabsuetraku2011 | Thailand | Observational  (Survey) | health insurance universal coverage scheme | delivery services | women who gave birth at study hospitals  PROGRESS plus: socio-economic status | Quality of care | Women insured by CSMBS were significantly less likely to perceive a delay and barrier to seeking delivery care. |
| Paredes 2016 | Philippines | Observational  Retrospective cohort | Health insurance | ANC, health facility delivery | **Target group:** Pregnant women  **PROGRESS Plus Measure:** Socioeconomic Status (SES) | Healthcare utilization | The intervention led to an increase in health facility delivery among pregnant women, however no significant impact was observed on the number of ANC visits or cesarean section.  **Inconclusive**  Women who were more educated tended to use more services compared to women who received little or no education. |
| Chayo, 2023 | Colombia | Observational  (Secondary data analysis) | Subsidized insurance scheme for the poor and contributive scheme for the working population | All health services  (Cancer related illness this paper) | **Women**  **Socio-economic status** | Other outcomes (cancer survival) | women living in a low SES residential area (vs. high SES) and who were affiliated to the subsidized tier of the health insurance scheme (vs. contributive) experienced a reduced probability of 5-year cervical cancer survival. These differences may be partially explained by the combined effects of socioeconomic and capital leverage as well as higher functioning health insurance on early procurement of better treatment after initial point of diagnosis  **Inequity in 5-year cervical cancer survival** |
| De Groot, 2023 | Kenya | Experimental  RCT | subsidized mobile phone-based health insurance program | All health services | **Women**  **Socio-economic status and place of residence** | Healthcare utilization  Healthcare expenditure | When living sufficiently close to one of the program-selected clinics (that had participated in the quality improvement program SafeCare), women in treatment villages were significantly more likely to seek health care for their children and for themselves at a formal provider  **Positive effect**  the mobile phone-based, subsidized health insurance coverage was successful in increasing **health insurance enrollment** and reducing **out-of-pocket expenditures** for low-income households in rural Western Kenya  **Positive effect** |
| Garg, 2023 | India | Observational  Survey | publicly funded health insurance (PFHI) | Institutional deliveries | **Women**  **Socio-economic** | Healthcare expenditure | Enrollment under PMJAY or other PFHI was not associated with any reduction in out-of-pocket expenditure or distress financing for caesarean or non-caesarean institutional deliveries across India  **No effect** |
| Langat, 2023 | Kenya | Observational  Retrospective medical records review | The National Hospital Insurance Fund (NHIF) | Inpatient and outpatient care | **Children**  **age** | Other outcomes (cancer survival) | The most likely treatment outcome in uninsured patients was death (49%), whereas in those with health-insurance at diagnosis and those who enrolled during treatment it was event-free survival (36% and 41% respectively). Overall survival and event-free survival were higher for insured versus uninsured patients  **Positive effect** |
| Latif, 2022 | Pakistan | Observational | Micro-insurance | All health services | **Women**  **Socio-economic status** | Implementation considerations  (barrier) | Findings show that distance impedes individuals from making panel (cashless) claims and thus increases the likelihood of out-of-pocket expenditures at nearby non-panel hospitals. This adverse effect is more pronounced for women as compared to men. Dissemination of information in social networks increases the usage of panel facilities, especially by women. Hence, this can be an effective mechanism in reducing the role that distance plays in the choice of health facility |
| Lee, 2023 | Indonesia | Observational  Secondary data analysis | public health insurance | Reproductive health services | **Women**  **Socio-economic status and place of residence** | Healthcare utilization  (Skilled birth attendance) | Insured women living in Java–Bali and in the richest wealth quintile were 6.4 times more likely to be attended by OBGYN/GP and 4.2 times more likely to deliver at a hospital compared with those without health insurance, living in Eastern Indonesia, and in the poorest income quantile. |
| Nasir, 2022 | Benin | Observational  Cross-sectional | Health insurance | All health services | **Women**  **Socio-economic status and place of residence** | Implementation considerations | For women in Benin to optimize the benefits that accrue from taking health insurance, health programmers and planners must look for ways to encourage enrolment into these schemes, particularly among women in the informal and rural parts of the country. Some of the key challenges confronting enrolment into the scheme which include high premium charges and lack of money to pay as premiums should be addressed. The government of Benin should subsidize premiums for poor rural women. |
| Ramos, 2023 | Peru | Observational | government-subsidized health insurance scheme | Maternal health services | **Women**  **Socio-economic status** | Healthcare utilization | Findings from the adjusted logistic regression confirmed that insured women were more likely to have accessed ANC services compared with uninsured women. |
| Tsala, 2022 | Congo | Observational  Cross-sectional | Health insurance | Reproductive health services | **Women**  **Socio-economic status** | Other outcomes  (insurance coverage) | With regard to SES, findings indicated that women of reproductive ages living in better-off households and advantaged neighborhoods had higher chances to own health insurance compared with their counterparts in poor households and disadvantaged neighborhoods. there were important geographical variations regarding health insurance coverage ranging from 1.2% in Bandundu and Kasai Oriental to 15.5% in Kinshasa the Capital City.  **Inequity between poor and rich/rural and urban** |
| Zhang, 2022 | Nigeria | Observational  (survey) | Health insurance | All health services | **Women**  **Socio-economic status, place of residence, and education** | Other outcomes  (insurance coverage) | Disparity exists in health insurance ownership as a higher proportion of those enrolled in health insurance were those with higher education attainment, in urban parts of the country, and those situated on higher wealth quintiles  **inequity** |
