# Supplementary material for: Financing for equity for women’s, children’s and adolescents’ health in low- and middle-income countries: A scoping review
Source: PLOS Glob Public Health. 2024 Sep 12;4(9):e0003573. doi: 10.1371/journal.pgph.0003573 (PMC11392393; doi:10.1371/journal.pgph.0003573)
Supplement: S3 Table — (DOCX) [file pgph.0003573.s006.docx]

**S3 Table of characteristics: user fees exemption (N=75)**

| **Author Year** | **Country** | **Study design** | **Health service covered** | **Target group and PROGRESS Plus**  **measures** | **Outcome(s)** | **Main Results**  **Is the intervention effective overall? (yes/no/inconclusive)** |
| --- | --- | --- | --- | --- | --- | --- |
| Ajayi 2017 | Nigeria | Observational (Surveys) | Maternal health services (institutional delivery, antenatal, skilled birth attendance) | Women  **PROGRESS Plus:**  Socioeconomic status and place of residence | Healthcare utilization | Only 12.9% of women in rural areas benefited from user-fee removal compared to 39.0% of women residing in cities. Only 12% of women belonging to the lowest socioeconomic status compared to 24% of women in high socioeconomic status benefited from user-fee removal in Nasarawa State.  ***Limited impact women of the lowest socioeconomic residing in underserved communities*** |
| Dennis 2020 | Kenya | Quasi-experimental (Interrupted time series) | antenatal care | Women  **PROGRESS Plus:**  Socioeconomic status | Healthcare utilization | User fee reductions in public  primary healthcare facilities was not associated with population-level increases in use of public facility-based ANC among ANC users nor on use of primary care facilities and content of care among users of public facilities.  When disaggregated by wealth groups, the findings further suggest that the 10/20 policy may have been more beneficial to better-off women compared to poorer women.  ***Limited impact on poorer women*** |
| Amoako Johnson 2016 | Ghana | Observational  Correlational | skilled birth care | Women  **PROGRESS Plus:**  place of residence | Healthcare utilization | the national-level the exemption and removal policies had a positive impact on uptake of skilled delivery care when compared to the cash and carry period.  although there was statistically significant increase in uptake of skilled birth care over the policy periods, the uptake remains substantially lower for amongst women with no formal education, those with partners with no formal education, women from the poorest households and rural areas when compared to other women  ***Limited impact women of the lowest socioeconomic, with no education and those residing in rural areas.*** |
| Lamichhane 2017 | Nepal | Quasi-experimental (Controlled before and after | Free delivery services in public facilities | Women  **PROGRESS Plus:**  socio-economic status and ethnicity | Healthcare utilization  Mortality | removal of user fees for deliveries in Nepal’s public facilities increased maternal health service use and lowered the likelihood of neonatal mortality among different sub-groups of women.  ***Positive impact in the initial phase for all women including lower caste and indigenous ethnicity*** |
| Zombre 2019 | Burkina Faso | Observational (Cross-sectional) |  | Children under 5  **PROGRESS Plus:**  place of residence | Healthcare Utilization  Morbidity | the probability of using health services was 17.2% (95% CI 15.0–26.6) higher among children living in the intervention district relative to the comparison district, which rose to 20.7% (95% CI 9.9–31.5) for severe illness episodes.  no significant differences in the probability of health services use according to socio-economic status [χ2 (5)=12.90, p=0.61].  ***Positive impact on utilization***  ***but no impact on reducing the risk of illness occurrence and socioeconomic inequities in the use of health services.***  no significant difference in the reduced  probability of an illness occurring in the intervention district compared to the non-intervention district [AME4.4; 95% CI 1.0–9.8)].  No impact |
| Nkwenti 2019 | Cameroon | Observational (Retrospective and cross-sectional) | Malaria treatment | Children under 5  **PROGRESS Plus:**  age | Healthcare utilization  Mortality | Increase in consultations (by 5.7% (p=0.001) and hospitalization for severe cases  no significant reduction in mortality due to malaria among under-five children (4.4%, p=0.533).  ***Positive impact on utilization but not on mortality*** |
| Obare 2018 | Kenya | Quasi-experimental  interrupted time series | health  facility deliveries | Women  **PROGRESS Plus:**  place of residence and socio-economic status | Healthcare utilization | There were no statistically significant immediate changes in the proportion of births occurring in public  facilities following the 2004, 2007 and 2013 user fee policy shifts among poor or rural women.  ***No impact on poor and rural women*** |
| Ravit 2018  *Removing user fees to improve access to caesarean delivery: a quasi-experimental evaluation in western Africa* | Mali and Benin | Quasi experimental | caesarean delivery | Women  **PROGRESS Plus:**  place of residence, education and socio-economic status | Healthcare utilization  Mortality | The free caesarean policy had a positive impact on caesarean section rates particularly in non-educated women, those living in rural areas and women in the middle-class wealth index). The policy contributes to the increase in the proportion of facility-based delivery and may also contribute to the decrease of neonatal mortality.  ***Positive impact on caesarean section rates in non-educated and rural women.*** |
| Garchitorena 2017 | Madagascar | Observational (cross-sectional) | Maternal and child health services | Women  **PROGRESS Plus:**  place of residence | Healthcare utilization | When fee exemptions were introduced for targeted medicines and services, the use of health care increased by 52% for children under age five, and over 25 percent for maternity consultations.  ***Positive impact*** |
| Fournier 2014 | Mali | Quasi-experimental  (Interrupted time series) | Caesarean section | Women  **PROGRESS Plus:**  place of residence | Healthcare utilization | caesarean rate increased from 0.25 to 1.5% for the entire population. For women living in cities with district hospitals that provided caesareans, the rate increased from 1.7% before the policy was enforced to 5.7% 83 months later.  No significant change in trends was observed among women living in villages with a healthcare centre or those in villages with no healthcare facility. For the latter, the caesarean rate increased from 0.4 to 1%.  ***No impact for women living in rural areas*** |
| Ajayi 2020 | Nigeria | Observational  Correlational | maternal health services | Women  **PROGRESS Plus:**  place of residence, education, socio-economic status | Healthcare utilization | Women who earned a monthly income of ≤20 000 naira (US$150) were 50% less likely to have a birth by CS compared with those who earned more. Compared with women who were educated to the tertiary level, women who had a secondary education or less were 51% less likely to give birth by CS.  ***No impact for low socio-economic and less educated women*** |
| Hatt 2013 | Low income countries | Literature review | Maternal health services | Women  **PROGRESS Plus:**  socio-economic status | Healthcare utilization  Mortality | The introduction of user fee exemptions appears to have resulted in increased rates of facility-based deliveries and caesarean sections in some contexts.  **Positive impact**  Impacts on maternal and neonatal mortality have not been conclusively demonstrated; exemptions for delivery care may contribute to modest reductions in institutional maternal mortality but the evidence is very weak.  **inconclusive**  unintended consequences for provider motivation and quality of care.  ***Limited impact on the poor*** |
| Ganle 2014 | Ghana | Descriptive study | maternal health services | Women  **PROGRESS Plus:**  socio-economic status, education and place of residence | Healthcare utilization | More urban women (40%) than rural, 53% more women in the highest wealth quintile than women in the lowest, 38% more women in the best performing region (Central Region) than the worst (Upper East Region), and 48% more women with at least secondary education than those with no formal education, accessed and used all components of skilled maternal health services in the five years preceding the survey.  ***Limited impact on the poor, less educated and rural women*** |
| Brault 2017 | Kenya | Descriptive (Case study) | Child health services | Children  **PROGRESS Plus:**  socio-economic status | Healthcare utilization | while removing user fees does increase uptake, inequities remain due to other barriers including out-of-pocket costs that include lost wages, travel, food and accommodations during hospitalization or care of a sick child.  Limited impact on the poor and challenges related to lack of resources and strain on existing resources due to increased patient volume |
| El-Khoury, 2012 | Mali | Observational (Survey) | Caesarian section deliveries | Women  **PROGRESS Plus:**  socio-economic status | Healthcare utilization | Fewer women in the poorest two-fifths of the population are receiving caesareans than what we would expect given their share in the population of women giving birth.  ***Limited impact on poor women*** |
| Tripathi 2014 | India | Observational (Secondary data analysis) | delivery | Women  **PROGRESS Plus:**  Place of residence | Healthcare expenditure | Out-of-pocket expenditure for delivery decreased from Rs. 5342 to Rs. 3565 between pre and post-intervention period. There was no significant difference in catastrophic health expenditures between pre-JSSK (21.2%) and post-JSSK (15.6%) periods (P=0.15).  ***Positive impact on out-of-Pocket expenditure*** |
| AbdouIllou 2015 | Burkina Faso | Observational (Survey) | Child health services consultations and medications | children under five  **PROGRESS Plus:**  Place of residence | Healthcare expenditure | The intervention benefited both poor and non-poor families and provided financial protection  ***Positive impact on the poor*** |
| Ravit 2018  *Do free caesarean section policies increase inequalities in Benin and Mali?* | Benin and Mali | Observational (Secondary data analysis) | c-section and facility-based delivery | women  **PROGRESS Plus:**  Place of residence and socio-economic status | Healthcare utilization | we found no significant change in access to C-section and FBD after the introduction of the free C-section policy.  ***No impact for the poor*** |
| Witter 2018 | Sierra Leone | Quasi-experimental (time‐series) | Maternal and child health services | Women and children  **PROGRESS Plus:**  Place of residence and socio-economic status | Healthcare utilization | Free Health Care Initiative was one important factor contributing to improvements in coverage and equity of coverage of essential services for mothers and children.  ***Positive impact*** |
| Parmar 2019  *How do supply- and demand-side interventions influence equity in*  *healthcare utilisation? Evidence from maternal healthcare in Senegal* | Senegal | Observational  Correlational | Maternal health services | Women  **PROGRESS Plus:**  Place of residence, education and socio-economic status | Healthcare utilization | When compared to supply-side (increasing availability of services), abolishing user benefited the poor more (reduced inequities) in utilization.  ***Positive impact for the poor but the rich still have higher utilization*** |
| Ferry 2012 | Tanzania | Observational Surveys | Obstetric and pediatric services | women  **PROGRESS Plus:**  Place of residence | Healthcare utilization | Overall, the richest 20% of the population accounted for 29.7% of admissions to the rural health centers whereas the poorest 20% of the population accounted for only 15.3% of admissions.  ***Positive impact on utilization but inequities persist.*** |
| Zombre 2017 | Burkina Faso | Quasi-experimental  controlled interrupted time-series analysis |  | children under five  **PROGRESS Plus:**  Place of residence | healthcare utilization | The initiation of the intervention more than doubled the utilization rate with an immediate 132.596% increase in intervention facilities. The effect of the intervention was 32.766% higher in facilities with higher workforce density and during the rainy season, but not significant in facilities with higher dispersed populations. Although the intervention effect was substantially significant immediately following its inception, the pace of growth, while positive over a first phase, decelerated to stabilize itself three years and 7 months later before starting to decrease slowly towards the end of the study period.  ***Positive impact on utilization but not sustainable*** |
| Chama-Chiliba 2016 | Zambia | Observational  logistic regression | facility-based deliveries in public health facilities | women  **PROGRESS Plus:**  Place of residence | healthcare utilization | The difference-in-difference estimates point to statistically insignificant changes in the proportion of women  giving birth at home and in public facilities, but significant changes are found for deliveries in private (faith-based)  facilities.  ***No impact on utilization in public health facilities*** |
| Edu 2017 | Nigeria: | Observational  mixed method | Maternal heath services | women  **PROGRESS Plus:**  Place of residence | Healthcare utilization | Results suggest weak evidence of change in maternal health care service utilization.  ***Limited impact*** |
| Lang'at 2019 | Kenya | Quasi-experimental (interrupted time series analysis) | Maternal heath services | women  **PROGRESS Plus:**  Place of residence | Healthcare utilization  Mortality | Significant sustained increase of 89, 97, and 98% was observed in the antenatal care visits, health facility deliveries, and live births respectively,  No significant changes were observed in the stillbirth rate and caesarean section rate  ***Positive impact on utilization but no impact on still birth*** |
| Wilkinson 2001 | South Africa | Observational  Correlational | Women and child health services | children and women  **PROGRESS Plus:**  Place of residence | Healthcare utilization | There was a sustained increase in new registrations (P = 0.0001) and total attendances (P = 0.0001) for curative services, and a fall in new registrations (P = 0.01) and total attendances for immunization and growth monitoring (P = 0.0002) over the study period. The upturn in demand for curative services started at the time of the first policy change. The decreases in antenatal registrations (P = 0.07) and attendances (P = 0.09) were not statistically significant.  ***No impact*** |
| Leone 2016 | five sub-Saharan countries | quasi-experimental analysis | Facility-birth and C-section | children  **PROGRESS Plus:**  Place of residence and socio-economic factors | Healthcare utilization | User fee reforms are associated with a significant percentage of the increase in access to facility births (27 percentage points) and to a much lesser extent to CS (0.7 percentage points). Poor (but not the poorest), and non-educated women, and those in rural areas benefitted the most from the reforms.  **Positive impact on facility birth but not on C-section** |
| Ridde 2013 | Burkina Faso | Quasi-experimental | children | children under five  **PROGRESS Plus:**  Place of residence | Healthcare utilization | The exemption benefited the children of poor families when health need was high and services near (RR=5.23; (1.30 to 20.99)).  ***Positive impact for the poor when services is near and health need high*** |
| Ridde 2015 | Burkina Faso | Observational  correlational | facility-based deliveries | Women  **PROGRESS Plus:**  Socio-economic status | Healthcare expenditure | The Burkinabe` policy led to a significant and sustained reduction in household OOP health spending across all socio-economic groups, but failed to properly target the poorest by ensuring a progressive payment system.  ***Positive impact among the poor*** |
| Owiti 2018 | Kenya | Observational (cross-sectional study) | maternal health services in public facilities | Women  **PROGRESS Plus:** place of residence | Implementation considerations  facilitator | Factors favoring the Free Maternal Service uptake included: a positive perception of the public health facility, living within close proximity, learning about the Program from a support group and a short waiting time before being examined by the doctor.  safe delivery, quality of service, accessing a health facility on foot, ANC attendance at a private and a non-profit health facility were associated with low uptake of the free maternal services.  **Distance, quality of care, awareness of the policy were the main factor affecting utilization** |
| Ponsar 2011 | Mali | Observational  Correlational | Malaria treatment | Children and pregnant women  **PROGRESS Plus:** socio-economic status | Healthcare utilization  Mortality | Removing user fees for vulnerable groups significantly improves utilization and coverage of essential health services, including for malaria interventions  findings indicate that in areas where services were underused, mortality was higher. In areas where the MSF intervention led to an increased uptake of essential health services, mortality was found to be reduced within the general and the under-5 population.  ***Positive impact on utilization and mortality*** |
| Ridde 2009 | Niger | Observational  mixed method | Maternal and child health | Children under five and pregnant women **PROGRESS Plus:** age and gender | Implementation considerations  facilitator | Enabling factors: a top-down phase of information and raising community awareness; appropriate incentive measures; a good drug supply system; and the organization of a medical evacuation system  Concerns were voiced about sustainability, the poor coordination of the availability of free services at different levels of the health pyramid. |
| Dzakpasu 2012 | Ghana | Quasi experimental  (time-series methods) | maternal health services | pregnant women  **PROGRESS Plus:** socio-economic status (the poor) | Healthcare utilization  (Facility delivery) | The increases in facility delivery were greatest among the poorest, leading to a decline in socioeconomic inequality.  ***Positive impact*** |
| Dalinjong 2018  *Are health facilities well equipped to provide basic quality childbirth services under the free maternal health policy? Findings from rural Northern Ghana* | Ghana | Observational  mixed method | maternal health services | pregnant women  **PROGRESS Plus:** place of residence (rural) | Implementation considerations | **Facilitators**: Reasonable waiting times, cleanliness of facilities as well as good interpersonal relationships with providers  **Barriers**: out of pocket payments (cost of drugs, transportation, lab tests), lack of, or inadequate supply of drugs and commodities, equipment, distance, water, electricity and emergency transport. |
| Ansah 2009 | Ghana | experimental  RCT | PHC services including diagnosis and drugs | Children under 5  **PROGRESS Plus:** socio-economic status  place of residence | Other outcomes  *Health care-seeking behavior* | the removal of out-of-pocket payments for health care changed health care-seeking behavior in children.  ***Positive impact on healthcare seeking behaviors*** |
| Nagpal 2019 | Lao PDR | Observational Secondary data analysis |  | Pregnant women  **PROGRESS Plus:** Ethnicity & socioeconomic status | Healthcare utilization  Healthcare expenditure | In 2013, the institutional births in the intervention sites accounted for 32.7% of all deliveries, up from 18% in 2010.  **Positive impact**  The DiD analysis did not find any statistical effect in reducing average OOP expenditure, although there was a reduction in OOP MH expenditure for mothers who delivered at a health centre, both at intervention and control sites  **No impact** |
| Asante-Sarpong 2016 | Ghana | Observational Cross-sectional | Normal deliveries and the management of all assisted deliveries including caesarean sections | Pregnant women  **PROGRESS Plus:** Place of residence | Implementation Consideration  facilitator | The results identified religion, parity, place of residence and maternal age as statistically significant predictors of delivery service use. In addition, awareness and full knowledge about the free maternal healthcare policy were also found to be statistically significant predictors of delivery service use. Mothers living in urban areas were 3.793 times more likely to use delivery services under the policy than those living in rural areas |
| Ameur 2012 | Burkina Faso | Observational Survey | total cost of the medical procedures, drugs and consumables, and observation | Women  **PROGRESS Plus:** place of residence & socioeconomic status | Healthcare expenditure | Medical expenses are lower and the distribution is reduced for all the sample women in Sebba, where there is a full exemption  **Positive impact** |
| De Allegri 2011 | Burkina Faso | Observational Survey | All ANC-related services and drugs | Pregnant women  **PROGRESS Plus:** place of residence | Implementation Consideration | Living within 5 km from a health facility was positively associated  with ANC utilisation, while traditional African religion, ethnicity (specifically being Samo or Marka), and higher levels of household wealth were all negatively associated with  ANC utilisation.  Ethnicity (specifically being Mossi,  Peuhl, Marka, or Other), living within 5 km from a health  facility, having attended at least 3 ANC visits were all positively associated with delivering in a health facility |
| Ganle 2019 | Ghana | Observational retrospective analysis of secondary data | All women are entitled to a “Maternal Benefit Package” | all women  **PROGRESS Plus:** Place of residence | Implementation considerations  Healthcare expenditure | findings from this study indicate that although maternal health care services including skilled facility delivery may be free in Ghana, a number of health system, socio‐cultural, and individual level factors still drive women towards home delivery (The results and discussion in this paper suggest that unless interventions are concurrently implemented to address other health system factors like insurance coverage, and socio‐cultural factors like cultural and religious beliefs that hinder uptake of skilled delivery services, the full benefits of user‐fee exemption for maternity care may not be realized in contexts like northern Ghana.)  In addition to expanding skilled birth services to hard‐to‐rich areas, new interventions such as supervised domiciliary delivery services, community mobilization and engagement, especially male involvement, should be considered.  Even though skilled delivery services including caesarean section are free in Ghana, costs that may be incurred during delivery may include supplies and medications, transportation, unofficial provider fees, as well as the opportunity costs of travel time and waiting time lost from productive activities. These are costs not covered under the current user‐fee exemption policy |
| Watson 2016 | Malawi | quasi-experimental study | maternal, neonatal, and  HIV services | women  **PROGRESS Plus:** Place of residence | Healthcare utilization | the introduction of user fees led to large, significant declines in outpatient attendances, which also translated into an indirect effect of reductions in new diagnoses of malaria and HIV. The removal of user fees largely reversed this effect.  **Positive impact** |
| McKinnon 2015 | Senegal | Observational Survey | delivery services (including normal delivery services, Caesarean section, and complications during pregnancy and labor) | Pregnant women  **PROGRESS Plus:** Socioeconomic status, education | Healthcare utilization  Implementation consideration | There were absolute increases in facility births across categories of all three socioeconomic variables.  **positive**  effects of the policy change were stronger among more educated women. The policy change was associated with increases of 4.6 facility deliveries per hundred live births (95% CI: 2.2, 7.0) for women with no education and 8.6 per hundred live births (95% CI: 5.4, 11.9) for women with at least some secondary education. |
| Witter 2016 | Benin, Burkina Faso,  Mali and Morocco. | Descriptive  case-study | Deliveries (normal, C-section) | pregnant women  **PROGRESS Plus:** Socioeconomic | Healthcare utilization  Healthcare expenditure | It is evident overall that countries have made progress over the past 15–20 years, and at best these policies may have contributed to maintaining the momentum, but there is no statistical evidence of that as yet. It is almost certainly too early to tell, as we have only 2–3 post-policy data points in each country, and the varying implementation documented by the research also underlines the need to be cautious about assuming immediate effectiveness of policies  **Inconclusive**  Looking at the difference between recorded payments prior to the policies and average payments now, households have made a substantial financial gain. In Burkina Faso, there was a reduction of 71 % for deliveries of all kinds. In Morocco, the gain was lower for normal deliveries (62 %), compared to 92 % for caesareans. The estimated saving for caesareans in Benin was in the region of 74 %, compared to 78 % for Mali.  **Positive impact**  In all three countries for which there was recent household survey data, the relative inequity between the poorest and the richest had declined over time (in that there have been bigger gains among the poorest). |
| Manthalu 2016 | Malawi | Modelling study | maternal and neonatal health care services | pregnant women  **Progress plus:**  place of residence and socioeconomic status | Healthcare utilization | User fee exemption led to a 15% increase in the mean proportion of women who made at least one antenatal care (ANC) visit during pregnancy, a 12% increase in average ANC visits and an 11% increase in the mean proportion of pregnant women who delivered at the facilities. **Positive impact** |
| Eze 2020 | Nigeria | Observational |  | pregnant women and children less than 5 years old  **PROGRESS Plus:**  socio-economic status and place of residence | Healthcare expenditure | No statistically significant difference in the benefit incidence a free Maternal and Child Health programme and OOP expenditure between the urban and rural dwellers and across socio-economic groups.  Limited impact on the poor and rural residents |
| Nguyen 2018 | Burkina Faso | quasi-experimental  controlled interrupted time series | facility-based delivery | Women  **PROGRESS Plus:**  Place of residence and socio-economic status | Healthcare utilization | Our study detected a significant positive sustained effect of both user fee reduction and removal on the use of facility-based delivery in Burkina Faso.  ***Positive impact*** |
| Ruhago, 2011 | Tanzania | Observational (Survey) | Free of charge ITNs | children under 5  **PROGRESS Plus:**  socioeconomic | Healthcare utilization | Ownership of ITNs increased from 29% in the 2007/08 national survey to 90% after the roll out of free ITNs in Mpanda, and use increased from 13% to 77%  **Positive impact** |
| Zombre 2021 | burkina faso | Quasi-experimental design | maternal and child healthcare, immunization, preventive and curative care, medical management of malnutrition | children under 5 years of age  **PROGRESS Plus:**  socioeconomic | Morbidity | the intervention may have had a positive impact on children’s nutritional status in some communities that our study design did not allow us to identify, our results show that the presence of the intervention was associated to some extent with the variance of the distribution of wasting and stunting in the communities.  Positive impact |
| Penfold, 2007 | Ghana | Quasi-experimental (Controlled before and after) | maternal health services | Women  **PROGRESS Plus:**  place of residence, education, socio-economic | Healthcare utilization | Results from  Central Region showed increases in facility deliveries mainly occurred in health centres (from  13.7% to 22.3% of deliveries), and were attended  by midwives (from 49.0% to 59.7%)  ***Positive impact*** |
| Samadoulougou 2022 | Burkina Faso | Observational (Secondary data analysis) | children  under 5 years of age: any consultation  Women: pre-and  post-natal consultations, and deliveries. | Women and under-5 children  **PROGRESS Plus**: socio-economic status | Healthcare utilization | Following the implementation of the  FHCP in the second quarter of 2016 for children under  five years of age, the inequalities between rich and poor healthcare seeking decreased in 2017–18. Tis could be explained by, among other things, the reduction of the affordability of care, thus allowing access to healthcare  for under-5 children from poor households  **positive impact** |
| Sunny 2021 | Nepal | quasi-experimental | a free newborn care program which reimbursed the  cost of treatment for all sick newborns admitted in public hospitals in Nepal | Newborns  **PROGRESS Plus**: age | Healthcare expenditure | The FNC program was implemented with the intention to overcome the burden of out of pocket expenditure on sick newborn treatment in the hospital. However, we  found no significant change in the OOPE for treatment  of sick newborn in the hospital before and after implementation of FNC. Since, The coverage of this package is not exhaustive to include diagnostics like PT/INR (Prothrombin Time/International  Normalised Ratio), reimbursement for blood products, baby diapers and surfactants for Respiratory Distress Syndrome (RDS) which has to be purchased out-of-pocket in many cases.  **No impact** |
| Paudel 2021 | Nepal | Observational  Cross-sectional | Special newborn care unit /  Neonatal intensive care unit (NICU / SNCU) | Newborns  **PROGRESS Plus**: age | Morbidity | FNC is effective in the management sick newborns  **Positive impact** |
| Karra, 2022 | Malawi | Experimental  RCT |  | married women of reproductive age  **PROGRESS Plus**: Place of Residence | Healthcare utilization | Contraceptive use after 2 y of intervention exposure increased by 5.9 percentage points, mainly through an increased use of contraceptive implants.  **Positive impact**  In addition to the increase in contraceptive use, we find that the |
| Lagarde 2022 | Zambia | Observational  Secondary data analysis | Antenatal services | Women who have had children  **PROGRESS Plus**: Place of Residence | Mortality  Healthcare utilization | We find no evidence that the increase in  institutional deliveries translated into a reduction in neonatal deaths  **no impact**  women from the poorest quintiles benefited directly from user fee removal, with an 18 percentage points increase in institutional deliveries in rural districts and 22 percentage points in peri-urban areas. By contrast, there was no change in the choices of women from the richest quintile in either group, as most women in these groups were already delivering in facilities before the policy change. In both rural and peri-urban areas, user fee removal led to a large increase in the likelihood of delivery in facilities (assisted and not) for women completing at least primary education, but not for those with no or incomplete primary education.  **Positive impact** |
| Muchiri 2021 | Kenya | Modelling Study | a free secondary education (FSE) program | Adolescents  **PROGRESS Plus**: Socioeconomic Status + Age | Other outcomes  *Teen pregnancy* | a decrease of approximately 4–5 percentage points in teenage motherhood as a result of the FSE policy—approximately a 12% drop. The magnitude of these results demonstrates the consequences of the cost of education in settings where the cost is prohibitive, especially for low-income households. I presume that if the government increases these subsidies, then teenage motherhood might decrease further. Teenage motherhood has immediate and lasting consequences for girls' health, education, and income potential.  **Positive impact** |
| Beaujoin 2021 | Burkina Faso | Observational  qualitative | breast and cervical cancer screening, prenatal and postnatal care, deliveries and c/s, universal healthcare for children under 5. | women and children under 5  **PROGRESS Plus**: age | Healthcare utilization | the user fee exemption policy contributes to improving access to reproductive care and family planning by facilitating the negotiation processes between women and their families within households. However, social norms and gender inequalities still limit women’s decision-making power. Which highlights the necessity of improving the women’s decision-making powers when it comes to health and family planning issues |
| Gunarathne 2021 | Sir-lanka | Observational  Cross-sectional Study | MCH services | pregnant women  **PROGRESS Plus**: gender | Healthcare expenditure | There is a statistically significant difference in direct non-medical OOPE between pregnant women who used only the government’s free health services and those who used private health services  **Positive impact** |
| Ochieng 2022 | Kenya | Observational  Qualitative | MCH services | most vulnerable women, newborns and infants  **PROGRESS Plus**: Socio-economic status & gender | Healthcare expenditure  Quality of care | Helped ease economic burden of health care for households to ensure that everyone could access care while  ensuring their autonomy in choosing the service  provider of their choice.  **Positive impact**  It enhanced health facility capacity to handle the patients’ needs and thus regained confidence among consumers, during pregnancy, childbirth, to the expiry date, convinced that delivery at the health facility is much safer as complications can be effectively handled. There was substantial quality improvement, environment, equipment, doctors and essential commodities were available, processes and outcomes. It appears to have transformed the attitude of consumers concerning going to health facilities for skilled delivery  **Positive effect** |
| Orangi 2021  *Impact of free maternity policies in Kenya: An interrupted time-series analysis* | Kenya | Quasi-experimental | MCH services | women & children  gender & age | Healthcare utilization | **The 2013 free-maternity** policy led to a 19.6%  and 28.9% level increase in normal deliveries and  caesarean sections, respectively, in public facilities  **positive**  **the 2017 Linda Mama programme** showed  a level decrease then a trend increase in PNC visits and a 1.1% trend decrease in caesarean sections in public facilities. In private and faith-based facilities, there was a reported level decrease in normal deliveries and caesarean sections and a trend increase in caesarean sections.  The free maternity policies show mixed  effects in increasing access to maternal health services |
| Orangi 2021  *Examining the implementation of the Linda Mama free maternity program in Kenya* | Kenya | Observational  mixed method | MCH services | women & children  socio-economic status & gender | Implementation considerations | although the Linda Mama program was aimed at extending financial risk protection, there were instances  where patients paid OOP either because of fees charged at healthcare facilities or they were asked to purchase items  out of the facilities due to their unavailability. This could be the result of the aforementioned perverse incentives created by the perception that the payment rates are low, driving healthcare providers to charge for the costs incurred during service provision not covered by the reimbursement |
| Oyugi 2021 | LICs & LMICs | Narrative/literature review | MCH services | Women  socio-economic status & gender | Healthcare expenditure  Morbidity | Our review elucidated adequate evidence of the financial implications of the FM policies on patients, providers and managers, and facilities. While the goals of the policy are to improve access to skilled birth and protect mothers from financial catastrophe, the review has shown that the free policies have, in most cases, reduced family expenditure on healthcare; however, they have not eliminated OOP payment.  **Limited impact**  Our findings reveal that FM policies are associated with positive outcomes of evidence-based practices such as  improved screening for key diseases such as syphilis and HIV in pregnancy, measuring vital statistics at ANC, reducing perinatal  complications, and improving immunization  **positive impact** |
| Meda IB 2019  (additional resources) | Burkina Faso | Observational  cross-sectional | RHS | women and children under five  gender, age | Healthcare expenditure | The study showed that a significant proportion of  women continue to pay for deliveries and EmOC (emergency obstetric care), services that should be completely free of charge. These  OOP payments were largely explained by drug and  consumable stock-outs and drug prescriptions that were  not available at the health facilities’ pharmacies.  **Limited impact** |
| Ridde 2012  (additional resource)  *The evaluation of an experiment in healthcare user fees*  *exemption for vulnerable groups in Burkina Faso* | Burkina Faso | Modelling study | Maternal and child services | children under the age of five years and indigent women  socio-economic status, age | Healthcare utilization  Healthcare expenditure  Quality of care | the percentage of women who delivered in CSPSs (country’s health and social promotion centers) doubled, whether they lived close to or far from maternity, and whether they were poor or less poor.  the percentage of children consulting a CSPS for first-line  treatment increased from 25% to 72% for the poorest populations and from 41% to 83% for children living more  than 10 kilometres from a CSPS.  **Positive impact**  The experiment reduced illness-related expenditures for  households. One year later, the majority of households affirmed that they had not spent any money on care for their children (57% to 67%), whereas this rate had been only 6% to 9% before the experiment.  the percentage of women with excessive expenditures was half the size in districts where the experiment was implemented (100% exemption) versus the district that only applied the national policy (80% subsidy)  **Positive impact**  In a context of perfect EGD (Essential Generic Drugs) availability where patients were no longer facing a financial barrier, some expressed fears regarding the possible impacts of this experiment on the  quality of medical prescriptions. For the children targeted by the experiment health workers improved their prescription practices by reducing the use of antibiotics by 62%. Likewise, they reduced the use of injectables in cases of acute respiratory infection by 72%.  Parturients who delivered in the experiment’s districts rated the quality of care more favourably than those in Djibo where they continued to pay  **Positive impact** |
| Shrestha 2018 | Nepal | Descriptive (Case study) | Newborn Care Services | Women  **PROGRESS Plus:**  socio-economic | Implementation  Considerations | The main challenges faced in implementation reported were **lack of infrastructure and human resources** to provide services and the **reimbursement** is not enough. |
| Kruk 2008 | Tanzania | Observational  Correlational | Delivery services | Women  **PROGRESS Plus:**  place of residence | Healthcare expenditure | 73.3% of women with facility delivery reported having made out-of-pocket payments  for delivery-related costs with transport costs (53.6%) and unofficial provider fees (26.6%) were the largest cost components in government facilities.  ***No impact on out-of-pocket*** |
| Ansu-Mensah 2021 | sub-Saharan Africa | Literature review | maternal healthcare | Women  **PROGRESS Plus:**  socio-economic | Quality of care | The study result revealed that the majority (93.3%) of the included studies indicate the quality of maternal healthcare was poor under the free financing policy era.  Reported factors contributing to the perception of poor maternal healthcare included: late  reimbursement of funds, heavy workload of providers, lack of essential drugs and stock-out of medical supplies, lack of policy definition, out-of-pocket payment, and inequitable distribution of staff  ***Negative impact on quality of care as perceived by providers and manager.*** |
| Dalinjong 2018  *The implementation of the free maternal health policy in rural Northern Ghana: synthesised results and lessons learnt* | Ghana | Observational (Survey) | childbirth services | Women  **PROGRESS Plus:**  place of residence | Implementation  Considerations | There is lack of and inadequacy of basic essential inputs including clean water, electricity, emergency transport, drugs, supplies, equipment as well as privacy. While women were satisfied with care, health providers were not due to health systems challenges. |
| Witter 2007 | Ghana | Observational (Qualitative) | Delivery Care | Women  **PROGRESS Plus:**  socio-economic status | Implementation considerations | Staff workloads increased as more women attended, and levels of compensation for services and staff were important to the scheme’s acceptance.  overloaded systems and poor capacity |
| Philibert 2014 | Burkina Faso | quasi-experimental design | Delivery care | women  **PROGRESS Plus:**  Place of residence | quality of care | Women’s satisfaction loaded satisfactorily on a three-dimension principal component analysis (PCA): 1-provider-patient interaction; 2-nursing care services; 3-environment  ***Positive perception on quality of care and*** no difference in perceived quality of care among women from different economic classes. |
| Ajayi, 2023 | Nigeria | Observational  mixed method | Delivery services | Women  Socio-economic | Implementation considerations | individual, sociocultural and health facility–related factors influenced the decision of women to give birth at home despite the free maternal healthcare policy. The primary reasons were lack of money, birth unpreparedness, lack of transportation, unsupportive husband, perceived non-necessity of a skilled birth attendant and health workers’ strikes |
| Browne, 2022 | Burkina-Faso | Observational  mixed method | Family planning | Women  Socio-economic status and age | Implementation considerations | it seems particularly important to increase awareness or knowledge of the Family planning policy among vulnerable groups such as adolescents, unmarried women, and women of lower socioeconomic status. These strategies should go beyond disseminating messages on the radio, either officially or informally. It also appears that misconceptions about FP persist, and that is still sometimes confused with abortion. |
| Furechi, 2023 | Kenya | Observational |  | Women  Socio-economic status | Implementation considerations | There was moderate level of women’s awareness of free maternal healthcare services that resulted in depressed utilization. |
| Gunarathna,  2023 | Sri-Lanka | Observational |  | Women  Socio-economic status and place of residence | Healthcare expenditure | Despite the free maternal policy, the OOP expenditure for ANC is high in rural Sri Lanka, and the low-income group reports a higher OOP expenditure for ANC  **Negative effect** |
| Ilboudo, 2023 | Burkina Faso | Quasi-experimental  Interrupted time-series |  | Women and children  Socio-economic status | Healthcare utilization  Healthcare expenditure | the user fee removal policy significantly increased the use of healthcare facilities for child consultations, use of health facilities for assisted deliveries, complicated deliveries, and second antenatal visits  **Positive effect**  The user fee removal policy has further reduced costs borne by households compared to cost levels before the introduction of the free health care policy. It has even eliminated delivery costs and care costs for children  **Positive effect** |
| Nzali, 2023 | Cameroon | Observational  (cross-sectional) |  | Children  Socio-economic status | Implementation considerations | Despite the current policy on free malaria treatments for children under 5 years old in health facilities, most guardians of children with malaria remain reluctant to seek adequate treatment in health facilities. This delay is found to be determined by social and economic factors such as educational and income levels of guardians |
