# Supplementary material for: Financing for equity for women’s, children’s and adolescents’ health in low- and middle-income countries: A scoping review
Source: PLOS Glob Public Health. 2024 Sep 12;4(9):e0003573. doi: 10.1371/journal.pgph.0003573 (PMC11392393; doi:10.1371/journal.pgph.0003573)
Supplement: S4 Table — (DOCX) [file pgph.0003573.s007.docx]

**S4 Table of characteristics: Vouchers (n=70)**

| **Reference** | **Country** | **Study design** | **Health service covered** | **Target group and PROGRESS Plus**  **measures** | **Outcome(s)** | **Main Results**  **Is the intervention effective overall? (yes/no/inconclusive)** |
| --- | --- | --- | --- | --- | --- | --- |
| Mehboob, 2015 | Africa, South East Asia, Latin America, Pakistan | Systematic review | Reproductive health services | Target group: Women  PROGRESS plus:  Socio-economic status | Healthcare Utilization | Given the utilization of the RH services in the areas where vouchers were introduced, a significant amount of evidence was found, Showing an increase in the use of healthcare services.  **Positive Impact**  Voucher programs seem to be more effective in reaching out  Poorest of the poor indicating the dismal need.  Accessibility of quality healthcare services  at lower cost, or in some cases even free of cost is  one of the prominent features of the demand-side  financing which makes it possible to increase the  outreach to the population to be served  **Positive Impact** |
| Bellows 2011 | Bangladesh, Cambodia, China, Kenya, Korea, India, Indonesia, Nicaragua Taiwan, and Uganda. | Systematic review | Reproductive health services | Target group: Women  PROGRESS plus: Socioeconomic status | Healthcare Utilization | The most commonly examined outcome was increased utilization of RH services. Two of the five utilization studies were cross-sectional reviews with positive results – voucher users receivers having higher utilization than no voucher users receivers or voucher areas having greater utilization than control areas. In Uganda there was a nonsignificant increase in utilization of STI services after the program was initiated in the general population; however, a significant increase was found among the poor  Located within 10 km of contracted health facilities.  **Positive Impact especially among the poor** |
| Eva 2015  Vouchers for family planning and sexual and reproductive health services: a review of voucher programs involving Marie Stopes International among 11 Asian and African countries | **Sub-Saharan Africa:** Uganda Kenya, Madagascar, Ethiopia, Zimbabwe, Malawi , Sierra Leone  **Asia**: Vietnam, Cambodia, Pakistan and Yemen | Observational study | Family planning and reproductive health services | Target : women  PROGRESS plus: Socioeconomic status | Healthcare Utilization  Quality of care | Evidence from all countries included in this review  Indicates that voucher schemes can increase the uptake of contraceptive services.  **Positive impact on utilization**  Outcomes related to reaching specific groups included Increased use of services by poor women and young people.  **Positive impact on reaching poor and young women**  Overall, voucher clients were satisfied with the  service they received, although comparisons in Sierra  Leone found that voucher clients – although  overwhelmingly satisfied – were less satisfied than  Non-voucher client**s.**  **inconclusive** |
| Njuki 2013 | Kenya | Observational (Survey) | Reproductive health services | Target: pregnant women  PROGRESS plus: socio-economic status | Healthcare utilization  Quality of care | The voucher scheme increased the utilization of maternal health services while not ever sued for family planning services  **Positive impact on maternal services but on family planning services**  The vouchers encouraged facilities enhance the comfort of their clients through improved meals, provision of warm bathing water, hot drinks and increased number of beds.  **Positive impact** |
| Schmidt 2010 | Bangladesh | Observational (Survey) | Maternal health services including ANC, facility-based delivery, PNC and cash benefits | Target: Women  PROGRESS plus: Socio-economic status | Healthcare utilization  Quality of care | Institutional deliveries increased by two and a half folds in voucher areas  **Positive Impact**  The voucher program increased the burden on public facilities in terms of workload  **Negative Impact** |
| Prata 2009 | Low, middle-income countries | Systematic review | Maternal health services | Target: pregnant women  PROGRESS plus: socio- economic status and place of residence | Healthcare utilization  Quality of care | Evidence suggests that the pilots have stimulated the use of reproductive health services by those who received the vouchers, and that the distribution of the vouchers has benefited mostly the poor.  **Positive impact**  By giving poor women a choice of provider, vouchers improve the quality of care from the bottom up, rather than from the top down.  **Positive impact**  The downside of OBA is possible ‘fraud and abuse’. Dishonest providers might claim reimbursement for fictitious patients and/or not provide the required quality of services for the price paid.  **Negative impact on fraud and abuse** |
| Ali 2018 | Pakistan | Observational (Prospective cohort) | Reproductive healthcare services | Target:  Married women of reproductive age  PROGRESS plus: socio-economic status, age, gender and place of residence | Healthcare utilization  Quality of care | There was lower contraceptive discontinuation observed compared with national trend.  **Positive impact**  Satisfaction with service through the vouchers was high, with a 90% satisfaction rate.  **Positive impact** |
| Pilasant 2016 | Myanmar | Observational (Survey) | Maternal health services | Target: pregnant women and newborns  PROGRESS plus: socio-economic status | Healthcare utilization  Quality of care | The utilization of most of the MCH services increased over time; in particular, delivery  by SBAs increased significantly (P < 0.01) after implementing MCHVS.  For infant’s first immunization, the data showed irregular  increases in numbers during alternating months due to  the immunization schedule of some facilities  **Positive Impact**  it was found that pregnant women who had experiences  with using vouchers and services provided in this  the program was satisfied in terms of free services and  compensation.  **Positive Impact** |
| Warren 2015 | Kenya | Observational (Survey) | Maternal and neonatal healthcare services | Target: pregnant women and neonates  PROGRESS plus: socio-economic status, age, and place of residence | Quality of care | Overall, the quality of postnatal care in all facilities was low, which indicates that the postnatal period continues to receive limited attention from both women and providers, even where a Safe Motherhood voucher exist.  **No impact on quality of care** |
| Meuwissen 2006  Does a competitive voucher program for adolescents improve the quality of reproductive health care? A simulated patient study in Nicaragua | Managua, Nicaragua | Quasi-experimental | Sexual and reproductive healthcare services, | Target: adolescent females between 12-20  PROGRESS plus: age, gender and socio-economic status | Quality of care | Voucher use by teenage girls was associated with a better perceived SRH care: User satisfaction was significantly higher in users-with-voucher compared with users-without-voucher.  **Positive impact** |
| Watt C 2015 | Kenya | Quasi-experimental | Reproductive health | Target: pregnant women  PROGRESS plus: Socio-economic status | Quality of care | Quality improvements were observed in PNC domain: maternal care, newborn care, and interpersonal skills.  **Positive impact** |
| Azmat 2013  *Social franchising and vouchers to promote long-term methods of family planning in rural Pakistan: a qualitative stocktaking with stakeholders* | Pakistan | Observational  qualitative | family planning | women  place of residence | Quality of care | Most Suraj providers said that IUCD insertion and  infection-prevention training enhanced their ability to  provide IUCD services and increased their standing in local communities. Clients showed positive attitudes towards modern contraceptive methods and identified providers and signboards as sources of information  **Positive impact** |
| Corby, 2011  Marie Stopes International | Madagascar | Technical report | Family planning health services | Target: women  PROGRESS plus: Socioeconomic status and place of residence | Healthcare Utilization  Healthcare expenditures | In some settings, SMS  Money transfer systems can significantly strengthen the reach, efficiency and sustainability of health services.  **Positive impact on reach, efficiency and sustainability of health services**  SMS money transfer systems can  Successfully reimburse health service providers in remote, rural and urban settings.  **Positive impact on reimbursement of services providers** |
| Meuwissen 2006  Impact of accessible sexual and reproductive health care on poor and underserved adolescents in Managua, Nicaragua: a quasiexperimental intervention study | Nicaragua | Quasi-experimental | Sexual and reproductive health care | Target : Adolescents girls  PROGRESS plus: socio economic status, place of residence and age | Healthcare utilization  Other outcomes  *health knowledge* | The voucher program succeeded in increasing access to SRHC for poor and underserved girls.  **Positive impact on health care utilization**  Voucher receivers answered significantly more questions correctly that were related to knowledge of contraceptives and sexually transmitted infections than no receivers.  **Positive impact on sexual and reproductive health knowledge** |
| Nguyen 2012 | Bangladesh | Observational (Survey) | Maternal health services | Target: Pregnant women and neonates  PROGRESS plus: Socioeconomic status | Healthcare utilization  Healthcare expenditure | We found that the program significantly increased the use of antenatal, delivery, and postnatal care with qualified provider. No significant effect of vouchers was found on the rate of Cesarean section.  Another encouraging finding is the program’s differentially large impact among the poorest women in the population.  **Positive impact on health care utilization especially among the poorest women with no impact on the rate of c-section.**  The amount of OOP payment is  about 34% lower in the intervention than in the comparison subdistrict.  **Positive impact on decreasing the out of pocket expenditure** |
| Dennis 2018 | Kenya | Quasi-experimental studies | Maternal health services | Target: women  PROGRESS plus: socioeconomic status and place of residence | Healthcare utilization | The voucher programme was associated with a 5.5% greater absolute increase in use  of facility delivery and substantial increases in use of  the private sector for all services.  **Positive impact on maternal health services utilization** |
| Meuwissen, 2006  Uncovering and responding to needs for sexual and reproductive health care among poor urban female adolescents in Nicaragua | Nicaragua | Observational (Survey) | Sexual and reproductive health services | Target: adolescents between the ages of 12-20 years old  PROGRESS plus: age and place of residence | Healthcare Utilization | Contraceptive use doubled among the sexually active non-pregnant voucher redeemers.  **Positive impact on contraceptive use among non-pregnant sexually active adolescents**. |
| Marchant, 2010 | Tanzania | Observational (Survey ) | Malaria prevention | **Target**: pregnant women  PROGRESS plus: place of residence and socioeconomic status | Healthcare utilization | This attrition was particularly marked  for the poorest women, among whom coverage was only  18%, compared with 37% for the richest.  **No impact on healthcare utilization by the poorest women**. |
| Brody 2013  The impact of vouchers on the use and quality of health care in developing countries: A systematic review | Developing countries | Systematic review | Reproductive and sexual health services, maternity services, and malaria treatment | Target: women  PROGRESS plus: Socio-economic status | Healthcare Utilization  Morbidity | The results in the areas of targeting, utilization, and quality of health goods and  services indicate that voucher programs are having a positive impact on health  service delivery.  **Positive impact on health service delivery**  The subsequent link that voucher programs improve the health of the population was found to be unstable in the data analyzed in this review.  **The impact of vouchers on the overall health of the targeted population was found to be inconclusive** |
| Agha 2011 | Pakistan | Quasi-experimental (pre-test/post-test non-experimental study) | Maternal health services- institutional delivery services | Target: pregnant women  PROGRESS plus: socio-economic status | Healthcare utilization | The findings of the assessment show that participation in the voucher scheme was associated with a 21.6  percentage point increase in institutional delivery in D.  G. Khan City, after adjusting for demographic and  socio-economic factors. The voucher scheme’s effect on PNC was particularly dramatic: participation in the voucher scheme was  associated with a 35-percentage point increase in PNC.  **Positive Impact on institutional deliveries and PNC services utilization** |
| Ahmed 2011 | Bangladesh | Observational (Survey) | Maternal health services | Target: Women  PROGRESS plus: socio-economic status and place of residence | Healthcare Utilization | Voucher recipients in the project  area were 3.6 times more likely to be assisted by skilled health personnel during delivery, 2.5 times more  likely to deliver the baby in a health facility, 2.8 times more likely to receive postnatal care (PNC),  2.0 times more likely to get antenatal care (ANC) services and 1.5 times more likely to seek treatment for  obstetric complications than pregnant women not in the program. Poor voucher recipients were 4.3 times more likely to deliver in a health facility and two times more  likely to use skilled health personnel at delivery than the non-poor recipients  **Positive impact on the maternal healthcare services and on improving health care access for poor voucher recipients.** |
| Eva 2015  Are Our Voucher Programmes Working? Evaluating Our Methods and Results in Six Countries | Countries in Africa and Asia | Technical report | Sexual and reproductive health services | Target: young women  PROGRESS plus: socio-economic status, age, and place of residence | Healthcare utilization | MSI voucher programs were successful at increasing uptake of family planning and  other SRH services. Increased uptake was found among all of the countries where these programs were implemented and for all of the services covered by the vouchers.**Positive impact on sexual and reproductive health services utilization.** |
| Chandir 2010 | Pakistan | Observational (Prospective cohort) | Immunization | Target: Infants  PROGRESS plus: Age | Healthcare Utilization | The DTP up-to-date immunization coverage at 18 weeks of age  increased two-fold (RR 2.20, 95% CI: 1.95–2.48, p < 0.001) in the incentive cohort compared to the no incentive cohort.  **Positive impact** |
| Grainger 2014 | Nicaragua ,Africa , Kenya, Uganda , Sierra Leone, Madagascar Asia, Armenia , India, Pakistan, Bangladesh, Cambodia , China , Indonesia, Korea, Myanmar, Taiwan, Vietnam | Systematic review | Sexual and reproductive health services, maternal services and child health services | Target: Children , women and adolescents  PROGRESS plus: Socio-economic status | Healthcare utilization | In all programs reviewed a positive response is observed,  with providers responding better to the clients and investing voucher revenue to make their facility more attractive,  and consumers changing their health seeking behavior to  access services which they were not previously using.  **Positive impact** |
| Njuki 2015 | Kenya | Observational (Qualitative study) | Sexual and reproductive health services | Target: women and pregnant women  PROGRESS plus: socioeconomic status | Healthcare utilization  Mortality | The majority of the facility managers and providers felt  that the voucher program increased access to services  and reduced inequities by making RH services affordable to the poor.  **Positive impact**  Majority of facility managers and healthcare providers  felt that the program was associated with a reduction of  maternal and newborn mortality.  **Positive impact** |
| Mia 2021 | Bangladesh | Observational (Survey) | Maternal healthcare services | Target: pregnant women  PROGRESS plus: Socio-economic status | Healthcare utilization | Women from the lowest socioeconomic group who were voucher recipients received substantially more components of antenatal care (mean score: 159.6±82.1) compared with non-recipients (mean score: 115.7±83.0)s. Voucher membership was associated with more complete ANC,  with a mean completeness score of 185.2±101.0 for voucher recipients and 139.6±93.3 for nonrecipients **Positive impact** |
| Obare 2014 | Kenya | Quasi-experimental design | Sexual and reproductive health care services and maternal health care services | Target: women and pregnant women  PROGRESS plus: socio-economic status | Healthcare utilization | These findings suggest that the voucher program contributed to improved access to institutional delivery by shifting births from home to health facilities.  **Positive impact** |
| Obare 2015 | Kenya | Observational (Survey) | Sexual and reproductive health care services and maternal health care services | Target: women and pregnant women  PROGRESS plus: socio-economic status | Healthcare utilization  Healthcare expenditure | The proportion of  women in voucher sites that had used the safe motherhood voucher increased from 15 % in the 2010–2011 survey to 44 % in the 2012 survey. Similarly, the proportion  that had ever used the family planning voucher increased  from 2 % in 2010–2011 to 7 % in 2012.  **Positive impact**  The reproductive health vouchers program in Kenya significantly contributed to reductions in the  proportions of women in the community that paid out-of-pocket for safe motherhood services at health facilities.  **Positive impact** |
| Ali 2019 | Pakistan | Observational (Survey) | Reproductive healthcare services and family planning | Target: women  PROGRESS plus: socio-economic status | Healthcare utilization  Other outcomes  *health knowledge* | Vouchers are a good financing tool to improve equity, increase access, and quality of services for the underserved thus contributing towards achieving universal health coverage targets.  **Positive impact**  Awareness of contraceptives increased by 30 percentage points among population in the intervention area.  **Positive Impact** |
| Bajracharya 2016 | Cambodia | Observational (Prospective cohort) | Reproductive health services | Target: married, non-pregnant women age 18-45  PROGRESS plus: socio-economic status, age and gender | Healthcare utilization | The largest gains in LARC uptake occurred among women from the lowest socioeconomic strata. This suggests that vouchers may be an effective strategy for giving access to LARCs to women who might be unable to obtain the methods they want without the help of a voucher.  **Positive Impact** |
| VandePoel 2014 | Cambodia | Observational (Survey) | Maternal healthcare services | Target: pregnant women  PROGRESS plus: socio-economic status | Healthcare utilization | Overall, the use of maternal health care increased substantially between 2001 and 2009.  **Positive impact** |
| Bellows 2016 | Low middle- income countries | Systematic review | Reproductive health services – family planning | Target: women  PROGRESS plus: socio-economic status | Healthcare utilization | It is not surprising that nearly all of the studies reported on changes in use and that most studies found a significant increase in contraceptive use.  **Positive impact** |
| Keya 2018 | Bangladesh | Observational (Survey) | Maternal health care services and neonates’ health services | Target: pregnant women and neonates  PROGRESS plus: socio-economic status | Healthcare utilization | There was a statistically significant increase in the use of  public facilities (DID 13.9 percentage points, p ¼ .004), a  sharp decline in the use of for-profit private facilities (  DID 17.9 percentage points, p < .001), and significant  increase in deliveries at nongovernment organization (NGO)  facilities. By end line,  one third of the births in voucher areas were attended by  MTPs with no significant difference between voucher and  control areas. Inequity reduced after the program implementation, particularly in the voucher areas  **Positive impact on utilization of public facilities and reducing inequity in delivery service utilization but no impact on the at having medically trained practitioner during delivery** |
| Meuwissen 2006  Perceived quality of reproductive care for girls in a competitive voucher programme. A quasi-experimental intervention study, Managua, Nicaragua | Nicaragua | Descriptive (Case study) | Sexual and reproductive health services | Target: adolescent girls  PROGRESS plus: socio-economic status and age | Healthcare utilization  Other outcomes  *Health knowledge* | During the voucher program, 94% of the girls left the clinic with a contraceptive method (B1, p = 0.01). This  improved practice continued after the program ended (p= 0.10), but the frequency of follow-up appointments decreased.  **Positive impact on utilization but no impact on follow-up**  Although the percentage of doctors discussing STI/HIV risks during a consultation increased, a considerable number of patients did not receive the desired information.  **No impact on improving knowledge of adolescent girls on STIs** |
| Boddam-Whetham 2016  Vouchers in Fragile States: Reducing Barriers to long-acting Reversible contraception in Yemen and Pakistan | Yemen & Pakistan | Descriptive  (Case study) | Reproductive health services | Target: women  PROGRESS plus: socio-economic status | Healthcare utilization | We found a much higher-than-expected uptake of LARC and PM services in voucher areas in the year 2014: 720 vs. 521 expected overall. This 38.0% difference is a strong indication of the positive effect of the family planning vouchers.  **Positive impact** |
| Brody 2013  Redeeming qualities: Exploring factors that affect women's use of reproductive health vouchers in Cambodia | Cambodia | Observational (Qualitative study) | Reproductive health services | Target: women  PROGRESS plus: socio-economic status | Healthcare utilization  Implementation considerations  facilitator | women expressed positive feelings towards the voucher program, reporting that they sought earlier and more frequent care with the vouchers  **Positive impact**  several areas for program improvement were identified including the importance of addressing preexisting demand-side barriers to using reproductive health services, the need for more comprehensive counselling during voucher distribution. Early information from program beneficiaries can lead to timely and responsive changes that can help to maximize program success. This study highlights the importance of tailoring voucher programs to specific community needs, a strategy that can lead to better program uptake |
| Obare 2013 | Kenya | Observational (Survey) | Reproductive health services | Target: women, neonates  PROGRESS plus: Socio-economic status | Healthcare utilization | The reproductive health vouchers programme in Kenya is associated with increased utilization of health facility delivery, skilled delivery care and postnatal care services by poor women from communities exposed to it.  **Positive impact** |
| Mahmood 2019 | Bangladesh | Observational (Survey) | Maternal health services | Target: pregnant women and neonates  PROGRESS plus: Socioeconomic status | Healthcare utilization | The predicted probabilities of cluster membership for voucher recipients showed that nearly half (43.4%) of voucher recipients were predicted to be members of cluster 1 (high utilisation). Cluster 3 (sufficient ANC, else low) also showed a higher proportion of voucher recipients compared to nonrecipients by 5.5% points. On the contrary, the predicted probability of voucher recipients belonging to cluster 4 (overall low utilisation) was significantly lower (9.8%) compared to voucher non-recipients (41.9%) (Figure 2). The predicted probabilities of belonging to cluster 2 (high utilisation except ANC) are also lower among voucher recipients (5%) compared to voucher non-recipients (6.5%).  **Positive impact on ANC utilization** |
| MacedodeOliveira 2010 | Mozambique | Observational (Cross sectional study) | Malaria treatment | Target:  Children under 5 years  PROGRESS plus: place of residence, | Healthcare utilization | By providing free LLINs to children under five years of age, the campaign was intended to increase HH ownership and usage of ITNs in these two provinces. These results show increased HH ownership and usage by children under five years of age, 50.2% and 60.3%.  **Positive impact** |
| Rahman 2012 | Bangladesh | Observational (Cross sectional study) | Maternal health services | Target: pregnant women  PROGRESS plus: place of residence | Healthcare utilization | Context regarded perceived long  distances to health facilities and high transport costs.  Regarding community support for the interventions, the  schemes were perceived to be acceptable and helpful  particularly to the most vulnerable.  **Positive Impact on health care access** |
| Kihara 2015 | Kenya | Observational (cross-sectional) | Maternal health services including antenatal, intrapartum and postpartum care and family planning | Target: women  PROGRESS plus: Socioeconomic status and place of residence | Health care utilization | The Voucher system increased the number of facility-based deliveries, antenatal care services for women.  **Positive impact** |
| Kramer 2017 | Tanzania | Descriptive  case study | Insecticide- treated nets | **Target**: pregnant women and infant  **PROGRESS plus**: place of residence | Mortality | The ITN voucher schemes contributed to the reduction of the impact of malaria related mortality rates, where a 55% decline was seen in under-5 children.  **Positive impact** |
| Atukunda 2019 | Uganda | Experimental (RCTs) | Family planning | Target: Women living with HIV  PROGRESS plus: place of residence | Healthcare utilization | The family planning vouchers increased the contraceptive uptake by women  **Positive impact** |
| Amendah 2013 | Kenya | Observational  cohort | Reproductive health and facility-based delivery | Target: women  PORGRESS plus: place of residence | Healthcare utilization | The voucher program improved facility-based delivery especially for poor women  **Positive impact** |
| Bellows 2013  A taxonomy and results from a comprehensive review of 28 maternal health voucher programmes | Asia (Armenia, Bangladesh, Cambodia, China, India, Indonesia, and Pakistan) Africa (Kenya, Sierra Leone, Uganda) | Systematic review | Maternal health voucher | Target: pregnant women  PROGRESS plus: socio-economic | Healthcare utilization | Voucher program can increase health care services utilization  **Positive impact** |
| Rob 2009 | Bangladesh | Quasi-experimental (Pre and post design) | Antenatal care (ANC) delivery and postnatal check-up (PNC) | Target: pregnant women  PROGRESS plus: socio-economic status and place of residence | Health care utilization | The vouchers increased institutional deliveries from 2 till 18 % and the utilization of ANC and PNC increased as well.  **Positive impact** |
| Janisch 2010 | Kenya | Observational (Survey) | Maternal health services, family planning and Gender violence recovery services | Target: women and pregnant women  PROGRESS plus: age and socioeconomic status | Health care utilization | The voucher scheme increased facility-based deliveries, family planning services mainly due to the large target group and easy accessibility to facilities  **Positive impact** |
| Ali 2020 | Pakistan | Observational (Prospective cohort) | Family planning, child immunization, | Target: women and children  PROGRESS plus: socio-economic status and place of residence | Healthcare utilization | The integrated approach of the voucher scheme led to an increase in immunization coverage and contraceptive use  **Positive impact**  Uptake of voucher scheme was high especially among poor women  **Positive impact** |
| Ir 2010 | Cambodia | Observational (Survey) | Maternal health services, hospitalization services, transportation, food allowance, and funeral cost | Target: pregnant women  PROGRESS plus: socio-economic status and place of residence | Healthcare utilization  Healthcare expenditure | The number of facility-based delvieries increased sharply after the introduction of the voucher schemes especially for poor women  **Positive impact**  Vouchers have a strong potential for reducing financial barriers to access health care  **Positive impact** |
| Bellows 2017 | Uganda | Observational (Survey) | Family planning services | Target : women  PROGRESS plus: socio-economic status | Healthcare expenditure | We estimated that the services provided in 2014 save nearly US$14 million in direct health care costs.  **Positive Impact** |
| Ensor 2017 | Cambodia | Observational (Retrospective study) | Reproductive health services and maternal healthcare services | Target: women, and pregnant women  PORGRESS plus: Socio-economic status | Healthcare expenditure | Vouchers implemented on their own appear to have a small, not significant effect on spending; a finding that is unsurprising since they are focused only on reproductive health services. However in areas where vouchers are combined with health equity funds (government or donor) the negative effect on spending is more substantial.  **No impact on health spending if implemented alone, but a positive impact when coupled with health equity funds** |
| Bellows 2013  Increase in facility-based deliveries associated with a maternal health voucher programme in informal settlements in Nairobi, Kenya | Kenya | Quasi-experimental (Interrupted time series) | ANC, PNC and Facility-based delivery | Target: women  PROGRESS plus: socio-economic status and place of residence | Healthcare utilization | Increase in deliveries with skilled birth attendants but not on facility-based deliveries  **Positive impact** |
| Kanya 2014 | Uganda | Observational (Retrospective study) | Maternal health services | Target: women  PROGRESS plus: Socio-economic status | Healthcare utilization | The voucher scheme increased the percentage of facility-based deliveries especially for the poor women  **Positive impact** |
| DeBrouwere 2009 | Cambodia  Rwanda  Uganda | Descriptive (Case study) | Maternal services | Target group: women and newborns  Progress plus:  Place of residence | Healthcare utilization    Implementation Consideration | Healthcare utilization  Vouchers (Cambodia): Institutional deliveries increased by 25% after the implementation of the voucher scheme, performance based contracting and health equity fund *(positive effect)*  Equally, investing in a supply-side intervention without community sensitization would not be very efficient and would become rapidly counter-productive (without patients, health personnel would lose their competence).  -The choice of an intervention depends on the context and whatever the intensity of the work performed; it never solves the problem on its own.  -Choices cause change in the health system, and nobody can predict how and to what extent a ‘good’ decision to improve access to care has a negative or a positive effect on other segments of  The health system. |
| Chersich 2016 | Low- and middle-income countries (not specified)  Rwanda  Indonesia | Systematic Review | Cash or voucher: Services covered depended on the scheme but overall the majority covered ANC, post-partum care, and maternity services. Those not focused on women would provide services to reduce poverty and/or food security  are covered in the minimum package | Target group:  Cash or voucher: Pregnant women  Progress plus:  Socio-economic status | Implementation considerations  barriers | Finally, some reports of corruption were noted. This involved, for example, health workers taking money intended for pregnant women, and giving vouchers to ineligible women in programmes that paid commissions to staff for each voucher distributed (Kenya, row 6). In many instances, programmes that used complex procedures for determining eligibility struggled to identify individuals requiring support, even ending up with the lowest uptake among the poorest women (India, row 4; Nepal, row 8). |
| Bakyono 2020 | Burkina Faso | Observational (Qualitative) | reproductive health (family planning) | rural women  place of residence | Implementation considerations  facilitator | The removal of the financial barrier is not sufficient to remove the other major barriers the use of family planning (FP) services by women in  union. For example, contextual factors and  interventions remain decisive in the use of modern contraceptives by  married women residing in rural areas in a situation of free FP services. So,  for a significant and lasting increase in contraceptive prevalence in Burkina Faso, the national strategy for free FP should be supported by proven strategies involvement of men in FP in addition empowerment and instructional strategies  of women and young girls in order to reduce their consistently high fertility preferences |
| Marchant 2011 | Tanzania | Observational (Survey) | antenatal care | Pregnant women  Progress plus:  place of residence | Healthcare utilization | Coverage of the ITN voucher was high (84% in  2007), but only 61% of optimal voucher protection was achieved. It was reduced by a combination of late attendance at clinic and staff not distributing vouchers at first visit. An increasing trend by gestational age in ITN  use was observed each survey year, rising in 2007 from 23% of first-trimester women to 30% of women postpartum **(positive effect)** |
| Njuki 2012 | Kenya | Observational (qualitative study) |  | Women  Progress plus:  place of residence | Implementation consideration  barriers | The findings showed promising prospects for the uptake of OBA GBVR services among target population. However, a number of factors affect the uptake of the services. These include lack of general awareness of the GBVR services vouchers, lack of understanding of the benefit package, immediate financial needs of survivors, as well as stigma and cultural beliefs that undermine reporting of cases or seeking essential medical services. Moreover, accreditation of only hospitals to offer GBVR services undermines access to the services in rural areas. Poor responsiveness from law enforcement agencies and fear of reprisal from perpetrators also undermine treatment options and access to medical services. Low provider knowledge on GBVR services and lack of supplies also affect effective provision and management of GBVR services. |
| Massavon 2019 | Uganda | Observational Qualitative study | antenatal  care (ANC) | Women  Progress plus:  place of residence | Healthcare utilization | Transport vouchers  were preferred over baby kits, although both interventions  were perceived to be necessary. Health-seeking  behaviors entailed perceived increased utilization of  maternal health services  ***Positive impact***  ***institutionnal deliveries*** |
| Francis Obare 2016 | Uganda | Quasi-experimental | Maternal health services | Target: women  PROGRESS plus: socio-economic status | Healthcare utilization | The voucher program increased access to facility-based deliveries and decreased home based deliveries. However, no change was seen in the use of PNC, or ANC  **Positive impact on facility-based delivery but no impact on ANC and PNC** |
| Azmat 2013  *Impact of social franchising on contraceptive use when complemented by vouchers: a quasi-experimental study in rural Pakistan* | Pakistan | Quasi-experimental | Family planning services | women  socio-economic status and place of residence | Healthcare utilization | use of modern contraceptive increased by 28.5%, and the overall contraceptive prevalence rate increased by 19.6%.  A significant change (11.1%) was recorded in the uptake of IUCDs, which were being promoted with vouchers. **Positive impact** |
| Malik, 2021 | Pakistan | Observational cross sectional | ANC visits, institutional births (IB) including normal vaginal and caesarean section delivery, postnatal care (PNC) visits, key vaccinations, family planning service | Pregnant Women and Mothers  place of Residence & socio-economic status | Healthcare utilization | We find that in these districts the proportion of births with some skilled assistance—at home as well as in a facility—did increase significantly while (surprisingly and significantly) it fell in the remaining districts of NPPI which did not use vouchers or contracting  **Positive impact** |
| Lee, 2022 | Several low-middle income countries | Systematic review (Interpretative Review) | Maternal health services | women  socio-economic status, gender | Implementation consideration  barrier | Despite its potential, mobile e-voucher programs may face high startup cost, such as investment in hardware, development of software systems, or additional training for the staff [23]. However, the long-term return on investment can be higher than the traditional paper voucher programs, as the costs per client will decline by saving on the administrative costs of printing and distribution, fraud control, monitoring, and claims processing and payment. For example, a previous study on a traditional maternal voucher program in Uganda suggested the use of mobile phones for making payments to reduce transaction costs  **The benefits of transitioning to mobile e-vouchers identified from the logic model can be summarized as scalability, transparency, and flexibility.** |
| Azmat 2021 | Pakistan | Literature review | family planning | Women  place of residence | Healthcare utilization | Social franchising used alongside free vouchers for long-term contraceptive choices significantly increased the awareness of modern contraception, ever use of modern contraceptive and the overall contraceptive prevalence rate. A significant change of 11.1% was recorded in the uptake of IUDs, which was being promoted with vouchers. The results show that the vouchers were effective in  increasing the use of any modern method between  baseline and end line by 8.2%  **Positive impact** |
| Nandi 2022 | Bangladesh | quasi-experimental | maternal health services | Women  Socioeconomic status, Place of residence | Healthcare utilization  Mortality | increases in the use of maternal health services, particularly on the probability of delivering in a health facility, that materialized 2 or more years after program implementation  **Positive impact**  improvements in stillbirths, neonatal, and infant mortality were not demonstrated  **no impact** |
| Mushi 2003 | Tanzania | Observational (Qualitative study) | Malaria treatment | Target : Pregnant women and young children  PROGRESS plus: Gender and age | Implementation consideration | Discount vouchers are a feasible system for targeted subsidies, although a substantial amount of time may be needed to achieve high awareness and uptake. Pregnant women are likely to be an ideal group for such targeting. Within a poor society, vouchers may not necessarily increase health equity, at least at first, since some cash is needed when using a voucher as part-payment. |
| Okal 2013 | Uganda | Observational (Survey) | Maternal health care services | Target: pregnant women  PROGRESS plus: socio-economic status and place of residence | Implementation considerations  facilitator | intensifying ongoing health education, continuous monitoring and evaluation, and integrating the voucher program with other services is likely to address some of the barriers. The public sector facilities were also seen as being well positioned to provide voucher services because of their countrywide reach, enhanced infrastructure, and referral networks. The voucher program also has the potential to address public sector constraints such as understaffing and supply shortages |
| Hoddinott 2020 | Bangladesh | Observational (Cross sectional study) | Child health nutrition | Target: children between 6-23 months of age  (refugees)  PROGRESS plus: socio-economic status  Refugee status | Child development | Household receipt of an e-voucher was associated with improved linear growth in children. This association is robust to the inclusion of maternal, household and location characteris- tics. The magnitude of the association is 0.38 SD (CI: 0.01, 0.74), and statistically significant at the five percent level.  Positive impact |
| Azmat, 2023 | Pakistan | Observational  Cross-sectional survey | Family planning | Women  Socio-economic status | Healthcare utilization  (Contraceptive use) | The prevalence for contraception in two-independent study sites, following closure of voucher intervention remained high than national average. This study provides evidence that family planning vouchers can bring about an enduring positive change in clients’ behaviors in using modern contraceptive methods among poor populations  **Positive effect** |
| Sultana, 2023 | Bangladesh | Observational  Cross-sectional |  | Women and child  Socio-economic status | Healthcare utilization  (Immunization coverage) | immunization coverage amongst the children of MHVS beneficiaries was higher than the coverage for children whose mothers were not registered in the voucher program. MHVS ensures utilization of safe motherhood practices, through ANCs during pregnancy, institutional delivery/delivery by SBAs and PNCs after delivery and through these services, mothers acquire knowledge regarding childhood immunization leading to increased child immunization coverage.  **Positive effect on child immunization through increasing safe motherhood practices** |
