# Supplementary material for: Financing for equity for women’s, children’s and adolescents’ health in low- and middle-income countries: A scoping review
Source: PLOS Glob Public Health. 2024 Sep 12;4(9):e0003573. doi: 10.1371/journal.pgph.0003573 (PMC11392393; doi:10.1371/journal.pgph.0003573)
Supplement: S5 Table — (DOCX) [file pgph.0003573.s008.docx]

**S5 Table of characteristics: Several interventions (n=28)**

| **Author Year** | **Country** | **Study design** | **Interventions**  **(separately)** | **Target group and PROGRESS Plus measures** | **Outcome(s)** | **Main Results**  **(yes/no/inconclusive)** |
| --- | --- | --- | --- | --- | --- | --- |
| Robertson 2013 | Zimbabwe | Experimental  RCT | CCT, UCT | children under 18  socioeconomic status (SES) | Healthcare utilization | The proportions of children aged 0–4 years with complete vaccination records was 3·1% greater in the UCT group and 1·8 greater in the CCT group than in the control group  **Positive impact on vaccination (UCT higher than in CCT)** |
| Crea 2015 | Zimbabwe | Experimental  RCT | CCT, UCT | children between 5-18  place of residence and socioeconomic status | Other outcomes  *Health vulnerability*  *Health outcomes* | however, cash transfers (CCT or UCT) seem to have no significant effect on health vulnerability. Given the relatively short amount of time between baseline and follow-up, it may be that not enough time had elapsed to detect the potential impact of cash transfers or to allow for changes in long-term health outcomes.  **No impact/inconclusive** |
| Walque 2017 | LMIC | systematic review | CCT, UCT | children, pregnant women and adolescents  socioeconomic status | Child development  *Height-for-age*  *Weight-for-height*  *Weight-for-age*  Mortality  Healthcare utilization  *Women*  *Child* | The evidence linking **CCTs** to improvements in child height was mixed, both across and within countries. In Mexico alone, four studies found a significant effect of the program on height, three found significant improvements only for specific subpopulations such as children ages zero to six months or children of mothers with no education, and one study found no significant effects in urban areas. Evaluations of other CCT programs were also inconclusive: in Bangladesh, Indonesia, Peru, and Tanzania, there were no significant effects on children’s height  The evidence for **UCTs** was also inconclusive. There was a significant improvement in children’s HAZ in Sri Lanka, but no effect on young children’s HAZ in Ecuador and Zambia  **CCT/UCT inconclusive**  The evidence of the impact of **CTs** on WHZ or wasting in general reveals little to no impact. Studies of CTs in Bangladesh, Nicaragua), and Tanzania) found no impact on wasting or WHZ.  **No impact CCT/UCT**  The effects of **CCT and UCT** programs on WAZ or the prevalence of underweight are mixed, although the majority of studies found no significant effects or only found effects in subgroups. **CCT** studies in Bangladesh (Ahmed and others 2009), Nicaragua, Peru, and Tanzania found no impact on WAZ or underweight; a different study in Nicaragua found a significant reduction in the prevalence of underweight. Findings regarding the impacts of **UCTs** on weight are also inconclusive; a study in found no impact on weight or the prevalence of underweight, and a study in Zambia found an increase in WAZ but no impact on the prevalence of underweight. In Burkina Faso, neither CCTs nor UCTs had an impact on  **Inconclusive CCT/UCT**  Four of the six studies found significant decreases in mortality rates in Brazil, India, and Mexico. More than half of the decline in infant mortality in Mexico resulted from reductions in respiratory and intestinal infections and nutritional deficiencies. However, studies in Indonesia (World Bank 2011) and Nepal (Powell-Jackson and others 2009) found no significant impact on neonatal or infant mortality  **Positive impact/ no impact**  A systematic review showed that **CCTs** increased antenatal care, skilled attendance at birth, and births at clinics. However, the results were mixed, generally depending on the focus of the program  **Positive impact**  Although a large majority of the studies reviewed found significantly positive effects of CTs on the probability that a child had received growth monitoring or health checkups, the impact depended on whether the transfer was conditional Whereas the studies on **conditional programs** revealed a significant increase in the percentage of children being taken to health facilities for growth monitoring or preventive care, the studies on **UCT**s did not find a significant increase  **CCT better effect than UCT**  Few studies have explicitly compared CCTs and UCTs in the same context. One experiment examined the impact of CCTs and UCTs on adolescent girls’ schooling and health outcomes in Malawi, concluding that CCTs outperformed UCTs for schooling outcomes, but UCTs outperformed CCTs for several other outcomes—for example, delaying marriage and childbearing**.**  **UCT Vs CCT** |
| Grepin 2019 | kenya | Experimental  RCTs | CCT _+_ vouchers (combination)  All vouchers covered the costs of ANC visits, delivery, and PNC visits. We administered two types of cash transfers (CCT)  and (UCT) – designed to help overcome transport  cost barriers | pregnant women  place of residence and socio-economic status | Healthcare utilization  *Institutional*  *deliveries* | we find a strong effect of the full voucher and the CCT on facility deliveries, especially when they were delivered in combination. Forty-eight percent of **women with access to both the CCT and the full voucher** delivered in a clinic or hospital, while only 36 percent of those with neither. |
| Barrington 2022 | Ghana | Observational  qualitative study | UCT+ HI (combination)  Ghana’s Livelihood Empowerment Against Poverty (LEAP 1000) is a government-run unconditional cash-transfer  program paired with health insurance premium waivers, targeted at poor households with pregnant | households with orphans and vulnerable children, elderly with no productive capacity, and persons with severe disability  socio-economic, status, age and disability | Healthcare utilization | Participants overwhelmingly emphasized that having insurance increased their access to and use of healthcare by reducing cost barriers. Even though several noted that not all costs were covered, most participants felt that they were able to use healthcare services more for themselves and their children with the insurance |
| Ravit 2022 | Burkina Faso | Quasi-experimental  Interrupted Time Series analysis | User fee exemption +PBF (combination)  supply-side intervention  (PBF) combined with a demand-side intervention (the gratuité) led to an increase in the rate of CS in Burkina Faso | Women  socio-economic status | Healthcare utilization | despite high FBD rates in Burkina Faso, the PBF intervention and the gratuité, less than 3% of women who gave birth in a health facility had a CS. This is far from the minimum of 5% recommended by the WHO. |
| Nunez 2016 | Argentina | Observational  Correlational | Combination  Plan Nacer was a combination of public insurance strategy and a pay-for-performance scheme. Then it was scaled up to include new age groups under the new name programa SUMAR in addition to the CCT | children younger than 5 year  place of residence and age | Morbidity | Our findings describe a substantial decrease in the prevalence of stunting and underweight among children covered by UHC programs (insurance &CTs) in Argentina between 2005 and 2013.We observed decreasing trends in the prevalence of stunting and severe stunting in both rural and urban populations. However, rural populations maintained a higher prevalence of stunting and obesity, requiring the commitment to continue improving development indicators in rural areas.  **Positive impact/urban more than rural** |
| Dennis 2019 | Kenya | Observational  Survey | Vouchers, fee exemption | pregnant women  socioeconomic status | Healthcare utilization  Quality of care | our findings suggest that before the free maternity services policy was introduced, full implementation of the voucher programme improved use of continuous care among ANC users  **Positive impact (vouchers)**  After controlling for all other variables in the model, there did not appear to be a general effect of the free maternity services policy on use of early ANC among all births or on either of the measured continuum of care outcomes. (ANC, PNC, facility based delivery)  **No impact (free maternity services)**  vouchers allowed women to seek care in public and private facilities, while the user fee removal policy only applied to public facilities. By making private sector services more accessible, the voucher programme may have contributed to reducing women’s barriers to timely maternal health service initiation and improving continuity of care.  **Positive impact (vouchers)** |
| Wang, 2014  *Health insurance coverage and its impact on maternal health care utilization in low- and middle-income countries* | 8 countries sub-Saharan Africa, West Asia, South and Southeast Asia | Technical report | HI, CBHI | Target: women  PROGRESS Plus  Measure: socioeconomic status | Healthcare utilization  *ANC*  *facility-based delivery*  Implementation consideration | Our results indicate that, health insurance coverage has a positive impact on women’s access to at least one antenatal care visit in Indonesia, Rwanda and Cambodia, and in Ghana and Indonesia insurance coverage has a positive impact on making at least four antenatal visits, as recommended by WHO. The characteristics of health insurance schemes in these countries may partially explain the higher frequency of antenatal visits.  Health insurance status shows positive effects on initiating antenatal care in the first trimester in three of the eight countries (Namibia, Burundi, and Indonesia)  **Positive impact ANC**  When assessing the impact of health insurance on the use of facility-based delivery care, we found strong evidence of positive effects of health insurance in Cambodia, Ghana, Indonesia, and Rwanda, where delivery care is fully covered by health insurance  **Positive impact**  Rwanda’s **community-based health insurance** aims to increase use of health care, especially for the poor. Our analysis showed that, although the great majority of Rwandan women receive antenatal care, only about one-third meet the recommended standard of at least four antenatal visits. A possible reason might be that many women lack knowledge about the extent of health services covered by insurance. |
| Wang, 2017 | Ghana, Indonesia and Rwanda | Observational  secondary data analysis | HI, CBHI   - The main form of insurance in Ghana is the National Health Insurance Scheme (NHIS) - CBHI in Rwanda, known as Mutuelle de Sante´, was initiated in 2004 and has been heavily promoted and subsidized by the government - Compared with Ghana and Rwanda, which have one dominating insurance scheme, Indonesia offers more health insurance options | **Target:**  women  **PROGRESS Plus**  socioeconomic | Healthcare utilization  Other outcomes  *Insurance coverage* | insurance coverage has a positive impact on women’s access to at least one antenatal care visit **in Indonesia and Rwanda**. **In Ghana and Indonesia**, insurance coverage shows a positive impact on women making at least four antenatal visits, as recommended by the WHO  **Positive impact ANC**  In all 3 countries, health insurance coverage contributed to an increase in use of facility-based delivery care.  **Positive impact FBD**  In all three countries, women from wealthy households are more likely to participate in health insurance. Research in Ghana shows that the insurance premium system based on income is not as efficient as it was meant to be, potentially causing exclusion of a large number of poor people from the program because they cannot afford to pay the premiums  **No equity** |
| Witter, 2017 | India | Literature review | CCT, social protection scheme  The federal government also finances a nation-wide Social Protection Mechanism for households living ‘below the poverty line’. This is the RSBY, which is tax-funded and purchases health care from public as well as ‘empanelled’ private health care facilities  In addition, in India, there is a tax-funded Conditional Cash Transfer Scheme (CCT)—the Janani Suraksha Yojana (JSY)—which offers a cash incentive to women who deliver in a health facility | **Target group:** Women  **PROGRESS Plus Measure:** Gender | Healthcare utilization  Implementation considerations  *Barriers* | Studies indicate that the RSBY has increased access to care for low-income women  **Positive impact social protection scheme**  the CCT program has increased the proportion of women delivering in institutions significantly  **Positive impact CCT**  However, a more gender aware design could have removed some major barriers. only five members may be enrolled per household. Thus, the RSBY leaves the choice of who is to be covered to household dynamics. Barriers to the social protection scheme (RSBY) include household dynamics. For instance, elderly and girls were shown to more likely be excluded when more than five members are available in the households. Other barriers include inadequate information on service coverage and included health facility.  **Social protection scheme**  gender-based vulnerabilities were not factored into the design of the scheme. Across all states, the scheme excludes women who already have two live births. As fertility levels are considerably higher among women from the two lowest wealth quintiles and among women with lower educational levels, the exclusion of women with more than two live births from the JSY scheme disproportionately affects marginalized groups of women.  **CCT**  In states of India with a high proportion of institutional deliveries, only women from households below the poverty line, and those above 18 years of age, are eligible for the JSY. Even among those satisfying all eligibility criteria, women from the most marginalized groups tend to be excluded. For example, in a Tamil Nadu study, only 25% of women who satisfied the eligibility criteria benefitted from the conditional cash transfer scheme. The main reasons for exclusion were difficulties encountered in producing the necessary papers to prove eligibility because of lack of information, time and contacts  **CCT** |
| Brals, 2019 | Kenya | quasi-experimental  Controlled before and after | Community healthcare plan, user fee exemption | Target: pregnant women  PROGRESS plus: place of residence and occupation | Healthcare utilization  Quality of care | This study provides evidence that the FMS programme was positively associated with facility deliveries. The number of facility deliveries increased by 75% after the introduction of the FMS programme.  **Positive impact on FBD for the FMS program**  antenatal care utilization did significantly increase after the introduction of the TCHP programme. The TCHP programme ensured that quality maternal services became available closer to their homes, reducing travel costs.  **Positive impact TCHP**  Stakeholder interviews with hospital staff indicated that, even though the number of deliveries substantially increased after introduction of the FMS programme in public facilities, the number of staff members was not increased and supplies were running out and were not replenished, resulting in a decline in the quality of delivery care provided by these facilities  **Negative impact of FMS** |
| Borghi, 2006 | low income countries | Narrative/literature review | Introduction of User fees, user fee exemption, SHI, CBHI | **Target:**  Women and children  **PROGRESS Plus**  **Measure:** socioeconomic status | Healthcare utilization  Quality of care | Use of maternal health services is highly sensitive to the official fees charged. Several reports show that use fell after user fees were introduced. The situation is especially  severe for poor people. In Nepal, the poorest people are twice as likely as those who are least poor to reduce use of child health services in response to an increase in price. One of the constraints to use of maternal health care in the presence of fees is household inability to access cash at  the time of need, especially in rural areas where subsistence farming is characterized by temporal or seasonal inability to pay. This issue was reportedly a major constraint for  between 40% and 50% of households in west Africa  **Negative impact introduction of user fees**  **Fee removal** for maternal health services has been effective in increasing the mean number of booked deliveries by 4·6% in South Africa  **Positive impact fee removal**  **The schemes** have been successful in increasing assisted-delivery rates for scheme members by 45% in Rwanda and 12% in The Gambia  **Positive impact CBHI**  a social insurance scheme in Bolivia was designed specifically to provide for maternal and child health services. The scheme succeeded in increasing use of antenatal and delivery care by 50% in public health facilities, especially by the poorest people**.**  **Positive impact SHI**  The increased workload facing health professionals is another threat to the effectiveness of fee removal, with  implications for staff motivation and quality of care. A rise in maternal mortality in a tertiary hospital in South Africa was partly attributed to the additional patient load after fee  removal not being matched by an adequate increase in staff and facilities.  **Negative impact user fee removal** |
| Vora, 2015 | India | Observational cross-sectional | Voucher, CCT  In 2005–06, the nation launched the world’s largest conditional cash transfer scheme for maternal  health, Janani Suraksha Yojana (JSY)  Implemented  throughout Gujarat by 2007, the “voucher like” scheme provides tribal and BPL women with free delivery in an accredited private facility and reimbursement for transport | ever-married women, ages 15–49, in the lowest two  wealth quintiles  **Progress plus:**  socioeconomic status and place of residence | Healthcare utilization | Receipt of voucher like scheme or CCT significantly predicted institutional delivery among poor, rural women in Gujarat, with the odds of women delivering in a health facility almost four times higher among those participating in either scheme compared to non-participants.  **Positive impact FBD CCT and vouchers** |
| Prinja, 2015 | India | Observational Survey | User fee exemption, CCT  introduction of conditional cash transfers for institutional delivery—Janani Surkaha Yojana (JSY)—and implementation of a program for free cashless delivery in public institutions—Janani Shishu Suraksha Karaykaram (JSSK) | Women  place of residence | Healthcare expenditure  Healthcare utilization | introduction of focused strategies, such as the JSY ‘conditional cash transfer scheme’ and JSSK ‘cashless delivery’, have contributed positively toward reducing OOP expenditures and thus minimizing financial barriers  **positive impact CCT, user fee exemption**  Increased public spending and focused schemes, such as JSY and JSSK, under the umbrella of NRHM have led to increased utilization of public sector health facilities.  Positive impact **CCT, user fee exemption** |
| Hunter 2017  *The effects of cash transfers and vouchers on the use and quality of maternity care services: A systematic review* | low- and middle-income  countries  **20 countries in Latin America, sub-Saharan Africa and Asia** | systematic review | CCT, vouchers | women of all ages who were pregnant or within 42 days of giving birth.  Progress plus:  socio-economic status | Healthcare utilization | conditional cash transfers that included among their conditionalities the uptake of antenatal care services appeared to have had an impact on the proportion of women receiving multiple antenatal check-ups, but findings were less clear with respect to the uptake of other maternity care services in the continuum including childbirth and postnatal care (not included as conditionalities)  **inconsistent impact CCT**  vouchers for maternity care services increased the uptake of services for which they provided eligibility or subsidy, including antenatal care, skilled attendant at birth, facility births and, to a lesser extent, postnatal care  **Positive impact Vouchers** |
| Johnson 2016 | Ghana | Observational  Retrospective cohort | User fee exemption, HI  The free delivery care policy covered antenatal care, normal deliveries, management of assisted and surgical deliveries. In 2007, the free delivery care policy was formally ended due to lack of funding and integrated into the NHIS, which was already functional since 2005 | pregnant women  **PROGRESS Plus:**  Socioeconomic status | Healthcare utilization | There is clear evidence to suggest that while maternity fee exemption interventions have had an overall positive impact and reduced the extent of inequalities in the uptake of skilled birth care, the benefits to the poorest women were marginal and insignificant throughout the last two decades. the effect of the policies on skilled birth care is dependent on wealth. There is clearly a significant disadvantage for the poorest women accessing skilled birth care under various **maternal fee exemption policies**. Over the last two decades, the probability of skilled birth care has remained low increasing from only 30 to 38% for the poorest. Similarly, for the poor the probabilities increased from 34 to 52% during the free delivery care but decline to 42% when maternity payments were incorporated into the NHIS. Nonetheless, the increase was consistently higher for the richest from 56 to 93%. **This suggests that user fee exemption and user fee removal policies have benefited the richer groups rather than the poorer groups.**  **Negative impact FBD for the poorest especially NHIS** |
| Morgan 2013 | Low- and middle-income countries | Narrative/literature review | CCT, vouchers  the effects of financial incentives—performance-based incentives (PBIs), insurance, user fee exemption programmes, conditional cash transfers, and vouchers— | **Target group:** Women  **PROGRESS Plus Measure:** Socioeconomic Status (SES) | Healthcare utilization  Quality of care | the majority of studies that report **on skilled birth attendance or facility-based deliveries** show incentives to providers and consumers correlated with improvements in these indicators. Similarly, among studies that report on the effect of incentives on caesarean sections, the evidence shows incentives correlated with increased use of caesarean section  **Positive impact**  The evidence around **antenatal care (ANC) is** also mostly positive, with ANC visits increasing across programmes  **Positive impact**  Incentives for **postnatal care (PNC)** and family planning (FP) were less common across programmes, and, overall, the evidence is weak. Among the **insurance and voucher** studies that reported results for PNC, there was a consistently positive relationship between the incentives and the use of postnatal care. No effect on PNC was reported **in supply-side or user fee exemption programmes,** and the two studies that measured the effect of **CCT programmes** on PNC found negative but insignificant results  **Positive impact insurance/vouchers, no impact user fee exemption SSF**  **Voucher schemes** in Bangladesh and Pakistan show that vouchers increased service utilization more among the poor than the non-poor, and early results from an ongoing evaluation of five voucher schemes in Bangladesh, Cambodia, Kenya, Uganda, and Tanzania also show positive results on service utilization and equity  **Positive impact and equity**  Among studies that do report on quality, the evidence is mixed. Some studies report improvements in quality as measured by various contents of care indicators, which are, in some cases, directly incentivized and, in some cases, not  inconclusive |
| Das 2020 | India | Observational cross-sectional | User fee exemption, CCT  The Indian government provides for free/subsidized services in public hospitals and conditional  cash transfer (CCT) schemes for delivery car | **Target group:** Pregnant women  **PROGRESS Plus Measure:** Socioeconomic Status (SES) | Healthcare expenditure | Women incurred OOPE for delivery care at home and public hospitals despite free/subsidized care. All women had OOPE. It was mainly due to informal payment, food and cloth for baby transport and wage loss. Receiving **CCT** incentive did not influence  OOPE though it compensates OOPE incurred by women in lower eco-  nomic groups.  women who delivered at home incurred more OOPE  than public health facilities. This may be due to **free maternity programme** which compensated the direct expenditure and referral transport. |
| Richard 2010 | LICs ( Bolivia, Burkina Faso, Cambodia, Ghana, Guinea, India, etc.) | Descriptive Case study | User fee exemption, vouchers, cost sharing, CTs, SHI, CBHI | **Target:** pregnant women  **PROGRESS plus:** Socio-economic status | Healthcare utilization | All schemes reported increased uptake of services, although few had robust evidence of the extent of the increase. Only Guinea (CBHI) showed poor progress, with only a 5% increase in the assisted delivery rate from 2000 to 2006 (from 17% to 22%) in the areas covered by the program  **Positive impact** |
| Gopalan 2014 | low- and middle-income countries | systematic review | Demand Side Financing (vouchers, CTs, subsidies, HI) | Women  place of residence and socio-economic status | Morbidity | The experimental evaluation studies indicated that **DSF** was effective on improving nutritional status, child growth indicators,  The experimental evaluation studies indicated that **DSF** was effective on improving HIV status and adult morbidity status. However, their effects on maternal health, diarrhea and malaria status were unknown. |
| Hunter 2017  *Demand-side financing for maternal and newborn health: What do we know about factors that affect implementation of cash transfers and voucher programmes?* | low- and middle-income countries (22 countries) | systematic review | Demand Side Financing (CTs, Vouchers) | women of all ages who were pregnant or within 42 days of giving birth.  Progress plus:  socio-economic status | Implementation considerations  Quality of care | This analysis has highlighted a series of well-documented challenges for the implementation of **DSF schemes** in maternal and newborn health. These include issues of programme scope (in terms of programme eligibility, size and timing of payments and voucher entitlements), wider problems in healthcare systems (including inadequate infrastructure and human resources, lack of medicines and problems with corruption) and the population’s awareness and perceptions of programmes and health services.  Research highlighted in our analysis indicates that the implementation of **DSF programmes** may reinforce existing healthcare system problems including poor quality of care, demands for informal fees and the systematic exclusion of vulnerable groups. Vouchers for maternity care services are often proposed as a means to improve quality of care however experiences indicate private providers may find reimbursement rates to be unattractive and engage in practices such as providing differential quality of care or ‘skimming’ programme users who require minimal intervention  ***negative impact*** |
| Jehan 2012 | Nepal, India, Bangladesh, Pakistan | Literature Review | CTs, vouchers  **Nepal:** CT to increase FBD  **India:** CTs, voucher like scheme  **Bangladesh**: voucher +CT  **Pakistan:** voucher | **Target group:** Pregnant Women  **PROGRESS Plus Measure:** Socioeconomic Status (SES) | Healthcare utilization  Quality of care  Implementation considerations | All schemes except India’s Chiranjeevi Yojana voucher like scheme (for which there are not yet reported data) report increased utilization of maternity services in areas where they are operational.  Positive impact  Preliminary assessments across schemes have found that service quality, in general, remains poor. Intuitively, a rapid increase in utilization is likely to place a considerable burden on facilities and compromise quality in the short term, and there is no evidence to suggest that this has stimulated expansion of or improvements in services  The delayed release of funds will result in continued out-of-pocket payments by women, or the need for providers to reimburse eligible women from their own non-scheme funds. As a result, service users’ trust may be eroded, not just for maternity care, but for all health services, and providers may avoid the schemes or perceive them to be a financial liability.  Where schemes involve financial incentives, policy-makers, funders and administrators must be alert to the possibility of misuse of funds, and some mis-use has been reported  Above all, when designing schemes intended to benefit specific groups, the opinions and needs of those groups should be sought, but they rarely are. |
| Montagu 2009 | multiple developing countries | Narrative/literature review | Health financing in general/ novel approaches to financing  **SRH** | women and children  socio-economic status | Implementation considerations | The challenges for the delivery of SRH services in developing countries that are both sustainable and equitable are not easily overcome. While some funding initiatives have proven themselves capable of withstanding political changes, this is not always the case, and priorities unrelated to health or equity drive many international and national funding decisions. How SRH services are financed is central to assuring equity and access and must be the starting point for design and assessment of new and existing programs |
| Ahmed 2021 | Bangladesh | Observational  mixed method  (quantitative & qualitative) | Vouchers, HI  Health Voucher Scheme (HVS) in each of Dhaka North City Corporation, Dhaka South City Corporation and Chattogram City Corporation, and an MHI scheme in Dhaka South City Corporation area  HVS: voucher scheme  MHI: micro health insurance scheme | **Target group**: women, children  **PROGRESS plus**: socio-economic status | Healthcare utilization  Healthcare expenditure | higher healthcare utilization.  the poor and extreme poor had higher healthcare utilization from MTPs (medically Trained Providers) in HVS and MHI schemes compared to a community-based health insurance of informal workers  the increase in utilization of healthcare of the scheme enrollees contributed to equity in the society by narrowing the gaps in health benefits among socioeconomic groups  positive impact/ more equity  The schemes thus resulted in more financial risk protection and released some disposable income of the households for spending elsewhere on goods such as food, education and clothing, which may have increased their level of welfare as non-health benefits.  The reporting schemes made progress towards UHC by securing more health care at a lower level of OOP payments. t the schemes have the potential to secure financial protection and increase service coverage and consequently contribute towards achieving UHC. |
| Kabia, 2018 | Kenya | Observational (Qualitative study) | Pro-poor health policy reforms: free maternity, user fee removal in PHCCS, health insurance subsidy program for poor | **Target group:**  women with disabilities living in poverty  **Progress Plus:**  disability, socioeconomic status, place of residence | Implementation considerations | In Kenya, women with disabilities living in poverty experienced advantages and disadvantages when seeking care under pro-poor financing reforms that targeted them. This was as a result of interactions of personal factors such as gender, disability, and poverty, with environmental factors such as disability unfriendly transportation systems and health systems structures and negative healthcare worker attitudes. In an effort to achieve equitable health care for all, health systems need to address the unique barriers that people with disabilities face when accessing healthcare. |
| Aye, 2023 | Burkina-Faso | Quasi-experimental  pre- and post-test design | User fee exemption and PBF implemented in parallel | Children under five  Age | Healthcare expenditure | User fee exemption: a significant reduction in the proportion of children under 5 incurring any OOPE and a reduction in the magnitude of the expenditure among those reporting OOPE (facility-based out-of-pocket expenditures (OOPEs) for outpatient services) produced an 84% reduction in the probability of incurring OOPE and reduced total OOPE by 54%  **positive effect**  PBF: no substantial effect either on the probability of incurring any OOPE or on the magnitude of OOPE among individuals seeking care at PBF facilities.  **No effect** |
| Richterman, 2023 | LMIC | Observational  Secondary data analysis | CTs (CCT/UCT) | children under five years of age and women. | Mortality | Cash transfer programs were associated with significant reductions in mortality among children under five years of age and women.  **Positive effect on maternal and child mortality**  **similar effects for conditional and unconditional programs (UCT=CCT)** |
