# Supplementary material for: Financing for equity for women’s, children’s and adolescents’ health in low- and middle-income countries: A scoping review
Source: PLOS Glob Public Health. 2024 Sep 12;4(9):e0003573. doi: 10.1371/journal.pgph.0003573 (PMC11392393; doi:10.1371/journal.pgph.0003573)
Supplement: S6 Table — (DOCX) [file pgph.0003573.s009.docx]

**S6 Table of characteristics: cost sharing and subsidies (n=20)**

| **Author Year** | **Country** | **Study design** | **Health service covered** | **Target group and PROGRESS Plus**  **measures** | **Outcome(s)** | **Main Results**  **Is the intervention effective overall? (yes/no/inconclusive)** |
| --- | --- | --- | --- | --- | --- | --- |
| Burnham 2004 | Uganda | Observational (Secondary data analysis) | antenatal clinics, family planning, immunization | Target:  children  PROGRESS Plus  Measure: socioeconomic status | healthcare utilization | Use of all services increased — even those that had never before been subject to fees. The loss of some autonomy by the health facility and diminished community governance of health facilities may have long term negative effects.  ***Positive impact on healthcare utilization*** |
| Sidze 2013 | Kenya | Narrative/literature review | Family planning services ,health education and information; Maternal health services; Childbirth services; Child health services, including immunization; and All other obstetric and gynecological services | Target: Children, Women, and Pregnant women  PROGRESS Plus  Measure: place of residence | Healthcare expenditure | As a percentage of total out of pocket expenditure on general health, households spent 14% on reproductive health services in 2009–10. A reality check in fact showed that the contribution of user fees for reproductive health services in various health districts was disproportionally higher than the government’s own financial contribution  ***Negative impact on Health expenditures*** |
| Meda 2018 | Burkina Faso | quasi experimental design | deliveries and emergency obstetric and neonatal care | Target: pregnant, women and neonate  PROGRESS Plus  Measure: place of residence | Mortality  healthcare utilization | The study reported a non-significant decrease in deaths per live births  ***Negative impact on Mortality***  The study’s findings suggested that the delivery subsidy was associated with an increase in institutional deliveries.  ***Positive impact on healthcare utilization*** |
| Fan, 2018 | China | Observational (Survey) | hospital delivery fees | Target: pregnant women living in rural areas  PROGRESS Plus  Measure: place of residence | Healthcare utilization | Suggests the intervention of RHDS policy had some influence on reducing the gap in hospital delivery rate between rural and urban women.  The study reported a reduction in the gap of hospital delivery after the policy.  ***Positive impact on Healthcare Utilization*** |
| De Allegri 2012 | Burkina Faso | Quasi-experimental  (Controlled before and after) | facility based delivery | Target: pregnant women  PROGRESS Plus  Measure: place of residence | Healthcare utilization | The percentage of women delivering in a health facility increased significantly.  The increase in utilization rates was across all socio-economic groups, leaving existing inequities in access unchanged, as demonstrated by stable significant differences in access.  ***Positive impact on Healthcare utilization*** |
| Mohanty, S. K. 2020 | India | Observational (secondary data analysis) | Not specified | **Target**: pregnant women  PROGRESS Plus  Measure: socioeconomic status | Healthcare utilization | Provision and use of public subsidy for institutional delivery in public health centers is pro-poor in India. Improving the quality of service in primary health centres is recommended to increase utilisation and reduce OOP payment for health care in India.  ***Positive impact on Healthcare utilization*** |
| Garg 2012 | India | Descriptive (case study) | provision of sanitary napkins | **Target group:**  adolescent girls  Place of residence and age | Morbidity | ***Positive Impact***  This scheme will reduce  the incidence and prevalence of RTIs among adolescent girls in the long run. |
| Langlois E., 2016 | Burkina Faso | quasi experimental |  | **Target group:**  Women  **Progress Plus:**  socio-economic status | Healthcare utilization | ***Positive impact on Healthcare utilization***  higher skilled birth attendance rate |
| Ganaba, R; 2016 | Burkina Faso | Descriptive (Case study) | 80 % for all emergency obstetric care – EmOC - including transport in case of referrals, 80 % of uncomplicated deliveries in districts hospitals and health centres and 60 % of uncomplicated deliveries in regional and national hospitals | **Target group:** women who have recently given birth  **Progress Plus:** place of residence | Healthcare utilization  Healthcare expenditure  Quality of care | ***Positive impact on Healthcare utilization***  Deliveries in health facilities have increased since the policy started, especially in rural areas and amongst women from poor households (by 4% from 2007-2010)  After the policy was implemented, between 2007 and 2010, the caesarean rate rose consistently above 1 % for the first time, however this increase was not statistically significant  With respect to trends in non-targeted services such as in medical ward, surgical ward, paediatrics, and outpatients services, the subsidy had very little significant effect  ***Positive impact on health expenditure***  Household payments for facility-based deliveries have reduced significantly, compared with payments before the policy, and the policy as a whole is affordable, costing about 2% of total public health expenditure.  hospitals with the best level of implementation of the subsidy offered higher quality of care |
| Wieser 2018 | Pakistan | Observational (Survey) | Subsidies on fortified complementary foods | **Target group:** 6–23-month-old children  **Progress Plus:** age and socio-economic status | Healthcare utilization  Healthcare expenditure  Morbidity | ***Positive impact on Healthcare utilization***  Price-based interventions with accompanying product information thus seem to be an effective instrument for demand creation but only in the case of sufficiently high price discounts of over 80 %.  ***Positive impact on health expenditure***  Wealthier households were more likely to buy FPCF. The net cost per DALY of the interventions ranged from a return per DALY averted of $US 783 to $US 65. Interventions targeted at poorer households were most cost-effective.  Providing all 6–23-month-old Pakistani children with 75 g of FPCF daily could reduce the DALY due to IoD, IDA and VAD by 41·8 %. |
| Somanathan, A., 2008 | Indonesia | quasi-experimental |  | **Target group:**  Children  **Progress Plus:**  socio-economic status | Healthcare utilization | ***Positive impact on Healthcare utilization***  the healthcard played an important role in protecting health care utilization  in the aftermath of the economic crisis, particularly among very young children (use of public outpatient services and public sector services generally declined far more for children without healthcards, so that the net impact of the healthcard was positive for children who owned healthcards) |
| Coronini-Cronberg 2007 | Thailand | Observational cross-sectional | health services in general | children  socioeconomic status, place of residence | Healthcare utilization | utilization seemed to have increased since the introduction of the 30-Baht Scheme. 52.5% of registered households claimed to have increased their use of health services since the introduction of the scheme **Positive impact** |
| Ferris 2019 | Peru | Observational survey | cervical test | women  socioeconomic status | Implementation considerations | cytology test results were acceptable to most surveyed Peruvian women. we demonstrated that a reimbursement incentive program designed to improve follow-up of cervical cytology test results were acceptable to most surveyed Peruvian women. Of note, our data indicated that even the most vulnerable population, women who spoke Quechua or who were deeply impoverished agreed that they would participate in our proposed incentive program. |
| Bhat 2009 | India | Observational survey |  | Women  socio-economic status, Place of residence | Healthcare expenditure  Quality of care | The Chiranjeevi Scheme has provided financial protection against the cost of delivery and EmOC to the marginalized section of the population. In the study, it was seen that a Chiranjeevi client saves around Rs 3,273 (about US$ 82) in delivery compared to those who did not avail of the benefits of the scheme. However, the scheme is not 100% cost-free to the BPL families as they had to pay outof-pocket expenses for medicines and transportation.  It is heartening to note that most clients of the Chiranjeevi Scheme and non-clients were quite satisfied with delivery-related services. They also reported positive behaviour from the service provider and the staff. This shows that the scheme is able to provide client-pleasing services at almost half of the cost of the regular private-sector charges |
| Ridde 2012  *Reducing the medical cost of deliveries in Burkina Faso is good for everyone, including the poor* | Burkina Faso | Observational cross‐sectional | delivery services  maternal health services  child births | Women  socio-economic status | Healthcare expenditure | Medical expenses for delivery decreased from a median of 4,060 F CFA in 2006 to 900 F CFA in 2010. All categories of the population benefited  from this policy, including the poorest. Yet despite the subsidy, women still carry a significant cost burden; half of them pay  more than they should, and few indigents are fully exempted. The greatest reduction in risk of excessive expenses was seen in women in the  bottom quintile living less than 5 km from the health centers |
| De Allegri 2015  (additional resource) | Burkina Faso | Observational  mixed method | Institutional delivery | women, poor women exempted  socio-economic status | Implementation considerations | the subsidy policy has been a major contributor to this success. Still, our study suggests that many other actions, beyond the mere reduction of user fees, are likely to have contributed to increase the number of facility-based deliveries. health workers, are consistently reported to have promoted facility-based delivery. This finding is suggestive that relationships between health workers and populations, as well as people’s perceptions of quality of care, are important factors in the use of assisted deliveries  **Barriers:** Although point-of-service financial barriers were not directly mentioned by households during the qualitative interviews, the quantitative data identified a clear trend suggesting that poorer households were more likely than wealthier ones to have experienced a home delivery. |
| Belaid 2012  (additional resource) | Burkina Faso | Observational  Qualitative | Institutional delivery | women, poor women exempted  socio-economic status | Implementation consideration | The “implementation gap" analysis has shown that equity and quality of care were neglected and should be taken into consideration. Moreover, it raises interactional factors  that have an influence on the process of implementation. This study has shown the importance of considering power relations between actors representing health system and communities |
| Ridde *2013*  (additional resource*)*  *Why do women pay more than they should? A mixed methods study of the implementation gap in a policy to subsidize the costs of deliveries in Burkina Faso* | Burkina Faso | Observational  mixed method | Institutional delivery | women, poor women exempted  socio-economic status, place of residence | Implementation consideration | The three most plausible explanations for this payment disparity are: (i) the payments were for products used that were not part of the delivery kit covered by the official fee; (ii) the implementers had difficulty in understanding the policy; and (iii) there was improper conduct on the part of some health workers. Institutional design and organizational practices, as well as weak rule enforcement and organizational capacity, need to be considered more carefully to avoid an implementation gap in this public policy. Even if there has been a progressive  distribution of this policy’s benefits, a more rigorous implementation would certainly help to make it even more equitable. (The aim of this study is to test and then (if confirmed) to understand the hypothesis that the amounts paid by women are more than the official fee, i.e., their 20% portion) |
| Ridde 2011  (additional resource)  *The National Subsidy for Deliveries and Emergency Obstetric Care in Burkina Faso* | Burkina Faso | Observational  Qualitative | Institutional delivery | women, poor women exempted  socio-economic status, gender | Healthcare utilization  Implementation consideration | The upward trend in assisted deliveries since 2004 continued after the policy’s introduction.  At the time of the study, fixed-rate reimbursement for delivery (output-based) and overestimation of input costs were financially advantageous to health workers (bonuses)  and management committees (hoarding). Very few of the worst-off have been exempted from payment because selection processes and criteria have not yet been defined and most health workers are unaware of this possibility. Despite relatively tight administrative controls, health workers have figured out how to take advantage  of the system. |
| Dasgupta, 2022 | Nigeria | Economic evaluation (cost-effectiveness analysis) | Subsidy for malaria case management | Children  Socio-economic status | Mortality  (Child mortality) | Targeted subsidization of case management of under-five malaria is a pro-poor intervention that leads to significant reductions in national under-five mortality |
