# Supplementary material for: Financing for equity for women’s, children’s and adolescents’ health in low- and middle-income countries: A scoping review
Source: PLOS Glob Public Health. 2024 Sep 12;4(9):e0003573. doi: 10.1371/journal.pgph.0003573 (PMC11392393; doi:10.1371/journal.pgph.0003573)
Supplement: S7 Table — (DOCX) [file pgph.0003573.s010.docx]

**S7 Table of characteristics: Community-Based Health Insurance (n=15)**

| **Author Year** | **Country** | **Study design** | **Health service covered** | **Target group and PROGRESS Plus**  **measures** | **Outcome(s)** | **Main Results**  **Is the intervention effective overall? (yes/no/inconclusive)** |
| --- | --- | --- | --- | --- | --- | --- |
| Hounton 2012 | Burkina Faso | Experimental (RCTs) | The benefit package included the minimum package of primary care services available in the district including antenatal care, laboratory exams, hospitalization fees, and transportation for emergencies | Target: women  PROGRESS Plus  Measure: socioeconomic status and place of residence | Mortality  healthcare utilization | No significant difference in overall mortality between households who could not have been members, non-members but whose households could have been members and members of the insurance scheme.  ***No impact***  There remains a statistically significant association between membership of the CBHI scheme and the utilization of health services after adjusting for the covariates.  ***Positive impact on*** healthcare utilization |
| Smith 2008 | Sub-Saharan African countries  Senegal, Mali, and Ghana | Experimental  RCTs | maternal health services | Target: women  PROGRESS Plus  Measure: socioeconomic status and place of residence | healthcare utilization  Healthcare expenditure | CBHI scheme is positively associated with the use of maternal health services, particularly in areas where utilization rates are very low and for more expensive delivery-related care.  CBHI membership has a positive effect on utilization of facility-based health care during episodes of illness  Results suggest that CBHI has an impact on reducing out-of-pocket expenditures at the point of service.  ***Positive impact on*** healthcare utilization and Health expenditures |
| Schoeps 2015 | Burkina Faso | Observational (Survey) | medication, laboratory tests, or x-rays ,surgery and in-patient treatment for up to 15 days at the hospital in Nouna | Target: children under 5 years of age  PROGRESS Plus  Measure: socioeconomic status and place of residence | Mortality  healthcare utilization  Healthcare expenditures | The intervention was associated with lowering the risk of mortality in children enrolled in health insurance as compared to the non-enrolled children.  Results suggest that the strong effect of health insurance enrolment on child mortality may be explained by increased utilization of health services by enrolled children.  Insurance enrolment was shown to decrease annual health expenditure.  ***Positive impact on*** Mortality/death rates, healthcare utilization, and Health expenditures |
| Nshakira-Rukundo 2019 | Uganda | Observational (Survey) | not defined | Target: children  PROGRESS Plus  Measure: place of residence | Morbidity | In terms of the distribution of stunting, children in the lowest socioeconomic welfare index had a higher prevalence of stunting.  Results indicate that there was no statistically significant association between CBHI participation and stunting.  ***No impact*** |
| Karra 2016 | low middle income countries | Systematic Review | family planning | Target: women  PROGRESS Plus  Measure: socioeconomic status | healthcare utilization | The CBHI intervention reported a negative impact on the use of family planning and reproductive health services.  ***Negative impact*** |
| Divya 2014 | Burkina-Faso | Observational Survey | not defined | Target:  children  PROGRESS Plus  Measure: socioeconomic status | healthcare utilization | The study reported higher utilization among women with AMBC.  Utilization was more equitable among women with AMBC  than among women without AMBC.  ***Positive impact on*** healthcare utilization |
| Kent Ranson  2006 | India | Observational Survey | not specified | Target: women  PROGRESS Plus  Measure: socioeconomic status & occupation | Healthcare expenditure | The study reported that the high cost of transportation, added to the cost of inpatient care, was enough to prevent poor insured women from going for hospitalization among many other factors such as compiling documents, and submitting the claim to the local Vimo, etc. |
| Lu, C.; 2016 | Rwanda | Observational Secondary data analysis | nutrition services | Target:  Children  PROGRESS Plus  Measure: place of residence | Morbidity | The intervention was associated with a lower probability of being stunted for Mutuelles enrollees, significantly lower than that for uninsured children.  ***Positive impact on*** Morbidity |
| Sinha, T.; 2006 | India | Observational Qualitative study | Under SEWA Insurance’s | Target:  women  PROGRESS Plus  Measure: place of residence | Healthcare expenditure  healthcare utilization | Studies have shown that the health insurance component: (1) provides significant financial protection; (2) appears not to have impacted on rates of hospital utilization; and (3) suffers from inequitable patterns of utilization, by socioeconomic status (in rural areas) and by place of residence (urban versus rural).  Several studies reported that utilization of health care, with the implementation of a CBHI scheme, increases more among insured households located close to the health care facility.  ***Positive impact on*** healthcare utilization and Health expenditures |
| Oraro, T, 2018 | Cameroon | Observational (Primary Study- Survey) | The Bamenda Ecclesiastical Provincial Health Assistance (BEPHA) scheme is a micro health insurance scheme set up by the Roman Catholic Church in North-West and South-West Cameroon | Target Group: women  Progress Plus component: Gender and Place of residence | Implementation consideration | health insurance enrolment amongst women is correlated with the need to minimize potential household health risks based on their direct knowledge of household healthcare needs. Our findings highlight the need for partnerships between health insurance schemes and governments in order to develop a financial safety net to limit the impact of illness on the poor. |
| Nshakira-Rukundo 2020  *Impact of voluntary community-based health insurance on child stunting: Evidence from rural Uganda* | Uganda | Observational (cross-sectional study) | Not specified | Children  place of residence | Morbidity  Healthcare utilization | We find that one year of a  household's participation in community-based health insurance was associated with a 4.3 percentage point less probability of stunting.  **Positive impact**  The expansion of community-based health insurance might have more dividends to improving health, in addition to financial protection and service utilization in rural developing countries. |
| Simieneh 2021 | Ethiopia | Observational (Cross-sectional) | not specified (febrile illness in children in this study) | under-five children in this study  socio-economic status, place of residence | Healthcare utilization | This study revealed that mothers` CBHI membership was significantly associated with the healthcare-seeking behavior (HSB) for childhood  illnesses and significantly affected HSB during childhood. This study indicated that being a member of the CBHI scheme contributed a 28.70% increase to HSB compared with non-members of the CBHI scheme using the Nearest Neighbor matching method at a 5% level of significance. This result  is consistent with a study conducted in Tanzania and Ghana. It is also compatible with the survey conducted  in Vietnam that indicates insured patients are more likely to seek healthcare services in terms of outpatient facilities  **positive impact** |
| Koch, 2022 | Rwanda | Observational (Cross-Sectional) |  | women ≥18 years who underwent c-section  Place of Residence | Healthcare expenditure | Even if medical costs and transportation are fully subsidized, there will still be a low rate of Catastrophic Health Expenditure (CHE) (3–4%) for impoverished patients. Not surprisingly, though, we found that when a higher percentage of costs are covered by insurance or by the government, far fewer patients are in danger of CHE. Consequentially, in the current system, the poorest patients who are fully subsidized with free care are better protected, while those in the next tier are more vulnerable to financial catastrophe.  **Positive impact** |
| Handebo, 2023 | Ethiopia | Observational  Cross-sectional |  | Women  Place of residence, and education | Implementation considerations | The overall CBHI enrollment among reproductive-age women in Ethiopia was relatively low. Especially among those residing in rural areas and having lower education level. Thus, improving reproductive-age women’s understanding of the CBHI system developing their trust in it, and enabling them recognize its valuable benefits would increase the enrollment of this population in the CBHI scheme. |
| Mussa, 2023 | Ethiopia | Observational  (Secondary data analysis) |  | Women  Socio-economic status | Healthcare utilization | we find no significant effects of community-based health insurance membership on utilization of maternal and child healthcare services. This may be due to the free availability of such services for everyone at the public health facilities, regardless of insurance membership  **No effect** |
