# Supplementary material for: Financing for equity for women’s, children’s and adolescents’ health in low- and middle-income countries: A scoping review
Source: PLOS Glob Public Health. 2024 Sep 12;4(9):e0003573. doi: 10.1371/journal.pgph.0003573 (PMC11392393; doi:10.1371/journal.pgph.0003573)
Supplement: S8 Table — (DOCX) [file pgph.0003573.s011.docx]

**S8 Table of characteristics: Performance-based financing (n=10)**

| **Author, Year** | **Country** | **Study design** | **Health service covered** | **Target group and PROGRESS Plus**  **measures** | **Outcome(s)** | **Main Results**  **Is the intervention effective overall? (yes/no/inconclusive)** |
| --- | --- | --- | --- | --- | --- | --- |
| Koulidiati 2021 | Burkina Faso | Quasi-experimental | curative care, child vaccination and growth monitoring visits | Target: Under-five children  PROGRESS Plus  Measure: place of residence | Healthcare utilization  Quality of Care | The study failed to detect any significant effects of PBF on effective coverage for curative child health services in Burkina Faso.  ***No impact***  The study did not find any significant effects on quality of care.  ***No impact*** |
| Skiles 2015 | Rwanda | Quasi-experimental | Child health services | Target: Under-five children  PROGRESS Plus  Measure: socioeconomic status | Morbidity  Quality of care  Healthcare utilization | No evidence to support the hypothesis that PBF districts experienced decreased morbidity from diarrhea, fever, or  symptoms of ARI relative to the comparison districts.  ***No impact***  PBF improved the quality of treatment received by poor children conditional on patients seeking care  ***Positive impact***  No increase in care  seeking for routine childhood illness under the supply-side incentive program  ***No impact*** |
| Sieleunou 2020 | Cameroon | Experimental (RCT) | Maternal and child health services | Target: Women and children  PROGRESS Plus  Measure: place of residence | Other outcomes  *Resources availability* | The PBF intervention had no effect on the stock-outs of antenatal care drugs, vaccines, integrated management of childhood illness drugs and labor and delivery drugs.  ***No impact***  The intervention was associated with a significant reduction of 34% in stock-outs of family planning medicines.  ***Positive impact*** on family planning drugs |
| Korachais 2020 | Burundi | Experimental (RCT) | Nutrition health services | Target: malnourished children  PROGRESS Plus  Measure: place of residence | Morbidity | Prevalence of chronic malnutrition among children aged 6–23  months remained high, above 50%.  PBF-N had no significant impact on neither chronic nor acute prevalence rates.  ***No impact*** |
| Lannes 2016 | Rwanda | Experimental (RCT) | Family planning and maternal and child health services | Target: women aged 15–49 years, women who were pregnant in the 2 years preceding the survey & children up to 5 years of age  PROGRESS Plus  Measure: socioeconomic status and place of residence | Healthcare utilization | Results suggest that PBF has an impact on increasing institutional deliveries and family planning, but not on prenatal care services. However, a positive impact was observed on institutional deliveries and family planning for the upper group only.  ***No impact on the poor***  PBF showed no impact on the probability of a child getting curative care. However, a positive impact was found for both wealth groups for preventive care.  ***No impact on curative care, positive impact only for preventive care*** |
| Van de Poel 2016 | Cambodia | Observational (Secondary data analysis) | Vaccinations, institutional delivery and antenatal care | Target: pregnant women  PROGRESS Plus  Measure: socioeconomic status | Healthcare utilization | There was an increase in the probability of a child being born in a public facility however there was is no significant effect on the probability of the poorest women delivering in a public facility.  ***No impact on the poor***  There is still no significant effect on the rate of vaccinations or antenatal care.  ***No impact*** |
| Mwase 2020 | Burkina Faso | Experimental (RCT) | Family planning and maternal health services | Target: pregnant women  PROGRESS Plus  Measure: socioeconomic status | Healthcare utilization | There was a positive effect of PBF on utilization of facility-based delivery and for PNC3+ visits primarily among the upper wealth group. Among the poorest 20%, there was an increase attributable to PBF for utilization of modern family planning methods.  The equity measures that accompanied the implementation of  PBF did not result in any additional benefit for the poorest  20%, but rather the opposite on certain indicators.  ***No impact on the ultra-poor*** |
| Brenner 2021 | Malawi | Quasi-experimental  (non-randomized controlled pre-post-test study) | maternal and newborn services | Target: women and newborns  PROGRESS Plus: place of residence: | Quality of care  *The RBF4MNH design did not directly incentivize ANC or any other maternal care services within the continuum. This particular scheme therefore provides an opportunity to explore whether performance-based incentives to a particular group of healthcare providers, namely midwives attached to a defined set of clinical services, namely facility-based childbirth, might have external benefits to other non-incentivized maternal care services* | In this study, we did not find evidence that the *Results-Based Financing For Maternal and Newborn Health* (RBF4MNH) scheme produced either positive or negative externalities on ANC service provision. This is relevant in so far, as our findings neither suggest an improvement nor a neglect of non-incentivized services provided by the same health worker cadre.  Increased utilization of PBF supported delivery services further **increased the workload of maternal care workers at these** facilities beyond capacity. This unintended erosion of workload capacity might not only have limited the RBF4MNH’s actual potential on improving incentivized processes of care, but also its potential to produce substantial externalities in related care services  Existing evidence related to RBF4MNH implementation suggests significant positive effects of the scheme on the clinical quality of childbirth care, mostly by increasing health workers’ adherence to technical standards as well as through improved supply chain management  **Positive impact** |
| Nkangu 2022 | Cameroon | Observational (qualitative study) | maternal services | Women  socioeconomic status | Quality of care  Implementation consideration | The key findings of this study indicate that introducing a PBF equity strategy has positively impacted the overall motivation of health providers (doctors, nurses, and midwives) in delivering care to those considered poor.  **Positive impact**  a delay in the payment of PBF  incentives was reported to be the main challenge that had a negative relationship with the consistent  provision of care to the poor and vulnerable, especially in private health facilities.  **Barrier**  The irregularity of the PBF payment mechanism shapes how providers engage in sustaining aspects of the PBF equity strategy, and the motivation and quality of services are likely to decrease if the payment mechanism is not addressed. Objective identification of the PAV is critical for proper classification, and meeting the 10% threshold by health facilities is important in increasing the utilization of services by the poor. The PBF equity strategy provides for the PAV; however, implementation challenges in identifying and classifying the PAV can affect the outcome. |
| Anselmi, 2023 | Mozambique | Observational  Cross-sectional | Maternal and child health services | women  Socio-economic status | Healthcare utilization  (HIV testing during ANC)  Other outcome  (health knowledge) | HIV testing during ANC increased, particularly for wealthier and more educated women, and in Gaza.  **Positive effect for those who are wealthier and more educated (inequity)**  Knowledge about MTCT (mother-to-child transmission) and PMTCT (prevention of mother-to-child transmission) also improved, particularly for less educated women  **More equity** |
