# Supplementary material for: Financing for equity for women’s, children’s and adolescents’ health in low- and middle-income countries: A scoping review
Source: PLOS Glob Public Health. 2024 Sep 12;4(9):e0003573. doi: 10.1371/journal.pgph.0003573 (PMC11392393; doi:10.1371/journal.pgph.0003573)
Supplement: S9 Table — (DOCX) [file pgph.0003573.s012.docx]

**S9 Table of characteristics: UCT (n=7)**

| **Author Year** | **Country** | **Study design** | **Health service covered** | **Target group and PROGRESS Plus**  **measures** | **Source of funding and providers** | **Outcome(s)** | **Main Results**  **Is the intervention effective overall? (yes/no/ inconclusive)** |
| --- | --- | --- | --- | --- | --- | --- | --- |
| Pega 2022 | LMIC | Systematic review | health services in general | **Target**: children and adults in LMICs  **PROGRESS Plus:** socio-economic status | Funder: Not specified  Provider: Not specified | Healthcare utilization  healthcare expenditure  Morbidity  Other outcomes  *nutrition* | This body of evidence suggests that unconditional cash transfers may not impact health services use among children and adults in low- and middle-income countries.  **No impact**  Unconditional cash transfers probably or may improve:  healthcare expenditure  **moderate and low-certainty evidence**  cash transfers (UCTs) have probably had a large, clinically meaningful, beneficial effect on the likelihood of having had any illness  Positive  Unconditional cash transfers probably or may improve:  some health outcomes (such as the likelihood of having secure access to food, and diversity in one's diet) |
| Handa 2016 | Zambia | Experimental  (RCT) | maternal and child health services: antenatal care, skilled attendance at birth | Target: pregnant women and children  PROGRESS Plus  Measure: place of residence | Funder: government  Provider: government | Healthcare utilization | some positive impact was noticed in regards to better access to maternal health services, specifically skilled attendance at birth.  ***Positive impact on utilization but not evidence of impact on health outcomes*** |
| Sibson 2018 | Niger | Experimental  (RCT) | N/A | **Target**: children  PROGRESS Plus  Measure: place of residence | Funder: not defined  Provider: not defined | Morbidity | The modified UCT plus 4 months supplementary  feeding did not reduce the prevalence of Global Acute Malnutrition compared with the standard UCT plus 4 months supplementary feeding  No effect |
| Tonguet-Papucci 2017 | Burkina Faso | Observational  Qualitative | N/A | **Target**: children  **PROGRESS Plus**  **Measure**: socioeconomic status | Funder: international organization  Provider: international organization | Healthcare expenditure  Other outcomes  *Nutrition*  *stress* | the availability of UCT supported families in spending more on healthcare to support the child’s health  improvement in the quality of the child’s diet and to increase the overall food stores in the house  cash transfers  reduced the stress and shame that occur when the family is hungry |
| Houngbe 2017 | Burkina Faso | Experimental  (RCT) | N/A | **Target**: children  **PROGRESS Plus**  **Measure**: socioeconomic status | Funder: international organization  Provider: international organization | Morbidity | No evidence that multiannual, seasonal UCTs reduced the cumulative incidence of wasting in young children.  No effect  No significant difference in children’s anthropometric measurements and stunting  Inconclusive |
| Grellety 2017 | Congo | Experimental  (RCT) | N/A | **Target**: children  PROGRESS Plus  Measure: socioeconomic status | Funder: international organization  Provider: international organization | Morbidity | the hazard ratio of reaching full recovery from severe acute malnutrition was 35% higher in the intervention group than the control group. Positive impact was seen after 6 months where children re-gained their mid-upper arm circumference measurements and weight-for-height/length Z-scores  Positive |
| Briaux 2020 | Togo | Experimental  (RCT) | package of community activities (including behavior change communication sessions, home visits, and integrated community case management of childhood illnesses and acute malnutrition  delivered to mother–child pairs during the first “1,000 days” of life. | Target: mothers and children  PROGRESS Plus  Measure: place of residence | Funder: international organization (World Bank and UNICEF)  Provider: government | Child development | UCTs had a protective effect on child’s linear growth in rural areas of Togo.  ***Positive impact*** |
