# Supplementary material for: Financing for equity for women’s, children’s and adolescents’ health in low- and middle-income countries: A scoping review
Source: PLOS Glob Public Health. 2024 Sep 12;4(9):e0003573. doi: 10.1371/journal.pgph.0003573 (PMC11392393; doi:10.1371/journal.pgph.0003573)
Supplement: S10 Table — (DOCX) [file pgph.0003573.s013.docx]

**S10 Table of characteristics: Introduction of user fees (n=5)**

| **Author Year** | **Country** | **Study design** | **Health service covered** | **Target group and PROGRESS Plus**  **measures** | **Source of funding and providers** | **Outcome(s)** | **Main Results**  **Is the intervention effective overall? (yes/no/inconclusive)** |
| --- | --- | --- | --- | --- | --- | --- | --- |
| Malama 2002 | Zambia | Observational (cross-sectional study) | Hospital care | **Target:**  children 3–6 years of age  **PROGRESS** **Plus**  **Measure**: place of residence and gender | Funder: Government  Provider: Government | Healthcare utilization | *“User fees appear to decrease differentially utilization of inpatient care for female children in rural Zambia”.*  ***Negative impact*** |
| Bratt 2002 | Ecuador | Experimental RCT | family planning and reproductive health services (contraceptive methods, hormonal and barrier methods, sterilization,  gynecological care, pre and post-natal visits and pediatric consultation) | **Target**: women  **PROGRESS** **Plus**  **Measure**: socioeconomic status (poor) | Funder: out of pocket  Provider: CEMOPLAF(a private non for profit agency operated 12 fp/rh clinics, and community based distribution , and Social marketing programs , | Healthcare utilization | *No evidence that the price increases (at the levels tested) had a disproportionate impact on utilization by poorer clients.*  *No impact* |
| Degregorio 2017 | Cameroon | Observational  Retrospective medical records review | cervical cancer screening | **Target**:  women  PROGRESS Plus  Measure: place of residence (rural) | Funder: fee for service and external findings  Provider: The Cameroon Baptist Convention Health Services (CBCHS) ( a nonprofit, faith-based health care) | healthcare utilization | The Women’s Health Program (WHP), despite charging fees, has experienced an increase in patient volume for cervical cancer screening nearly every year since its establishment in 2007.  The WHP is sustained primarily on fees-for-service, with external funding.  ***Positive impact*** |
| Johnson 2012 | Mali | Observational (Qualitative study (ethnographic) | Malaria treatment | **Target group:**  Women  **Progress plus:** Gender, socioeconomic status | **Funding:**  Government  **Providers:** government | Healthcare utilization | *User fees for health care not only decreased utilization of health services, but also resulted in delayed presentation for care, incomplete or inadequate care, compromised food security and household financial security, and reduced agency for women in health care decision making.*  ***Negative Impact*** |
| Pot 2018 | Malawi | Observational Qualitative study | maternal health services | **Target**: women  **PROGRESS Plus**  **Measure**: place of residence | Funder: Government  Provider: Government | Healthcare utilization  Implementation considerations | Data from the health centre indicated that the number of skilled deliveries and ANC visits decreased by approximately 50% immediately after the reintroduction of user-fees  *“Different actors are frustrated about user fees and their impact on the poor.”*  *“User-fees eroded trust between women and health workers.”*  ***Negative impact*** |
