# Supplementary material for: Financing for equity for women’s, children’s and adolescents’ health in low- and middle-income countries: A scoping review
Source: PLOS Glob Public Health. 2024 Sep 12;4(9):e0003573. doi: 10.1371/journal.pgph.0003573 (PMC11392393; doi:10.1371/journal.pgph.0003573)
Supplement: S11 Table — (DOCX) [file pgph.0003573.s014.docx]

**S11 Table of characteristics: Microcredit (n=2)**

| **Author Year** | **Country** | **Study design** | **Interventions** | **Health service covered** | **Target group and PROGRESS Plus measures** | **Outcome(s)** | **Main Results**  **(yes/no/inconclusive)** |
| --- | --- | --- | --- | --- | --- | --- | --- |
| Adams, 2013 | Bangladesh | Descriptive case study | **Microcredit** |  | Women  socioeconomic status | Mortality | Evidence suggests various positive effects of microcredit on health  outcomes such as child mortality |
| You 2016 | China | Quasi -experimental | **Microcredit** |  | Target: Children  PROGRESS plus: Place of residence and socio-economic status | Healthcare expenditure | Moreover, formal microcredit appears to be a complement to health insurance in  improving child health through two mechanisms—it enhances affordability for out-of-pocket health care expenditure and helps  buffer consumption against adverse health shocks and financial risk incurred by current health insurance arrangements. Government efforts in expanding health insurance for rural households would be more likely to achieve its optimal goals of improving child health outcomes if combined with sufficient access to formal microcredit.  **Positive impact** |
